# Exploiting thiol-functionalized benzosiloxaboroles for achieving diverse substitution patterns – synthesis, characterization and biological evaluation of promising antibacterial agents†

**DOI:** 10.1039/d4md00061g

**Published:** 2024-03-20

**Authors:** Krzysztof Nowicki, Joanna Krajewska, Tomasz M. Stępniewski, Monika Wielechowska, Patrycja Wińska, Anna Kaczmarczyk, Julia Korpowska, Jana Selent, Paulina H. Marek-Urban, Krzysztof Durka, Krzysztof Woźniak, Agnieszka E. Laudy, Sergiusz Luliński

**Affiliations:** a Faculty of Chemistry, Warsaw University of Technology Noakowskiego 3 00-664 Warsaw Poland krzysztof.nowicki2.dokt@pw.edu.pl sergiusz.lulinski@pw.edu.pl; b Department of Pharmaceutical Microbiology and Bioanalysis, Medical University of Warsaw Banacha 1b 02-097 Warsaw Poland alaudy@wp.pl; c GPCR Drug Discovery Lab, Research Programme on Biomedical Informatics (GRIB), Hospital del Mar Medical Research Institute (IMIM) – Department of Medicine and Life Sciences, Pompeu Fabra University (UPF) Carrer del Dr. Aiguader, 88 08003 Barcelona Spain; d Faculty of Chemistry, University of Warsaw Pasteura 1 00-093 Warsaw Poland

## Abstract

Benzosiloxaboroles are an emerging class of medicinal agents possessing promising antimicrobial activity. Herein, the expedient synthesis of two novel thiol-functionalized benzosiloxaboroles 1e and 2e is reported. The presence of the SH group allowed for diverse structural modifications involving the thiol-Michael addition, oxidation, as well as nucleophilic substitution giving rise to a series of 27 new benzosiloxaboroles containing various polar functional groups, *e.g.*, carbonyl, ester, amide, imide, nitrile, sulfonyl and sulfonamide, and pendant heterocyclic rings. The activity of the obtained compounds against selected bacterial and yeast strains, including multidrug-resistant clinical strains, was investigated. Compounds 6, 12, 20 and 22–24 show high activity against *Staphylococcus aureus*, including both methicillin-sensitive (MSSA) and methicillin-resistant (MRSA) strains, with MIC values in the range of 1.56–12.5 μg mL^−1^, while their cytotoxicity is relatively low. The *in vitro* assay performed with 2-(phenylsulfonyl)ethylthio derivative 20 revealed that, in contrast to the majority of known antibacterial oxaboroles, the plausible mechanism of antibacterial action, involving inhibition of the leucyl-tRNA synthetase enzyme, is not responsible for the antibacterial activity. Structural bioinformatic analysis involving molecular dynamics simulations provided a possible explanation for this finding.

## Abstract 2

A series of new benzosiloxaboroles with diverse substitution patterns were synthesized from simple SH-substituted precursors followed by comprehensive characterization and evaluation as promising antibacterial agents.

## Graphical abstract

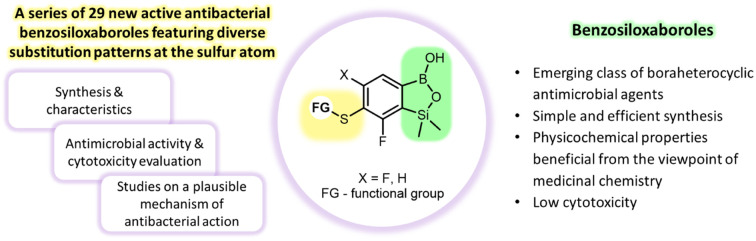

## Introduction

**Fig. 1:**
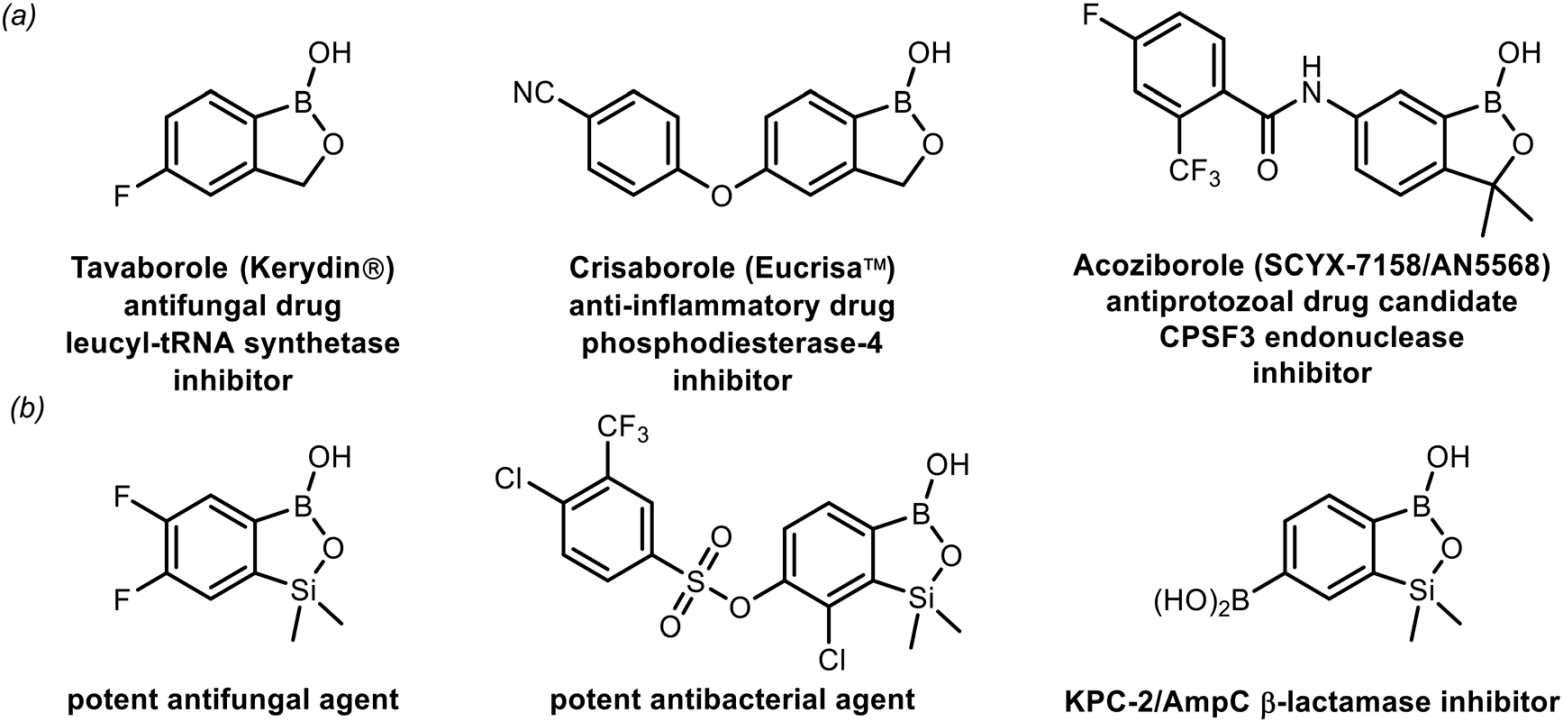
(a) Examples of biologically active benzoxaboroles already introduced into clinical use (both marketed and under clinical trials); (b) examples of benzosiloxaboroles showing antimicrobial activity.

In the last two decades, strong interest in boron heterocycles was stimulated to a significant extent by the discovery of the biological activity of benzoxaboroles – internal hemiesters of 2-(hydroxymethyl)phenylboronic acid.^1–6^ They emerged as a novel class of small-molecule therapeutic agents possessing strong antimicrobial,^7–11^ anti-inflammatory^12,13^ as well as anti-cancer activity.^14–16^ Recent intensive efforts resulted in the preparation of over 10 000 benzoxaborole derivatives. So far, two of them have been successfully commercialized,^17,18^ while several others are in various phases of clinical trials (Fig. 1a).^19–22^ Recently, our group has given attention to silicon analogues of benzoxaboroles–benzosiloxaboroles.^23–28^ The introduction of the SiMe_2_ group to the oxaborole ring in place of the methylene group resulted in increased Lewis acidity and lipophilicity, which may be beneficial for biological activity.^23^ Moreover, the strategy for the synthesis of these compounds is different, which opens up possibilities for enriching substitution patterns at the boracyclic scaffold.^23–27,29^ Our preliminary microbiological studies show that simple fluorinated benzosiloxaboroles are active against selected yeast strains,^23^ whereas more extended systems demonstrate potent antibacterial activity, especially against Gram-positive cocci, including multidrug-resistant clinical strains.^26,27^ Some derivatives were also found to be effective inhibitors of KPC-2/AmpC β-lactamases responsible for drug resistance in bacteria (Fig. 1b).^25^

**Fig. 2:**
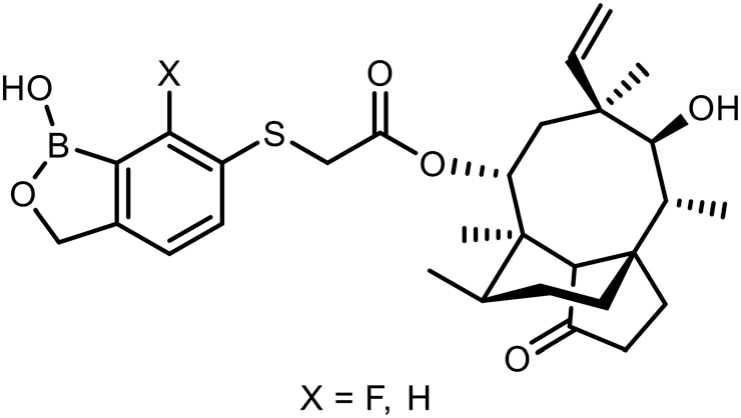
Pleuromutilin-functionalized benzoxaboroles obtained from respective thiol precursors.^32^

The sulfide group is widely used in medicinal chemistry as a linker and many benzoxaboroles containing this moiety have been already reported.^30,31^ However, thiol-functionalized benzoxaboroles themselves have not been widely exploited so far. Only two *S*-functionalized benzoxaboroles were synthesized through direct transformation of a thiol group at the benzoxaborole (Fig. 2). These compounds were investigated as potential anti-*Wolbachia* agents, however they did not exhibit significant potency.^32^ Considering the growing potential of benzosiloxaboroles in medicinal chemistry we decided to extend the library of these compounds by utilization of a thiol substituent. The proposed general concept is practical due to the high reactivity of the thiol group, which can be easily converted into other sulfur-based groups, such as thioether, thioester, sulfonamide, *etc.* Thus, it was successfully validated by synthesis of 27 new functionalized benzosiloxaboroles followed by comprehensive evaluation of antimicrobial activity and cytotoxicity. This work was complemented by a study on the plausible mechanism of action of one of the obtained compounds.

## Results and discussion

### Synthesis

**Scheme 1:**
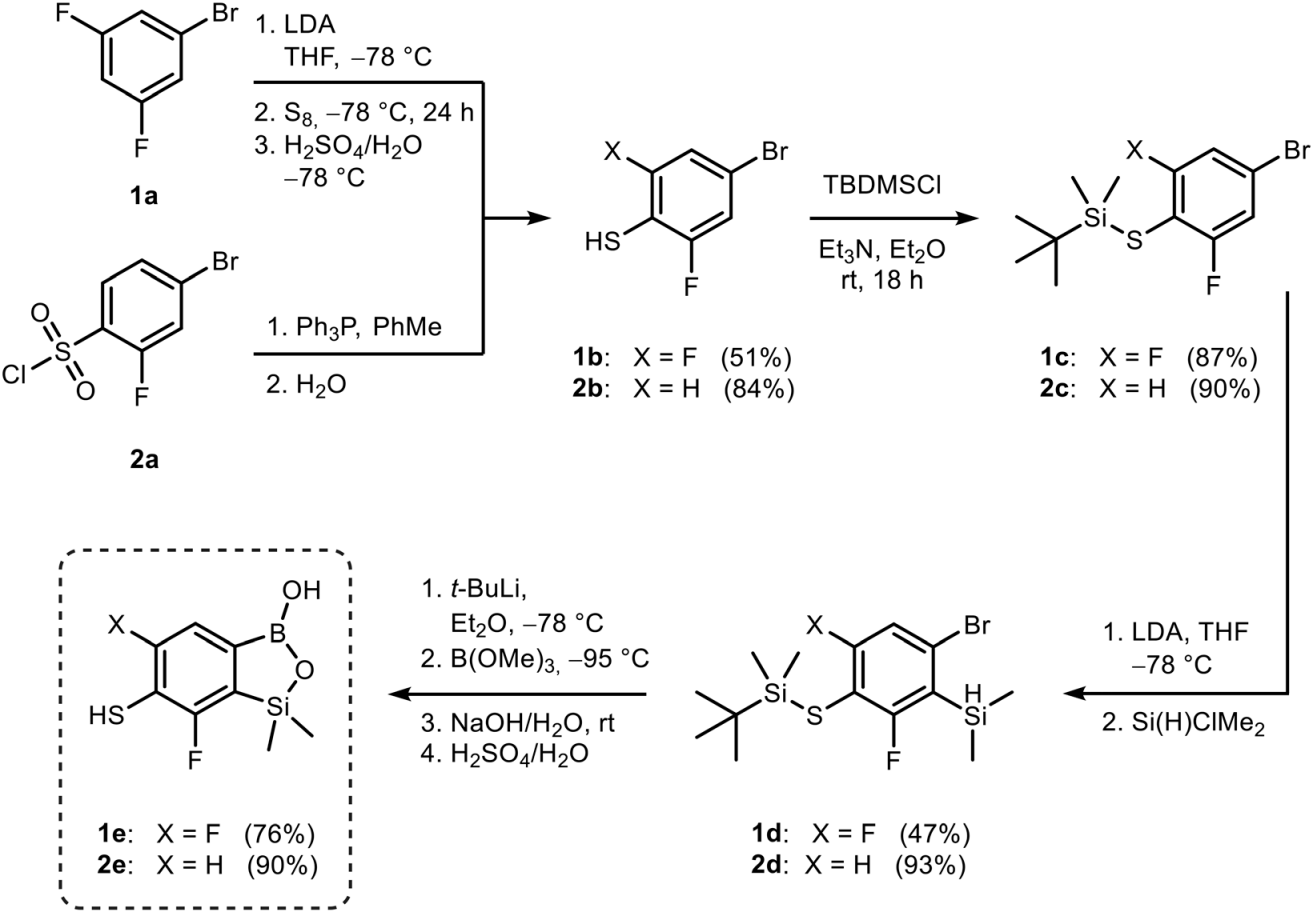
Synthesis of thiol-functionalized fluorinated benzosiloxaboroles 1e and 2e.

The approach to fluorinated thiol-functionalized benzosiloxaboroles 1e and 2e involved a general four-step protocol (Scheme 1) starting with the preparation of appropriate halogenated thiophenols from inexpensive starting materials. The synthesis of thiophenol 1b was accomplished through deprotonative lithiation of 1-bromo-3,5-difluorobenzene with LDA in THF at −78 °C followed by addition of sulfur and hydrolytic workup. The respective disulfide 1b**_D** was also formed as a byproduct (*ca.* 5%). Notably, when the reaction mixture was warmed to room temperature prior to hydrolysis, bis((4-bromo-2,6-difluorophenyl)thio)methane 1b**_CH2** was formed to a significant extent (ESI, Scheme S1). The structures of 1b**_D** and 1b**_CH2** were confirmed by single-crystal X-ray diffraction. The extensive formation of 1b**_CH2** is intriguing but not fully clear. It was rationalized by the reaction of 1b**_D** with lithium enolate formed from LDA-induced cleavage of THF according to the mechanism proposed for a similar transformation^33^ (ESI,† Scheme S2). Thiophenol 2b was synthesized by the reduction of 4-bromo-2-fluorobenzene-1-sulfonyl chloride with PPh_3_. Compounds 1b and 2b were converted to respective TBDMS thioethers 1c and 2c which were subjected to deprotonation with LDA in THF at −78 °C followed by trapping of corresponding aryllithium intermediates with chlorodimethylsilane.^23^ In both cases, the reaction occurred regioselectively at the position between fluorine and bromine atoms in agreement with a strong cumulated *ortho*-acidifying effect of those two halogen substituents.^34,35^ Finally, the conversion of functionalized arylsilanes 1d and 2d to respective benzosiloxaboroles 1e and 2e was performed as described by us previously.^23,25^ It involved a Br/Li exchange reaction using *t*-BuLi in Et_2_O and subsequent boronation of resultant aryllithiums with B(OMe)_3_ at very low temperatures (≤95 °C) followed by hydrolysis. The simultaneous Si–H bond cleavage and deprotection of the thiol group was cleanly performed under alkaline conditions. After acidification, 1e and 2e were isolated as white powders.

The incorporation of various side chains into the structure of benzosiloxaboroles was achieved through the thiol-Michael addition reaction with 1e and 2e as effective *S*-nucleophiles (Table 1). The thiol-Michael addition is broadly applicable and usually proceeds under mild conditions; moreover, it can be regarded as a “click chemistry” method owing to 100% atom economy.^36,37^ The syntheses involving selected Michael acceptors proceeded smoothly under relatively mild conditions (temperature range from 0 to 25 °C) and resulted in preparation of 19 *S*-linked functionalized benzosiloxaboroles with good yields (>70%).

**Table 1:** Synthesis of benzosiloxaboroles 3–21*via* thiol-Michael addition reaction

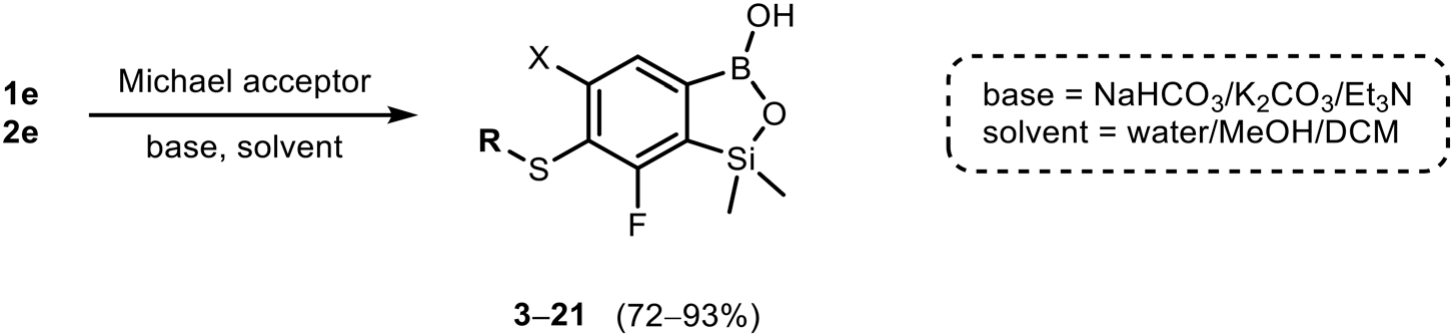					
Compound	X	R	Michael acceptor	Base	Yield, %
3	F	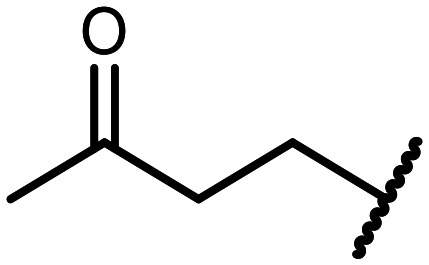	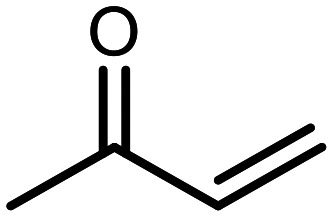	—	90
4	H	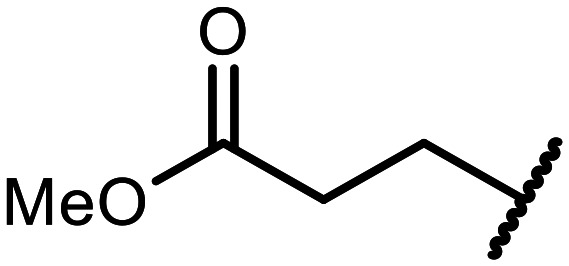	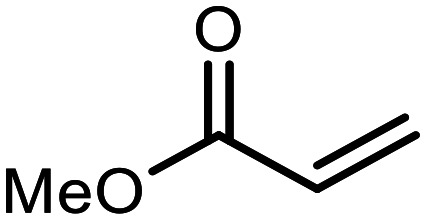	NaHCO_3_	93
5	F	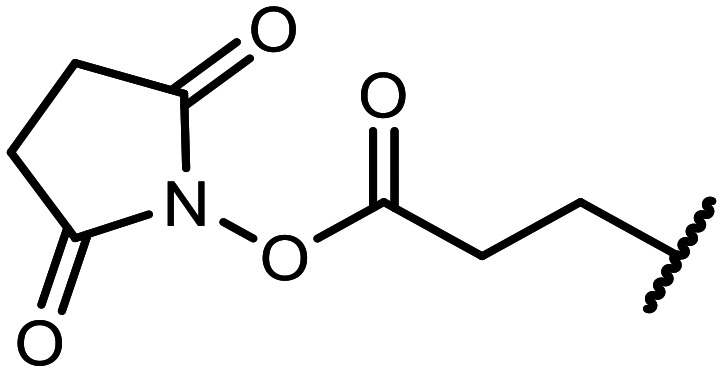	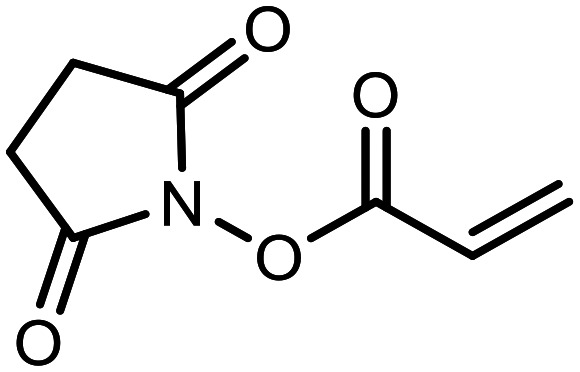	Et_3_N	79
6	F	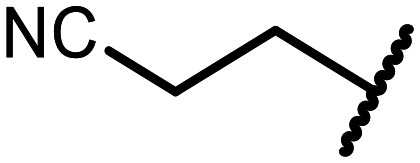	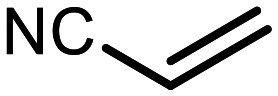	K_2_CO_3_	89
7	F	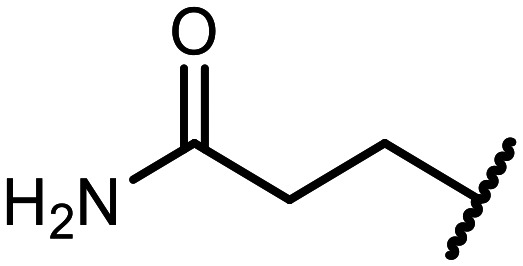	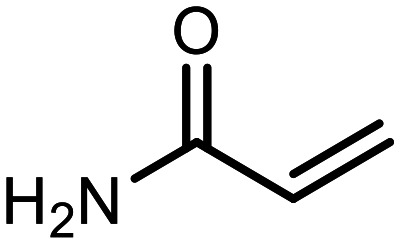	K_2_CO_3_	80
8	F	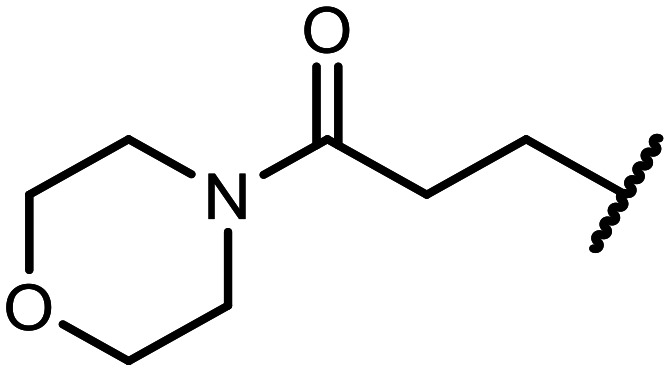	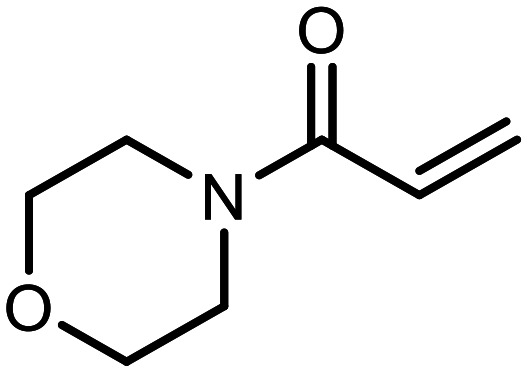	K_2_CO_3_	90
9	F	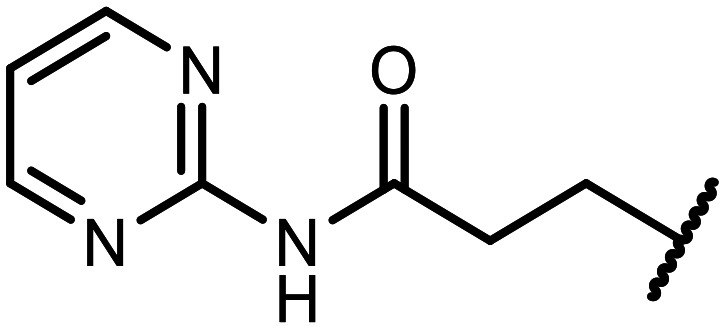	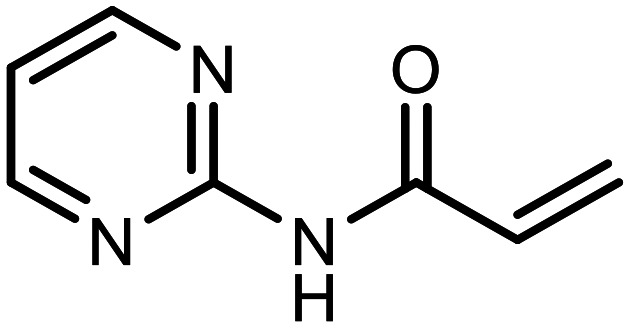	K_2_CO_3_	82
10	F	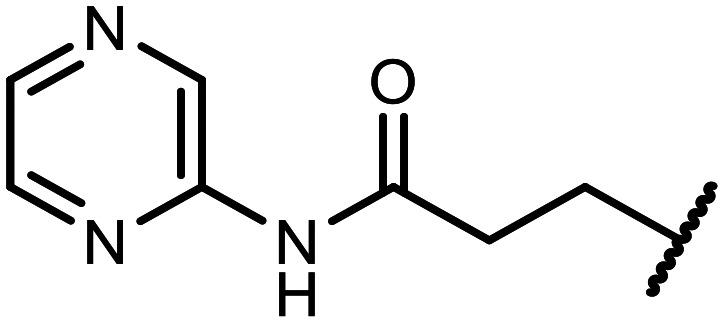	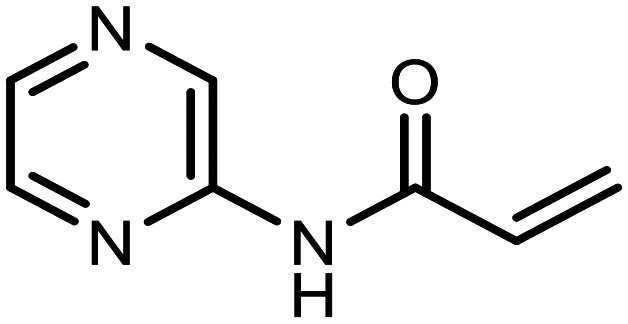	K_2_CO_3_	86
11	F	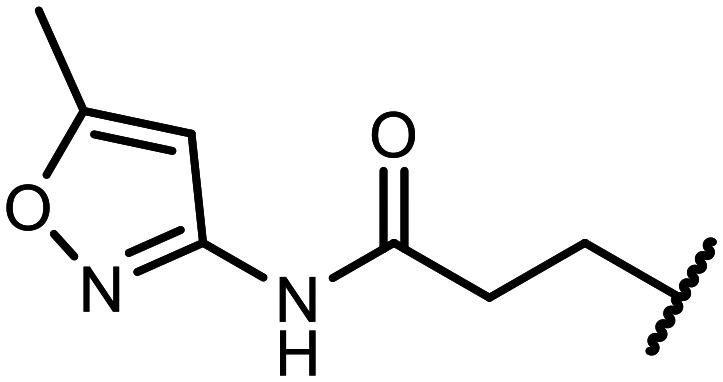	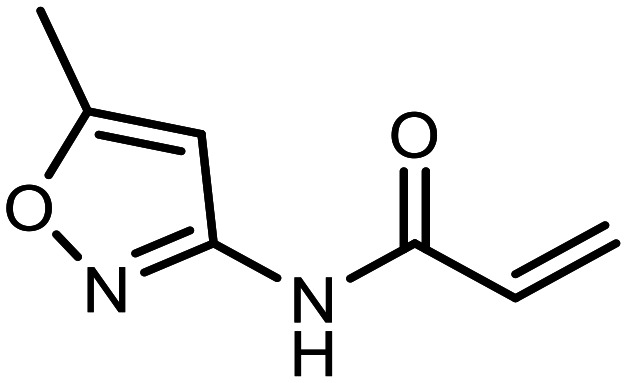	K_2_CO_3_	75
12	F	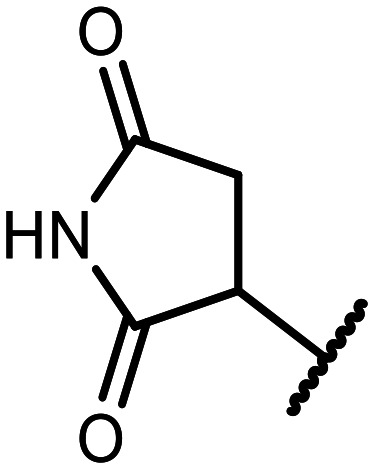	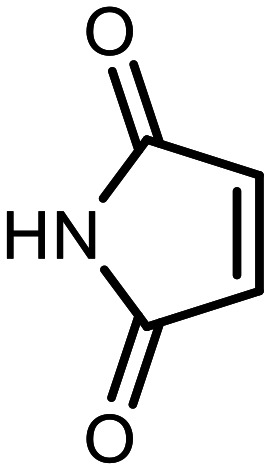	K_2_CO_3_	87
13	H				90
14	F	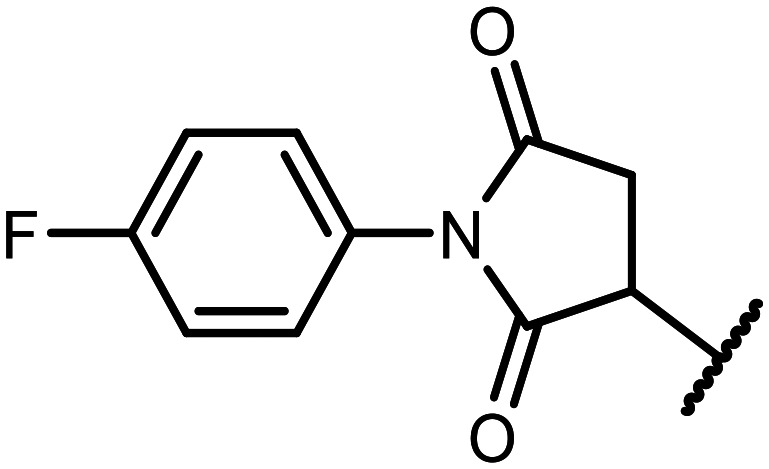	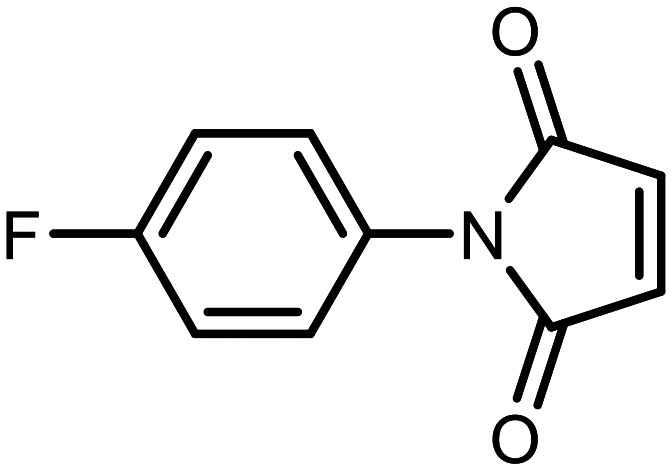	Et_3_N	83
15	H				85
16	F	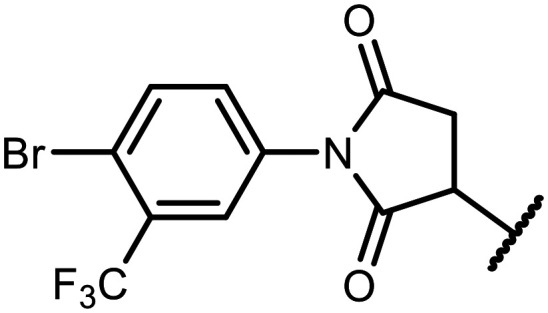	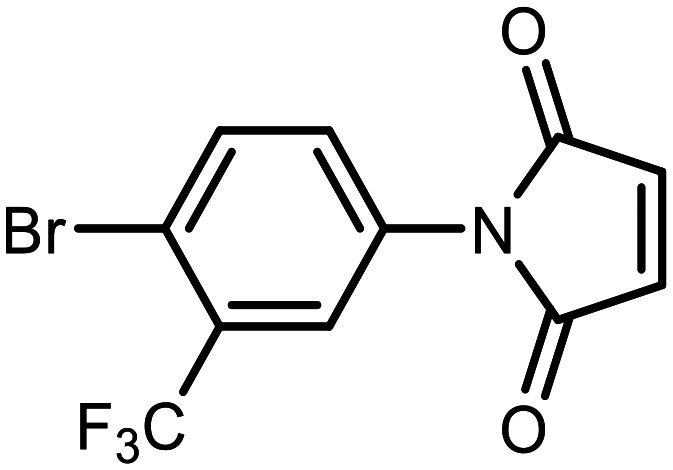	Et_3_N	79
17	H				85
18	F	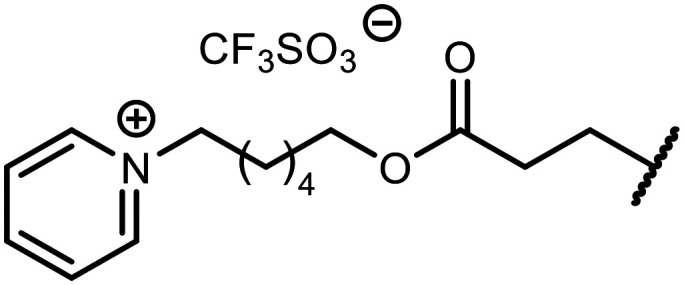	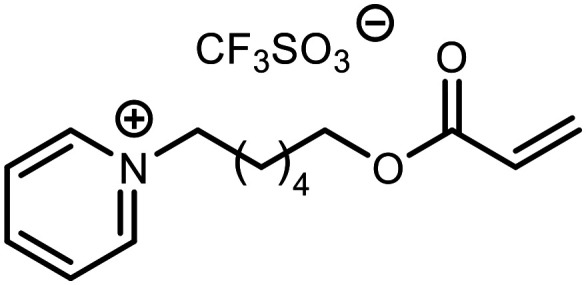	K_2_CO_3_	83
19	H				79
20	F	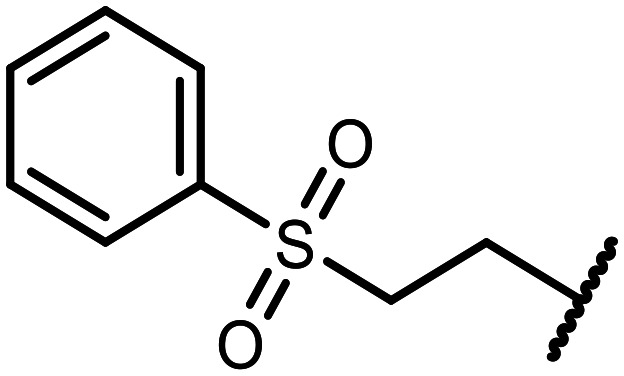	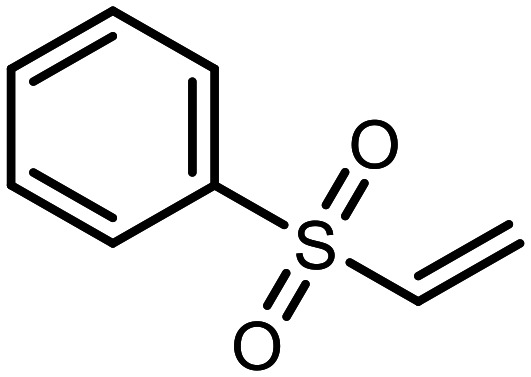	K_2_CO_3_	81
21	H				72

In the case of compound 3, the reaction occurred in water without the need for the use of a base. All other syntheses required the use of a base to generate more effectively active anionic forms of 1e and 2e. The wide representation of Michael acceptors used in the syntheses of benzosiloxaboroles 3–21 includes α,β-unsaturated ketones, esters, nitriles, amides, imides and sulfones. Reactions leading to compounds 3, 4, 6, 7, 12, 13, 20 and 21 utilized readily available substrates. Protocols for the preparation of the Michael acceptors used in the syntheses of 5, 9–11, 14–19 are available in the ESI.† We assumed that the introduction of pendant substituents containing various polar end groups to the benzosiloxaborole may result in specific interactions with targeted biomolecules which would be beneficial for antibacterial activity. Moreover, benzosiloxaboroles 7–12 containing an amide group constitute an interesting group as it seems that bioconjugates of benzosiloxaboroles with amino acids or peptides could be obtained analogously. Benzosiloxaboroles 12–17 possess a pendant succinimide ring where the presence of a nitrogen atom enables further functionalization. Compounds 18 and 19 are specific as they can be regarded as ionic liquids (ILs) which might potentially improve solubility and the drug delivery process.^38^ Derivatives 20 and 21 feature a pendant phenylsulfonyl moiety and thus show structural similarity to benzosiloxaboroles decorated with arylsulfonate and arylsulfonamide groups which exhibit potent activity against Gram-positive cocci.^26,27^

**Scheme 2:**
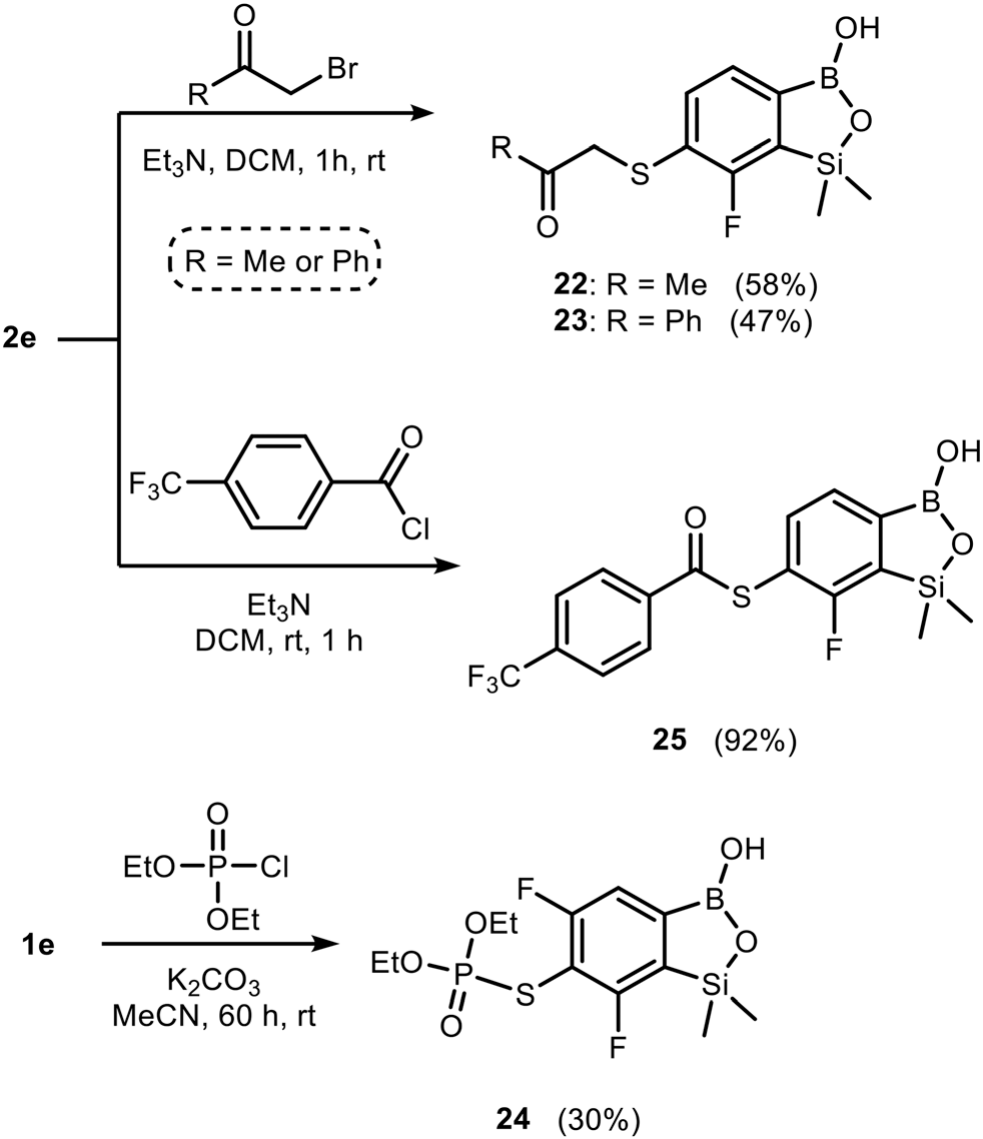
Synthesis of benzosiloxaboroles 22–25 through treatment of 1e or 2e with electrophiles featuring reactive C–Hal or P–Cl bonds.

Benzosiloxaboroles bearing side chains linked *via* the thioether group can also be obtained by treatment of 1e or 2e with electrophilic partners comprising reactive C–Hal (Hal = Cl, Br) or P–Cl bonds. Thus, respective products 22–25 were isolated using α-bromoketones, 4-(trifluoromethyl)benzoyl chloride and diethyl chlorophosphate (Scheme 2).

**Scheme 3:**
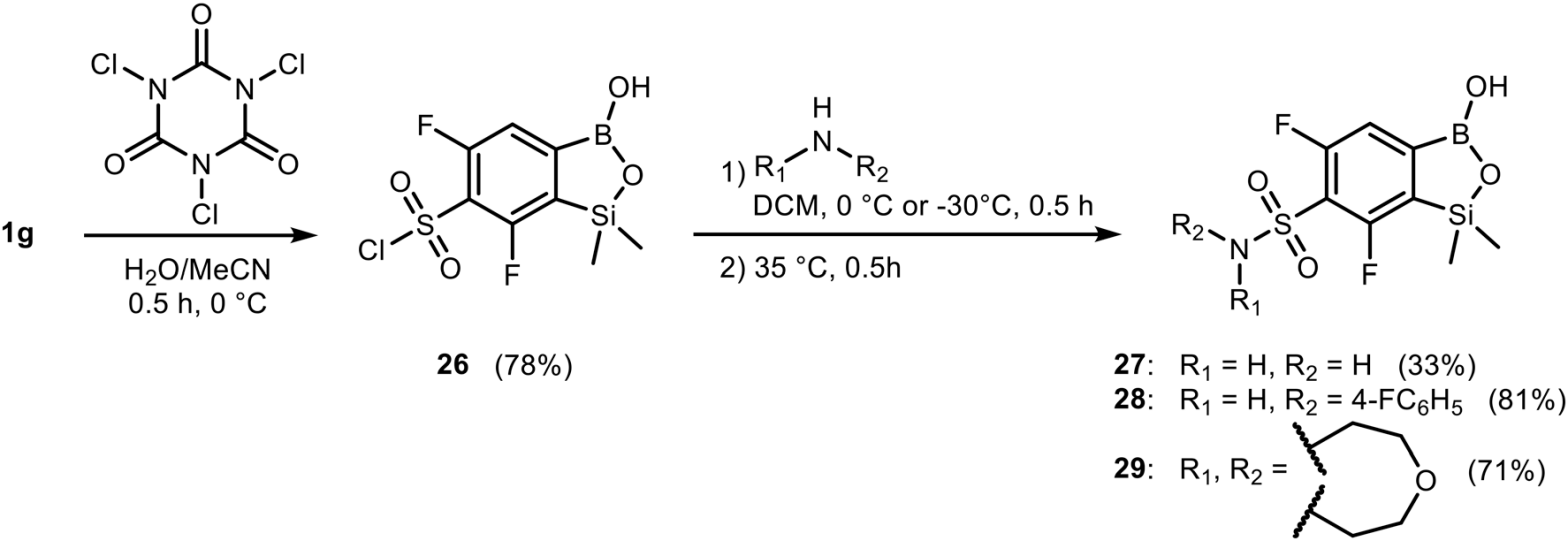
Oxidation of 1e to the corresponding sulfonyl chloride derivative 26 and subsequent conversion to sulfonamides 27, 28 and 29.

Thiols can also be easily oxidized into sulfonyl chlorides using trichloroisocyanuric acid (TCCA).^39,40^ Accordingly, benzosiloxaborole 26 was prepared by chemoselective oxidation of the SH group in 1e and subsequently converted to sulfonamides 27–29 (Scheme 3). Since sulfonamides are widely exploited as antibacterial drugs,^41–44^ we reasoned that their combination with the benzosiloxaborole scaffold could result in enhanced antimicrobial potency.

The obtained compounds are white solids that are well soluble in most organic solvents. They were characterized by multinuclear NMR (^1^H, ^13^C, ^11^B, ^19^F, ^31^P) spectroscopy and HRMS analyses. The molecular structures of selected benzosiloxaboroles 1e, 6, 11, 12, 14, 15, 20, 22, 24, 26, 27 and 29 were determined by X-ray crystallography (see Fig. S3 in the ESI†) showing that the geometric parameters of benzosiloxaborole cores are similar as in structures reported previously.^45^

Since acidity is an important parameter in medicinal chemistry, the p*K*_a_ values of the benzosiloxaborole precursors 1e and 2e, as well as the selected promising compounds 6, 20 and 22–24 were measured (Table 2) by potentiometric titration with aq. 0.1 M NaOH in H_2_O/MeOH (1 : 1 v/v). In the case of 1e and 2e possessing an acidic thiol group, both apparent p*K*_a1_ and p*K*_a2_ values were obtained. Since the acidic properties of the aromatic thiol group^46^ and siloxaborole^23^ moiety are comparable, it is likely that p*K*_a1_ values represent the formation of an equilibrium mixture of both possible monoanions, *i.e.*, the species with the deprotonated SH group and the boronate anion resulting from the coordination of OH^−^ to the boron atom. Difluoro-substituted benzosiloxaboroles 1e, 6, 20 and 24 are characterized by approximately one unit lower p*K*_a_ values than their monofluoro counterparts 2e, 22 and 23, which is consistent with previous findings.^23^ Overall, p*K*_a_ values of mono- and difluoro derivatives are very similar within each of these groups indicating that the type of pendant sulfur-based moiety plays a minor role. All studied compounds are sufficiently acidic to exist in respective anionic forms under standard physiological conditions (pH = 7.4), which should be beneficial for solubility in biological systems.

**Table 2:** Acidity constants (p*K*_a_ values) of studied compounds determined in H_2_O/MeOH (1 : 1)

Compound	1e	2e	6	20	22	23	24
p*K*_a_	4.7 (7.9)^a^	6.2 (8.7)^a^	5.2	5.7	6.4	6.3	4.9

a p*K*_a1_ and p*K*_a2_ (in parentheses) values were determined.

### Antimicrobial activity

We have recently proven that various benzosiloxaboroles display high antibacterial and antifungal activities.^23,25–27^ Thus, in this study, we tested the ability of all obtained compounds to inhibit the growth of selected standard strains of bacteria (6 Gram-positive strains and 11 Gram-negative strains) and yeasts (7 strains). All obtained results are presented in Table 3 and Tables S1 and S2 in the ESI.† Most compounds displayed moderate to weak activity against Gram-positive cocci, with MICs ranging from 12.5 to >400 μg mL^−1^ (Table 3). However, derivatives 6, 12, 20, and 22–24 were highly active against standard staphylococci, including MRSA, with MIC = 1.56–6.25 μg mL^−1^. MRSA strains are of great clinical concern. Not only are they resistant to almost all β-lactams (except ceftaroline and ceftobiprole), but they are often resistant to many other antibiotic classes (macrolides, tetracyclines, aminoglycosides, fluoroquinolones), which severely limits therapeutic options.^47,48^ Consequently, they entered the WHO list of the most dangerous high-priority pathogens, for which the search for new antibiotics is urgently needed.^49^ Thus, in this study, agents highly active against the standard MRSA strain were subsequently tested against five clinical MRSA strains (Table 4). Compound 23 bearing the benzoylmethylthio functionality displayed the highest potency (MIC = 1.56–3.12 μg mL^−1^), followed by 2-(phenylsulfonyl)ethylthio- and acetylmethylthio derivatives 20 and 22, respectively (MRSA: MIC = 3.12–6.25 μg mL^−1^). Their activity was comparable or only 3- to 6-fold lower than our reference agent linezolid, which is an antibiotic indicated in infections caused by multi-drug resistant Gram-positive cocci, including MRSA.^48^ Interestingly, their MICs for *S. aureus* were comparable with linezolid breakpoints (according to CLSI, strains are classified as sensitive when linezolid MIC is ≤ 4 μg mL^−1^ and as resistant when MIC is ≥8 μg mL^−1^).^50^ Thus, 20, 22, and 23 are promising anti-MRSA agents comparable with the previously described *N*-methyl arylsulfonamide benzosiloxaboroles (MRSA: MIC = 3.12–6.25 μg mL^−1^).^27^ It should be noted that the structural homologue of 22, namely compound 3, bearing the additional methylene spacer between the sulfur atom and the carbonyl group, is much less active.

**Table 3:** The antibacterial activity of tested agents against standard Gram-positive strains

Agent^c^	MIC/[MBC],^a^ μg mL^−1^ (diameter of inhibition zone, mm)					
	*S. aureus* ATCC 6538P	*S. aureus* ATCC 43300 MRSA	*S. epidermidis* ATCC 12228	*E. faecalis* ATCC 29212	*E. faecium* ATCC 6057	*B. subtilis* ATCC 6633^b^
1e	12.5 [25] (19)	12.5 [25] (19)	**1.56** [50] (24)	12.5 [100] (17)	12.5 [400] (24)	NT (22)
2e^d^	12.5 [25] (20)	12.5 [25] (20)	**6.25** [25] (23)	12.5 (18)	12.5 (18)	NT (23)
3	50 [100] (23)	50 [200] (19)	25 [200] (25)	25 [200] (19)	12.5 [200] (21)	NT (25)
4	12.5 [*25/400*]^f^ (27)	12.5 (24)	25 (22)	200 (12)	100 (13)	NT (23)
5	400 (15)	400 (—)	400 (—)	400 (—)	400 (—)	NT (—)
6	**6.25** [*12.5/>400*]^f^ (30)	12.5 (19)	50 (—)	400 (—)	400 (—)	NT (23)
7	50 (24)	100 (—)	100 (19)	400 (12)	200 (12)	NT (15)
8	50 (25)	50 (25)	100 (19)	400 (—)	200 (—)	NT (14)
9	25 [400] (17)	50 [400] (12)	50 [400] (11)	200 (13)	200 (15)	NT (15)
10	50 [400] (24)	50 [400] (25)	50 [400] (22)	200 (13)	100 (15)	NT (19)
11	25 [400] (21)	50 [400] (14)	25 [400] (13)	200 (12)	200 (14)	NT (17)
12	**6.25** [100] (20)	12.5 [200] (23)	**6.25** [400] (28)	25 (20)	12.5 (23)	NT (27)
13	25 [200] (20)	25 (22)	25 (24)	25 (19)	12.5 (20)	NT (24)
14^c^	50 (22)	50 (24)	50 (20)	25 (20)	50 (20)	NT (19)
15^c^	25 (23)	25 (21)	12.5 (21)	12.5 (20)	25 (22)	NT (19)
16^c^	50 [100] (20)	50 [100] (20)	50 [100] (18)	25 [200] (18)	50 (18)	NT (19)
17	25 [100] (19)	25 [100] (19)	12.5 [100] (18)	12.5 [200] (17)	25 (18)	NT (19)
18	25 [100] (19)	50 [200] (17)	50 [200] (19)	200 (—)	100 (—)	NT (18)
19	25 [100] (20)	25 [100] (18)	25 [100] (21)	100 [400] (12)	100 (12)	NT (19)
20	**3.12** [*12.5/200*]^f^ (30)	**6.25** [200] (28)	25 [400] (25)	100 (14)	100 (15)	NT (25)
21	12.5 [200] (26)	12.5 [400] (25)	25 (22)	200 (15)	200 (13)	NT (21)
22	**1.56** [200] (25)	**6.25** [200] (25)	**6.25** [12.5] (23)	25 [400] (16)	25 (12)	NT (24)
23	**1.56** [*3.12/200*]^f^ (31)	**3.12** [*25/100*]^f^ (31)	12.5 [50] (25)	50 [200] (17)	50 (17)	NT (24)
24	**6.25** [*12.5/>400*]^f^ (32)	12.5 [>400] (27)	100 (20)	>400 (11)	400 (11)	NT (21)
25^e^	12.5 [25] (11)	12.5 [25] (11)	**3.12** [25] (16)	12.5 [25] (11)	12.5 [>50] (12)	NT (15)
26	200 [400] (14)	200 (15)	200 (13)	200 (14)	100 (14)	NT (15)
27	400 (—)	>400 (—)	>400 (—)	400 (—)	400 (—)	NT (—)
28	50 (22)	100 (16)	50 [100] (19)	400 (—)	400 (—)	NT (22)
29	200 [400] (16)	400 (—)	400 (—)	> 400 (—)	400 (—)	NT (12)
**LIN** g	1 [>128] (25)	2 [>128] (25)	1 [>128] (26)	2 [>128] (15)	2 [>128] (14)	NT (30)

a Only the MBC values ≤400 μg mL^−1^ are presented.

b The growth type of *B. subtilis* in MHB medium prevented reading the MIC values of tested agents.

c The MIC and MBC values of the agent were determined up to 200 μg mL^−1^. In the table, only MBC values ≤200 μg mL^−1^ are presented. The tested agent dissolved in DMSO precipitated after implementation into MHB medium at a concentration above 200 μg mL^−1^.

d The MIC and MBC values of the agent were determined up to 100 μg mL^−1^. In the table, only MBC values ≤100 μg mL^−1^ are presented. The tested agent dissolved in DMSO precipitated after implementation into MHB medium at a concentration above 100 μg mL^−1^.

e The MIC and MBC values of the agent were determined up to 50 μg mL^−1^. In the table, only MBC values ≤50 μg mL^−1^ are presented. The tested agent dissolved in DMSO precipitated after implementation into MHB medium at a concentration above 50 μg mL^−1^.

f The Eagle effect was observed during the determination of the MBC value of the same tested agents against *S. aureus* strains.^51,52^ The Eagle effect is shown in italic face.

g LIN, linezolid, was used as a reference agent active against Gram-positive bacteria. The diameter of a commercial disc containing 0.03 mg of linezolid was 6 mm; the MIC of linezolid was determined according to the CLSI recommendations.^53^

**Table 4:** The antibacterial activity of 6, 12, 20, and 22–25 agents against *S. aureus* MRSA strains

Agent	MIC [MBC], μg mL^−1^					
	ATCC 43300 MRSA	NMI 664 K MRSA	NMI 1576 K MRSA	NMI 1712 K MRSA	NMI 1991 K MRSA	NMI 2541 K MRSA
6	12.5 [>400]	12.5 [*25/>400*]^a^	12.5 [*25/>400*]^a^	12.5 [*12.5/>400*]^a^	12.5 [*25/>400*]^a^	12.5 [*25/>400*]^a^
12	12.5 [200]	12.5 [200]	**6.25** [100]	12.5 [200]	12.5 [100]	12.5 [200]
20	**6.25** [200]	**6.25** [*12.5/400*]^a^	**6.25** [*12.5/400*]^a^	**6.25** [*12.5/400*]^a^	**6.25** [*12.5/400*]^a^	**6.25** [*25/400*]^a^
22	**6.25** [200]	**6.25** [200]	**6.25** [200]	**6.25** [400]	**3.12** [200]	**3.12** [200]
23	**3.12** [*25/100*]^a^	**3.12** [*3.12/200*]^a^	**3.12** [*6.25/100*]^a^	**3.12** [*25/200*]^a^	**1.56** [*3.12/200*]^a^	**1.56** [*6.25/200*]^a^
24	12.5 [>400]	**6.25** [*25/>400*]^a^	**6.25** [*12.5/400*]^a^	12.5 [*25/>400*]^a^	12.5 [*12.5/>400*]^a^	12.5 [*12.5/>400*]^a^
25	12.5 [25]	12.5 [25]	25 [50]	12.5 [25]	25 [50]	12.5 [25]
**LIN** b	2 [>128]	1 [>128]	1 [>128]	1 [>128]	1 [>128]	1 [>128]

a The Eagle effect was observed when determining the MBC value of the same tested agents against *S. aureus* strains.^51,52^ The Eagle effect is shown in italic face.

b LIN, linezolid, was used as a reference agent, active against *S. aureus* strains. The MIC of linezolid was determined according to the CLSI recommendations.^53^

The minimal bactericidal concentrations (MBCs) of most tested agents for staphylococci were established at 2–16 × MIC, whereas for enterococci these values were usually above the highest tested concentration (>400 μg mL^−1^) (Table 3). The lowest MBC values against *S. aureus* strains were obtained for compounds 1e, 2e and 25 (MBCs 25–50 μg mL^−1^). Interestingly, for 4, 6, 20, 23, and 24 the Eagle effect (also known as the paradoxical growth)^51,52^ was observed in the case of *S. aureus* ATCC 6538P and five MRSA clinical strains, which is in line with previous results obtained for benzosiloxaboroles decorated with arylsulfonate^26^ and *N*-methyl arylsulfonamide^27^ groups. Consequently, two MBC values were determined (Tables 3 and 4). According to CLSI recommendations, MBC is the lowest concentration that kills at least 99.9% of bacteria.^54^ In our study, the first MBCs were 2- or 4-fold higher than the MICs. However, a progressive increase in the number of surviving bacteria was observed at concentrations beyond it, followed by a subsequent decrease at 100–>400 μg mL^−1^. If the bacterial population was reduced again to the MBC threshold, a second MBC value was reported.

Furthermore, only agents 12 and 13 displayed weak activity against Gram-negative rods with MICs ranging from 25 to >400 μg mL^−1^ (ESI,† Table S1). However, considering that Gram-negative bacilli resistance is frequently associated with efflux pumps' activity,^55–57^ we also determined the MICs in the presence of their inhibitor, *i.e.*, phenylalanine-arginine-β-naphthylamide (PAβN).^57–59^ It turned out that the activity of agents 12 and 13 against *Enterobacterales* is affected by the efflux phenomenon, as their MICs are reduced at least 4-fold in the presence of PAβN. Compounds 4, 20, and 21 are also actively extruded from bacterial cells, whereas for other compounds the efflux assay confirmed the lack of any activity.

We have previously reported that some benzosiloxaboroles are potent antifungals, with MICs ranging from 0.78–12.5 μg mL^−1^.^23,26,27^ Thus, we investigated the activity of our new derivatives against 7 standard yeasts. The moderate antifungal activity was found for agents 14–17, and 25 with MICs ranging from 12.5 to >200 μg mL^−1^ for *Candida* spp. and from 6.25–200 μg mL^−1^ for *Saccharomyces cerevisiae* (ESI,† Table S2). Compounds 1e, 3, 4, 18, 19, 21–23 displayed only weak activity with MICs ranging from 100 to >400 μg mL^−1^ for *Candida* spp. and 6.25–400 μg mL^−1^ for *S. cerevisiae*.

### Cytotoxicity studies

To evaluate the cytotoxic effect of the tested compounds, an MTT-based assay was performed. Human normal lung fibroblasts MRC-5 were treated with representative compounds including the most active ones in the concentration range of 6.25–400 μg mL^−1^ for 48 h. All viability data are summarized in Table S3 (ESI†). Whenever possible, the respective IC_50_ values were calculated and summarized in Table 5. The representative plots demonstrating sigmoidal dose–response curves for the tested compounds are shown in the ESI† (Fig. S1). Overall, the results indicate rather weak cytotoxicity as for most compounds (except for 16 and 28), IC_50_ values are above 100 μg mL^−1^. Moreover, based on available IC_50_ and MIC (for standard and clinical *S. aureus* MRSA strains) values, the selectivity index (SI) was calculated for several compounds as the IC_50_/MIC ratio (Table 5).^60^ Within the group of most active compounds (6, 12, 20, 22–24), SI values were generally higher than 10, indicating their potential as antibacterial agents with respect to *S. aureus* MRSA.

**Table 5:** Summary of IC_50_ and SI values. SI values were calculated as SI = IC_50_/MIC with respect to *S. aureus* ATCC 43300 MRSA standard strain and *S. aureus* MRSA clinical strains (NMI). For the latter, SI-range values are given (calculated on the basis of the lowest and highest MIC values)

Agent	IC_50_ [μg mL^−1^]	SI for *S. aureus* ATCC 43300 MRSA	SI range for *S. aureus* MRSA clinical strains (NMI)
6	140.0	**11.2**	**11.2**
12	167.4	**13.4**	**13.4–26.8**
14	133.3	2.7	n/a
15	145.0	5.8	n/a
16	76.2	1.5	n/a
17	139.5	5.6	n/a
20	122.0	**19.5**	**19.5**
22	>200	**>32.0**	**>32.0**
23	126.1	**40.4**	**40.4–80.8**
24	>400	**>32.0**	**>32.0**
25	111.7	8.9	4.5–8.9
27	>400	>	n/a
28	74.9	0.7	n/a
29	>400	>	n/a
**LIN**	>400	**>200**	**>400**
Cisplatin	1.9	n/a	n/a

### Studies on the mechanism of antibacterial activity

We have undertaken research devoted to determining the most probable mechanism of antibacterial action. Numerous reports indicate that related benzoxaboroles exhibit antimicrobial activity through the oxaborole tRNA trapping (OBORT) mechanism^10,61–65^ involving inhibition of leucyl-tRNA synthetase (LeuRS). Considering structural similarity to benzoxaboroles we assumed that benzosiloxaboroles are active through this mechanism. Our working hypothesis was supported by the fact that benzosiloxaboroles are rather bacteriostatic than bactericidal,^26,27^ which is consistent with the specificity of the OBORT mechanism. The enzyme inhibition assay was performed for compound 20 as it features high activity against *S. aureus* MRSA strains and the respective favorable SI = 19.5 (Table 5). Moreover, due to the presence of the SO_2_ group in the pendant substituent, it shows structural analogy to previously reported benzosiloxaboroles bearing sulfonate and sulfonamide groups which also exhibit high activity against *S. aureus*.^26,27^ We obtained *S. aureus* MRSA LeuRS in the *E. coli* expression system. From 400 mL culture, approximately 20 mg of 6× His tagged LeuRS (with a molecular mass of 92.3 kDa) was purified to more than 90% homogeneity, which was confirmed by sodium dodecylsulfate-polyacrylamide gel electrophoresis (SDS-PAGE; Fig. S2, ESI†). Next, we used an *in vitro* aminoacylation assay to investigate the ability of 20 to inhibit *S. aureus* MRSA LeuRS activity consistent with affecting the transfer of the ^14^C-radiolabeled leucine to a total tRNA. We found that 20 did not show the expected activity (residual activity of 92.9% at 25 μM), compared to reference arylsulfonamide-substituted benzoxaborole PT662 known as a potent *S. aureus* MRSA LeuRS inhibitor^10^ (residual activity 30.8% at 25 μM).

**Fig. 3:**
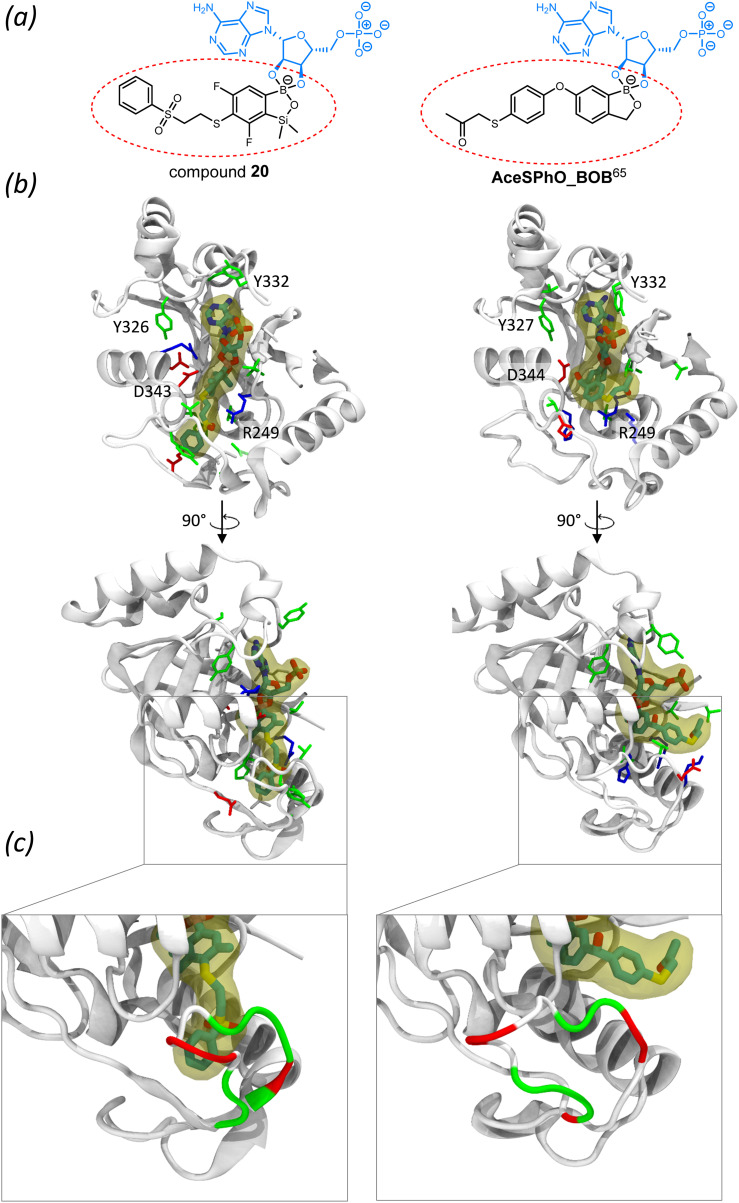
Structural determinants of LeuRS inhibition by oxaborole derivatives; (a) 2D representations of ligands. Since known LeuRS oxaborole inhibitors work by forming complexes with tRNA nucleotides, both compounds have been represented as AMP adducts. The studied compound 20 is depicted on the left and a known potent inhibitor (**AceSPhO_BOB** (ref. 65)) is depicted on the right; (b) comparison of the binding modes of AMP adducts of both compounds with LeuRS. On the left, the binding mode of compound 20 obtained through docking (using MOE (ref. 66)) into a homology model of the *S. aureus* MRSA LeuRS, and on the right, the binding mode of **AceSPhO_BOB** (ref. 65) as seen in the crystal structure [PDB code: 7BZJ]. Residues in proximity of the ligands are colored according to the residue property (white – nonpolar, green – polar, blue – negatively charged, red – positively charged). Labels were added to selected residues adjacent to the ligand for reference; (c) contacts between the pendant group of both compounds and its interaction with a polar LeuRS loop. The loop is colored according to the residue property (the same color code as above).

Subsequent bioinformatic analysis provided possible explanations for this disclosure. Compound 20 possesses a high degree of structural similarity to potent antibacterial compound 1-((4-((1-hydroxy-1,3-dihydrobenzo[*c*][1,2]oxaborol-6-yl)oxy)phenyl)thio)propan-2-one,^65^**AceSPhO_BOB**, which has been crystallized in complex with *Streptococcus pneumoniae* leucyl-tRNA synthetase (Fig. 3a, right). By using the crystallized complex as a reference, we generated a putative *S. aureus* MRSA LeuRS/(20 bound to AMP) complex through docking. A comparison of binding modes of both compounds (Fig. 3b) revealed that although the oxaborole moiety of 20 occupies a similar space within the protein as for **AceSPhO_BOB**,^65^ the pendant groups of both compounds are oriented very differently. In the case of 20, the phenylsulfonyl group forms tight interactions with LeuRS, penetrating the cavity between structured regions of the protein and a loop (Fig. 3c). Clearly, it is not surprising as in 20 the large substituent is attached at the *para* position with respect to the boron atom, while for **AceSPhO_BOB** (ref. 65) an analogous moiety is located at the *meta* position (Fig. 3a). It should be stressed that the loop comprises mostly polar and negatively charged residues (Fig. 3c). Such an environment is highly unfavorable for the primarily hydrophobic pendant group of 20 which would likely aggravate binding of this compound.

**Fig. 4:**
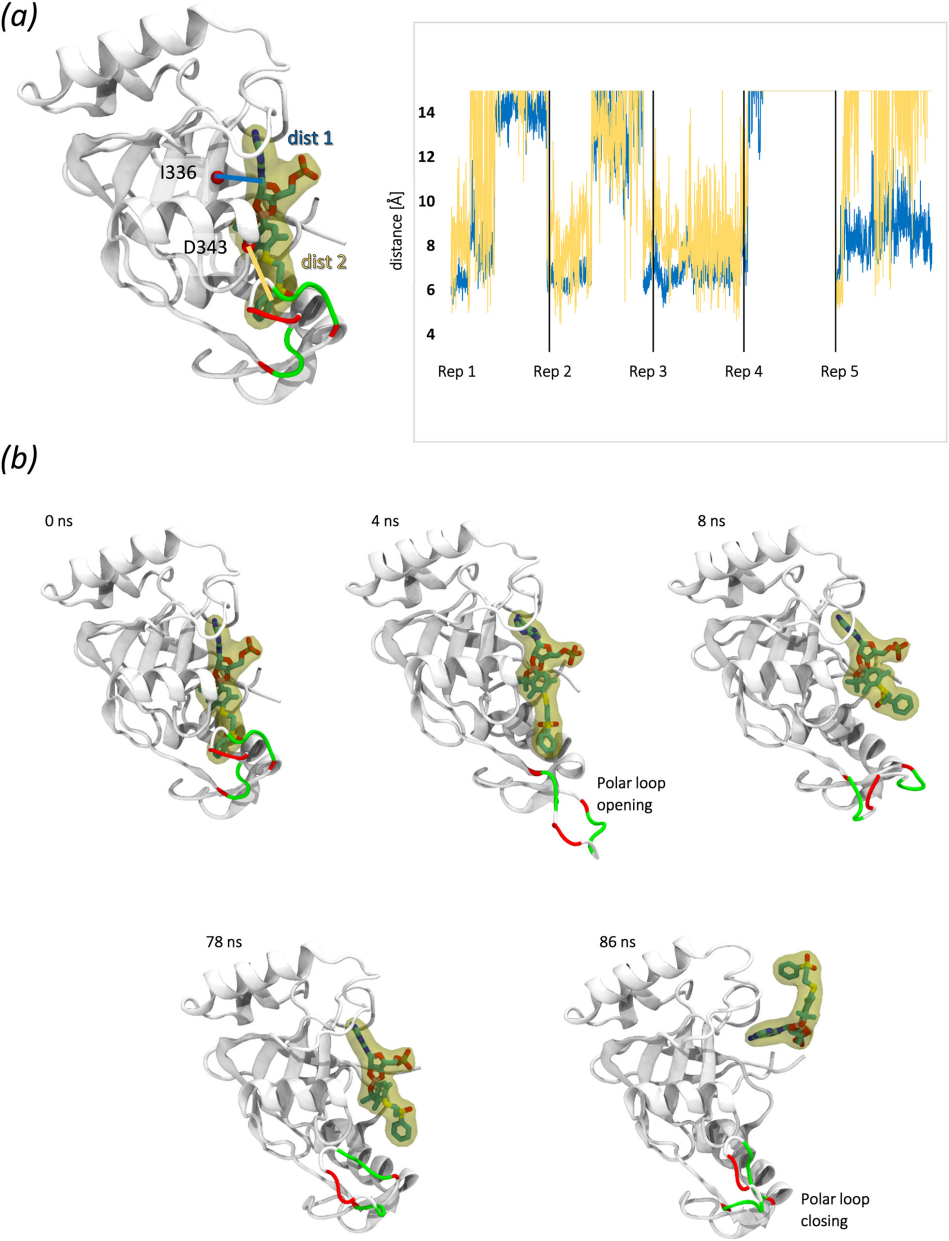
MD evaluation of interactions between the ligand – AMP adduct of benzosiloxaborole 20 and *S. aureus* MRSA LeuRS; (a) initial structure of the (AMP adduct of 20)/LeuRS complex subjected to 5 × 1 μs of unbiased MD simulations. The distances between the protein and the benzosiloxaborole core (blue) and the pendant group (yellow) are depicted as lines, and their evolution across the simulations is plotted; (b) evolution of the position of the AMP adduct of 20 across one unbiased simulation run. The loop initially interacting with the ligand is colored based on the residue property (white – nonpolar, green – polar, red – negatively charged).

To further evaluate the stability of the generated complex, we have carried out extensive (5 × 1 μs) unbiased MD simulation of the generated ligand, *i.e.*, (20 bound to AMP)/LeuRS complex. Importantly, as parameters for silicon atoms are not available within the utilized simulation forcefield, this atom in the ligand structure was replaced with a carbon atom (as it shares multiple chemical properties with silicon atoms). Unbiased simulations were performed in order to observe changes in the structure of the complex, which could help rationalize the lack of affinity of studied compounds towards LeuRS. The relative positions of the protein and atoms in the benzosiloxaborole core and the pendant moiety were plotted to monitor the stability of the ligands (Fig. 4a). Intriguingly, we found that in each of the replicates both fragments assume distances characteristic to unbound conformations. A more detailed analysis of one of the unbinding events (Fig. 4b) observed in the unbiased simulations reveals the following sequence of events: in the initial frames the polar loop quickly unbinds (likely due to unfavorable interactions with the ligand). This is followed by destabilization of the pendant group, which later leads to full unbinding and displacement of 20. The observed unbinding event, preceded by the opening of the polar loop, would suggest that 20 does not interact with LeuRS as the largely polar and electronegative environment of the loop cannot accommodate the hydrophobic and electronegative pendant moiety of the studied ligand.

The lack of activity of 20 towards LeuRS indicates a different mechanism of antibacterial activity. Bioinformatic analysis suggests that this inability might be linked with the placement of its bulky sulfur-linked moiety in the *para* position with respect to the boron atom. Importantly, this would also suggest that none of the compounds within this series, which have a large hydrophobic/negatively charged pendant group in the *para* position, would inhibit LeuRS. Although LeuRS remains the most frequently identified molecular target for antimicrobial organoboron compounds, few examples of differently acting boron-based antimicrobial agents are also known. For instance, in the case of bis(indolyl)methylboronic acid derivatives, it was found that their antibacterial activity might be linked with a strong binding affinity to the peptidoglycan layer of the Gram-positive bacteria cell wall.^67^ On the other hand, some benzoxaboroles were identified as potent inhibitors of other bacterial enzymes, such as NADH dehydrogenase,^9^ enoyl acyl carrier protein FabI^68^ or carbonic anhydrases.^69^ These findings are in line with our disclosure and prove that benzosiloxaboroles, like other boron-based antimicrobials, could utilize another antibacterial mechanism than OBORT as well.

## Conclusions

Thiol-functionalized benzosiloxaboroles proved to be very useful precursors for the preparation of a wide variety of novel derivatives. Most of them were obtained by the thiol-Michael addition reaction, which turned out to be a convenient tool for the preparation of many structurally diverse benzosiloxaboroles. Some of them were also synthesized through the nucleophilic substitution reaction of thiolates with appropriate electrophilic partners as well as through chemoselective SH-oxidation and subsequent transformations. We intended to decorate new derivatives with extended substituents containing multiple polar groups as we assumed that such functionalizations may result in a stronger and specific binding to biological targets by means of polar interactions, *e.g.*, hydrogen bonds. Regardless of the substitution pattern at the sulfur atom, the studied benzosiloxaboroles display a comparable acidity (p*K*_a_ of 4.7–6.4 in H_2_O/MeOH, 1 : 1). Compounds 6, 12, 20 and 22–24 show strong bacteriostatic activity, especially against *S. aureus*, including multidrug-resistant clinical strains (*S. aureus* MRSA). Overall, moderate activity against Gram-positive bacteria is common. Furthermore, SAR analysis indicates that the antibacterial properties are enhanced for difluorinated derivatives. Following the initial premises, the antibacterial activity of the studied benzosiloxaboroles seemed to be based on the tRNA-targeting OBORT mechanism. However, experimental investigation revealed that the studied agent 20 does not effectively inhibit LeuRS as IC_50_ > 200 μM. Hence, it can be concluded that benzosiloxaboroles have a different molecular target in a bacterial cell than benzoxaboroles. The lack of affinity to LeuRS was also confirmed by bioinformatic structural analysis. To summarize, the potential of SH-substituted benzosiloxaboroles for the synthesis of a diverse library of structurally extended derivatives was amply demonstrated and represents a significant progress in the field. Selected results of antimicrobial activity screening are promising. Hopefully, the presented findings will be followed by future research, eventually leading to development of new boron-based antibiotics.

## Experimental section

### General comments

Solvents used for reactions were dried by heating to reflux with sodium/benzophenone and distilled under argon. Starting materials and other reagents including halogenated benzenes, alkyllithiums, diisopropylamine, trimethyl borate, *tert*-butyl(chloro)dimethylsilane, or chlorodimethylsilane were used as received without further purification. Reactions in which organometallic compounds were used were carried out under an argon atmosphere. Detailed procedures for the synthesis of Michael acceptors used in reactions leading to the formation of compounds 5, 9–11, 14–19 as well as α-bromoketones used in syntheses of 22 and 23 are given in ESI,† section S1. ^1^H, ^13^C, ^19^F and ^31^P NMR spectra were recorded on an Agilent NMR 400 MHz DDR2 spectrometer. ^11^B NMR spectra were recorded on a Bruker AVANCE III 300 MHz spectrometer. In the ^13^C NMR spectra the resonances of boron-bound carbon atoms were not observed in most cases as a result of their broadening by a quadrupolar boron nucleus. ^1^H and ^13^C NMR chemical shifts are given relative to TMS using residual solvent resonances. ^11^B and ^19^F NMR chemical shifts are given relative to BF_3_·Et_2_O and CFCl_3_, respectively. ^31^P NMR chemical shifts are given relative to 85% phosphoric acid solution in D_2_O. CSD deposition numbers 2190658 (for 2e), 2190659 (for 1b**_D**), 2190660 (for 1b**_CH2**), 2190661 (for 6), 2190662 (for 11), 2190663 (for 12), 2190664 (for 14), 2190665 (for 15), 2190666 (for 20), 2296881 (for 22), 2190667 (for 24), 2258836 (for 26), 2190668 (for 29), 2190669 (for 27), contain the ESI† crystallographic data for this paper.

### Synthesis

#### 4-Bromo-2,6-difluorobenzenethiol (1b)

A solution of 1-bromo-3,5-difluorobenzene (58.0 g, 0.301 mol) in THF (90 ml) was added dropwise to a stirred solution of LDA (0.316 mol) freshly prepared from *n*-BuLi (9.6 M in hexane, 32.9 mL, 0.316 mol) and diisopropylamine (46.3 ml, 33.45 g, 0.331 mol) in THF (300 ml) at −78 °C. The resultant brown solution was stirred for 30 minutes, followed by an addition of sulfur (9.61 g, 0.301 mol). The mixture was stirred overnight at −78 °C. Then it was acidified at −78 °C using 1.5 M aq. H_2_SO_4_ until the pH = 1. The resulting orange slurry was then slowly warmed up to room temperature. During warming, gradual dissolution of the pale precipitate was observed. The organic phase was separated and the aqueous phase extracted with Et_2_O (3 × 30 mL). The combined organic solutions were dried over anhydrous MgSO_4_ and evaporated to give a yellow colored residue which was distilled under reduced pressure (b.p. 40–45 °C, *ca.* 10^−3^ mbar) to give 1b as a pale yellow oil. Yield 34.21 g (51%). ^1^H NMR (400 MHz, CDCl_3_) *δ* 7.13–7.02 (m, 2H), 3.56 (s, 1H) ppm. ^13^C NMR (101 MHz, CDCl_3_) *δ* 159.1 (d, *J* = 248.9 Hz), 159.0 (d, *J* = 249.1 Hz), 117.7 (t, *J* = 11.8 Hz), 115.5–115.1 (m), 108.9–107.8 (m) ppm. ^19^F NMR (376 MHz, CDCl_3_) *δ* −107.22 to −107.32 (m) ppm.

#### ((4-Bromo-2,6-difluorophenyl)thio)(*tert*-butyl)dimethylsilane (1c)

To a solution of 1b (51.79 g, 0.230 mol) and *tert*-butyldimethylsilyl chloride (38.15 g, 0.253 mol) in Et_2_O (300 mL), triethylamine (27.94 g, 38.5 mL, 0.276 mol) was slowly added dropwise under argon. Immediate precipitation of a white solid was observed during the dropwise addition. The resulting thick oily suspension was stirred for 2 hours. Then, it was triturated with heptane (250 mL) and stirred overnight at ambient temperature. The white precipitate was filtered off under an argon atmosphere and washed with heptane (3 × 50 mL). Then the filtrate was concentrated under reduced pressure to give residual yellow oil, which was subsequently distilled under high vacuum (b.p. 110–115 °C, *ca.* 10^−3^ mbar) to give 1c as a pale yellow oil. Yield 67.85 g (87%). ^1^H NMR (400 MHz, CDCl_3_) *δ* 7.14–7.05 (m, 2H), 1.00 (s, 9H), 0.19 (s, 6H) ppm. ^13^C NMR (101 MHz, CDCl_3_) 163.83 (d, *J* = 249.5 Hz), 163.77 (d, *J* = 249.5 Hz), 120.63 (t, *J* = 12.2 Hz), 115.42 (dd, *J* = 29.4, 2.1 Hz), 115.42 (d, *J* = 12.9 Hz), 108.07 (t, *J* = 23.8 Hz), 26.09, 19.06, −3.38 ppm. ^19^F NMR (376 MHz, CDCl_3_) *δ* −100.76 (d, *J* = 5.9 Hz) ppm.

#### ((4-Bromo-3-(dimethylsilyl)-2,6-difluorophenyl)thio)(*tert*-butyl)dimethylsilane (1d)

A solution of 1c (33.90 g, 0.100 mol) in Et_2_O (25 ml) was added dropwise to a stirred solution of LDA freshly prepared from *n*-BuLi (9.6 M in hexane, 12.0 ml, 0.115 mol) and diisopropylamine (14.17 g, 19.6 ml, 0.140 mol) in THF (100 ml) at −78 °C. After *ca.* 30 min stirring at −78 °C chlorodimethylsilane (14.19 g, 16.35 ml, 0.150 mol) was added slowly and the mixture was stirred for 30 min at −78 °C. During the addition, precipitation of a white solid was observed. The resulting slurry was then allowed to warm to room temperature. Upon warming up, it was concentrated under reduced pressure. The residual thick oily slurry was triturated with heptane (100 mL) under argon and the obtained suspension was filtered under argon in order to remove the solid byproduct LiCl. The precipitate was washed with heptane (3 × 20 ml). The filtrate was then concentrated and finally subjected to fractional distillation under high vacuum (b.p. 110–114 °C, *ca.* 10^−3^ mbar) to give 1d as a pale yellow oil. Yield 18.60 g (47%). ^1^H NMR (400 MHz, CDCl_3_) *δ* 7.16 (dd, *J* = 7.6, 1.7 Hz, 1H), 4.75 (dp, *J* = 4.9, 3.9 Hz, 1H), 1.00 (s, 9H), 0.42 (dd, *J* = 3.9, 2.0 Hz, 6H), 0.18 (t, *J* = 0.9 Hz, 6H) ppm. ^13^C NMR (101 MHz, CDCl_3_) *δ* 167.8 (dd, *J* = 243.8, 4.9 Hz), 164.5 (dd, *J* = 250.7, 6.1 Hz), 128.3 (dd, *J* = 15.3, 11.9 Hz), 121.7 (dd, *J* = 35.9, 3.8 Hz), 117.0 (dd, *J* = 26.5, 3.9 Hz), 107.4 (dd, *J* = 29.1, 22.4 Hz), 26.1, 19.1, −3.3 (d, *J* = 4.3 Hz), −3.32 to −3.49 (m) ppm. ^19^F NMR (376 MHz, CDCl_3_) *δ* −85.13 (t, *J* = 5.8 Hz), −99.94 ppm.

#### 5,7-Difluoro-6-mercapto-1,1-dimethylbenzo[*c*][1,2,5]oxasilaborol-3(1*H*)-ol (1e)

A solution of 1d (18.60 g, 0.047 mol) in Et_2_O (volume ratio 1 : 1) was added dropwise to a solution of *t*-BuLi (1.8 M in pentane, 52.0 mL, 0.094 mol) in Et_2_O (150 mL) at −95 °C. After 30 min of stirring at −95 °C, B(OMe)_3_ (10.4 mL, 9.73 g, 0.094 mol) was added slowly to the colorless mixture at −95 °C. The resulting white suspension was warmed slowly to *ca.* 0 °C, quenched with 1 M aq. NaOH (100 mL) and stirred at room temperature until evolution of H_2_ ceased. The two-phase mixture was concentrated under reduced pressure in order to remove solvents and other volatile organic components. The residual aqueous alkaline solution was transferred to a separatory funnel and washed with hexane (60 mL). Then it was placed in a beaker, cooled in an ice bath and carefully neutralized by an addition of *ca.* 50 mL 2 M aq. H_2_SO_4_ (to reach the pH = 1). The precipitated abundant white solid was filtered and washed several times with distilled water. Then it was recrystallized with heptane (70 mL) and filtered. Finally, the product was dried *in vacuo* to give 1e as a white powder. Yield 8.80 g (76%). ^1^H NMR (400 MHz, CDCl_3_) *δ* 7.32 (d, *J* = 7.9 Hz, 1H), 5.82 (s, 1H), 3.71 (s, 1H), 0.49 (s, 6H) ppm. ^11^B NMR (96 MHz, CDCl_3_) *δ* 29.4 ppm. ^13^C NMR (101 MHz, CDCl_3_) *δ* 161.1 (dd, *J* = 249.5, 3.9 Hz), 160.3 (dd, *J* = 244.5, 5.3 Hz), 159.1 (d, *J* = 5.3 Hz), 138.6 (broad), 129.6 (dd, *J* = 35.5, 2.5 Hz), 114.1 (dd, *J* = 19.8, 3.2 Hz), 112.1 (dd, *J* = 25.0, 22.0 Hz), −0.8 (s) ppm. ^19^F NMR (376 MHz, CDCl_3_) *δ* −100.05 (d, *J* = 8.1 Hz), −106.07 (t, *J* = 7.9 Hz) ppm. HRMS (ESI, negative ion mode) calcd. for C_8_H_8_BF_2_O_2_SSi^−^ [M − H]^−^: 245.0070; found: 245.0077.

#### 4-Bromo-2-fluorobenzenethiol (2b)

The synthesis was accomplished according to a protocol previously reported.^70^ To a solution of 4-bromo-2-fluorosulfonyl chloride (15.00 g, 0.055 mmol) in toluene (150 mL), under argon, Ph_3_P (47.00 g, 0.179 mmol) was added in portions over 20 min. The temperature increased to *ca.* 60 °C. The mixture was stirred overnight. Then H_2_O (50 mL) was added and the stirring was continued for 1 hour. The resulting slurry was filtered and the solid Ph_3_PO was washed with cold toluene (2 × 25 mL). The aqueous layer was discarded and the toluene layer was extracted with 10% NaOH (2 × 50 mL). The alkaline aqueous extract was washed with toluene (50 mL), acidified with 1.5 M aq. H_2_SO_4_ and extracted with DCM (2 × 50 mL). The organic layer was separated and dried over Na_2_SO_4_ and the solvent was evaporated under reduced pressure. The residue was then distilled under high vacuum (b.p. 40–45 °C, *ca.* 10^−3^ mbar) to give pure 2b. Yield 9.57 g (84%). ^1^H NMR (400 MHz, CDCl_3_) *δ* 7.25–7.21 (m, 1H), 7.20–7.16 (m, 1H), 7.15 (dd, *J* = 6.5, 0.6 Hz, 1H), 3.58 (d, *J* = 1.3 Hz, 1H) ppm. ^13^C NMR (101 MHz, CDCl_3_) *δ* 158.7 (d, *J* = 247.8 Hz), 131.3 (d, *J* = 2.6 Hz), 127.8 (d, *J* = 4.0 Hz), 119.20 (d, *J* = 25.5 Hz), 119.05 (d, *J* = 8.6 Hz), 118.2 (d, *J* = 20.2 Hz) ppm. ^19^F NMR (376 MHz, CDCl_3_) *δ* −107.32 to −107.56 (m) ppm.

#### ((4-Bromo-2-fluorophenyl)thio)(*tert*-butyl)dimethylsilane (2c)

The synthesis was performed as described for 1c starting with 2b (9.00 g, 0.043 mol). The product 2c was obtained as a pale yellow oil (b.p. 90–95 °C, *ca.* 10^−3^ mbar). Yield 12.58 g (90%). ^1^H NMR (400 MHz, CDCl_3_) *δ* 7.31 (t, *J* = 8.1 Hz, 0H), 7.24 (dd, J = 8.1, 2.1 Hz, 0H), 7.17 (ddd, *J* = 8.3, 2.1, 0.8 Hz, 0H), 0.98 (s, 1H), 0.18 (d, *J* = 0.8 Hz, 1H) ppm. ^13^C NMR (101 MHz, CDCl_3_) *δ* 163.1 (d, *J* = 249.1 Hz), 138.9 (d, *J* = 1.4 Hz), 127.5 (d, *J* = 3.9 Hz), 121.3 (d, *J* = 8.8 Hz), 119.4 (d, *J* = 27.2 Hz), 118.3 (d, *J* = 19.8 Hz), 26.2, 19.0, −3.4 (d, *J* = 1.0 Hz) ppm. ^19^F NMR (376 MHz, CDCl_3_) *δ* −101.73 (t, *J* = 7.9 Hz) ppm.

#### ((4-Bromo-3-(dimethylsilyl)-2-fluorophenyl)thio)(*tert*-butyl)dimethylsilane (2d)

The synthesis was performed as described for 1d starting with 2c (12.58 g, 0.045 mol). The product 2d was obtained as a pale yellow oil (105–115 °C, *ca.* 10^−3^ mbar). Yield 13.79 g (93%). ^1^H NMR (400 MHz, CDCl_3_) *δ* 7.29–7.22 (m, 1H), 4.86–4.70 (m, 1H), 0.98 (s, 9H), 0.43 (dd, *J* = 3.9, 2.0 Hz, 6H), 0.17 (d, *J* = 0.8 Hz, 6H) ppm. ^13^C NMR (101 MHz, CDCl_3_) *δ* 167.1 (d, *J* = 243.4 Hz), 139.9 (d, *J* = 1.8 Hz), 129.0 (d, *J* = 3.8 Hz), 129.0, 126.3 (d, *J* = 35.5 Hz), 117.8 (d, *J* = 25.1 Hz), 26.3, 19.0, −3.2 (d, *J* = 4.3 Hz), −3.4 (d, *J* = 1.1 Hz) ppm. ^19^F NMR (376 MHz, CDCl_3_) *δ* −86.09 ppm.

#### 7-Fluoro-6-mercapto-1,1-dimethylbenzo[*c*][1,2,5]oxasilaborol-3(1*H*)-ol (2e)

The synthesis was performed as described for 1e starting with 2d (21.20 g, 0.056 mol). The product 2e was obtained as a white powder. Yield 10.02 g (90%). ^1^H NMR (400 MHz, CDCl_3_) *δ* 7.48 (dd, *J* = 7.5, 1.6 Hz, 1H), 7.38 (t, *J* = 7.3 Hz, 1H), 5.43 (s, 1H), 3.72 (d, *J* = 1.8 Hz, 1H), 0.51 (s, 6H) ppm. ^11^B NMR (96 MHz, CDCl_3_) *δ* 29.7 ppm. ^13^C NMR (101 MHz, CDCl_3_) *δ* 160.0 (d, *J* = 242.3 Hz), 134.8 (d, *J* = 32.4 Hz), 132.7 (d, *J* = 1.1 Hz), 128.0 (d, *J* = 3.4 Hz), 122.4 (d, *J* = 22.8 Hz), −0.8 ppm. ^19^F NMR (376 MHz, CDCl_3_) *δ* −102.89 (d, *J* = 6.8 Hz) ppm. HRMS (ESI, negative ion mode) calcd. for C_8_H_9_BFO_2_SSi^−^ [M − H]^−^: 227.0164; found: 227.0170.

#### 4-((5,7-Difluoro-3-hydroxy-1,1-dimethyl-1,3-dihydrobenzo[*c*][1,2,5]oxasilaborol-6-yl)thio)butan-2-one (3)

To the mixture of compound 1e (246 mg, 1.0 mmol) and but-2-en-3-one (140 mg, 2.0 mmol) was added water (0.10 mL). It was stirred for 5 minutes at room temperature and then the solvent was removed under reduced pressure to give pure 3 as a white solid. Yield 285 mg (90%). ^1^H NMR (400 MHz, CDCl_3_) *δ* 7.33 (dd, *J* = 7.7, 0.7 Hz, 1H), 5.48 (s, 1H), 3.13 (t, *J* = 7.2 Hz, 3H), 2.74 (t, *J* = 7.2 Hz, 3H), 2.32–1.98 (m, 4H), 0.48 (d, *J* = 0.7 Hz, 7H) ppm. ^11^B NMR (96 MHz, CDCl_3_) *δ* 29.3 ppm. ^13^C NMR (101 MHz, CDCl_3_) *δ* 206.7, 165.0 (dd, *J* = 251.8, 2.8 Hz), 164.4 (dd, *J* = 247.1, 4.1 Hz), 143.4, 130.2 (dd, *J* = 33.9, 2.8 Hz), 114.6 (dd, *J* = 21.4, 3.5 Hz), 113.4 (dd, *J* = 24.9, 22.3 Hz), 43.7, 30.1, 28.2 (t, *J* = 2.9 Hz), −0.7 ppm. ^19^F NMR (376 MHz, CDCl_3_) *δ* −95.76 (d, *J* = 7.3 Hz), −101.83 (t, *J* = 7.5 Hz) ppm. HRMS (ESI, negative ion mode) calcd. for C_12_H_14_BF_2_O_3_SSi^−^ [M − H]^−^: 315.0489; found: 315.0499.

#### Methyl 3-((7-fluoro-3-hydroxy-1,1-dimethyl-1,3-dihydrobenzo[*c*][1,2,5]oxasilaborol-6-yl)thio)propanoate (4)

To the mixture of compound 2e (235 mg, 1.0 mmol) and methyl acrylate (250 mg, 2.9 mmol) was added water (5 mL). It was stirred for 5 minutes at room temperature and then the solution of sodium bicarbonate (100 mg, 1.2 mmol) in water (1 mL) was added. The resulting mixture was concentrated under reduced pressure. The residue was diluted with diethyl ether (5 mL) and acidified with 1.5 M aq. H_2_SO_4_. Then the organic layer was separated and dried with anhydrous MgSO_4_. The solvent was evaporated to give crude yellow oil. It was dried under high vacuum (*ca.* 10^−3^ mbar) for 1 hour. Then it was dissolved in heptane and heated. The hot solution was transferred to a beaker. After cooling slowly, precipitation was observed. The product was obtained as a white solid. Yield 300 mg (93%). ^1^H NMR (400 MHz, CDCl_3_) *δ* 7.52 (dd, *J* = 7.4, 1.6 Hz, 1H), 7.44 (dd, *J* = 7.5, 7.0 Hz, 1H), 5.26 (s, 1H), 3.69 (s, 3H), 3.22 (dd, *J* = 7.7, 7.1 Hz, 2H), 2.67 (t, *J* = 7.4 Hz, 2H), 0.49 (s, 6H) ppm. ^11^B NMR (96 MHz, CDCl_3_) *δ* 29.5 ppm. ^13^C NMR (101 MHz, CDCl_3_) *δ* 172.1, 162.4 (d, *J* = 244.2 Hz), 135.1 (d, *J* = 32.3 Hz), 133.3, 127.9 (d, *J* = 3.4 Hz), 125.5 (d, *J* = 20.5 Hz), 52.0, 34.1, 27.7 (d, *J* = 2.6 Hz), −0.7 ppm. ^19^F NMR (376 MHz, CDCl_3_) *δ* −101.24 (dd, *J* = 6.8, 1.9 Hz) ppm. HRMS (ESI, negative ion mode) calcd. for C_12_H_15_BFO_4_SSi^−^ [M − H]^−^: 313.0532; found: 313.0538.

#### 2,5-Dioxopyrrolidin-1-yl 3-((5,7-difluoro-3-hydroxy-1,1-dimethyl-1,3-dihydrobenzo-[*c*][1,2,5]oxasilaborol-6-yl)thio)propanoate (5)

To a solution of 1e (246 mg, 1.0 mmol) and succinimidyl acrylate (169 mg, 1.0 mmol) in DCM (5 mL) was added triethylamine (0.21 mL, 1.5 mmol). The mixture was stirred for 1 hour at room temperature. Then it was concentrated under reduced pressure. The residue was dissolved in acetone (5 mL), acidified with 1 M aq. HCl and stirred for 10 minutes. Then acetone was evaporated and the acidic aqueous layer was separated from the crude oily product. Upon addition of hexane (5 mL) and vigorous stirring, precipitation was observed. The suspension was filtered and the precipitate was washed with water and hexane to give pure 5 as a white solid. Yield 328 mg (79%). ^1^H NMR (400 MHz, CDCl_3_) *δ* 7.35 (d, *J* = 7.6 Hz, 1H), 5.30 (d, *J* = 0.7 Hz, 1H), 3.22 (t, *J* = 7.7 Hz, 2H), 2.92 (dd, *J* = 8.5, 7.0 Hz, 2H), 2.83 (s, 4H), 0.49 (s, 6H) ppm. ^11^B NMR (96 MHz, CDCl_3_) *δ* 29.0 ppm. ^13^C NMR (101 MHz, CDCl_3_) *δ* 169.0, 166.8, 165.0 (dd, *J* = 252.6, 2.3 Hz), 164.4 (dd, *J* = 247.8, 3.9 Hz), 144.1, 130.4 (d, *J* = 36.4 Hz), 114.8 (dd, *J* = 21.3, 4.1 Hz), 112.2 (dd, *J* = 24.8, 22.0 Hz), 32.1, 28.6, 25.6 (t, *J* = 1.5 Hz), −0.7 ppm. ^19^F NMR (376 MHz, CDCl_3_) *δ* −95.50, −101.55 ppm. HRMS (ESI, negative ion mode) calcd. for C_15_H_15_BF_2_NO_6_SSi^−^ [M − H]^−^: 414.0445; found: 414.0460.

#### 3-((5,7-Difluoro-3-hydroxy-1,1-dimethyl-1,3-dihydrobenzo[*c*][1,2,5]oxasilaborol-6-yl)thio)propanenitrile (6)

A solution of potassium carbonate (138 mg, 1.0 mmol) in water (1.5 mL) was added dropwise to a solution of 1e (246 mg, 1.0 mmol) and acrylonitrile (53 mg, 1.0 mmol) in methanol (3 mL). The mixture was stirred for 24 hours at room temperature. Then it was concentrated under reduced pressure to remove the organic solvent. The residue was diluted with water (1 mL) and acidified with 1.5 M aq. H_2_SO_4_. Upon addition of the acid, formation of a white solid was observed. The acidic aqueous phase was discarded and the solid residue was washed with water and hexane and vigorously stirred to give the suspension. Then it was filtered and the precipitate was dried *in vacuo* (*ca.* 10^−3^ mbar). The pure product 6 was obtained as a white powder. Yield 266 mg (89%). ^1^H NMR (400 MHz, CDCl_3_) *δ* 7.39 (d, *J* = 7.7 Hz, 1H), 5.33 (s, 1H), 3.17 (t, *J* = 7.4 Hz, 2H), 2.64 (t, *J* = 7.3 Hz, 2H), 0.51 (s, 6H) ppm. ^11^B NMR (96 MHz, acetone-*d*_6_) *δ* 28.9 ppm. ^13^C NMR (101 MHz, CDCl_3_) *δ* 165.0 (dd, *J* = 252.7, 2.4 Hz), 164.4 (dd, *J* = 248.0, 3.7 Hz), 145.3–143.6 (bm), 130.5 (dd, *J* = 33.7, 3.0 Hz), 117.6, 114.9 (dd, *J* = 21.2, 3.6 Hz), 111.3 (dd, *J* = 24.9, 22.0 Hz), 29.6 (t, *J* = 2.9 Hz), 19.0, −0.7 ppm. ^19^F NMR (376 MHz, CDCl_3_) *δ* −95.45 (d, *J* = 6.7 Hz), −101.54 (t, *J* = 7.1 Hz) ppm. HRMS (ESI, negative ion mode) calcd. for C_11_H_12_BF_2_NO_2_SSi^−^ [M − H]^−^: 298.0335; found: 298.0341. HRMS (ESI, positive ion mode) calcd. for C_11_H_14_BF_2_NO_2_SSi^+^ [M + H]^+^: 300.0492; found: 300.0487.

#### 3-((5,7-Difluoro-3-hydroxy-1,1-dimethyl-1,3-dihydrobenzo[*c*][1,2,5]oxasilaborol-6-yl)thio)propanamide (7)

A solution of potassium carbonate (138 mg, 1.0 mmol) in water (1 mL) was added dropwise to a solution of 1e (246 mg, 1.0 mmol) and acrylamide (71 mg, 1.0 mmol) in a methanol/water mixed solvent system (4 mL, 1 : 1, v/v). The mixture was stirred for 10 minutes at 50 °C. Then it was concentrated under reduced pressure to remove the organic solvent. The aqueous residue was acidified with 1.5 M aq. H_2_SO_4_. Upon addition of the acid, formation of a white solid was observed. The acidic aqueous phase was discarded and the solid residue was washed with water and hexane and vigorously stirred to give the suspension. Then it was filtered and the precipitate was dried *in vacuo* (*ca.* 10^−3^ mbar). The pure product 6 was obtained as a white powder. Yield 254 mg (80%). ^1^H NMR (400 MHz, DMSO-*d*_6_) *δ* 9.45 (s, 1H), 7.49 (d, *J* = 8.3 Hz, 1H), 7.31 (s, 1H), 6.85 (s, 1H), 3.05 (t, *J* = 7.2 Hz, 2H), 2.31 (t, *J* = 7.2 Hz, 2H), 0.40 (s, 6H) ppm. ^11^B NMR (96 MHz, DMSO-*d*_6_) *δ* 28.9 ppm. ^13^C NMR (101 MHz, DMSO-*d*_6_) *δ* 172.2, 164.6 (dd, *J* = 249.6, 3.0 Hz), 164.1 (dd, *J* = 245.5, 4.3 Hz), 145.3, 130.6 (dd, *J* = 33.9, 2.7 Hz), 115.0 (dd, *J* = 21.0, 3.3 Hz), 113.0 (dd, *J* = 25.3, 22.4 Hz), 35.8, 30.1 (t, *J* = 3.0 Hz), −0.3 ppm. ^19^F NMR (376 MHz, DMSO-*d*_6_) *δ* −96.36 (d, *J* = 6.8 Hz), −102.67 (t, *J* = 7.5 Hz) ppm. HRMS (ESI, positive ion mode) calcd. for C_11_H_15_BF_2_NO_3_SSi^+^ [M + H]^+^: 318.0598; found: 318.0595.

#### 3-((5,7-Difluoro-3-hydroxy-1,1-dimethyl-1,3-dihydrobenzo[*c*][1,2,5]oxasilaborol-6-yl)thio)-1-morpholinopropan-1-one (8)

The synthesis was performed as described for 7 starting with 1e (246 mg, 1.0 mmol) and 1-morpholinoprop-2-en-1-one (141 mg, 1.0 mmol). The product was obtained as a white solid. Yield 329 mg (85%). ^1^H NMR (400 MHz, CDCl_3_) *δ* 7.36 (d, *J* = 7.9 Hz, 1H), 6.78 (s, 1H), 3.66 (dd, *J* = 5.7, 3.7 Hz, 4H), 3.62 (d, *J* = 5.0 Hz, 2H), 3.42 (t, *J* = 4.8 Hz, 2H), 3.23 (t, *J* = 7.4 Hz, 2H), 2.63 (t, *J* = 7.4 Hz, 2H), 0.46 (s, 6H) ppm. ^11^B NMR (96 MHz, CDCl_3_) *δ* 29.3 ppm. ^13^C NMR (101 MHz, CDCl_3_) *δ* 169.7, 164.9 (dd, *J* = 251.5, 2.7 Hz), 164.3 (dd, *J* = 246.9, 4.0 Hz), 143.7, 130.3 (dd, *J* = 34.0, 2.8 Hz), 114.7 (dd, *J* = 21.4, 3.4 Hz), 113.3 (dd, *J* = 24.9, 22.4 Hz), 66.8, 66.5, 45.9, 42.2, 33.6, 30.0 (t, *J* = 3.0 Hz), −0.7 ppm. ^19^F NMR (376 MHz, CDCl_3_) *δ* −95.81 (d, *J* = 7.0 Hz), −101.98 (t, *J* = 7.3 Hz) ppm. HRMS (ESI, positive ion mode) calcd. for C_15_H_21_BF_2_NO_4_SSi^+^ [M + H]^+^: 388.1016; found: 388.1014.

#### 3-((5,7-Difluoro-3-hydroxy-1,1-dimethyl-1,3-dihydrobenzo[*c*][1,2,5]oxasilaborol-6-yl)thio)-*N*-(pyrimidin-2-yl)propanamide (9)

The synthesis was performed as described for 7 starting with 1e (246 mg, 1.0 mmol) and *N*-(pyrimidin-2-yl)acrylamide (149 mg, 1.0 mmol). The product was obtained as a white solid. Yield mg 324 (82%). ^1^H NMR (400 MHz, DMSO-*d*_6_) *δ* 10.60 (s, 1H), 9.48 (s, 1H), 8.60 (d, *J* = 4.8 Hz, 2H), 7.50 (d, *J* = 8.2 Hz, 1H), 7.15 (t, *J* = 4.9 Hz, 1H), 3.16 (t, *J* = 7.0 Hz, 2H), 2.82 (t, *J* = 7.0 Hz, 2H), 0.40 (s, 6H) ppm. ^11^B NMR (96 MHz, DMSO-*d*_6_) *δ* 29.0 ppm. ^13^C NMR (101 MHz, DMSO-*d*_6_) *δ* 169.8, 164.6 (dd, *J* = 249.6, 2.8 Hz), 164.0 (dd, *J* = 245.5, 4.2 Hz), 158.6, 157.9, 145.4, 130.5 (dd, *J* = 33.8, 2.5 Hz), 117.0, 115.0 (dd, *J* = 21.1, 3.2 Hz), 112.8 (dd, *J* = 25.4, 22.3 Hz), 37.3, 29.7 (d, *J* = 3.1 Hz), −0.4 ppm. ^19^F NMR (376 MHz, DMSO-*d*_6_) *δ* −96.36 (d, *J* = 6.7 Hz), −102.60 (t, *J* = 7.5 Hz) ppm. HRMS (ESI, positive ion mode) calcd. for C_15_H_17_BF_2_N_3_O_3_SSi^+^ [M + H]^+^: 396.0816; found: 396.0814.

#### 3-((5,7-Difluoro-3-hydroxy-1,1-dimethyl-1,3-dihydrobenzo[*c*][1,2,5]oxasilaborol-6-yl)thio)-*N*-(pyrazin-2-yl)propanamide (10)

The synthesis was performed as described for 7 starting with 1e (246 mg, 1.0 mmol) and *N*-(pyrazin-2-yl)acrylamide (149 mg, 1.0 mmol). The product was obtained as a white solid. Yield 340 mg (86%). ^1^H NMR (400 MHz, CDCl_3_) *δ* 9.50 (s, 1H), 8.57 (s, 1H), 8.35 (d, *J* = 2.6 Hz, 1H), 8.25 (dd, *J* = 2.7, 1.5 Hz, 1H), 7.30 (d, *J* = 7.7 Hz, 1H), 7.08 (s, 1H), 3.30 (t, *J* = 6.9 Hz, 2H), 2.75 (t, *J* = 6.9 Hz, 2H), 0.45 (s, 6H) ppm. ^11^B NMR (96 MHz, CDCl_3_) *δ* 29.1 ppm. ^13^C NMR (101 MHz, CDCl_3_) *δ* 169.4, 165.0 (dd, *J* = 251.9, 2.6 Hz), 164.5 (dd, *J* = 247.7, 3.8 Hz), 147.9, 144.2, 141.9, 140.2, 137.1, 130.3 (dd, *J* = 32.5, 2.0 Hz), 114.6 (dd, *J* = 21.4, 3.4 Hz), 112.5 (dd, *J* = 26.1, 21.6 Hz), 37.8, 29.5 (t, *J* = 2.8 Hz), −0.7 ppm. ^19^F NMR (376 MHz, CDCl_3_) *δ* −95.56 (d, *J* = 6.8 Hz), −101.58 (t, *J* = 7.3 Hz) ppm. HRMS (ESI, positive ion mode) calcd. for C_15_H_17_BF_2_N_3_O_3_SSi^+^ [M + H]^+^: 396.0816; found: 396.0815.

#### 3-((5,7-Difluoro-3-hydroxy-1,1-dimethyl-1,3-dihydrobenzo[*c*][1,2,5]oxasilaborol-6-yl)thio)-*N*-(5-methylisoxazol-3-yl)propanamide (11)

The synthesis was performed as described for 7 starting with 1e (246 mg, 1.0 mmol) and *N*-(5-methylisoxazol-3-yl)acrylamide (152 mg, 1.0 mmol). The product was obtained as a white solid. Yield 358 mg (90%). ^1^H NMR (400 MHz, acetone-*d*_6_) *δ* 10.08 (s, 1H), 8.79 (s, 1H), 7.41 (d, *J* = 8.1 Hz, 1H), 6.57 (s, 1H), 3.26 (t, 2H), 2.77 (t, *J* = 6.9 Hz, 2H), 2.35 (d, *J* = 0.9 Hz, 3H), 0.42 (s, 6H) ppm. ^11^B NMR (96 MHz, acetone-*d*_6_) *δ* 28.8 ppm. ^13^C NMR (101 MHz, acetone-*d*_6_) *δ* 169.4, 169.1 (d, *J* = 9.0 Hz), 164.9 (dd, *J* = 250.1, 2.8 Hz), 164.4 (dd, *J* = 246.0, 4.1 Hz), 158.1 (d, *J* = 9.9 Hz), 130.4 (dd, *J* = 34.1, 2.8 Hz), 114.3 (dd, *J* = 21.2, 3.5 Hz), 112.6 (dd, *J* = 25.3, 22.3 Hz), 96.2 (d, *J* = 3.5 Hz), 36.60, 36.56, 11.5, −1.6 ppm. ^19^F NMR (376 MHz, acetone-*d*_6_) *δ* −96.94 (d, *J* = 6.9 Hz), −103.46 (t, *J* = 7.5 Hz) ppm. HRMS (ESI, positive ion mode) calcd. for C_15_H_18_BF_2_N_2_O_4_SSi^+^ [M + H]^+^: 399.0812; found: 399.0812.

#### 3-((5,7-Difluoro-3-hydroxy-1,1-dimethyl-1,3-dihydrobenzo[*c*][1,2,5]oxasilaborol-6-yl)thio)pyrrolidine-2,5-dione (12)

A solution of potassium carbonate (100 mg, 0.7 mmol) in water (3 mL) was added dropwise to a solution of 1e (152 mg, 0.6 mmol) and maleimide (60 mg, 0.6 mmol) in DCM (3 mL). The mixture was stirred for 0.5 hours at room temperature. Then it was concentrated under reduced pressure to remove the organic solvent. The residue was diluted with acetone (3 mL), acidified with 1.5 M aq. H_2_SO_4_ and stirred for 10 minutes. Then acetone was evaporated and the residual aqueous suspension was filtered. The precipitate was washed with water to give pure 12 as a white solid. Yield 184 mg (87%). ^1^H NMR (400 MHz, DMSO-*d*_6_) *δ* 11.81 (s, 1H), 7.96 (d, *J* = 7.9 Hz, 1H), 4.72 (dd, *J* = 9.2, 3.7 Hz, 1H), 3.59 (dd, *J* = 18.4, 9.0 Hz, 1H), 0.84 (s, 6H) ppm. ^11^B NMR (96 MHz, DMSO-*d*_6_) *δ* 29.3 ppm. ^13^C NMR (101 MHz, DMSO-*d*_6_) *δ* 176.8, 176.4, 166.5–163.5 (m), 165.8–163.0 (m), 147.2, 130.6 (d, *J* = 33.7 Hz), 115.0 (dd, *J* = 20.9, 3.4 Hz), 110.0 (t, *J* = 24.1 Hz), 43.6, 37.2, −0.3 ppm. ^19^F NMR (376 MHz, DMSO-*d*_6_) *δ* −95.14, −101.44 ppm. HRMS (ESI, negative ion mode) calcd. for C_12_H_11_BF_2_NO_4_SSi^−^ [M − H]^−^: 342.0234; found: 342.0246.

#### 3-((7-Fluoro-3-hydroxy-1,1-dimethyl-1,3-dihydrobenzo[*c*][1,2,5]oxasilaborol-6-yl)thio)pyrrolidine-2,5-dione (13)

The synthesis was performed as described for 12 starting with 2e (228 mg, 1.0 mmol) and maleimide (97 mg, 1.0 mmol). The product was obtained as a white solid. Yield 293 mg (90%). ^1^H NMR (400 MHz, CDCl_3_) *δ* 8.46 (s, 1H), 7.63 (dd, *J* = 7.4, 6.8 Hz, 1H), 7.55 (dd, *J* = 7.4, 1.6 Hz, 1H), 5.57 (s, 1H), 4.25 (dd, *J* = 9.1, 4.1 Hz, 1H), 3.22 (dd, *J* = 19.0, 9.2 Hz, 1H), 2.76 (dd, *J* = 18.9, 4.1 Hz, 1H), 0.50 (d, *J* = 0.8 Hz, 6H) ppm. ^11^B NMR (96 MHz, CDCl_3_) *δ* 29.5 ppm. ^13^C NMR (101 MHz, CDCl_3_) *δ* 175.3, 174.5, 163.7 (d, *J* = 246.3 Hz), 137.9, 135.8 (d, *J* = 33.0 Hz), 128.3 (d, *J* = 3.5 Hz), 120.8 (d, *J* = 21.0 Hz), 43.8 (d, *J* = 2.6 Hz), 37.2, −0.8 ppm. ^19^F NMR (376 MHz, CDCl_3_) *δ* −98.70 (dd, *J* = 6.8, 1.3 Hz) ppm. HRMS (ESI, negative ion mode) calcd. for C_12_H_12_BFNO_4_SSi^−^ [M − H]^−^: 324.0328; found: 324.0340.

#### 3-((5,7-Difluoro-3-hydroxy-1,1-dimethyl-1,3-dihydrobenzo[*c*][1,2,5]oxasilaborol-6-yl)thio)-1-(4-fluorophenyl)pyrrolidine-2,5-dione (14)

The synthesis was performed as described for 5 starting with 1e (123 mg, 0.5 mmol) and 1-(4-fluorophenyl)-1*H*-pyrrole-2,5-dione (96 mg, 0.5 mmol). The product was obtained as a white solid. Yield 186 mg (85%). ^1^H NMR (400 MHz, acetone-*d*_6_) *δ* 7.50 (d, *J* = 7.9 Hz, 1H), 7.30–7.21 (m, 4H), 4.55 (dd, *J* = 9.1, 3.7 Hz, 1H), 3.47 (dd, *J* = 18.5, 9.1 Hz, 1H), 2.82 (dd, *J* = 18.5, 3.7 Hz, 1H), 0.44 (d, *J* = 0.9 Hz, 6H) ppm. ^11^B NMR (96 MHz, acetone-*d*_6_) *δ* 29.4 ppm. ^13^C NMR (101 MHz, acetone-*d*_6_) *δ* 174.6, 174.1, 166.1 (dd, *J* = 251.7, 2.0 Hz), 165.6 (dd, *J* = 247.5, 3.4 Hz), 162.8 (d, *J* = 246.0 Hz), 148.0, 131.5 (dd, *J* = 34.7, 2.4 Hz), 129.63 (d, *J* = 8.9 Hz), 129.56 (d, *J* = 3.5 Hz), 116.5 (d, *J* = 23.0 Hz), 115.3 (dd, *J* = 21.1, 3.5 Hz), 110.8–109.8 (m), 43.3 (t, *J* = 2.2 Hz), 36.7, −0.75 (d, *J* = 2.3 Hz) ppm. ^19^F NMR (376 MHz, acetone-*d*_6_) *δ* −95.60 (d, *J* = 5.9 Hz), −102.17 to −102.46 (m), −114.64 (tt, *J* = 8.4, 5.0 Hz) ppm. HRMS (ESI, negative ion mode) calcd. for C_18_H_14_BF_3_NO_4_SSi^−^ [M − H]^−^: 436.0453; found: 436.0462.

#### 3-((7-Fluoro-3-hydroxy-1,1-dimethyl-1,3-dihydrobenzo[*c*][1,2,5]oxasilaborol-6-yl)thio)-1-(4-fluorophenyl)pyrrolidine-2,5-dione (15)

The synthesis was performed as described for 5 starting with 2e (114 mg, 0.5 mmol) and 1-(4-fluorophenyl)-1*H*-pyrrole-2,5-dione (96 mg, 0.5 mmol). The product was obtained as a white solid. Yield 175 mg (83%). ^1^H NMR (400 MHz, acetone-*d*_6_) *δ* 7.75 (t, *J* = 7.2 Hz, 1H), 7.65 (dd, *J* = 7.4, 1.7 Hz, 1H), 7.30–7.20 (m, 4H), 4.64 (dd, *J* = 9.3, 4.2 Hz, 1H), 3.52 (dd, *J* = 18.5, 9.3 Hz, 1H), 2.87 (dd, *J* = 18.5, 4.2 Hz, 1H), 0.43 (d, *J* = 2.3 Hz, 6H) ppm. ^11^B NMR (96 MHz, acetone-*d*_6_) *δ* 29.2 ppm. ^13^C NMR (101 MHz, acetone-*d*_6_) *δ* 174.2, 173.4, 163.2 (d, *J* = 243.8 Hz), 162.0 (d, *J* = 245.8 Hz), 136.8, 135.5 (d, *J* = 32.8 Hz), 128.9 (d, *J* = 8.8 Hz), 128.8 (d, *J* = 3.1 Hz), 128.2 (d, *J* = 3.4 Hz), 121.8 (d, *J* = 21.0 Hz), 115.6 (d, *J* = 23.0 Hz), 42.8 (d, *J* = 1.9 Hz), 36.3, −1.6 (d, *J* = 1.9 Hz) ppm. ^19^F NMR (376 MHz, acetone-*d*_6_) *δ* −100.69 (dd, *J* = 7.0, 1.6 Hz), −114.64 (tt, *J* = 8.2, 5.5 Hz) ppm. HRMS (ESI, negative ion mode) calcd. for C_18_H_15_BF_2_NO_4_SSi^−^ [M − H]^−^: 418.0547; found: 418.0559.

#### 1-(4-Bromo-3-(trifluoromethyl)phenyl)-3-((5,7-difluoro-3-hydroxy-1,1-dimethyl-1,3-dihydrobenzo[*c*][1,2,5]oxasilaborol-6-yl)thio)pyrrolidine-2,5-dione (16)

The synthesis was performed as described for 5 starting with 1e (123 mg, 0.5 mmol) and 1-(4-bromo-3-(trifluoromethyl)phenyl)-1*H*-pyrrole-2,5-dione (160 mg, 0.5 mmol). The product was obtained as a white solid. Yield 240 mg (85%). ^1^H NMR (400 MHz, acetone-*d*_6_) *δ* 8.00 (d, *J* = 8.5 Hz, 1H), 7.71 (d, *J* = 2.5 Hz, 1H), 7.53 (d, *J* = 9.5, 2.5 Hz, 1H), 7.50 (d, *J* = 7.9 Hz, 1H), 4.58 (dd, *J* = 9.1, 3.8 Hz, 1H), 3.59–3.45 (m, 1H), 2.89 (dd, *J* = 18.6, 3.8 Hz, 1H), 0.43 (s, 6H) ppm. ^11^B NMR (96 MHz, acetone-*d*_6_) *δ* 29.9 ppm. ^13^C NMR (101 MHz, acetone-*d*_6_) *δ* 174.3, 173.7, 166.1 (dd, *J* = 251.5, 2.7 Hz), 165.6 (dd, *J* = 247.5, 3.5 Hz), 148.1 (broad), 136.7, 133.1, 132.6, 131.5 (dd, *J* = 34.1, 3.1 Hz), 130.8 (q, *J* = 31.6 Hz), 126.8 (q, *J* = 5.7 Hz), 123.6 (q, *J* = 272.8 Hz), 119.6 (d, *J* = 1.9 Hz), 115.3 (dd, *J* = 21.1, 3.4 Hz), 110.6–110.1 (m), 43.5 (t, *J* = 2.2 Hz), 36.9, −0.79 (d, *J* = 1.9 Hz) ppm. ^19^F NMR (376 MHz, acetone-*d*_6_) *δ* −63.34, −95.65 (d, *J* = 5.6 Hz), −102.32 (dd, *J* = 7.9, 5.6 Hz) ppm. HRMS (ESI, negative ion mode) calcd. for C_19_H_13_BBrF_5_NO_4_SSi^−^ [M − H]^−^: 563.9526; found: 563.9535.

#### 1-(4-Bromo-3-(trifluoromethyl)phenyl)-3-((7-fluoro-3-hydroxy-1,1-dimethyl-1,3-dihydrobenzo[*c*][1,2,5]oxasilaborol-6-yl)thio)pyrrolidine-2,5-dione (17)

The synthesis was performed as described for 5 starting with 2e (114 mg, 0.5 mmol) and 1-(4-bromo-3-(trifluoromethyl)phenyl)-1*H*-pyrrole-2,5-dione (160 mg, 0.5 mmol). The product was obtained as a white solid. Yield 216 mg (79%). ^1^H NMR (400 MHz, acetone-*d*_6_) *δ* 7.99 (dd, *J* = 8.5, 0.7 Hz, 1H), 7.78–7.73 (m, 1H), 7.67 (d, *J* = 2.5 Hz, 2H), 7.65 (dd, *J* = 7.5, 1.7 Hz, 1H), 7.50 (ddq, *J* = 8.5, 2.4, 0.6 Hz, 1H), 4.67 (dd, *J* = 9.3, 4.3 Hz, 1H), 3.56 (dd, *J* = 18.1, 9.3 Hz, 1H), 2.94 (dd, *J* = 18.6, 4.3 Hz, 1H), 0.42–0.41 (m, 6H) ppm. ^11^B NMR (96 MHz, acetone-*d*_6_) *δ* 29.7 ppm. ^13^C NMR (101 MHz, acetone-*d*_6_) *δ* 174.7, 173.8, 164.1 (d, *J* = 244.0 Hz), 146.2 (broad), 137.8, 136.7, 136.7 (d, *J* = 20.8 Hz), 133.2, 132.7, 130.7 (q, *J* = 31.6 Hz), 129.1 (d, *J* = 3.3 Hz), 127.0 (q, *J* = 5.7 Hz), 123.6 (q, *J* = 272.8 Hz), 122.5 (d, *J* = 21.0 Hz), 119.6 (q, *J* = 2.0 Hz), 43.9 (d, *J* = 2.2 Hz), 37.3, −0.8 (d, *J* = 1.3 Hz) ppm. ^19^F NMR (376 MHz, acetone-*d*_6_) *δ* −63.30, −100.61 (dd, *J* = 7.1, 1.6 Hz) ppm. HRMS (ESI, negative ion mode) calcd. for C_19_H_14_BBrF_4_NO_4_SSi^−^ [M − H]^−^: 545.9620; found: 545.9629.

#### 1-(6-((3-((5,7-difluoro-3-hydroxy-1,1-dimethyl-1,3-dihydrobenzo[*c*][1,2,5]oxasilaborol-6-yl)thio)propanoyl)oxy)hexyl)pyridin-1-ium trifluoromethanesulfonate (18)

To a solution of *N*-(6-hydroxyhex-1-yl)pyridinium triflate (1.32 g, 4.0 mmol) in THF (20 mL) was added Et_3_N (0.70 mL, 5.0 mmol) followed by dropwise addition of acryloyl chloride (0.32 mL, 4.0 mmol) at 0 °C. The mixture was stirred for 2 h and the mixture was quenched with saturated aq. NaHCO_3_ (10 mL). The organic phase was separated, washed with brine and dried with anhydrous Na_2_SO_4_. The solvent was removed to leave the viscous residue which was dissolved in MeOH (5 mL) and a solution of 1e (492 mg, 2.0 mmol) in MeOH (5 mL) was added followed by the addition of a solution of K_2_CO_3_ (0.28 g, 2.0 mmol) in water (3 mL). The mixture was stirred for 6 h at rt followed by removal of MeOH under reduced pressure. The aqueous phase was discarded and the remaining viscous residue was washed with water (2 × 5 mL) and dissolved in acetone (10 mL). Water (10 mL) was added and the solution was concentrated to remove acetone. The aqueous phase was decanted from over the viscous residue which was again dissolved in a mixture of acetone (10 mL) and water (10 mL). Evaporation of acetone and removal of the aqueous phase followed by drying *in vacuo* afforded the product as a pale yellow viscous material. Yield 1.045 g (83%). ^1^H NMR (400 MHz, DMSO-*d*_6_) *δ* 9.49 (s, 1H), 9.08 (dt, *J* = 6.1, 1.4 Hz, 2H), 8.60 (tt, *J* = 7.8, 1.4 Hz, 1H), 8.18–8.13 (m, 2H), 7.50 (d, *J* = 8.1 Hz, 1H), 4.58 (t, *J* = 7.5 Hz, 3H), 3.95 (t, *J* = 6.6 Hz, 2H), 3.11 (t, *J* = 6.7 Hz, 2H), 2.56 (t, *J* = 6.7 Hz, 2H), 1.91 (p, *J* = 7.6 Hz, 2H), 1.52 (p, *J* = 6.7 Hz, 2H), 1.36–1.24 (m, 4H), 0.41 (s, 6H) ppm. ^11^B NMR (96 MHz, DMSO-*d*_6_) *δ* 29.0 ppm. ^13^C NMR (101 MHz, DMSO-*d*_6_) *δ* 170.9, 164.3 (dd, *J* = 249.5, 2.7 Hz), 163.7 (dd, *J* = 245.5, 3.8 Hz), 145.5, 144.7, 130.4, 128.1, 120.7 (q, *J* = 322.3 Hz), 114.6 (dd, *J* = 20.9, 3.1 Hz), 112.2–111.4 (m), 63.9, 60.7, 34.5, 30.6, 29.2, 27.8, 25.0, 24.7, −0.7 ppm. ^19^F NMR (376 MHz, DMSO-*d*_6_) *δ* −77.79 (s, 3F), −96.23 (d, *J* = 6.9 Hz, 1F), −102.57 (t, *J* = 7.4 Hz, 1F) ppm. HRMS (ESI, positive ion mode) calcd. for C_22_H_29_BF_2_NO_4_SSi^+^ [M–OTf]^+^: 480.1642; found: 480.1643.

#### 1-(6-((3-((7-Fluoro-3-hydroxy-1,1-dimethyl-1,3-dihydrobenzo[*c*][1,2,5]oxasilaborol-6-yl)thio)propanoyl)oxy)hexyl)pyridin-1-ium trifluoromethanesulfonate (19)

The synthesis was performed as described for 18 using 2e (456 mg, 2.0 mmol). The product was obtained as a pale yellow viscous material. Yield 0.97 g (79%). ^1^H NMR (400 MHz, DMSO-*d*_6_) *δ* 9.30 (broad s, 1H), 9.08 (dd, *J* = 6.8, 1.3 Hz, 2H), 8.60 (tt, *J* = 7.8, 1.4 Hz, 1H), 8.18–8.13 (m, 2H), 7.63 (dd, *J* = 7.5, 1.7 Hz, 1H), 7.52 (t, *J* = 7.5 Hz, 1H), 4.59 (t, *J* = 7.4 Hz, 2H), 4.01 (t, *J* = 6.6 Hz, 2H), 3.22 (t, *J* = 6.9 Hz, 2H), 2.65 (t, *J* = 6.9 Hz, 2H), 1.92 (p, *J* = 7.6 Hz, 2H), 1.55 (p, *J* = 6.7 Hz, 2H), 1.37–1.25 (m, 4H), 0.40 (s, 6H) ppm. ^11^B NMR (96 MHz, DMSO-*d*_6_) *δ* 28.9 ppm. ^13^C NMR (101 MHz, DMSO-*d*_6_) *δ* 171.1, 160.9 (d, *J* = 241.2 Hz), 145.5, 144.7, 141.5 (broad), 134.4 (d, *J* = 31.8 Hz), 131.5, 128.5 (d, *J* = 3.0 Hz), 128.1, 125.3 (d, *J* = 19.9 Hz), 120.7 (q, *J* = 322.3 Hz), 64.0, 60.7, 33.6, 30.6, 27.8, 26.4, 25.0, 24.7, −0.7 ppm. ^19^F NMR (376 MHz, DMSO-*d*_6_) *δ* −77.79 (s, 3F), −103.03 (dd, *J* = 7.5, 1.9 Hz, 1F) ppm. HRMS (ESI, positive ion mode) calcd. for C_22_H_30_BFNO_4_SSi^+^ [M–OTf]^+^: 462.1736; found: 462.1733.

#### 5,7-Difluoro-1,1-dimethyl-6-((2-(phenylsulfonyl)ethyl)thio)benzo[*c*][1,2,5]oxasilaborol-3(1*H*)-ol (20)

The synthesis was performed as described for 6 starting with 1e (132 mg, 0.54 mmol) and phenyl vinyl sulfone (90 mg, 0.54 mmol). The product 20 was obtained as a white powder. Yield 179 mg (81%).^1^H NMR (400 MHz, CDCl_3_) *δ* 8.11–7.82 (m, 2H), 7.73–7.64 (m, 1H), 7.58 (dd, *J* = 8.3, 7.0 Hz, 2H), 7.31 (d, *J* = 7.7 Hz, 1H), 5.52 (s, 1H), 3.38–3.10 (m, 4H), 0.48 (s, 6H) ppm. ^11^B NMR (96 MHz, CDCl_3_) *δ* 29.6 ppm. ^13^C NMR (101 MHz, CDCl_3_) *δ* 164.9 (dd, *J* = 252.6, 2.2 Hz), 164.2 (dd, *J* = 248.1, 3.8 Hz), 138.4, 134.1, 130.6 (dd, *J* = 34.0, 2.4 Hz), 129.5, 128.1, 114.8 (dd, *J* = 21.4, 2.7 Hz), 111.5 (dd, *J* = 26.4, 21.2 Hz), 56.5, 26.8 (t, *J* = 3.0 Hz), −0.7 ppm. ^19^F NMR (376 MHz, CDCl_3_) *δ* −95.54 (d, *J* = 6.3 Hz), −101.60 (t, *J* = 7.0 Hz) ppm. HRMS (ESI, negative ion mode) calcd. for C_16_H_16_BF_2_O_4_S_2_Si^−^ [M − H]^−^: 413.0315; found: 413.0326.

#### 7-Fluoro-1,1-dimethyl-6-((2-(phenylsulfonyl)ethyl)thio)benzo[*c*][1,2,5]oxasilaborol-3(1*H*)-ol (21)

The synthesis was performed as described for 6 starting with 2e (228 mg, 1.0 mmol) and phenyl vinyl sulfone (168 mg, 1.0 mmol). The product 21 was obtained as a white sticky solid. Yield 284 mg (72%). ^1^H NMR (400 MHz, CDCl_3_) *δ* 7.98–7.84 (m, 2H), 7.75–7.66 (m, 1H), 7.67–7.55 (m, 2H), 7.48 (dd, *J* = 7.4, 1.5 Hz, 1H), 7.29 (dd, *J* = 7.4, 7.0 Hz, 1H), 5.12 (s, 1H), 3.41–3.19 (m, 4H), 0.47 (s, 6H) ppm. ^11^B NMR (96 MHz, CDCl_3_) *δ* 29.4 ppm. ^13^C NMR (101 MHz, CDCl_3_) *δ* 162.6 (d, *J* = 244.9 Hz), 142.1, 138.5, 135.5 (d, *J* = 32.4 Hz), 134.2, 133.5, 129.5, 128.2 (d, *J* = 3.4 Hz), 128.1, 123.6 (d, *J* = 20.5 Hz), 55.8, 25.3 (d, *J* = 2.8 Hz), −0.7 ppm. ^19^F NMR (376 MHz, CDCl_3_) *δ* −100.91 (dd, J = 6.8, 1.9 Hz) ppm. HRMS (ESI, negative ion mode) calcd. for C_16_H_17_BFO_4_S_2_Si^−^ [M − H]^−^: 395.0409; found: 395.0421.

#### 1-((7-Fluoro-3-hydroxy-1,1-dimethyl-1,3-dihydrobenzo[*c*][1,2,5]oxasilaborol-6-yl)thio)propan-2-one (22)

The synthesis was performed as described for 5 starting with 2e (228 mg, 1.0 mmol) and bromoacetone (205 mg, 1.5 mmol). The product was obtained as a pale yellow solid. Yield 164 mg (58%). ^1^H NMR (400 MHz, acetone-*d*_6_) *δ* 7.58 (dd, *J* = 7.5, 1.7 Hz, 1H), 7.46 (t, *J* = 7.4 Hz, 1H), 4.01 (s, 2H), 2.28 (s, 3H), 0.44–0.43 (m, 6H) ppm. ^11^B NMR (96 MHz, acetone-*d*_6_) *δ* 28.9 ppm. ^13^C NMR (101 MHz, acetone-*d*_6_) *δ* 201.6, 161.5 (d, *J* = 242.0 Hz), 134.8 (d, *J* = 32.1 Hz), 131.9, 128.0 (d, *J* = 3.2 Hz), 125.5 (d, *J* = 19.9 Hz), 42.1 (d, *J* = 1.9 Hz), 27.4, −1.6 ppm. ^19^F NMR (376 MHz, acetone-*d*_6_) *δ* −103.66 (dd, *J* = 7.1, 1.6 Hz) ppm. HRMS (ESI, negative ion mode) calcd. for C_11_H_13_BFO_3_SSi^−^ [M − H]^−^: 283.0426; found: 283.0437.

#### 2-((7-Fluoro-3-hydroxy-1,1-dimethyl-1,3-dihydrobenzo[*c*][1,2,5]oxasilaborol-6-yl)thio)-1-phenylethan-1-one (23)

The synthesis was performed as described for 5 starting with 2e (228 mg, 1.0 mmol) and 2-bromoacetophenone (299 mg, 1.5 mmol). The product was obtained as an yellow solid. Yield 153 mg (47%). ^1^H NMR (400 MHz, acetone-*d*_6_) *δ* 8.15 (s, 1H), 8.11–8.03 (m, 2H), 7.70–7.63 (m, 1H), 7.59–7.51 (m, 4H), 4.69–4.65 (m, 2H), 0.44 (s, 6H) ppm. ^11^B NMR (96 MHz, acetone-*d*_6_) *δ* 28.8 ppm. ^13^C NMR (101 MHz, acetone-*d*_6_) *δ* 193.4, 161.7 (d, *J* = 242.2 Hz), 135.7, 134.7 (d, *J* = 32.1 Hz), 133.4, 132.5, 132.2, 128.6 (d, *J* = 14.4 Hz), 128.0 (d, *J* = 3.1 Hz), 125.5 (d, *J* = 19.9 Hz), 38.9 (d, *J* = 1.9 Hz), −1.6 ppm. ^19^F NMR (376 MHz, acetone-*d*_6_) *δ* −103.33 (dd, *J* = 6.1, 2.8 Hz) ppm. HRMS (ESI, negative ion mode) calcd. for C_16_H_15_BFO_3_SSi^−^ [M − H]^−^: 345.0583; found: 345.0598. HRMS (ESI, positive ion mode) calcd. for C_16_H_17_BFO_3_SSi^+^ [M + H]^+^: 347.0739; found: 347.0739.

#### *S*-(5,7-Difluoro-3-hydroxy-1,1-dimethyl-1,3-dihydrobenzo[*c*][1,2,5]oxasilaborol-6-yl) *O*,*O*-diethyl phosphorothioate (24)

To a solution of 1e (246 mg, 1.0 mmol) in MeCN (3 mL), potassium carbonate (450 mg, 3.3 mmol) and diethyl chlorophosphate (0.35 ml, 417 mg, 2.4 mmol) were added respectively. The mixture was stirred for 60 hours at room temperature. Then it was concentrated under reduced pressure to remove the organic solvent. The residue was diluted with water and acidified with 1.5 M aq. H_2_SO_4_. Upon addition of the acid, the formation of the pale thick oil was observed. It was dissolved in Et_2_O (15 mL). The acidic aqueous phase was separated followed by extraction with Et_2_O (2 × 10 ml). The extracts were added to the organic phase, which was dried over anhydrous MgSO_4_. The solvent was removed under reduced pressure. The crude product was recrystallized under mild conditions with the mixture of water, DCM and heptane, filtered and dried *in vacuo* (10^−3^ mbar) to give pure product 24 as a white powder. Yield 115 mg (30%). ^1^H NMR (400 MHz, CDCl_3_) *δ* 7.34 (d, *J* = 7.3 Hz, 1H), 4.99 (s, 1H), 4.37–4.28 (m, 4H), 1.37 (td, *J* = 7.0, 1.1 Hz, 6H), 0.47 (s, 6H) ppm. ^11^B NMR (96 MHz, CDCl_3_) *δ* 28.5 ppm. ^13^C NMR (101 MHz, CDCl_3_) *δ* 165.2 (dd, *J* = 254.8, 4.2 Hz), 164.7 (dd, *J* = 252.6, 4.7 Hz), 146.9–146.0 (bm), 130.5 (d, *J* = 34.4 Hz), 120.2–108.9 (m), 105.2–104.4 (m), 64.6 (d, *J* = 5.6 Hz), 15.8 (d, *J* = 8.0 Hz), −0.8 ppm. ^19^F NMR (376 MHz, CDCl_3_) *δ* −94.49 (t, *J* = 3.8 Hz), −100.15 (dd, *J* = 7.7, 4.3 Hz) ppm. ^31^P NMR (162 MHz, CDCl_3_) *δ* 20.5 (t, *J* = 3.7 Hz) ppm. HRMS (ESI, negative ion mode) calcd. for C_12_H_17_BF_2_O_5_PSSi^−^ [M − H]^−^: 381.0360; found: 381.0372. HRMS (ESI, positive ion mode) calcd. for C_12_H_19_BF_2_O_5_PSSi^+^ [M + H]^+^: 383.0516; found: 383.0512.

#### *S*-(7-Fluoro-3-hydroxy-1,1-dimethyl-1,3-dihydrobenzo[*c*][1,2,5]oxasilaborol-6-yl) 4-(trifluoromethyl)benzothioate (25)

Compound 2e (228 mg, 1.0 mmol) was dissolved in DCM (3 mL). The solution was cooled to 0 °C followed by consecutive addition of Et_3_N (0.2 mL) and *p*-CF_3_PhCOCl (1.1 mmol). The mixture was stirred for 1 h and then the solvent was removed under reduced pressure. The residue was mixed with water (3 mL) and hexane (5 mL) resulting in a white suspension. It was acidified with a few drops of aq. H_2_SO_4_ and stirred for 30 min. Then it was filtered and washed with water and hexane to give pure 25 as a white powder. Yield 0.37 g (92%). ^1^H NMR (500 MHz, CDCl_3_) *δ* 8.17–8.13 (m, 2H), 7.79–7.76 (m, 2H), 7.68 (dd, *J* = 7.3, 1.5 Hz, 1H), 7.62 (dd, *J* = 7.4, 6.3 Hz, 1H), 5.27 (broad, 1H), 0.53 (s, 6H) ppm. ^11^B NMR (96 MHz, CDCl_3_) *δ* 28.7 ppm. ^13^C NMR (101 MHz, DMSO-*d*_6_) *δ* 182.6, 159.1 (d, *J* = 249.5 Hz), 134.6, 134.3, 131.3 (d, *J* = 32.9 Hz), 130.4 (d, *J* = 32.8 Hz), 123.3 (d, *J* = 3.6 Hz), 123.3, 121.2 (q, *J* = 3.7 Hz), 120.0, 117.3, 111.6 (d, *J* = 21.7 Hz), −5.5 (d, *J* = 16.6 Hz) ppm. ^19^F NMR (376 MHz, DMSO-*d*_6_) *δ* −67.95, −102.87 (d, *J* = 6.3 Hz) ppm. HRMS (ESI, negative ion mode) calcd. for C_16_H_12_BF_4_O_3_SSi^−^ [M − H]^−^: 399.0300; found: 399.0312.

#### 5,7-Difluoro-3-hydroxy-1,1-dimethyl-1,3-dihydrobenzo[*c*][1,2,5]oxasilaborole-6-sulfonyl chloride (26)

Compound 1e (738 mg, 3.0 mmol) was dissolved in the mixture of MeCN (12 mL) and water (3 mL). The solution was cooled to 0 °C followed by slow addition of trichloroisocyanuric acid (1.011 g, 4.4 mmol) in portions. The precipitation of a white solid was observed. The mixture was stirred for 30 min. Then the suspension was filtered and the filter cake was washed with ethyl acetate (2 × 9 ml). The filtrate was transferred to the separatory funnel and the layers were separated. The organic phase was dried over anhydrous MgSO_4_. The solvent was removed under reduced pressure and the solid residue was washed with hexane and filtered to give pure product 26 as a white powder. Yield 0.734 g (78%). ^1^H NMR (400 MHz, CDCl_3_) *δ* 7.52 (d, *J* = 8.8 Hz, 1H), 0.54 (s, 6H) ppm. ^11^B NMR (96 MHz, CDCl_3_) *δ* 28.7 ppm. ^13^C NMR (101 MHz, CDCl_3_) *δ* 160.6 (d, *J* = 269.7 Hz), 159.3 (d, *J* = 264.5 Hz), 32.8 (d, *J* = 36.0 Hz), 123.3–122.6 (m), 116.6 (dd, *J* = 19.8, 4.0 Hz), −0.9 ppm. ^19^F NMR (376 MHz, CDCl_3_) *δ* −95.5, −101.9 (d, *J* = 8.9 Hz) ppm. HRMS (ESI, negative ion mode) calcd. for C_8_H_7_BClF_2_O_4_SSi^−^ [M − H]^−^: 310.9579; found: 310.9586.

#### 5,7-Difluoro-3-hydroxy-1,1-dimethyl-1,3-dihydrobenzo[*c*][1,2,5]oxasilaborole-6-sulfonamide (27)

Compound 26 (313 mg, 1.0 mmol) was dissolved in DCM (6 mL). The solution was cooled to −30 °C followed by a consecutive addition of ammonia, introduced into the solution through a hose, which was generated *in situ* as a result of a parallel reaction of potassium carbonate (10 g) with ammonium carbonate (10 g) at 60 °C in a separate flask. The mixture was stirred for 0.5 hours at −30 °C. Then it was allowed to warm to the room temperature, warmed up and stirred at 35 °C for another 0.5 hours. The resulting suspension was diluted with water and acidified with a few drops of conc. aq. HCl. Upon addition of the acid, the precipitate was dissolved. Then the organic solvent was removed under reduced pressure. The residual suspension was filtered and the first fraction of pure product (white solid) was collected. The filtrate was concentrated *in vacuo* (*ca.* 1 mbar) to give an orange solid residue. It was washed several times with AcOEt and MeOH and filtered. The filtrate was then concentrated under reduced pressure to give a green solid residue. It was washed in hexane and consequently dissolved with AcOEt. Undissolved ammonium chloride was then removed through filtration. The filtrate was concentrated *in vacuo* and the residue was washed with hexane. The resulting suspension was then filtered to give the second fraction of pure product (white solid). Both fractions containing pure product 27 were combined together. Yield 42 mg + 55 mg (33%). ^1^H NMR (400 MHz, DMSO-*d*_6_) *δ* 9.62 (s, 1H), 8.01 (s, 2H), 7.59 (d, *J* = 9.5 Hz, 1H), 0.46 (s, 6H) ppm. ^11^B NMR (96 MHz, DMSO-*d*_6_) δ 28.7 ppm. ^13^C NMR (101 MHz, DMSO-*d*_6_) *δ* 160.6 (dd, *J* = 258.5, 1.9 Hz), 159.6 (dd, *J* = 253.6, 4.2 Hz), 149.5, 132.0 (d, *J* = 31.5 Hz), 122.5 (d, *J* = 18.7 Hz), 116.1 (dd, *J* = 20.9, 3.3 Hz), −0.3 ppm. ^19^F NMR (376 MHz, DMSO-*d*_6_) *δ* −98.62 (d, *J* = 4.0 Hz), −105.59 (dd, *J* = 9.6, 3.9 Hz) ppm. HRMS (ESI, negative ion mode) calcd. for C_8_H_9_BF_2_NO_4_SSi^−^ [M − H]^−^: 292.0077; found: 292.0090.

#### 5,7-Difluoro-*N*-(4-fluorophenyl)-3-hydroxy-1,1-dimethyl-1,3-dihydrobenzo[*c*][1,2,5]oxasilaborole-6-sulfonamide (28)

Compound 26 (156 mg, 0.5 mmol) was dissolved in DCM (3 mL). The solution was cooled to 0 °C followed by a consecutive dropwise addition of a solution of 4-fluoroaniline (139 mg, 1.3 mmol) in DCM (1 mL). The mixture was stirred for 0.5 hours at 0 °C. Then it was allowed to warm to room temperature and warmed up and stirred at 35 °C for another 0.5 hours. The resulting suspension was concentrated under reduced pressure. The residue was suspended in water and acidified with conc. aq. HCl. The resulting precipitate was filtered and washed with water and hexane to give pure product 28 as a creamy white solid. Yield 157 mg (81%). ^1^H NMR (400 MHz, CDCl_3_) *δ* 7.36 (d, *J* = 9.2 Hz, 1H), 7.17–7.10 (m, 2H), 6.98–6.89 (m, 3H), 0.44 (s, 6H) ppm. ^11^B NMR (96 MHz, CDCl_3_) *δ* 28.7 ppm. ^13^C NMR (101 MHz, CDCl_3_) *δ* 161.10 (d, *J* = 220.2 Hz), 160.97 (d, *J* = 246.2 Hz), 160.63 (dd, *J* = 257.4, 2.5 Hz), 132.4 (d, *J* = 38.2 Hz), 131.3 (d, *J* = 2.8 Hz), 124.1 (d, *J* = 8.4 Hz), 116.4 (d, *J* = 22.9 Hz), 115.9 (dd, *J* = 21.0, 3.7 Hz), −0.9 ppm. ^19^F NMR (376 MHz, CDCl_3_) *δ* −97.64 (d, *J* = 2.3 Hz), −105.00 (dd, *J* = 9.5, 2.4 Hz), −115.34 (tt, *J* = 8.3, 4.6 Hz) ppm. HRMS (ESI, negative ion mode) calcd. for C_14_H_12_BF_3_NO_4_SSi^−^ [M − H]^−^: 386.0296; found: 386.0309.

#### 5,7-Difluoro-1,1-dimethyl-6-(morpholinosulfonyl)benzo[*c*][1,2,5]oxasilaborol-3(1*H*)-ol (29)

The synthesis was performed as described for 28 starting with 26 (156 mg, 0.5 mmol) and morpholine (96 mg, 1.3 mmol). The product 29 was obtained as a white solid. Yield 134 mg (71%). ^1^H NMR (400 MHz, DMSO-*d*_6_) *δ* 9.68–9.43 (m, 1H), 7.57 (d, *J* = 9.7 Hz, 1H), 3.63–3.53 (m, 7H), 3.02 (t, *J* = 4.7 Hz, 4H), 0.37 (s, 6H) ppm. ^11^B NMR (96 MHz, DMSO-*d*_6_) *δ* 28.8 ppm. ^13^C NMR (101 MHz, DMSO-*d*_6_) *δ* 160.8 (d, *J* = 260.1 Hz), 160.0 (dd, *J* = 255.9, 3.2 Hz), 151.6, 132.0 (d, *J* = 35.1 Hz), 116.7–115.6 (m), 114.8–113.7 (m), 65.5, 45.3, −0.8 ppm. ^19^F NMR (376 MHz, DMSO-*d*_6_) *δ* −95.82, −103.04 (d, *J* = 9.6 Hz) ppm. HRMS (ESI, positive ion mode) calcd. for C_12_H_17_BF_2_NO_5_SSi^+^ [M + H]^+^: 364.0652; found: 364.0651.

### Determination of acidity constants (p*K*_a_ values)

The acidity constants of benzosiloxaboroles 1e, 2e, 6, 20 and 22–24 were determined by standard pH titration with 0.05 M aq. NaOH. The measurements were performed using a thermostated (25.0 °C) glass vessel equipped with a magnetic stirrer and a 10 mL burette. Prior to the measurement, the pH glass electrode was calibrated using borax and phosphate buffers. A solution of the analyzed compound (*ca.* 20–40 mg) in a mixture of MeOH/H_2_O (v/v 1 : 1, 30 mL) was titrated until the pH of the solution exceeded the value of 12.0.

### Antimicrobial activity

Direct antimicrobial activity was determined against the following standard strains: (1) Gram-positive cocci: methicillin-sensitive *Staphylococcus aureus* ATCC 6538P (MSSA), methicillin-resistant *S. aureus* subsp. *aureus* ATCC 43300 (MRSA), *S. epidermidis* ATCC 12228, *Enterococcus faecium* ATCC 6057, *E. faecalis* ATCC 29212, *Bacillus subtilis* ATCC 6633; (2) Gram-negative bacteria from *Enterobacteriales* order: *Klebsiella pneumoniae* ATCC 13883, *Escherichia coli* ATCC 25922, *Enterobacter cloacae* DSM 6234, *Proteus mirabilis* ATCC 12453, *Serratia marcescens* ATCC 13880; (3) Gram-negative non-fermentative rods: *Acinetobacter baumannii* ATCC 19606, *Pseudomonas aeruginosa* ATCC 27853, *Stenotrophomonas maltophilia* ATCC 12714, *S. maltophilia* ATCC 13637, *Burkholderia cepacia* ATCC 25416, *Bordetella bronchiseptica* ATCC 4617; (4) yeasts: *Candida albicans* ATCC 90028, *C. krusei* ATCC 6258, *C. parapsilosis* ATCC 22019, *C. tropicalis* (Castellani) Berkhout ATCC 750, *C. tropicalis* IBA 171, *C. guilliermondii* IBA 155, and *Saccharomyces cerevisiae* ATCC 9763. Moreover, 5 clinical strains of methicillin-resistant *S. aureus* were also included: NMI 664 K, NMI 1576 K, NMI 1712 K, NMI 1991 K, and NMI 2541 K. All strains were stored at −80 °C. Prior to testing, each bacterial strain was subcultured twice on tryptic soy agar TSA (bioMerieux) medium and yeast strains on Sabouraud dextrose agar (bioMerieux) for 24–48 h at 30 °C to ensure viability. The antimicrobial activity was evaluated as previously described^26^ by the disc-diffusion method and the MIC determination assays according to the EUCAST (ref. 71 and 72) and CLSI (ref. 53 and 73) recommendations. Assessment of bactericidal (MBC) and fungicidal (MFC) activities was performed according to the CLSI recommendations.^54,74^ The following reference agents were used: fluconazole (in the case of fungi), linezolid (for Gram-positive bacteria), and nitrofurantoin (for Gram-negative rods). The new benzosiloxaboroles were dissolved in DMSO (Sigma). Depending on the solubility, the MIC and MBC/MFC values were determined up to 50 mg L^−1^ for agent 23, up to 100 μg mL^−1^ for agent 2e, up to 200 μg mL^−1^ for agents 14, 15, 16, and 25, and up to 400 μg mL^−1^ for the remaining compounds.

Considering that the MDR efflux pumps often contribute to Gram-negative rod resistance, the MIC values of studied agents, with or without the pump inhibitor, PAβN (20 μg mL^−1^) (Sigma), were determined.^75^ The MIC was determined using Mueller–Hinton II broth medium (MHB) (Becton Dickinson) by the broth microdilution method, according to the CLSI guideliness.^53^ To minimize the influence of PAβN on the destabilization of bacterial cell covers, the tests were conducted in the presence of 1 mM MgSO_4_ (Sigma).^76^ At least a 4-fold reduction in the MIC value after the addition of PAβN was considered significant.^56,77^

### Cytotoxicity studies

MRC-5 pd30 human fibroblasts (ECACC) were cultured in MEME, Minimum Essential Medium Eagle (Merck), supplemented with 10% fetal bovine serum (Merck), 2 mM l-glutamine, antibiotics (100 U mL^−1^ penicillin, 100 μg mL^−1^ streptomycin, Merck) and 1% non-essential amino acids (Merck). Cells were grown in 75 cm^2^ cell culture flasks (Sarstedt), in a humidified atmosphere of CO_2_/air (5/95%) at 37 °C. MTT-based viability assay was conducted as described previously.^26^ Optical densities were measured at 570 nm using a BioTek microplate reader. All measurements were carried out in three replicates and the results expressed as a percent of viable cells *versus* control cells. Cisplatin (0.5–30 μg mL^−1^) and linezolid (6.25–400 μg mL^−1^) were used as the positive and negative control, respectively. The selectivity index (SI) was calculated as 
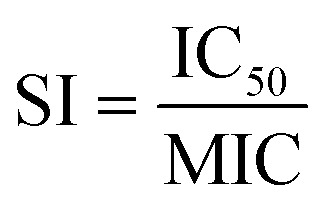
, in accordance with previously described protocols.^60^

### Studies on the mechanism of antibacterial action

#### Cloning, expression, and purification of *Staphylococcus aureus* LeuRS

The LeuRS gene from MRSA (PW-71) was amplified by PCR using the following primers: 5′-CGCGGATCCGTTGAATTACAACCACAATC-3′ and 5′-ATAGTTTAGCGGCCGCTT ATTTAGCTACAATATTG-3′. The amplified *leuRS* was digested with BamHI and NotI and ligated into pETDuet-1 vector (Novagen) which carries an N-terminal His tag. After sequencing, the pETDuet1-leuRS plasmid was transformed into *E. coli* BL21(DE3)pLys cells, which were then grown overnight in 20 mL of Luria–Bertani (LB) medium containing 100 μg mL^−1^ ampicillin and 30 μg mL^−1^ chloramphenicol. Then the overnight culture was inoculated into 400 mL of Super Broth medium containing ampicillin and chloramphenicol at the same concentration, and the cells were grown until the OD600 reached 0.6. Isopropyl β-d-1-thiogalactopyranoside (IPTG) (1 mM) was added to induce protein expression, and the culture was shaken overnight at 20 °C. Cells were harvested by centrifugation at 4000 rpm for 10 min at 4 °C. The cell pellet was resuspended in 30 mL of His-binding buffer (50 mM HEPES, 150 mM NaCl, 5 mM imidazole, pH 7.9), and the bacteria were disrupted by sonication. Cell debris was removed by centrifugation at 20 000*g* for 20 min at 4 °C. The cleared lysate was loaded onto the HisTrap HP 5 ml column mounted on a AKTA Purifier10 FPLC system (GE Healthcare). *S. aureus* MRSA LeuRS was eluted with imidazole gradient in extraction buffer (50–500 mM). Fractions containing *S. aureus* MRSA LeuRS were dialyzed overnight against 25 mM HEPES (pH 8.0), 150 mM NaCl, 5 mM 2ME, 0–20% glycerol and stored at −20 °C. The protein concentration in the final solution was 2.86 mg mL^−1^ (determined by the Bradford method and bovine serum albumin as a standard).^78^

#### Enzyme inhibition assay

The aminoacylation reaction was performed in a 25 μL reaction volume with 7.5 nM *S. aureus* MRSA LeuRS, 2 mg mL^−1^*E. coli* total tRNA (Roche), 40 μM ^14^C-Leu (120 μCi mmol^−1^) in 50 mM HEPES-KOH buffer (pH 8.0) containing 30 mM MgCl_2_, 30 mM KCl, 5 mM 2ME, 0.2 U mL^−1^ pyrophosphatase and 0.05% BSA. Unless stated otherwise, the test compound (1 μL, DMSO solution), *S. aureus* MRSA LeuRS, and *E. coli* total tRNA were pre-incubated for 10 min at 37 °C before the reaction was initiated with 2 mM ATP. At specific times, a 10 μL aliquot was spotted on 3MM Whatmann paper soaked for 10 min in 10% (w/v) cold trichloroacetic acid (TCA). All papers washed three times with 10% (w/v) TCA and once with acetone at 10-min intervals and dried in air. Each paper was transferred into a vial and soaked into 6 mL scintillation liquid. Subsequent measurements were performed using a scintillation counter (Canberra-Packard). Each experiment was performed in duplicate.

#### Complex generation

To generate a homology model of the *S. aureus* MRSA leucyl-tRNA synthetase [NCBI sequence code NZ_LFVO01000005.1],^79,80^ the *T. thermopilus* leucyl-tRNA synthetase [PDB code: 2V0C]^81^ was used as a template. The PDB and the modelled sequence were aligned in MOE.^66^ Due to high homology, positions in the PDB where the sequence diverged were mutated to *S. aureus* residues and subsequently minimized. Importantly, as known inhibitors of LeuRS work by forming a stable adduct with tRNA in the enzyme editing site,^10,61–65^ the compound was analyzed in a nucleotide adduct form (here AMP; to mimic the terminus of a tRNA). The AMP adduct of 20 was then docked into the structure. 100 initial poses were generated using the triangle matcher method, and then refined through a quick energy minimization (while maintaining a rigid protein) and rescored using the GBVI/WSA dG function. The pose selection process was guided with known crystallographic data of similar compounds – in detail we selected only poses in which the AMP adduct retained binding characteristics observed in other complexes (*i.e.*, PDB code: 7BZJ).

#### Molecular dynamics simulations

For MD simulation, the complex was protonated using MOE,^66^ solvated (using TIP3P model water) and neutralized with NaCl ions (0.15 M concentration) using the CHARMM-GUI webserver.^82^ Simulations were carried out using Gromacs5.1.4.^83^ Systems were subjected to 1000 cycles of energy minimization, followed by a 10 ns equilibration with positional restraints applied to backbone (400 kJ mol^−1^ nm^−2^) and sidechain (40 kJ mol^−1^ nm^−2^) atoms. After equilibration, we carried out unrestrained MD simulations (5 × 1 μs). For all simulations, we utilized a 2 fs timestep. H-bonds were restrained using the LINCS algorithm.

The pressure and temperature of the system were maintained at 1 bar and 303 K, respectively using the Nose-Hoover thermostat and the Parrinello–Rahman barostat. For non-bonding interactions, we applied a cutoff at 12 Å using the Verlet scheme, and a force switch at 10 Å for vdW interactions. Electrostatic interactions were treated using PME. System parameters were obtained from the CHARMM36m forcefield.^84^ Parameters of the ligand were extracted from the CGenFF forcefield^85,86^ using Paramchem.^87–89^ As the simulated ligand contained a silicon atom, for which parameters are not available in the CGenFF forcefield, it was replaced with a carbon atom (which holds similar chemical properties). Simulations were analyzed using VMD.^90^

## Author contributions

S. L. and A. E. L.: conceptualization of the paper and supervision of the research; S. L.: funding acquisition, S. L. and A. E. L.: design of the experiments; K. N., A. K., and J. Ko.: synthesis and compound characterization, J. Kr.: antimicrobial activity studies; M. W. enzyme inhibition assay; P. W.: cytotoxicity assay; P. H. M.-U., K. D. and K. W.: single-crystal X-ray diffraction; K. N., T. M. S. and J. S.: bioinformatic analysis; K. N., A. E. L., and S. L.: analysis of all data; K. N., J. Kr., T. M. S., A. E. L. and S. L.: writing the original draft. All authors have read and agreed to the published version of the manuscript.

## Conflicts of interest

There are no conflicts to declare.

## Supplementary Material

MD-015-D4MD00061G-s001

MD-015-D4MD00061G-s002
