# Supplementary material for: Exploiting thiol-functionalized benzosiloxaboroles for achieving diverse substitution patterns – synthesis, characterization and biological evaluation of promising antibacterial agents
Source: RSC Med Chem. 2024 Mar 20;15(5):1751–72. doi: 10.1039/d4md00061g (PMC11110727; doi:10.1039/d4md00061g)
Supplement: MD-015-D4MD00061G-s001 [file MD-015-D4MD00061G-s001.pdf]

Electronic Supplementary Material (ESI) for RSC Medicinal Chemistry.
This journal is © The Royal Society of Chemistry 2024

Exploiting thiol-functionalized benzosiloxaboroles for achieving diverse substitution patterns – synthesis, characterization and biological evaluation of promising antibacterial agents

Krzysztof Nowicki,[a,*] Joanna Krajewska,[b] Tomasz M. Stępniewski,[c] Monika Wielechowska,[a] Patrycja Wińska,[a] Anna Kaczmarczyk,[a] Julia Korpowska,[a] Jana Selent,[c] Paulina H. Marek-Urban,[a] Krzysztof Durka,[a] Krzysztof Woźniak,[d] Agnieszka E. Laudy,[b,*] Sergiusz Luliński[a,*]

[a]*Faculty of Chemistry, Warsaw University of Technology, Noakowskiego 3, 00-664 Warsaw, Poland*

[b]*Department of Pharmaceutical Microbiology and Bioanalysis, Medical University of Warsaw, Banacha 1b, 02-097, Warsaw, Poland*

[c]*GPCR Drug Discovery Lab, Research Programme on Biomedical Informatics (GRIB), Hospital del Mar Medical Research Institute (IMIM) - Department of Medicine and Life Sciences, Pompeu Fabra University (UPF), Carrer del Dr. Aiguader, 88, 08003 Barcelona, Spain*

[d]*Faculty of Chemistry, University of Warsaw, Pasteura 1, 00-093 Warsaw, Poland*

*Corresponding authors. Email addresses: krzysztof.nowicki2.dokt@pw.edu.pl (K. Nowicki), alaudy@wp.pl (A. E. Laudy), sergiusz.lulinski@pw.edu.pl (S. Luliński).

Supporting Information

List of contents

1. Synthesis……………………………………………………………………………….......................S2
2. Antimicrobial activity………………………………………………………………….……….….…S6
3. Cytotoxicity……………………………………………………………………………………..S8
4. Purification of MRSA LeuRS ………………………………………………………...……...… .S10
5. Single-crystal X-ray diffraction ……………………………………….…………...……....…S11
6. References…..……………………………………………………………..…………..……...…..S21
7. NMR spectra……………………………………………..……………….……….......…S22

1. Synthesis

Compounds **1b_CH2** and **1b_D** were isolated as byproducts in the synthesis of **1b** (**Scheme S1**). The extensive formation of **1b_CH2** is intriguing but not fully clear. It was rationalized by the reaction of **1b_D** with lithium enolate formed from LDA-induced cleavage of THF according to the mechanism proposed for a similar transformation[1] (**Scheme S2**).

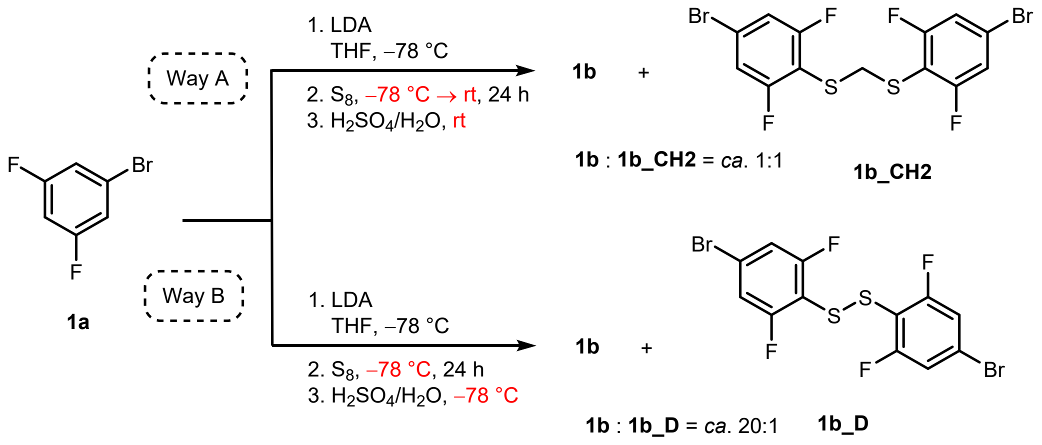

Scheme S1. The first stage of the synthesis of the precursor **1b** carried out under different conditions.

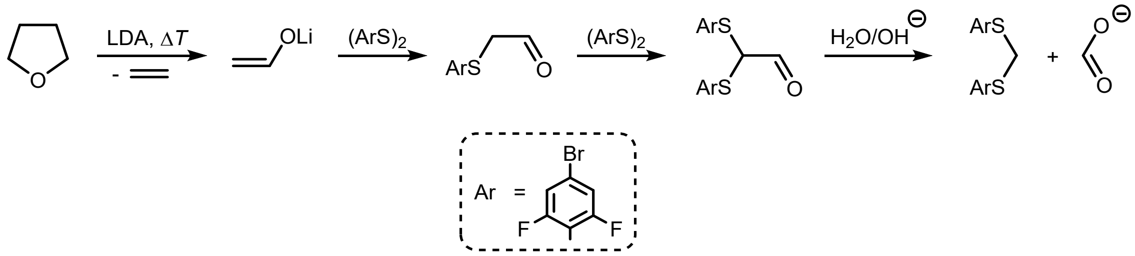

Scheme S2. The plausible mechanism of the formation of **1b_CH2** initiated by THF ring cleavage under the influence of LDA at increased temperature.

Succinimidyl acrylate (**S1**) and functionalized acrylamides (**S2–S4**) were obtained as described in the literature,[2–5] *i.e.*, by *O*- or *N*-acylation of respective starting materials with acryloyl chloride in the presence of Et_3N (**Scheme S3**).

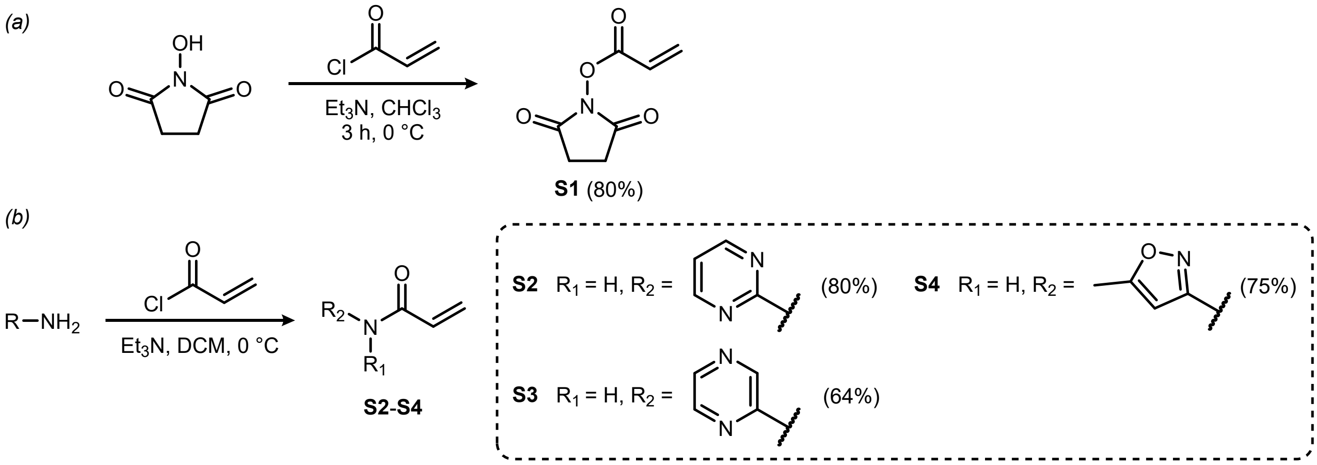

Scheme S3. Synthesis of Michael acceptors (**S1–S4**) used in reaction leading to benzosiloxaboroles: **5** (a) and **9–11** (b).

N-Aryl maleimides were obtained utilizing two-step protocol described in the literature,[6,7] *i.e.*, by the reaction of maleic anhydride with appropriate arylamines followed by the treatment of resultant intermediate by sodium acetate in acetic anhydride (**Scheme S4**).

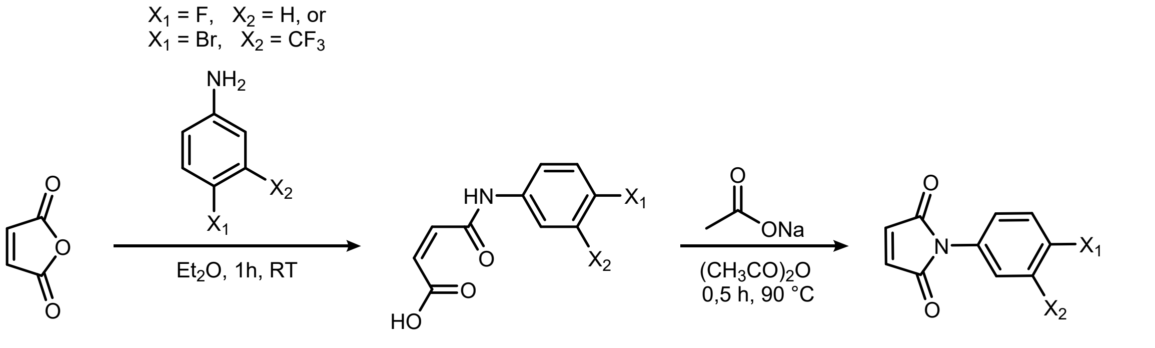

S5: X_1 = F, X_2 = H, (93%)
S7: X_1 = Br, X_2 = CF_3 (58%)

S6: X_1 = F, X_2 = H (57%)
S8: X_1 = Br, X_2 = CF_3 (58%)

Scheme S4. Synthesis of Michael acceptors **S6** and **S8** used in reactions leading to benzosiloxaboroles **14–17**.

Compound **S9** was synthesized in two-step protocol. The first step involved the reaction between 6-bromohexan-1-ol and pyridine as described in literature[8]. Resultant *N*-(6-hydroxyhex-1-yl)pyridinium bromide was then subjected to ion exchange with KOTf in order to give the final *N*-(6-hydroxyhex-1-yl)pyridinium triflate (**Scheme S5**).

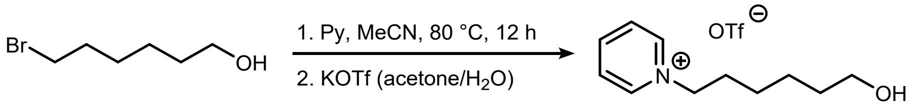

S9 (84%)

Scheme S5. Synthesis of compound **S9** used in reactions leading to benzosiloxaboroles **18** and **19**.

Synthesis of α-bromoketones was achieved through bromination of acetone and acetophenone with bromine and NBS, respectively, in accordance with the protocols already reported[9,10] (**Scheme S6**).

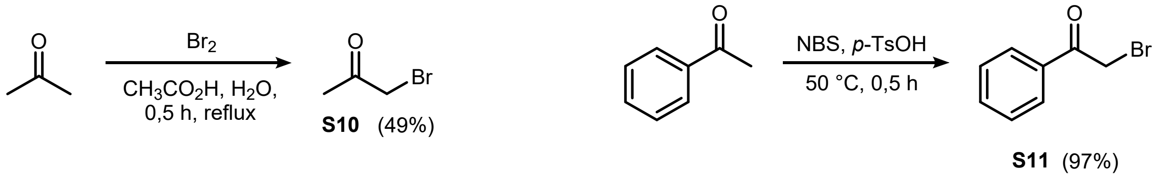

S10 (49%)

S11 (97%)

Scheme S6. Synthesis of α-bromoketones, *i.e.*, bromoacetone (**S10**) and 2-bromoacetphenone (**S11**) used as electrophiles in reactions leading to benzosiloxaboroles **22** and **23**, respectively.

Bis((4-bromo-2,6-difluorophenyl)thio)methane (1b_CH2). The compound was isolated through crystallization with hexane, as a byproduct during the synthesis of **1b** conducted in the initial variant. ^{1}H NMR (400 MHz, $CDCl_3$) δ 7.11–7.05 (m, 4H), 4.27 (s, 2H) ppm. ^{13}C NMR (101 MHz, $CDCl_3$) δ 164.3 – 161.4 (m), 123.8–122.5 (m), 116.5–114.9 (m), 108.6 (t, *J* = 22.4 Hz), 38.3 (p, *J* = 3.2 Hz) ppm. ^{19}F NMR (376 MHz, $CDCl_3$) δ -102.64 to –102.78 (m) ppm.

1,2-Bis(4-bromo-2,6-difluorophenyl)disulfane (1b_D). The compound was isolated as a residue after distillation during the synthesis of **1b** conducted in the final variant. ^{1}H NMR (400 MHz, $CDCl_3$) δ 7.18–7.11

(m, 4H) ppm. ^{13}C NMR (101 MHz, $CDCl_3$) δ 165.8–160.1 (m), 125.1 (t, *J* = 12.4 Hz), 116.0 (m), 112.4 (t, *J* = 23.1 Hz) ppm. ^{19}F NMR (376 MHz, $CDCl_3$) δ –102.11 (d, *J* = 5.6 Hz) ppm.

Succinimidyl acrylate (S1).[2] ^{1}H NMR (400 MHz, $CDCl_3$) δ 6.70 (dd, *J* = 17.3, 0.9 Hz, 1H), 6.32 (dd, *J* = 17.3, 10.7 Hz, 1H), 6.16 (dd, *J* = 10.7, 0.9 Hz, 1H), 2.86 (s, 4H) ppm.

***N*-(Pyrimidin-2-yl)acrylamide (S2)**.[3] ^{1}H NMR (400 MHz, $CDCl_3$) δ 9.41 (s, 1H), 8.65 (d, *J* = 4.8 Hz, 2H), 7.02 (t, *J* = 4.9 Hz, 1H), 6.93 (dd, *J* = 17.0, 10.3 Hz, 1H), 6.54 (dd, *J* = 17.0, 1.5 Hz, 1H), 5.85 (dd, *J* = 10.3, 1.5 Hz, 1H) ppm.

***N*-(Pyrazin-2-yl)acrylamide (S3)**.[4] ^{1}H NMR (400 MHz, DMSO-d_6) δ 11.01 (s, 1H), 9.43 (d, *J* = 1.6 Hz, 1H), 8.42 (dd, *J* = 2.6, 1.5 Hz, 1H), 8.38 (d, *J* = 2.5 Hz, 1H), 6.62 (dd, *J* = 17.1, 10.1 Hz, 1H), 6.36 (dd, *J* = 17.0, 1.8 Hz, 1H), 5.85 (dd, *J* = 10.1, 1.8 Hz, 1H) ppm.

***N*-(5-Methylisoxazol-3-yl)acrylamide (S4)**.[5] ^{1}H NMR (400 MHz, DMSO-d_6) δ 11.11 (s, 1H), 6.70 (s, 1H), 6.45 (dd, *J* = 17.0, 10.1 Hz, 1H), 6.31 (dd, *J* = 17.1, 2.0 Hz, 1H), 5.82 (dd, *J* = 10.1, 2.0 Hz, 1H), 2.38 (d, *J* = 0.9 Hz, 3H) ppm.

4-((4-Fluorophenyl)amino)-4-oxobut-2-enoic acid (S5). The synthesis was performed according to the previously described protocol[6,7] using 4-fluoroaniline. The product was obtained as a yellow solid. Yield 3.91 g (93%) ^{1}H NMR (400 MHz, DMSO-d_6) δ 13.05 (s, 1H), 10.41 (s, 1H), 7.67–7.56 (m, 2H), 7.22–7.08 (m, 3H), 6.44 (d, *J* = 12.0 Hz, 1H), 6.28 (d, *J* = 12.0 Hz, 1H) ppm. ^{13}C NMR (101 MHz, DMSO-d_6) δ 167.3, 163.6, 158.8 (d, *J* = 240.7 Hz), 135.4 (d, *J* = 2.6 Hz), 132.2, 130.7, 121.7 (d, *J* = 7.9 Hz), 115.9 (d, *J* = 22.3 Hz) ppm. ^{19}F NMR (376 MHz, DMSO-d_6) δ –118.52 (tt, *J* = 8.9, 4.9 Hz) ppm.

1-(4-Fluorophenyl)-1H-pyrrole-2,5-dione (S6). The synthesis was performed according to the previously described protocol[6,7]. The product was obtained as a yellow solid. Yield 2.06 g (57%). ^{1}H NMR (400 MHz, $CDCl_3$) δ 7.37–7.28 (m, 2H), 7.21–7.09 (m, 2H), 6.85 (d, *J* = 1.8 Hz, 2H) ppm. ^{13}C NMR (101 MHz, $CDCl_3$) δ 169.4, 161.8 (d, *J* = 247.9 Hz), 134.2, 127.9 (d, *J* = 8.5 Hz), 127.1 (d, *J* = 3.1 Hz), 116.2 (d, *J* = 22.9 Hz). ^{19}F NMR (376 MHz, DMSO-d_6) δ –113.92 (tt, *J* = 8.8, 5.3 Hz).

4-((4-Bromo-3-(trifluoromethyl)phenyl)amino)-4-oxobut-2-enoic acid (S7). The synthesis was performed according to the previously described protocol[6,7] using 4-bromo-3-(trifluoromethyl)aniline. The product was obtained as a cream-colored solid. Yield 3.95 g (58%). ^{1}H NMR (400 MHz, Acetone-d_6) δ 13.17–11.80 (m, 1H), 10.24 (s, 1H), 7.74 (d, *J* = 2.5 Hz, 1H), 7.36 (d, *J* = 8.7 Hz, 1H), 7.30 (dd, *J* = 8.8, 2.5 Hz, 1H), 6.01 (d, *J* = 12.0 Hz, 1H), 5.88 (d, *J* = 12.0 Hz, 1H) ppm. ^{13}C NMR (101 MHz, Acetone-d_6) δ 166.9, 163.8, 138.7, 135.6, 131.4, 130.3, 128.6 (q, *J* = 30.6 Hz), 124.1, 122.8 (q, *J* = 273.3 Hz), 118.2 (q, *J* = 5.8 Hz), 111.9 (d, *J* = 2.1 Hz) ppm. ^{19}F NMR (376 MHz, DMSO-d_6) δ –61.78 ppm.

1-(4-Bromo-3-(trifluoromethyl)phenyl)-1H-pyrrole-2,5-dione (S8). The synthesis was performed according to the previously described protocol[6,7]. The product was obtained as a cream-colored solid. Yield 1.97 g (58%). ^{1}H NMR (400 MHz, $CDCl_3$) δ 7.79 (d, *J* = 8.6 Hz, 1H), 7.76 (d, *J* = 2.5 Hz, 1H), 7.49–7.43 (m, 1H), 6.89 (d, *J* = 0.6 Hz, 2H) ppm. ^{13}C NMR (101 MHz, $CDCl_3$) δ 168.6, 135.7, 134.5, 131.0 (q, *J* = 31.9 Hz), 129.7 (d, *J* = 1.2 Hz), 125.8 (q, *J* = 5.5 Hz), 125.0 (d, *J* = 5.6 Hz), 126.6–118.0 (m), 118.8 (q, *J* = 1.9 Hz) ppm. ^{19}F NMR (376 MHz, $CDCl_3$) δ –62.97 ppm.

***N*-(6-Hydroxyhex-1-yl)pyridinium triflate (S9).** A mixture of 6-bromohexan-1-ol (6.0 g, 33.0 mmol) and pyridine (5.4 mL, 66.0 mmol) in MeCN (50 mL) was stirred at 80 °C for 12 h. The solvent was removed under reduced pressure and the obtained solid was washed with Et_2O and dried *in vacuo* to give *N*-(6-hydroxyhex-1-yl)pyridinium bromide[8] as a white solid. Yield 7.10 g (86%). The bromide salt (6.50 g, 25.0 mmol) was subjected to ion exchange using KOTf (4.70 g, 25.0 mmol) in acetone/H_2O. The organic phase was separated and the solvent was removed under reduced pressure to leave the product as a colorless viscous liquid which dried under high vacuum prior to use in a subsequent acylation step. Yield 8.06 g (98%) ^{1}H NMR (400 MHz, acetone-d_6) δ 9.24–9.18 (m, 2H), 8.73 (tt, J = 7.8, 1.4 Hz, 1H), 8.26 (t, J = 7.0 Hz, 2H), 4.86–4.82 (m, 2H), 3.79 (broad, 1H), 3.50 (t, J = 6.2 Hz, 2H), 2.15–2.10 (m, 2H), 1.53–1.40 (m, 6H) ppm. ^{13}C NMR (101 MHz, acetone-d_6) δ 146.60, 145.78, 129.32, 122.13 (q, J = 321.5 Hz), 62.65, 61.97, 33.16, 32.05, 26.40, 25.94 ppm. ^{19}F NMR (376 MHz, acetone-d_6) δ –78.90 ppm.

Bromoacetone (S10).[9] ^{1}H NMR (400 MHz, $CDCl_3$) δ 3.88 (t, J = 0.5 Hz, 2H), 2.37 (d, J = 0.4 Hz, 3H) ppm.

2-Bromoacetophenone (S11).[10] ^{1}H NMR (400 MHz, $CDCl_3$) δ 8.02–7.96 (m, 2H), 7.65–7.58 (m, 1H), 7.53–7.46 (m, 2H), 4.46 (d, J = 0.3 Hz, 2H) ppm.

2. Antimicrobial activity

Table S1. The antibacterial activity of tested agents against standard Gram-negative strains.

agent	MIC in μg·mL^{-1} [MBC in μg·mL^{-1}][a] / ×-fold reduction of MIC in the presence of PAßN[b] (diameter of inhibition zone in mm)										
	E. coli ATCC 25922	*K. pneumoniae* ATCC 13883	*P. mirabilis* ATCC 12453	*E. cloacae* DSM 6234	*S. marcescens* ATCC 13880	*A. baumannii* ATCC 19606	*P. aeruginosa* ATCC 27853	*S. maltophilia* ATCC 13637	*S. maltophilia* ATCC 12714	*B. cepacia* ATCC 25416[c]	*B. bronchiseptica* ATCC 4617[c]
**1g**	>400 (-)	>400 (-)	>400 (-)	>400 (-)	>400 (-)	>400 (-)	>400 (-)	>400 (-)	>400 (-)	>400 (-)	>400 (-)
**2e**[e]	>100 (-)	>100 (-)	>100 (-)	>100 (-)	>100 (-)	>100 (-)	>100 (-)	>100 (-)	>100 (-)	>100 (-)	>100 (-)
**3**	>400 (-)	>400 (-)	>400 (-)	>400 (-)	>400 (-)	>400 (-)	>400 (-)	400 (-)	200 (-)	>400 (-)	50 (-)
**4**	400/**8** (15)	>400/**8** (-)	>400 (-)	>400/**8** (-)	>400 (-)	>400/2 (-)	>400 (-)	>400/2 (-)	>400/2 (-)	>400 (-)	>400 (-)
**5**	>400 (-)	>400 (-)	>400 (-)	>400 (-)	>400 (-)	>400 (-)	>400 (-)	>400 (-)	>400 (-)	>400 (-)	>400 (-)
**6**	>400/**2** (-)	>400 (-)	>400 (-)	>400 (-)	>400 (-)	>400 (-)	>400 (-)	>400/2 (-)	>400 (-)	>400 (-)	>400 (-)
**7**	>400/**4** (-)	>400 (-)	>400 (-)	>400 (-)	>400 (-)	>400 (-)	>400 (-)	>400 (-)	>400 (-)	>400 (-)	>400 (-)
**8**	>400 (-)	>400 (-)	>400 (-)	>400 (-)	>400 (-)	>400 (-)	>400 (-)	>400 (-)	>400 (-)	>400 (-)	>400 (-)
**9**	>400 (-)	>400 (-)	>400 (-)	>400 (-)	>400 (-)	>400 (-)	>400 (-)	>400 (-)	>400 (-)	>400 (-)	>400 (-)
**10**	>400 (-)	>400 (-)	>400 (-)	>400 (-)	>400 (-)	>400 (-)	>400 (-)	>400 (-)	>400 (-)	>400 (-)	>400 (-)
**11**	>400 (-)	>400 (-)	>400 (-)	>400 (-)	>400 (-)	>400 (-)	>400 (-)	>400 (-)	>400 (-)	>400 (-)	>400 (-)
**12**	50 [200] (17)	400/**4** (17)	>400/**4** (14)	>400/**8** (-)	200/2 (28)	100 [200]/2 (18)	>400 (-)	50 [100] (13)	200/2 (13)	>400 (-)	12.5 [50] (25)
**13**	25 [50] (19)	200/**4** (19)	400/**4** (18)	400/**4** (-)	100/2 (29)	50 [100] (20)	200 [400] (-)	25 [50] (19)	50 [200] (17)	>400 (-)	12.5 [25] (26)
**14**[d]	>200/2 (-)	>200 (-)	>200 (-)	>200 (-)	>200 (-)	>200 (-)	>200 (-)	25 [100] (11)	100 (-)	>200 (-)	200 (11)
**15**[d]	>200 (-)	>200 (-)	>200 (-)	>200 (-)	>200 (-)	>200 (-)	>200 (-)	>200 (-)	>200 (-)	>200 (-)	>200 (-)
**16**[d]	>200 (-)	>200 (-)	>200 (-)	>200 (-)	>200 (-)	>200 (-)	>200 (-)	>200 (-)	>200 (-)	>200 (-)	200 (-)
**17**	>200 (-)	>200 (-)	>200 (-)	>200 (-)	>200 (-)	>200 (-)	>200 (-)	>200 (-)	>200 (-)	>200 (-)	200 (-)
**18**	>400 (-)	>400 (-)	>400 (-)	>400 (-)	>400 (-)	>400 (-)	>400 (-)	>400 (-)	>400 (-)	>400 (-)	>400 (-)
**19**	>400 (-)	>400 (-)	>400 (-)	>400 (-)	>400 (-)	>400 (-)	>400 (-)	>400 (-)	>400 (-)	>400 (-)	>400 (-)
**20**	>400/**32** (-)	>400/**4** (-)	>400 (-)	>400/**4** (-)	>400 (-)	>400 (-)	>400 (-)	>400 ( )	>400 (-)	>400 (-)	>400 (-)
**21**	>400/**16** (11)	>400 (-)	>400 (-)	>400 (-)	>400 (-)	>400 (-)	>400 (-)	>400 (-)	>400 (-)	>400 (-)	>400 (-)
**22**	>400 (-)	>400 (-)	>400 (-)	>400 (-)	>400 (-)	>400 (-)	>400 (-)	>400 (-)	>400 (-)	>400 (-)	>400 (-)
**23**	>400 (-)	>400 (-)	>400 (-)	>400 (-)	>400 (-)	>400 (-)	>400 (-)	>400 (-)	>400 (-)	>400 (-)	>400 (-)
**24**	>400 (-)	>400 (-)	>400 (-)	>400 (-)	>400 (-)	>400 (-)	>400 (-)	>400 (-)	>400 (-)	>400 (-)	>400 (-)
**25**[f]	>50 (-)	>50 (-)	>50 (-)	>50 (-)	>50 (-)	>50 (-)	>50 (-)	>50 (-)	>50 (-)	>50 (-)	>50 (-)
**26**	>400 (-)	>400 (-)	>400 (-)	>400 (-)	>400 (-)	>400 (-)	>400 (-)	>400 (-)	>400 (-)	>400 (-)	>400 (-)
**27**	>400/2 (-)	>400 (-)	>400 (-)	>400 (-)	>400 (-)	>400 (-)	>400 (-)	>400 (-)	>400 (-)	>400 (-)	>400 (-)
**28**	>400/**4** (-)	>400 (-)	>400 (-)	>400 (-)	>400 (-)	>400 (-)	>400 (-)	>400 (-)	>400 (-)	>400 (-)	>400 (-)
**29**	>400/2 (-)	>400 (-)	>400 (-)	>400 (-)	>400 (-)	>400 (-)	>400 (-)	>400 (-)	>400 (-)	>400 (-)	>400 (-)
NF[g]	8 [8] (24)	32 [32] (23)	128 [>128] (9)	32 [32] (17)	128 [>128] (12)	64 [128] (9)	>128 [>128] (-)	128 [>128] (-)	128 [>128] (-)	32 [32] (12)	64 [128] (-)

PAβN: efflux pump inhibitor. The significant decreases (at least a 4-fold) in the MIC values of tested compounds after the addition of PAβN are shown in boldface. The test was performed in the MHB medium supplemented with 1 mM $MgSO_4$.

(-): The inhibition zone was not observed in the disc-diffusion method. The diameter of the paper discs was 9 mm.

[a] Only the MBC values ≤400 μg·mL^{-1} are presented.

[b] In the table, only at least 2-fold decreases in the MIC values of tested compounds after the addition of PAβN are presented.

[c] The growth of *B. cepacia* ATCC 25416 and *B. bronchiseptica* ATCC 4617 strains was inhibited in the MHB medium supplemented with 1 mM $MgSO_4$ and 20 μg·mL^{-1} PAβN.

[d] The MIC and MBC values of the substance were determined up to 200 μg·mL^{-1}. In the table, only the MBC values ≤ 200 μg·mL^{-1} are presented. The tested substance dissolved in DMSO precipitated after implementation into the MHB medium at a concentration above 200 μg·mL^{-1}.

[e] The MIC and MBC values of the substance were determined up to 100 μg·mL^{-1}. In the table, only the MBC values ≤100 μg·mL^{-1} are presented. The tested substance dissolved in DMSO precipitated after implementation into the MHB medium at a concentration above 100 μg·mL^{-1}.

[f] The MIC and MBC values of the substance were determined up to 50 μg·mL^{-1}. In the table, only the MBC values ≤ 50 μg·mL^{-1} are presented. The tested substance dissolved in DMSO precipitated after implementation into the MHB medium at a concentration above 50 μg·mL^{-1}.

[g] NF, nitrofurantoin was used as a reference agent active against Gram-negative bacteria. The diameter of a commercial disc containing 0.3 mg of nitrofurantoin was 6 mm; the MIC of nitrofurantoin was determined according to the CLSI recommendations.[11]

Table S2. The antifungal activity of tested agents against yeasts strains.

	MIC in μg·mL^{-1} [MFC in μg·mL^{-1}][a] (Diameter of inhibition zone in mm)						
agent	*C. albicans* ATCC 90028	*C. krusei* ATCC 6258	*C. parapsilosis* ATCC 22019	*C. tropicalis* ATCC 750	*C. tropicalis* IBA 171	*C. guilliermondii* IBA 155	*S. cerevisiae* ATCC 9763
**1e**	100 (15)	400 (13)	400 (-)	400 (-)	200 (-)	>400 (-)	200 (17)
**2e**[c]	>100 (-)	>100 (-)	>100 (-)	>100 (-)	>100 (-)	>100 (-)	>100 (-)
**3**	>400 (-)	200 (-)	100 (-)	>400 (-)	400 (-)	200 (-)	400 (-)
**4**	>400 (12)	200 (12)	400 (-)	>400 (-)	>400 (-)	400 (12)	**12.5** [400] (32)
**5**	>400 (-)	>400 (-)	>400 (-)	>400 (-)	>400 (-)	>400 (-)	>400 (-)
**6**	>400 (-)	>400 (-)	>400 (-)	>400 (-)	>400 (-)	>400 (-)	>400 (-)
**7**	>400 (-)	>400 (-)	>400 (-)	>400 (-)	>400 (-)	>400 (-)	>400 (-)
**8**	>400 (-)	>400 (-)	>400 (-)	>400 (-)	>400 (-)	>400 (-)	>400 (-)
**9**	>400 (-)	>400 (-)	>400 (-)	>400 (-)	>400 (-)	>400 (-)	>400 (-)
**10**	>400 (-)	>400 (-)	>400 (-)	>400 (-)	>400 (-)	>400 (-)	>400 (-)
**11**	>400 (-)	>400 (-)	>400 (-)	>400 (-)	>400 (-)	>400 (-)	>400 (-)
**12**	>400 (-)	>400 (-)	>400 (-)	>400 (-)	>400 (-)	>400 (-)	>400 (-)
**13**	>400 (-)	>400 (-)	>400 (-)	>400 (-)	>400 (-)	>400 (-)	>400 (-)
**14**[b]	200 (21)	**12.5** [100] (30)	100 (27)	200 (20)	200 (19)	100 (31)	50 (30)
**15**[b]	200 (19)	25 (29)	200 (23)	>200 (14)	>200 (15)	200 (28)	200 (26)
**16**[b]	50 (25)	25 (21)	50 (24)	50 (19)	50 (19)	**12.5** [50] (30)	**6.25** [50] (35)
**17**	100 (20)	100 (18)	50 (22)	100 (14)	200 (16)	50 [200] (27)	**6.25** [100] (33)
**18**	>400 (-)	>400 (-)	400 (14)	400 (-)	400 (-)	>400 (-)	>400 (-)
**19**	>400 (-)	>400 (-)	400 (16)	400 (15)	400 (16)	>400 (-)	>400 (-)
**20**	>400 (-)	>400 (-)	>400 (-)	>400 (-)	>400 (-)	>400 (-)	400 (16)
**21**	400 (-)	>400 (-)	400 (-)	>400 (-)	400 (-)	>400 (-)	**6.25** [100] (30)
**22**	>400 (-)	200 (12)	>400 (-)	>400 (-)	>400 (-)	>400 (-)	400 (17)
**23**	>400 (-)	200 (12)	400 (-)	400 (-)	400 (-)	>400 (-)	25 [200] (24)
**24**	>400 (-)	>400 (-)	>400 (-)	>400 (-)	>400 (-)	>400 (-)	>400 (-)
**25**[d]	50 (-)	>50 (-)	>50 (-)	>50 (-)	50 (-)	50 (-)	25 (-)
**26**	>400 (-)	>400 (-)	>400 (-)	>400 (-)	>400 (-)	>400 (-)	>400 (-)
**26**	>400 (-)	>400 (-)	>400 (-)	>400 (-)	>400 (-)	>400 (-)	>400 (-)
**27**	>400 (-)	>400 (-)	>400 (-)	>400 (-)	>400 (-)	>400 (-)	>400 (-)
**28**	>400 (-)	>400 (-)	>400 (-)	>400 (-)	>400 (-)	>400 (-)	>400 (-)
FL[f]	1 (43)	64[f] (16)	2 (32)	0.38 (40)	0.38 (39)	0.75 (40)	16[g] (12)

The highest activity against yeasts indicated by the low MIC values (≤12.5 μg·mL^{-1}) is shown in boldface.

(-): The inhibition zone was not observed in the disc-diffusion method. The diameter of the paper discs was 9 mm.

[a] Only the MFC values ≤400 μg·mL^{-1} are presented.

[b] The MIC and MFC values of the substance were determined up to 200 μg·mL^{-1}. In the table, only the MFC values ≤200 μg·mL^{-1} are presented. The tested substance dissolved in DMSO precipitated after implementation into the RPMI medium at a concentration above 200 μg·mL^{-1}.

[c] The MIC and MFC values of the substance were determined up to 100 μg·mL^{-1}. In the table, only the MFC values ≤100 μg·mL^{-1} are presented. The tested substance dissolved in DMSO precipitated after implementation into the RPMI medium at a concentration above 100 μg·mL^{-1}.

d The MIC and MFC values of the substance were determined up to 50 μg·mL^{-1}. In the table, only the MFC values ≤50 μg·mL^{-1} are presented. The tested substance dissolved in DMSO precipitated after implementation into the RPMI medium at a concentration above 50 μg·mL^{-1}.

[d] FL, fluconazole was used as a reference antifungal agent; the diameter of a commercial disc containing 0.025 mg of fluconazole was 6 mm; the MIC value of fluconazole was determined by the Etest method.[12]

[e] The ellipse was visible pointing the MIC value 64 μg·mL^{-1}. However, with macro-colonies up to a concentration ≥256 μg·mL^{-1}. In accordance with the recommendations for the Etest method, the MIC value of fluconazole against *C. krusei* can also be interpreted as ≥256 μg·mL^{-1}.[12,13] *C. krusei* is intrinsically resistant to fluconazole.

f The ellipse was visible pointing the MIC value 16 μg·mL^{-1}, with colonies up to concentration ≥256 μg·mL^{-1}. There are no recommendations for the Etest method interpretation of the MIC value of fluconazole against *S. cerevisiae*. The obtained MIC 16 μg·mL^{-1} is in line with the published results.[14]

3. Cytotoxicity

Table S3. The viability of human normal lung fibroblasts, MRC-5 after 48 h treatment with the tested compounds.

	viability of MRC-5 [% of control ± SD]													
compound concentration [μg·mL^{-1}]	**6**	**12**	**14**	**15**	**16**	**17**	**20**	**22**	**23**	**24**	**25**	**27**	**28**	**29**	LIN[a]
400	29.5±5.8	0.4±0.2	0.1±0.1	6.7±0.1	0.1±0.0	0.0±0.0	3.4±0.9	41.4±0.4	1.0±1.3	60.5±3.8	1.4±0.2	66.2±2.4	15.6±0.1	60.4±5.2	85.7±0.2
200	43.6±5.1	46.9±2.7	27.3±3.2	41.5±0.7	0.4±0.2	29.5±0.3	38.8±4.8	69.6±3.7	32.0±0.3	76.5±3.8	0.9±0.2	68.5±3.4	32.5±0.0	69.6±2.4	85.7±3.2
100	90.4±0.5	76.4±3.5	52.0±2.8	65.0±0.1	46.0±1.4	71.2±1.3	68.5±4.9	78.4±4.5	64.2±3.6	73.0±4.0	57.3±2.9	74.3±7.9	47.7±3.7	74.1±0.5	83.9±0.5
50	108.3±3.0	86.1±6.9	80.3±7.5	69.9±1.8	62.7±2.5	89.2±8.5	84.0±1.5	94.4±2.1	85.9±5.1	78.2±3.0	70.0±4.4	79.7±7.5	65.5±1.8	80.8±3.4	85.7±0.2
25	104.7±6.7	94.9±2.3	87.5±3.5	73.7±0.8	78.5±1.6	87.6±0.2	93.5±2.1	85.2±1.1	83.3±4.4	81.6±2.6	68.9±8.7	79.3±9.5	75.1±5.4	86.8±4.3	86.2±2.6
12.5	112.1±1.8	104.4±1.9	88.8±5.4	77.9±4.3	84.8±2.7	96.1±2.3	95.3±1.0	91.5±2.5	94.3±0.1	83.9±0.1	79.1±3.9	74.1±1.4	82.6±1.2	90.6±4.5	86.0±1.9
6.25	104.0±5.7	103.0±1.5	90.4±7.6	89.2±0.3	97.0±0.2	99.9±4.6	101.2±1.7	92.1±0.1	99.3±8.4	95.5±1.4	86.3±3.2	74.1±3.1	93.3±1.9	90.1±3.4	90.4±1.1

[a]LIN, linezolid was used as a reference agent active against Gram-positive bacteria.

Table S4. The viability of human normal lung fibroblasts, MRC-5 after 48 h treatment with cisplatin.

cisplatin concentration. [μg/mL]	viability [%]
30.0	6.2±1.1
15.0	15.4±1.9
7.50	34.4±1.4
3.75	31.8±0.2
1.87	30.3±0.7
0.94	62.6±0.8
0.47	86.4±0.1

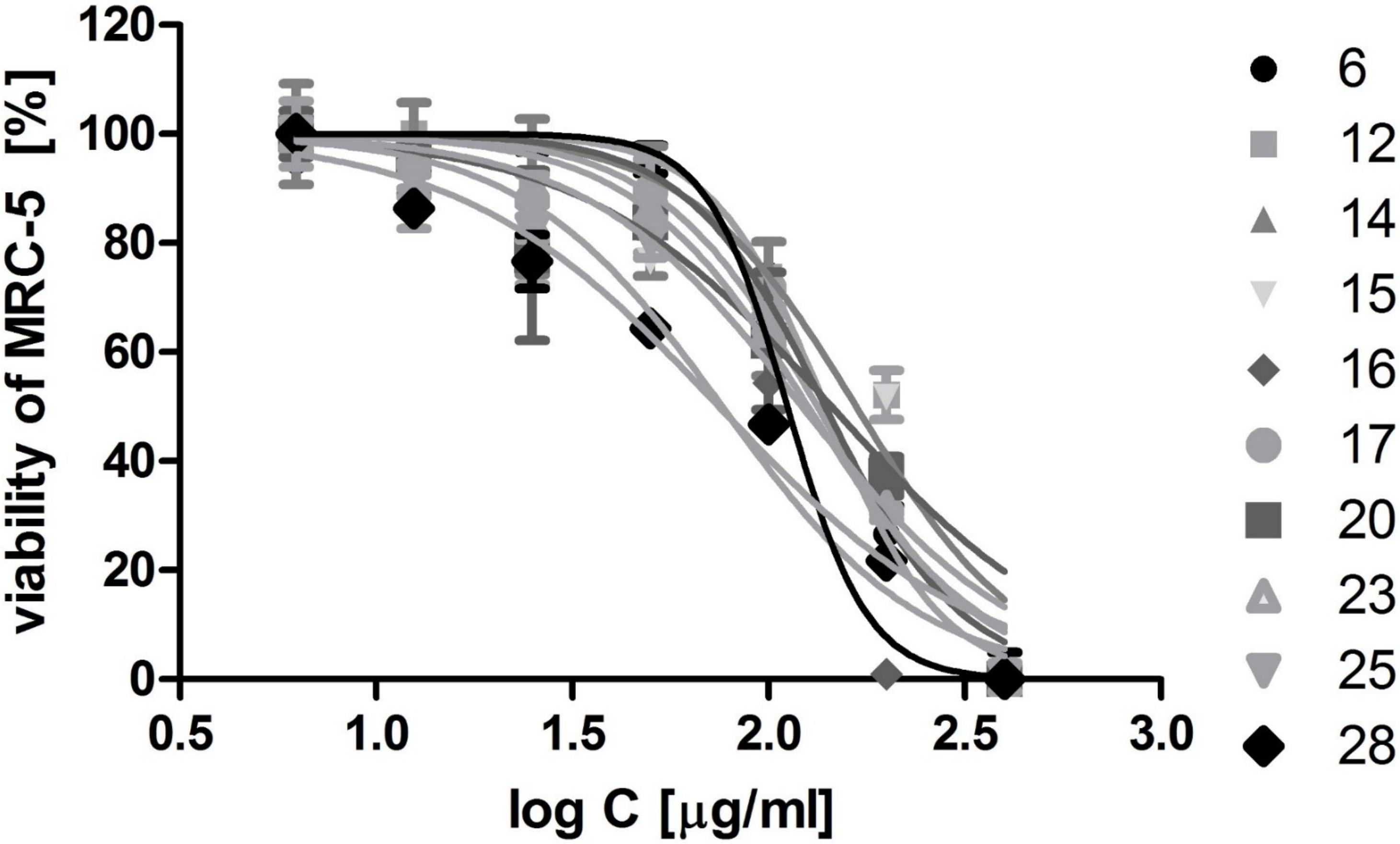

Figure S1. Sigmoidal dose response curves for compounds **6**, **12**, **14**, **15**, **16**, **17**, **20**, **23**, **25** and **28** determined for MRC-5 after fitting the obtained MTT data. Plots were generated using GraphPad Prism program.

4. Purification of MRSA LeuRS

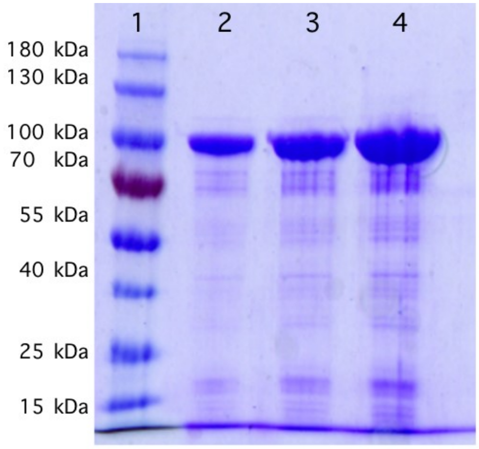

Figure S2. 1-Marker (PageRuler™ Prestained Protein Ladder, 10 to 180 kDa); 2-LeuRS 2.5 μg; 3-LeuRS 5.0 μg, 4- LeuRS 10.0 μg.

5. Single-crystal X-ray diffraction

Single crystals of all studied systems were prepared by slow solvent evaporation at room temperature from corresponding concentrated $CHCl_3$ solutions. Obtained crystals were measured on SuperNova diffractometer equipped with Atlas detector (Cu-K_α radiation, $\lambda = 1.54184$ Å or Mo-K_α radiation, $\lambda = 0.71073$ Å). In all the cases a selected crystal was maintained at low temperature ($T = 100$ K) with the use of Oxford Cryosystems nitrogen gas-flow device. The crystal structures were established in a conventional way *via* X-ray data refinement employing the Independent Atom Model (IAM). Data reduction and analysis were carried out with the *CrysAlisPro* suites of programs.[15] All structures were solved by direct methods using *SHELXS-97*[16] and refined using *SHELXL-2016*.[17] The refinement was based on F^2 for all reflections except those with highly negative values of F^2. Weighted R factors (wR) and all goodness-of-fit ($GooF$) values are based on F^2. Conventional R factors are based on F with F set to zero for negative F^2. The $F_o^2 > 2\sigma(F_o^2)$ criterion was used only for calculating R factors and is not relevant to the choice of reflections for the refinement. All non-hydrogen atoms were refined anisotropically. All carbon-bound hydrogen atoms were placed in calculated positions. The positions of O–H hydrogen atoms were derived from difference electron density maps. The O–H distances were fixed to 0.87 Å with standard deviation of 0.01 Å. Structure **6** was refined as 2-component twin. The methylene groups were found to be disordered over two positions related by the symmetry mirror. In contrast to other structures, structure **12** crystalizes as water solvate. Crystals **14** and **15** are isostructural. All-important crystallographic data including measurement, reduction, structure solution and refinement details are included in **Tables S5–S8** or in the associated CIF files. Deposition numbers 2190658 (for **2e**), 2190659 (for **1b_D**), 2190660 (for **1b_CH2**), 2190661 (for **6**), 2190662 (for **11**), 2190663 (for **12**), 2190664 (for **14**), 2190665 (for **15**), 2190666 (for **20**), 2296881 (for **22**), 2190667 (for **24**), 2258836 (for **26**), 2190668 (for **29**), 2190669 (for **27**), contain the supplementary crystallographic data for this paper.

Table S5. Selected crystal data, data collection and refinement parameters for **2e**, **1b_CH2**, **1b_D** and **6**.

	**2e**	**1b_CH2**	**1b_D**	**6**
Chemical formula	$C_8H_{10}BFO_2SSi$	$C_{13}H_6Br_2F_4S_2$	$C_{12}H_4Br_2F_4S_2$	$C_{11}H_{12}BF_2NO_2SSi$
M_r	228.12	462.10	448.09	299.18
Crystal system, space group	Orthorhombic, *Pbca*	Orthorhombic, *Pccn*	Orthorhombic, $P2_12_12_1$	Monoclinic, *Pm*
T / K	100	100	100	100
a, b, c / Å	11.0854 (2), 12.6784 (2), 15.9392 (3)	14.4495 (2), 7.5081 (1), 13.6340 (2)	7.2662 (1), 10.2301 (1), 18.6721 (3)	8.7169 (3), 7.4368 (3), 10.3692 (3)
α, β, γ / °	90, 90, 90	90, 90, 90	90, 90, 90	90, 103.038 (3), 90
V / Å^3	2240.18 (7)	1479.13 (4)	1387.97 (3)	654.86 (4)
Z	8	4	4	2
Radiation type	Cu $K\alpha$	Cu $K\alpha$	Cu $K\alpha$	Cu $K\alpha$
μ / mm^{-1}	3.50	9.93	10.55	5.37
Crystal size / mm	0.23 × 0.16 × 0.08	0.12 × 0.11 × 0.07	0.20 × 0.11 × 0.08	0.22 × 0.17 × 0.08
T_{min}, T_{max}	0.612, 1.000	0.751, 1.000	0.692, 1.000	0.697, 1.000
No. of measured, independent and observed [$I > 2\sigma(I)$] reflections	10110, 2331, 2177	15232, 1501, 1486	15762, 2781, 2767	1720, 1720, 1623
R_{int}	0.021	0.021	0.022	0.042
$(\sin \theta/\lambda)_{max}$ / Å^{-1}	0.631	0.623	0.623	0.619
$R[F^2 > 2\sigma(F^2)]$, $wR(F^2)$, S	0.030, 0.091, 1.08	0.021, 0.054, 1.11	0.013, 0.032, 1.12	0.047, 0.120, 1.08
No. of reflections	2331	1501	2781	1720
No. of parameters	127	96	181	234
No. of restraints	0	0	0	148
Largest diff. peak/hole / e·Å^{-3}	0.37, -0.29	0.65, -0.72	0.20, -0.38	1.76, -0.88

Table S6. Selected crystal data, data collection and refinement parameters for **11**, **12**, **14** and **15**.

	**11**	**12**	**14**	**15**
Chemical formula	$C_{15}H_{17}BF_2N_2O_4SSi$	$C_{12}H_{12}BF_2NO_4SSi \cdot H_2O$	$C_{18}H_{15}BF_3NO_4SSi$	$C_{18}H_{16}BF_2NO_4SSi$
M_r	398.26	361.20	437.27	419.28
Crystal system, space group	Triclinic, *P*-1	Monoclinic, *C*2/*c*	Monoclinic, $P2_1/n$	Monoclinic, $P2_1/n$
T / K	100	100	100	100
a, b, c / Å	8.6314 (4), 10.5815 (4), 20.2719 (10)	26.9983 (14), 8.3870 (1), 16.1371 (5)	11.9609 (2), 6.7922 (1), 25.2398 (3)	11.9955 (4), 6.7688 (2), 24.8601 (7)
α, β, γ / °	80.173 (4), 80.300 (4), 82.873 (4)	90, 119.051 (5), 90	90, 102.880 (1), 90	90, 102.673 (3), 90
V / Å^3	1789.57 (14)	3194.3 (2)	1998.91 (5)	1969.34 (11)
Z	4	8	4	4
Radiation type	Cu *K*α	Cu *K*α	Cu *K*α	Cu *K*α
μ / mm^{-1}	2.66	2.95	2.50	2.44
Crystal size / mm	0.13 × 0.11 × 0.09	0.20 × 0.12 × 0.09	0.20 × 0.09 × 0.08	0.12 × 0.10 × 0.03
T_{min}, T_{max}	0.866, 1.000	0.712, 1.000	0.850, 1.000	0.915, 1.000
No. of measured, independent and observed [$I > 2\sigma(I)$] reflections	11958, 6540, 5418	14650, 3361, 3215	24573, 3948, 3736	7478, 3812, 2949
R_{int}	0.030	0.021	0.022	0.032
$(\sin \theta/\lambda)_{max}$ / Å^{-1}	0.608	0.631	0.620	0.619
$R[F^2 > 2\sigma(F^2)]$, $wR(F^2)$, S	0.046, 0.124, 1.06	0.028, 0.076, 1.04	0.025, 0.071, 1.04	0.037, 0.095, 1.00
No. of reflections	6540	3361	3948	3812
No. of parameters	481	224	265	256
No. of restraints	4	0	1	1
Largest diff. peak/hole / e·Å^{-3}	0.36, -0.38	0.56, -0.25	0.33, -0.25	0.32, -0.34

Table S7. Selected crystal data, data collection and refinement parameters for **20**, **22**, **24** and **26**.

	**20**	**22**	**24**	**26**
Chemical formula	$C_{16}H_{17}BF_2O_4S_2Si$	$C_{11}H_{14}BFO_3SSi$	$C_{12}H_{18}BF_2O_5PSSi$	$C_8H_8O_4BSiF_2ClS$
M_r	414.31	284.18	382.19	312.55
Crystal system, space group	Triclinic, *P*-1	Triclinic, *P*-1	Monoclinic, $P2_1/n$	Monoclinic, $P2_1/c$
T / K	100	100	100	100
a, b, c / Å	9.4513 (4), 9.8968 (4), 12.2922 (6)	6.8604(4), 9.1623(4), 11.9467(5)	13.0595 (14), 7.3061 (7), 18.839 (3)	12.2732(3), 10.0040(1), 11.9960(3)
α, β, γ / °	91.269 (4), 112.587 (4), 118.044 (4)	101.758(4), 103.715(4), 105.684(4)	90, 91.507 (16), 90	90, 119.008(3), 90
V / Å^3	907.49 (8)	672.94(6)	1796.9 (4)	1288.11(6)
Z	2	2	4	4
Radiation type	Cu $K\alpha$	Cu $K\alpha$	Cu $K\alpha$	Cu *K*a
μ / mm^{-1}	3.66	3.09	3.44	5.328
Crystal size / mm	0.13 × 0.12 × 0.08	0.482 × 0.248 × 0.066	0.14 × 0.07 × 0.06	0.112 × 0.092 × 0.068
T_{min}, T_{max}	0.837, 0.982	0.615, 1.000	0.893, 1.000	0.897, 1.000
No. of measured, independent and observed [$I > 2\sigma(I)$] reflections	12561, 3714, 3564	6224, 2603, 2243	10700, 3683, 3030	13035, 2660, 2660
R_{int}	0.019	0.044	0.049	0.022
$(\sin \theta/\lambda)_{max}$ / Å^{-1}	0.631	0.935	0.631	0.631
$R[F^2 > 2\sigma(F^2)]$, $wR(F^2)$, S	0.027, 0.077, 0.99	0.108, 0.317, 1.451	0.048, 0.135, 1.02	0.0238, 0.0655, 1.075
No. of reflections	3714	2603	3683	2660
No. of parameters	235	163	211	166
No. of restraints	0	0	1	1
Largest diff. peak/hole / e·Å^{-3}	0.43, -0.42	2.711, -0.679	0.48, -0.40	0.37, -0.38

Table S8. Selected crystal data, data collection and refinement parameters for **27** and **28**.

	**27**	**28**
Chemical formula	$C_{14}H_{13}BF_3NO_4SSi$	$C_{12}H_{16}BF_2NO_5SSi$
M_r	387.21	363.22
Crystal system, space group	Triclinic, P-1	Monoclinic, $P2_1/c$
T / K	100	100
a, b, c / Å	6.5260 (5), 10.5500 (5), 13.1827 (7)	10.3865 (4), 16.0059 (5), 10.0154 (4)
α, β, γ / °	101.774 (4), 90.259 (5), 106.776 (6)	90, 113.281 (4), 90
V / Å^3	848.76 (9)	1529.44 (11)
Z	2	4
Radiation type	Mo $K\alpha$	Cu $K\alpha$
μ / mm^{-1}	0.31	3.08
Crystal size / mm	0.14 × 0.06 × 0.02	0.20 × 0.18 × 0.10
T_{min}, T_{max}	0.900, 1.000	0.457, 1.000
No. of measured, independent and observed [$I > 2\sigma(I)$] reflections	6440, 3897, 3022	5846, 2948, 2744
R_{int}	0.025	0.022
(sin θ/λ)$_{max}$ / Å^{-1}	0.684	0.619
$R[F^2 > 2\sigma(F^2)]$, $wR(F^2)$, S	0.045, 0.106, 1.01	0.031, 0.084, 1.07
No. of reflections	3897	2948
No. of parameters	234	208
No. of restraints	2	0
Largest diff. peak/hole / e·Å^{-3}	0.47, -0.40	0.38, -0.40

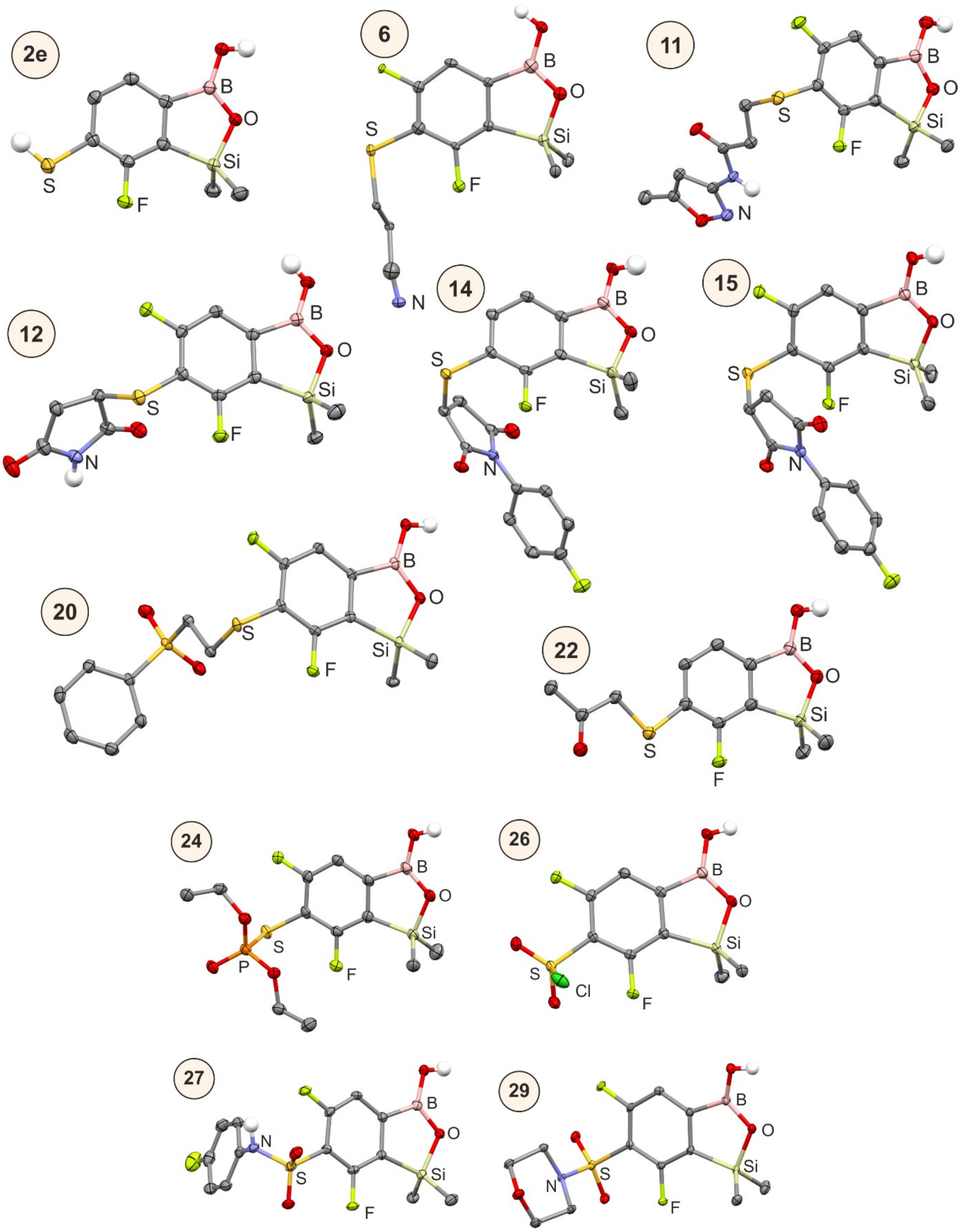

Figure S3. The molecular structures of **2e**, **6**, **11**, **12**, **14**, **15, 20, 22, 24**, **26**, **27** and **29**. Thermal motions given as ADPs at the 50% probability level. C–H hydrogen atoms were omitted for clarity.

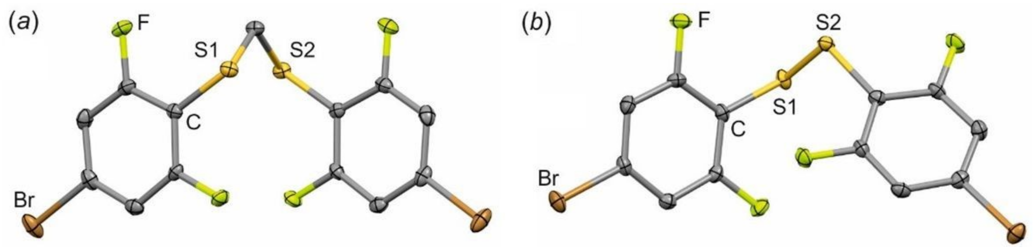

Figure S4. The molecular structures of **1b_CH2** (*a*) and **1b_D** (*b*). Thermal motions given as ADPs at the 50% probability level. C−H hydrogen atoms were omitted for clarity.

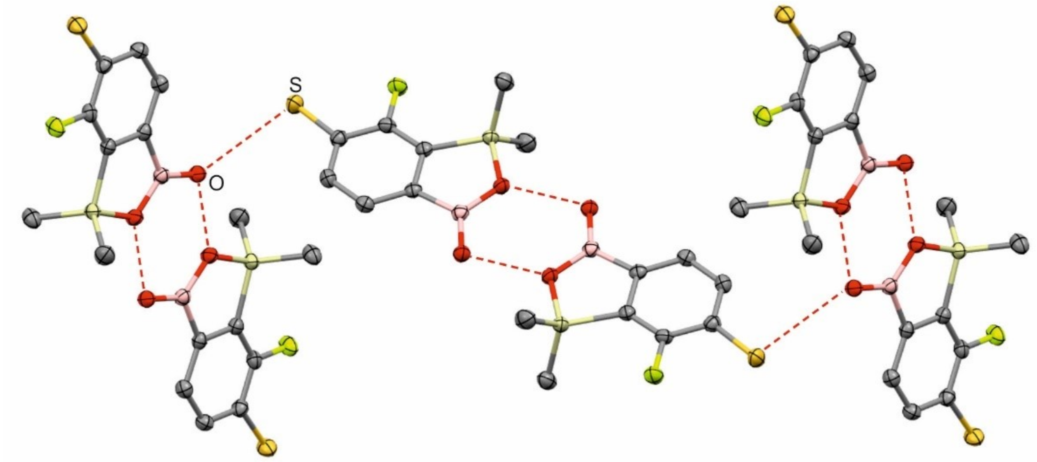

Figure S5. Fragment of supramolecular structures of **2e** showing the formation of molecular chains based on O−H…O and S−H…O. Hydrogen atoms are omitted for clarity.

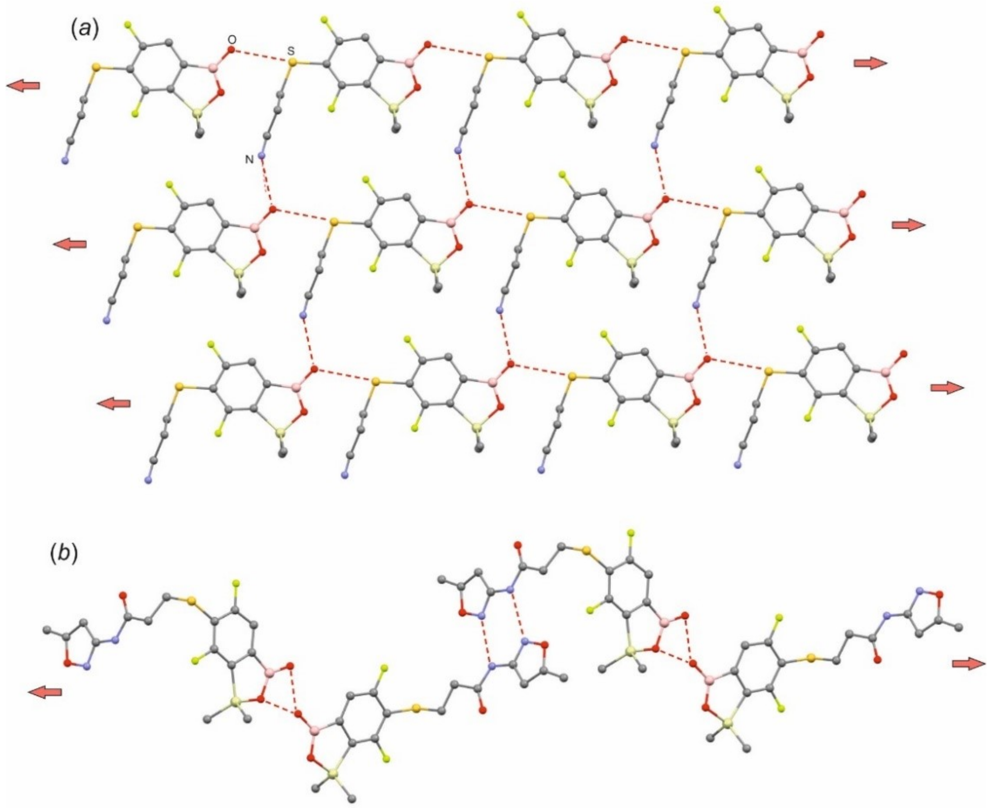

Figure S6. Supramolecular structures of **6** (*a*) and **11** (*b*) showing the formation of hydrogen bond network. Hydrogen atoms are omitted for clarity.

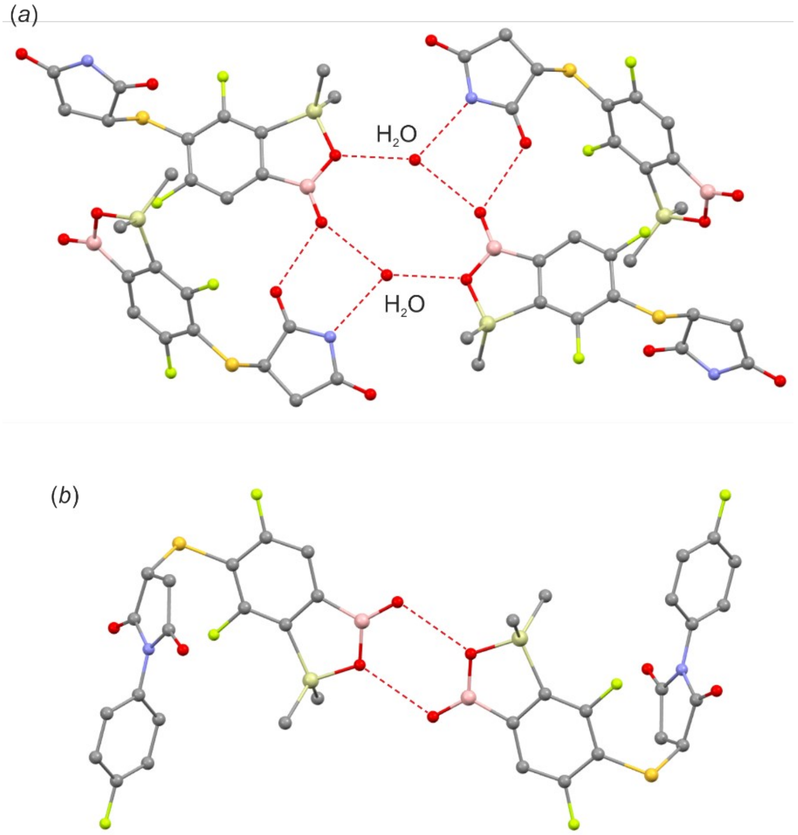

Figure S7. Fragments of supramolecular structures showing the formation of hydrogen bond interactions in **12** (*a*) and **14** (*b*). Hydrogen atoms are omitted for clarity.

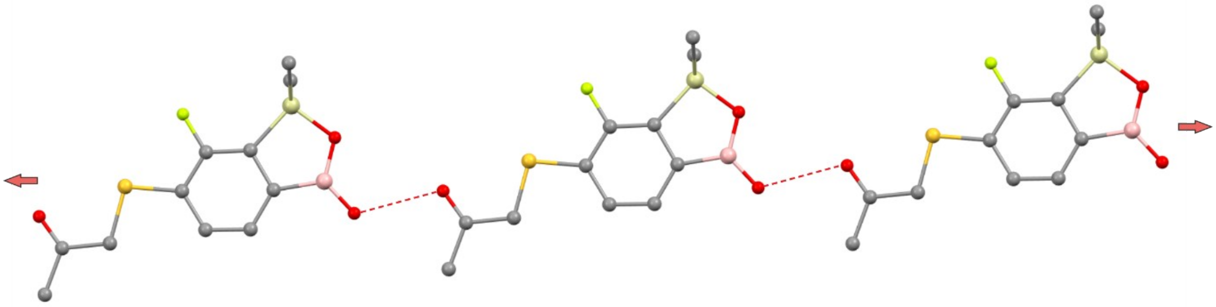

Figure S8. Hydrogen-bonded chains in crystal structure **22**.

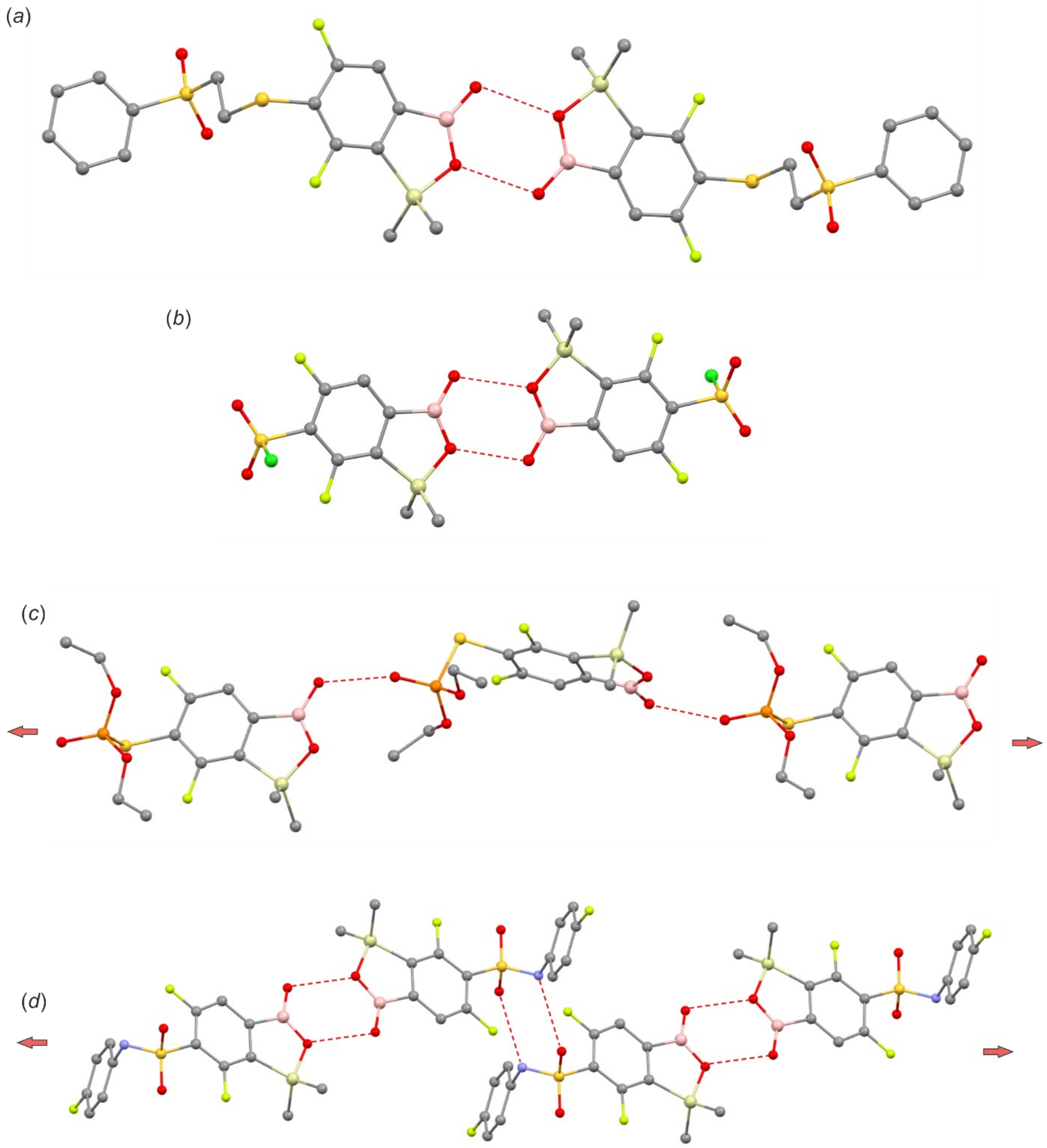

Figure S9. Hydrogen-bonded centrosymmetric dimers formed in crystal structure **22** (*a*) and **24** (*b*). Molecular chains observed in structures **24** (*c*) and **28** (*d*).

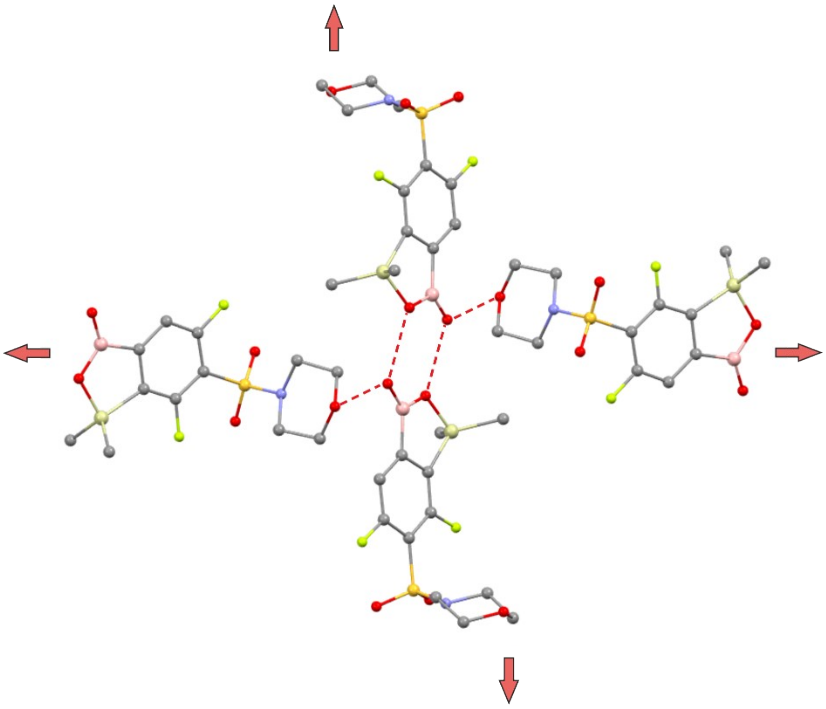

Figure S10. Fragment of supramolecular structures of **29** showing the formation of hydrogen bond network. Hydrogen atoms are omitted for clarity.

6. References

1 Q. Chen, G. Yu, X. Wang, Y. Huang, Y. Yan and Y. Huo, *Org. Biomol. Chem.*, 2018, **16**, 4086–4089.
2 T. J. Paul, A. K. Strzelczyk, M. I. Feldhof and S. Schmidt, *Biomacromolecules*, 2020, **21**, 2913–2921.
3 D. C. Wan, H. T. Pu and G. J. Yang, *Chin. Chem. Lett.*, 2007, **18**, 1141–1144.
4 K. Kokosza, J. Balzarini and D. G. Piotrowska, *Nucleosides, Nucleotides and Nucleic Acids*, 2014, **33**, 552–582.
5 F. A. Al-Sagheer, A. A. M. Ali, M. A. Reyad and M. Z. Elsabee, *Polym. Int.*, 1997, **44**, 88–94.
6 N. E. Searle (to E. I. du Pont de Nemours and Co., Inc.) U.S. pat. 2,444,536 (1948) [C.A., 42, 7340c (1948)].
7 M. P. Cava, A. A. Deana, K. Muth and M. J. Mitchell, in *Org. Synth.*, eds. A. S. Kende and J. P. Freeman, Wiley, 1st edn., 2003, pp. 93–93.
8 J. Park, Y. Choi, S. S. Lee and J. H. Jung, *Org. Lett.*, 2019, **21**, 1232–1236.
9 P. A. Levene, in *Org. Synth.*, eds. A. S. Kende and J. P. Freeman, Wiley, 1st edn., 2003, p. 12.
10 V. V. Angeles-Dunham, D. M. Nickerson, D. M. Ray and A. E. Mattson, *Angew. Chem. Int. Ed.*, 2014, **53**, 14275–14609.
11 Clinical and Laboratory Standards Institute. 2012. Methods for dilution antimicrobial susceptibility tests Clinical and Laboratory Standards Institute.
12 ETEST. Application guide, BioMerieux., https://www.biomerieux-usa.com/sites/subsidiary_us/files/supplementary_inserts_-_16273_-_b_-_en_-_eag_-_etest_application_guide-3.pdf., (accessed 1 December 2023).
13 A. Espinel-Ingroff, *Diagn. Microbiol. Infect. Dis.*, 1994, **19**, 217–220.
14 M. A. Pfaller, M. Bale, B. Buschelman, M. Lancaster, A. Espinel-Ingroff, J. H. Rex and M. G. Rinaldi, *J. Clin. Microbiol.*, 1994, **32**, 1650–1653.
15 Rigaku Oxford Diffraction, Crysalis Pro v. 1.171.38.46, 2018.
16 G. M. Sheldrick, *Acta Crystallogr. A Found. Crystallogr.*, 2008, **64**, 112–122.
17 G. M. Sheldrick, *Acta Crystallogr. C Struct. Chem.*, 2015, **71**, 3–8.

7. NMR spectra

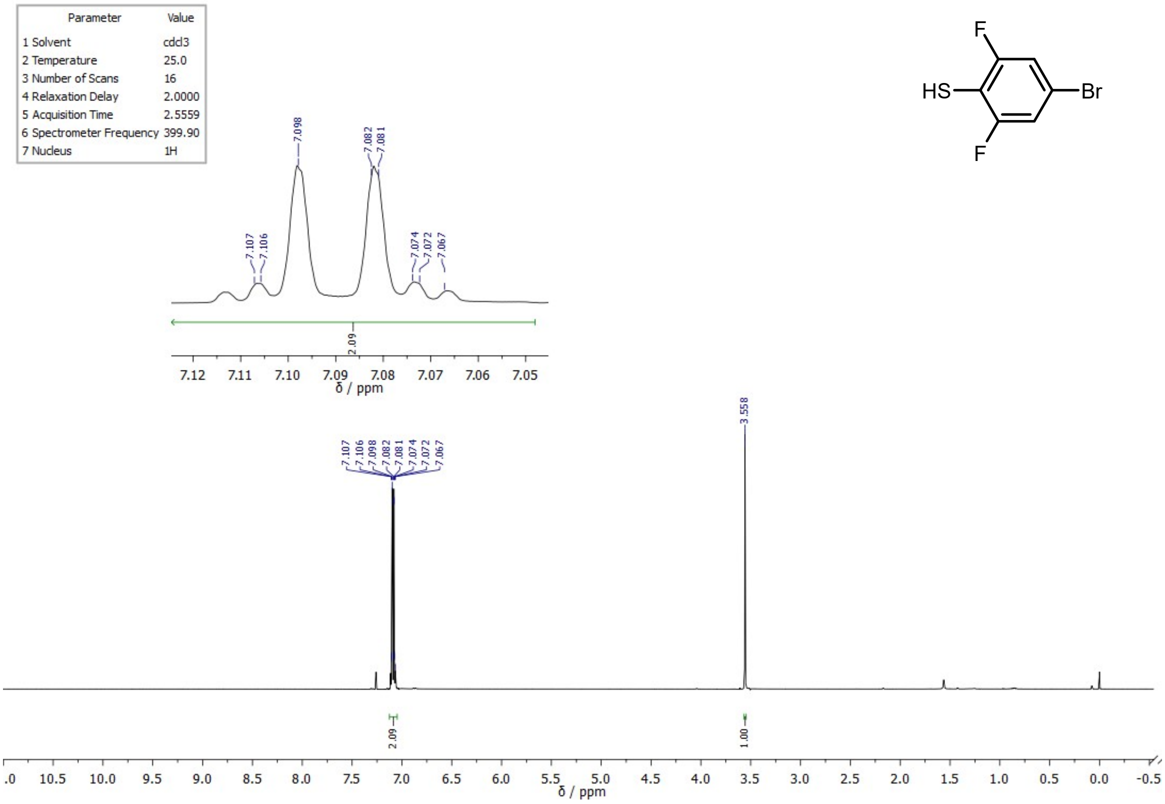

Figure S11. ^{1}H NMR spectrum (400 MHz, $CDCl_3$) of **1b**.

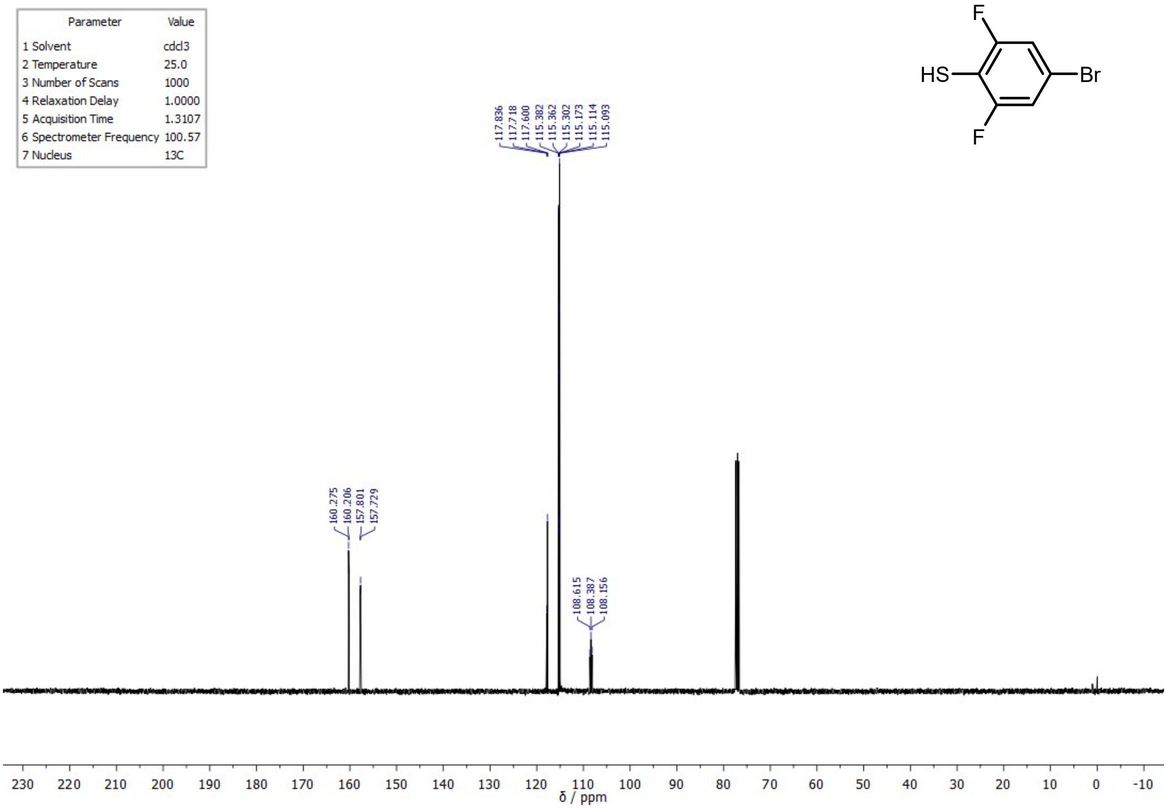

Figure S12. ^{13}C NMR spectrum (101 MHz, $CDCl_3$) of **1b**.

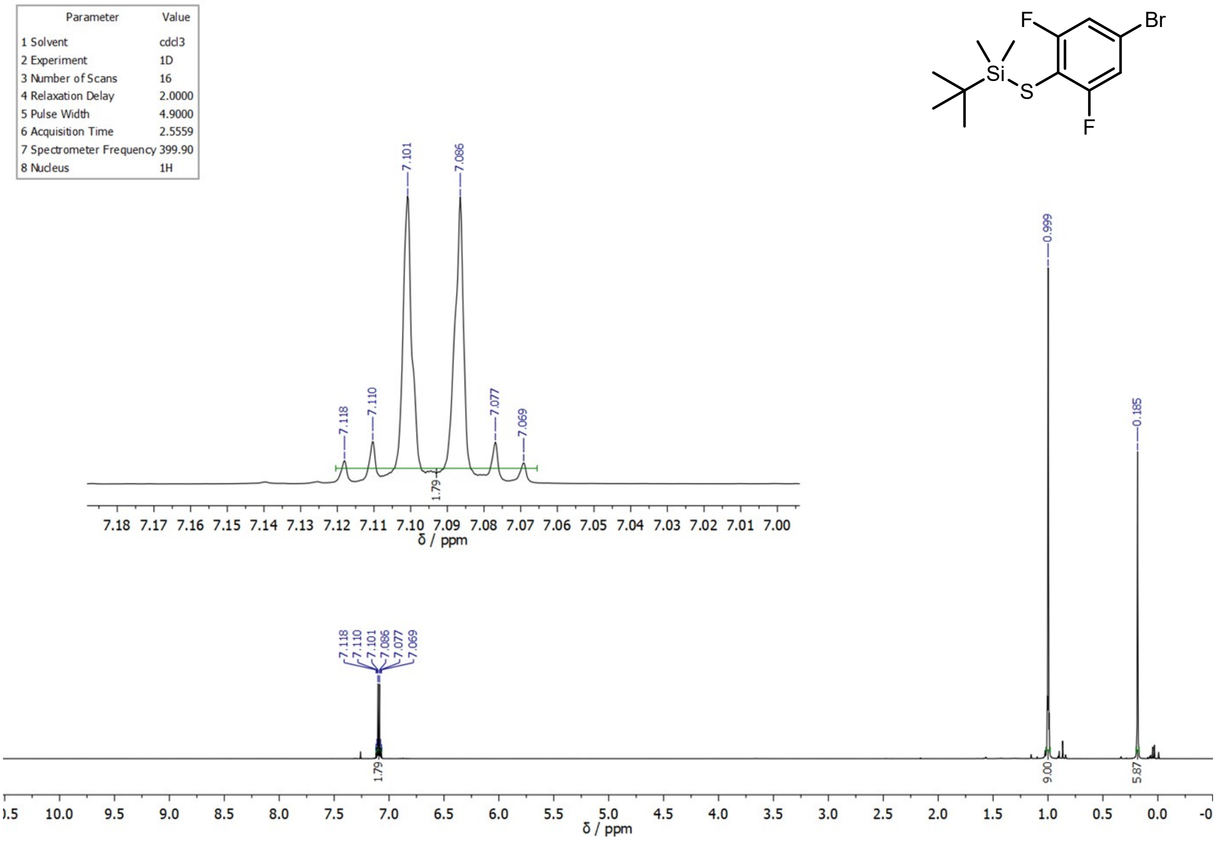

Figure S13. ^{1}H NMR spectrum (400 MHz, $CDCl_3$) of **1c**.

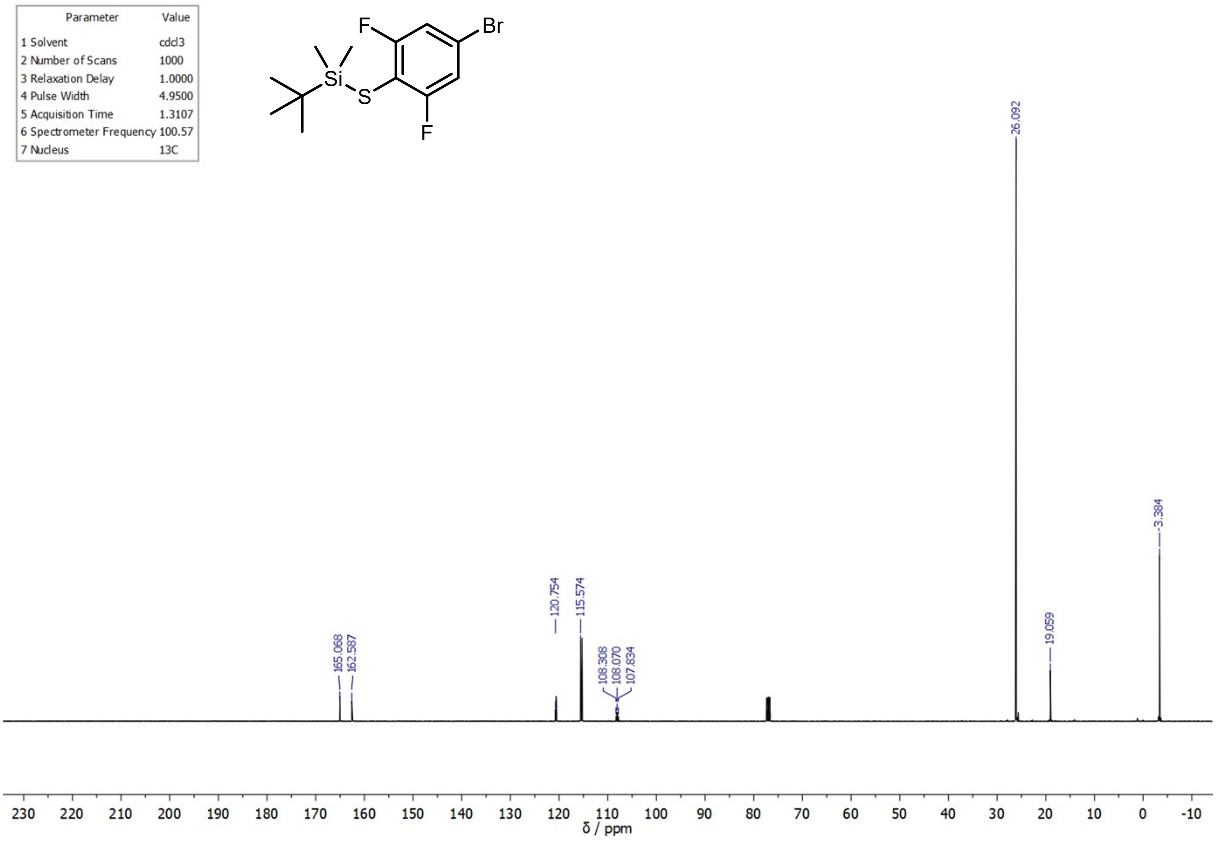

Figure S14. ^{13}C NMR spectrum (101 MHz, $CDCl_3$) of **1c**.

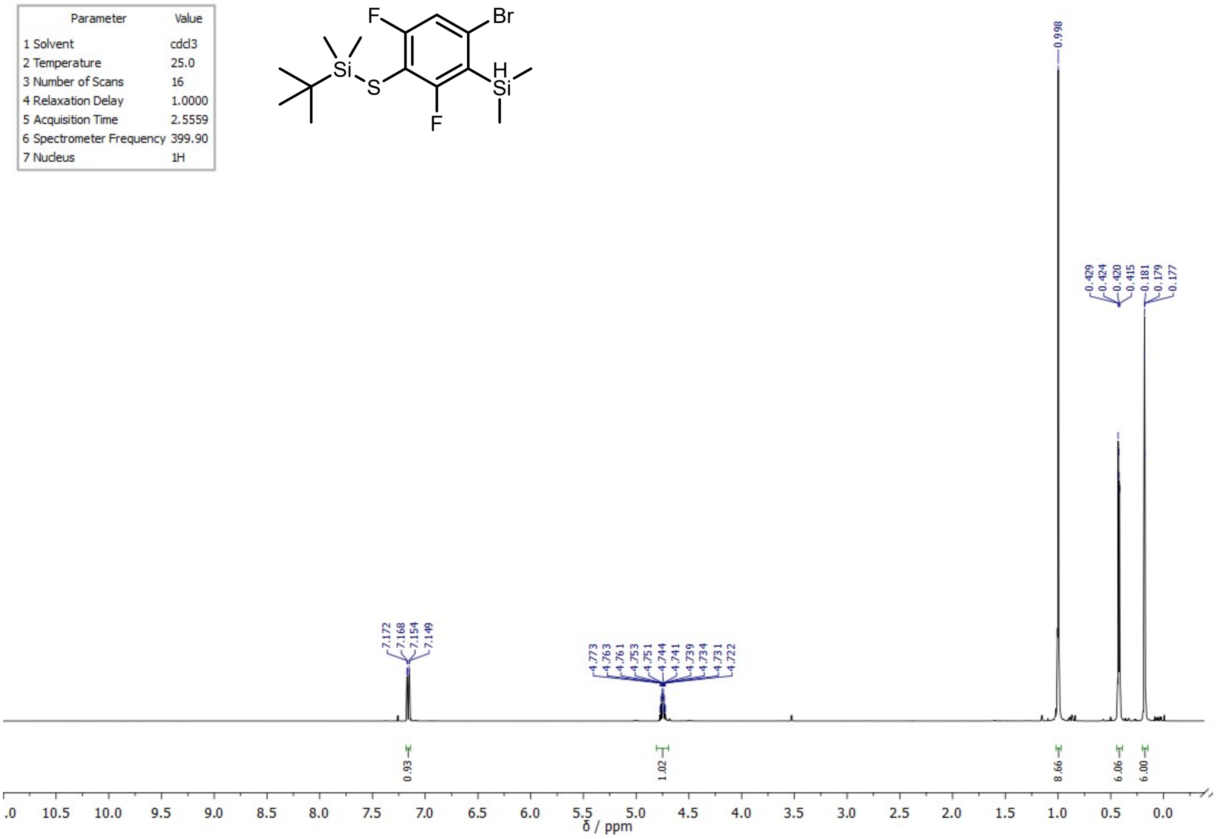

Figure S15. ^{1}H NMR spectrum (400 MHz, $CDCl_3$) of **1d**.

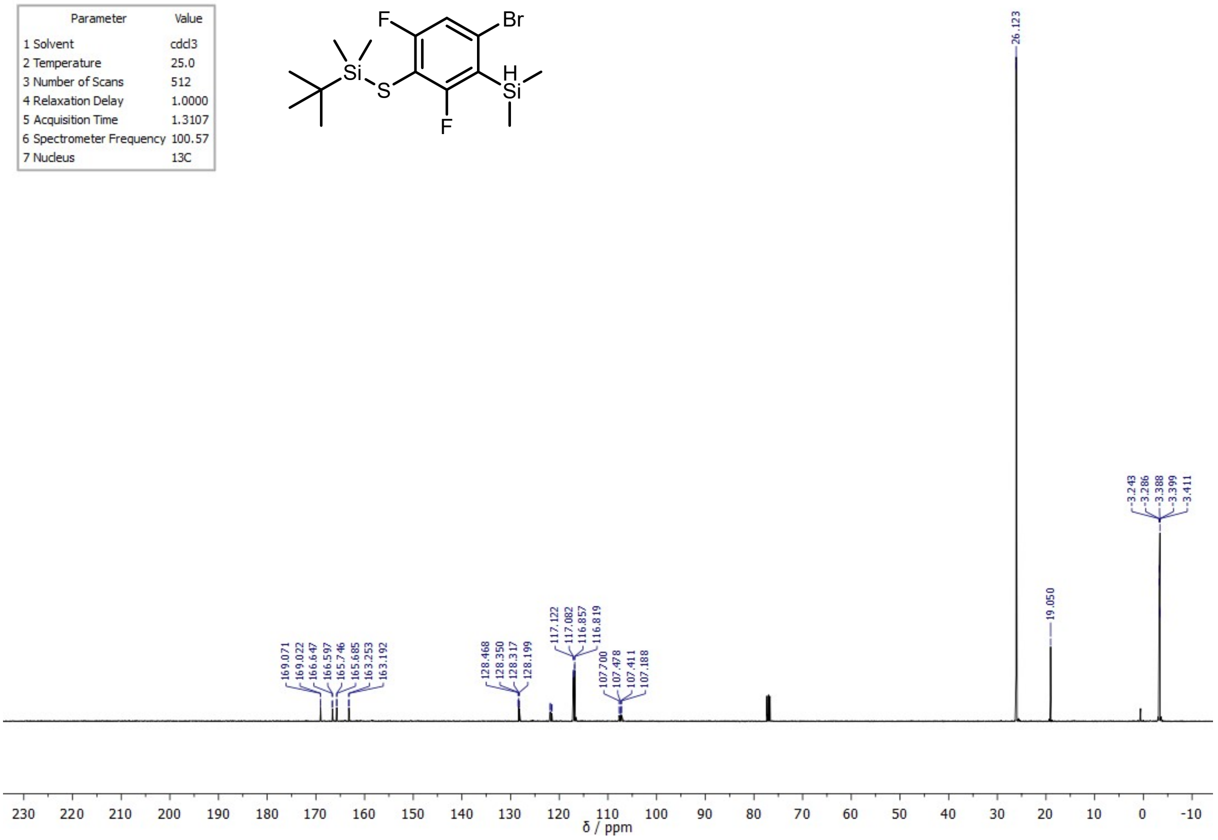

Figure S16. ^{13}C NMR spectrum (101 MHz, $CDCl_3$) of **1d**.

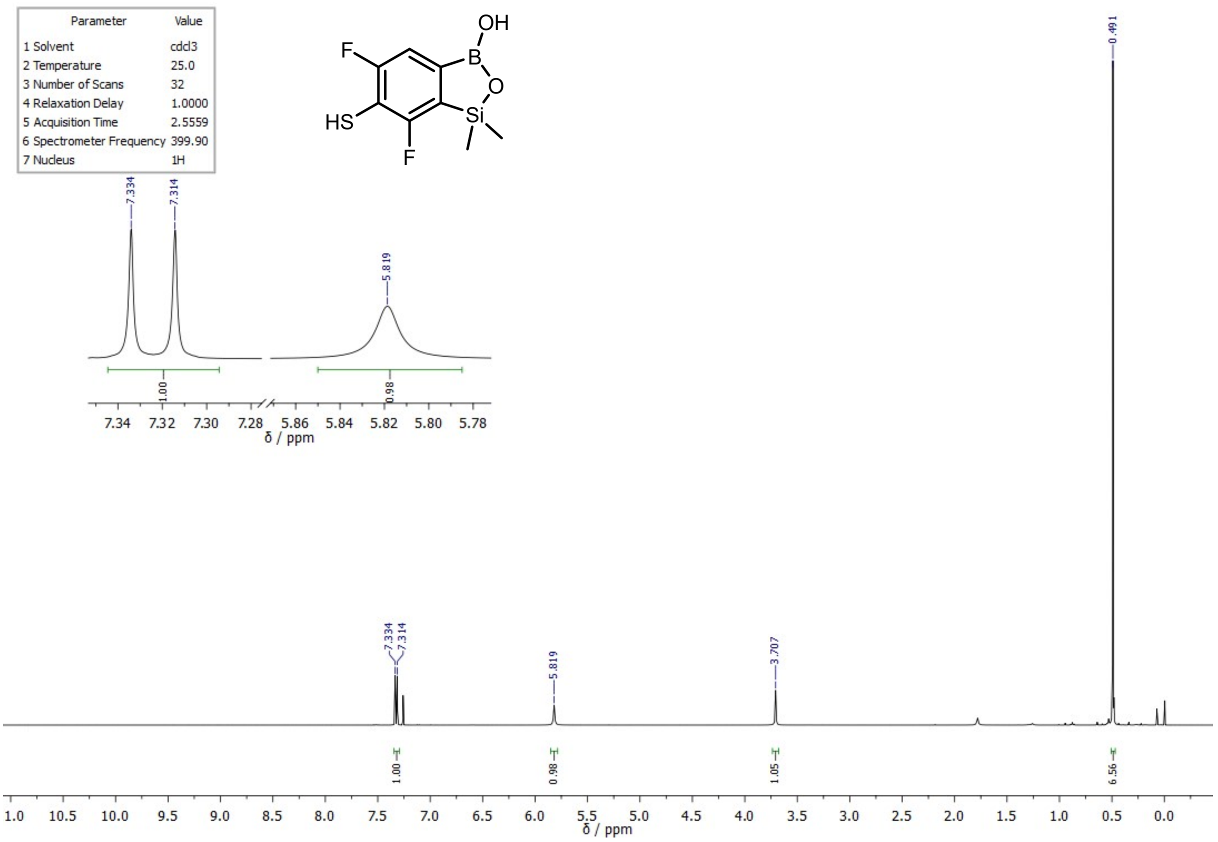

Figure S17. ^{1}H NMR spectrum (400 MHz, $CDCl_3$) of **1e**.

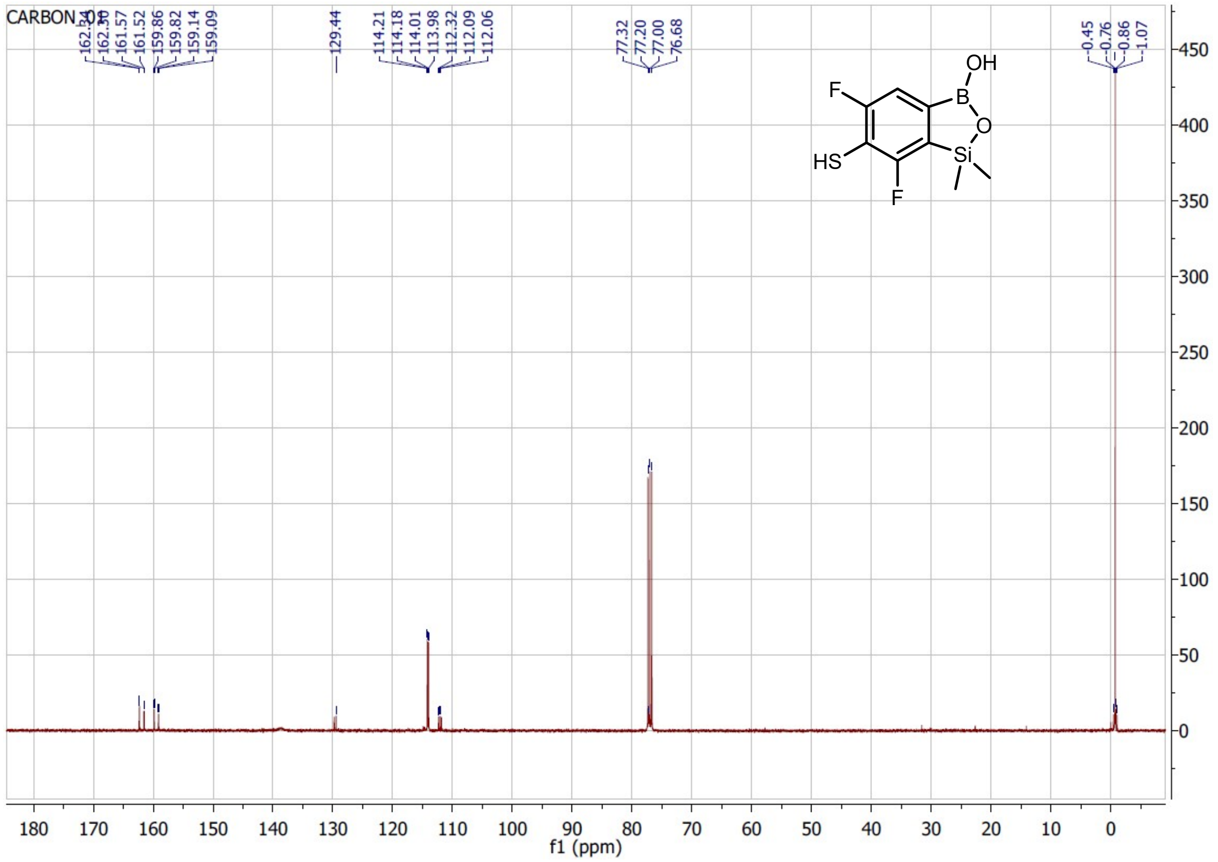

Figure S18. ^{13}C NMR spectrum (101 MHz, $CDCl_3$) of **1e**.

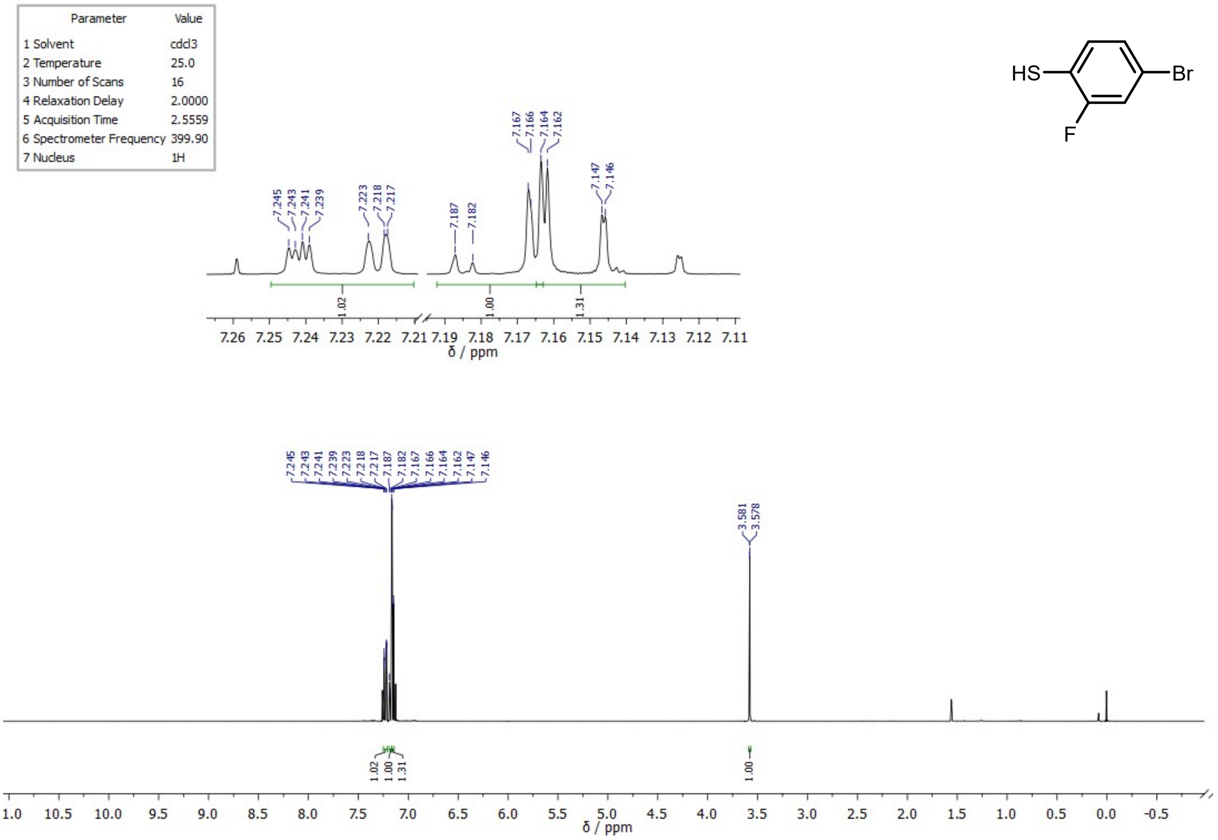

Figure S19. ^{1}H NMR spectrum (400 MHz, $CDCl_3$) of **2b**.

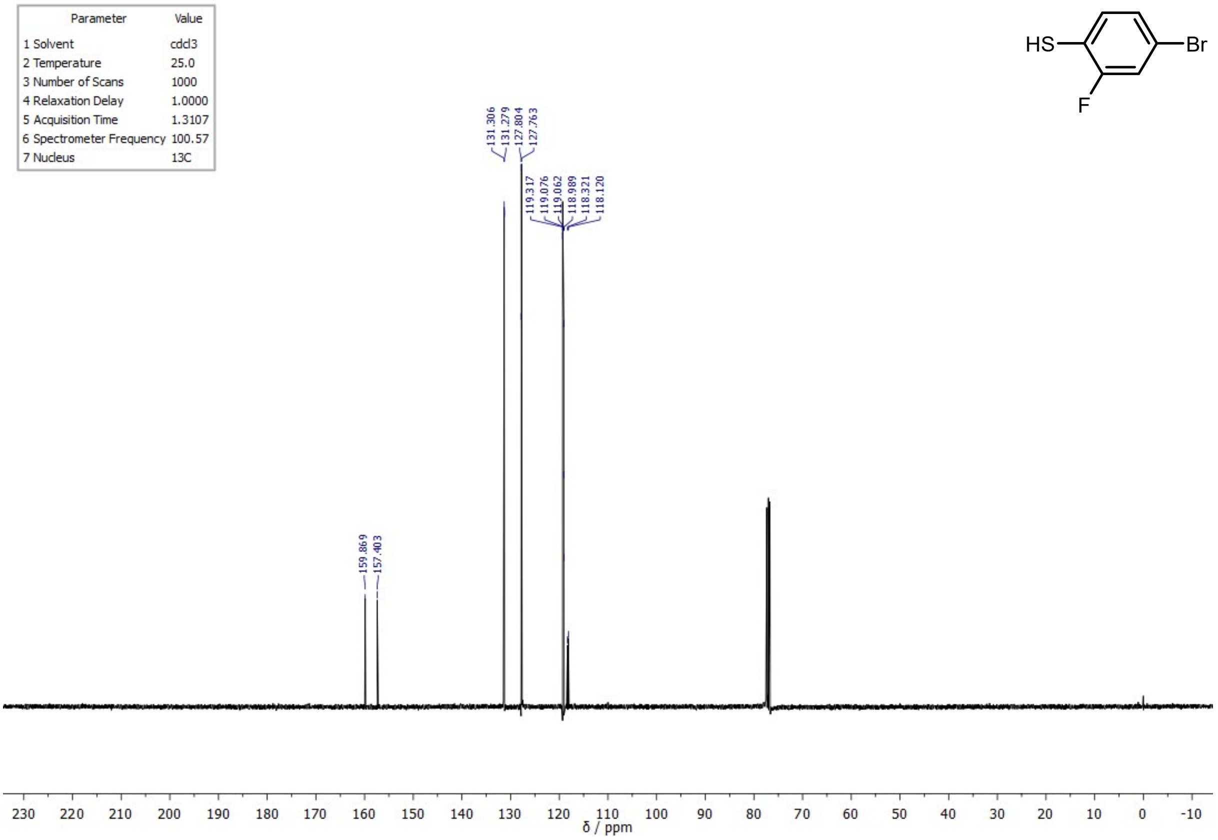

Figure S20. ^{13}C NMR spectrum (101 MHz, $CDCl_3$) of **2b**.

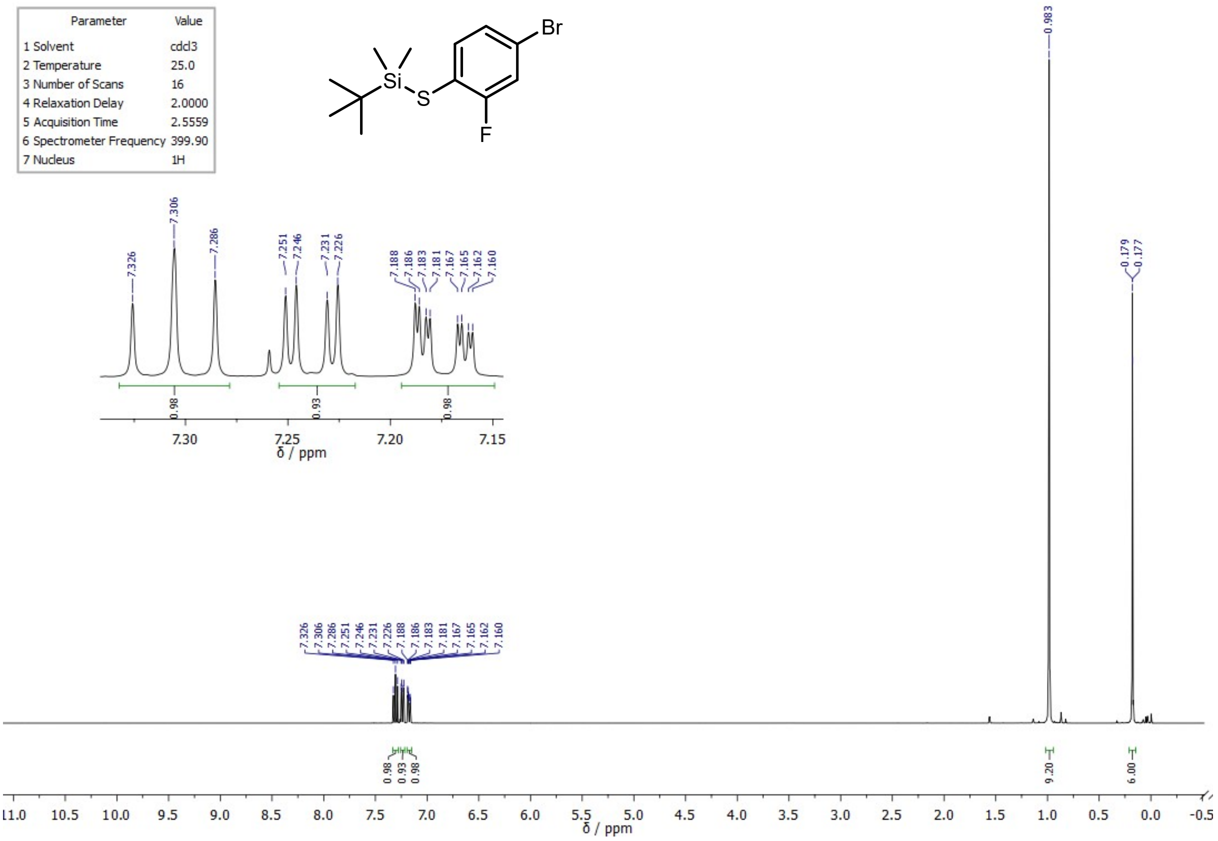

Figure S21. ^{1}H NMR spectrum (400 MHz, $CDCl_3$) of **2c**.

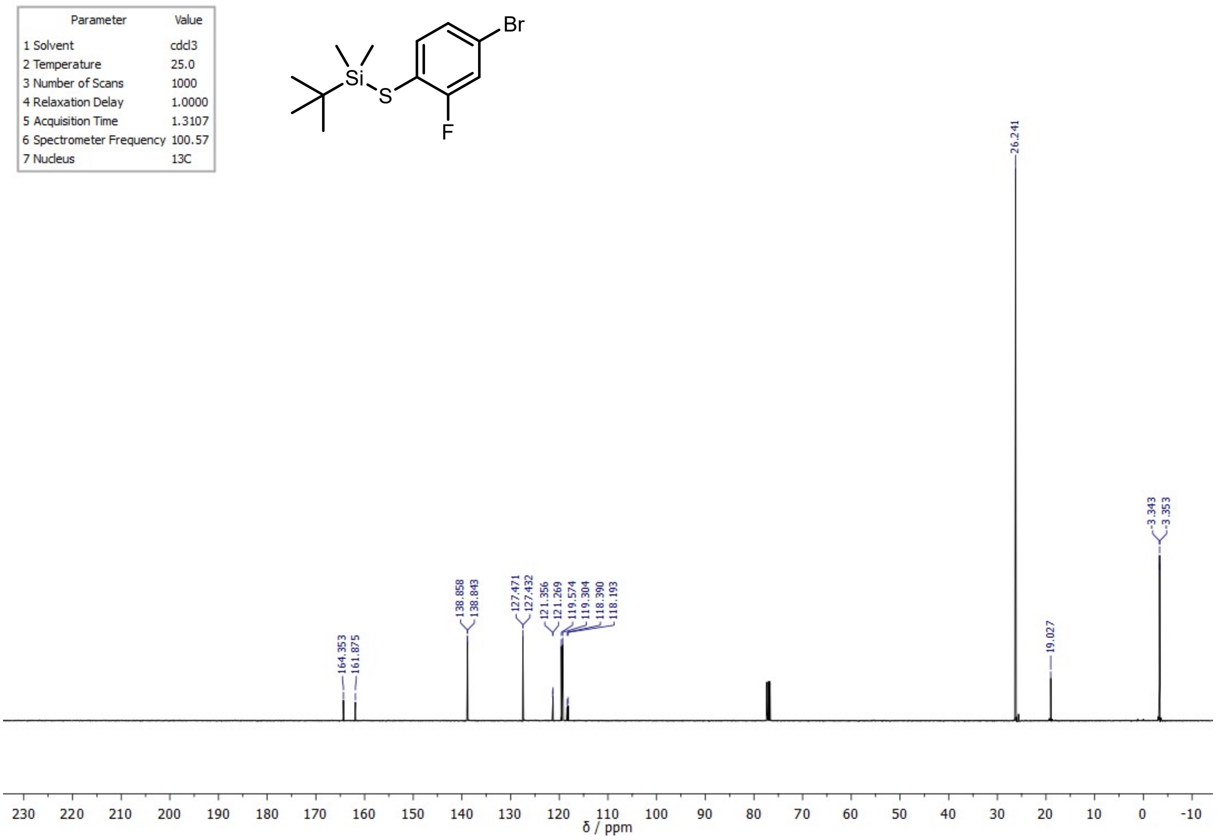

Figure S22. ^{13}C NMR spectrum (101 MHz, $CDCl_3$) of **2c**.

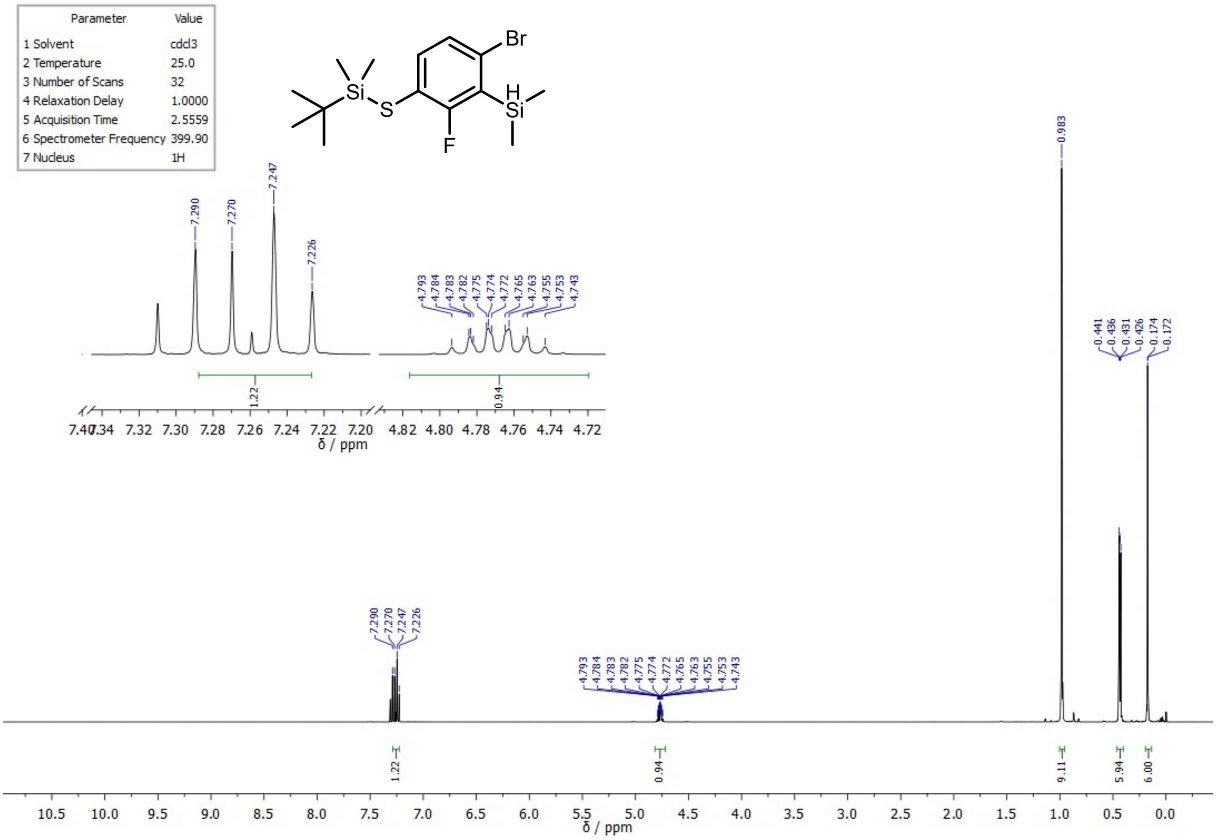

Figure S23. ^{1}H NMR spectrum (400 MHz, $CDCl_3$) of **2d**.

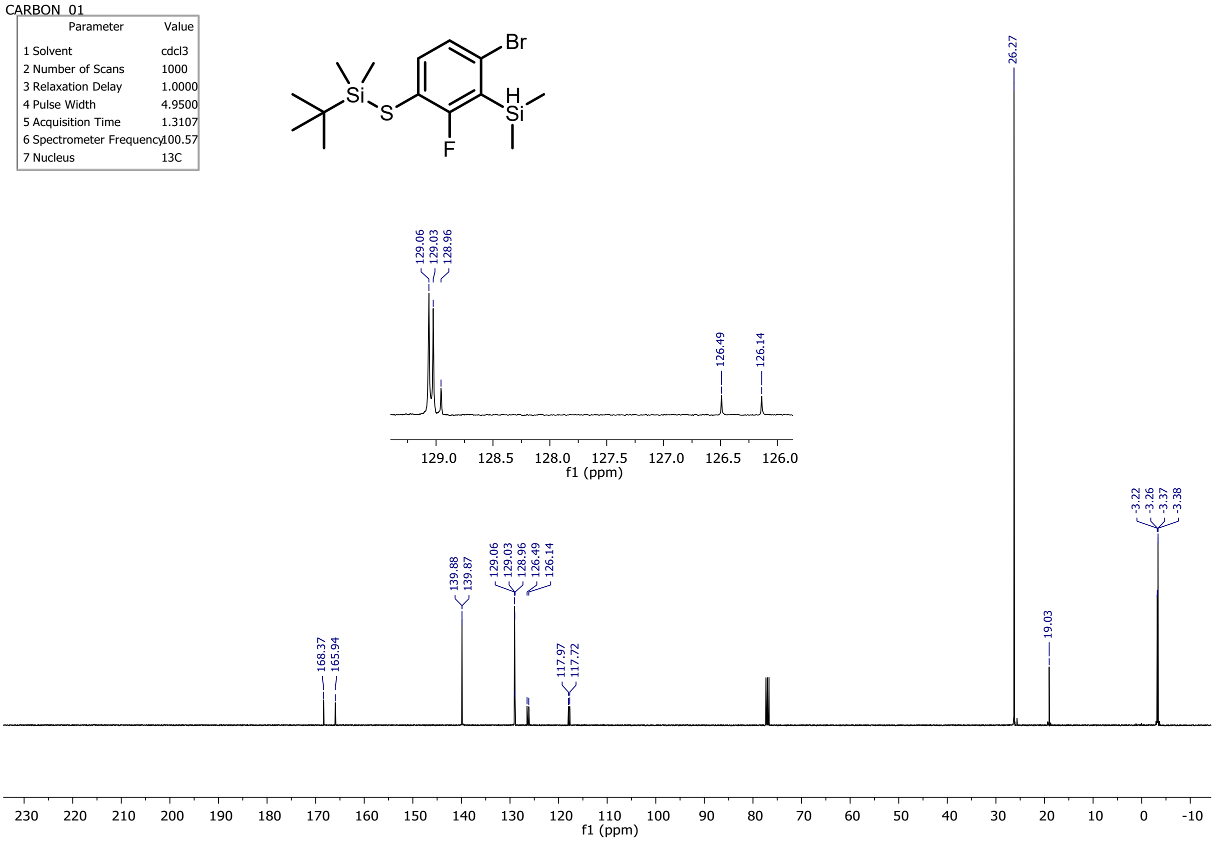

Figure S24. ^{13}C NMR spectrum (101 MHz, $CDCl_3$) of **2d**.

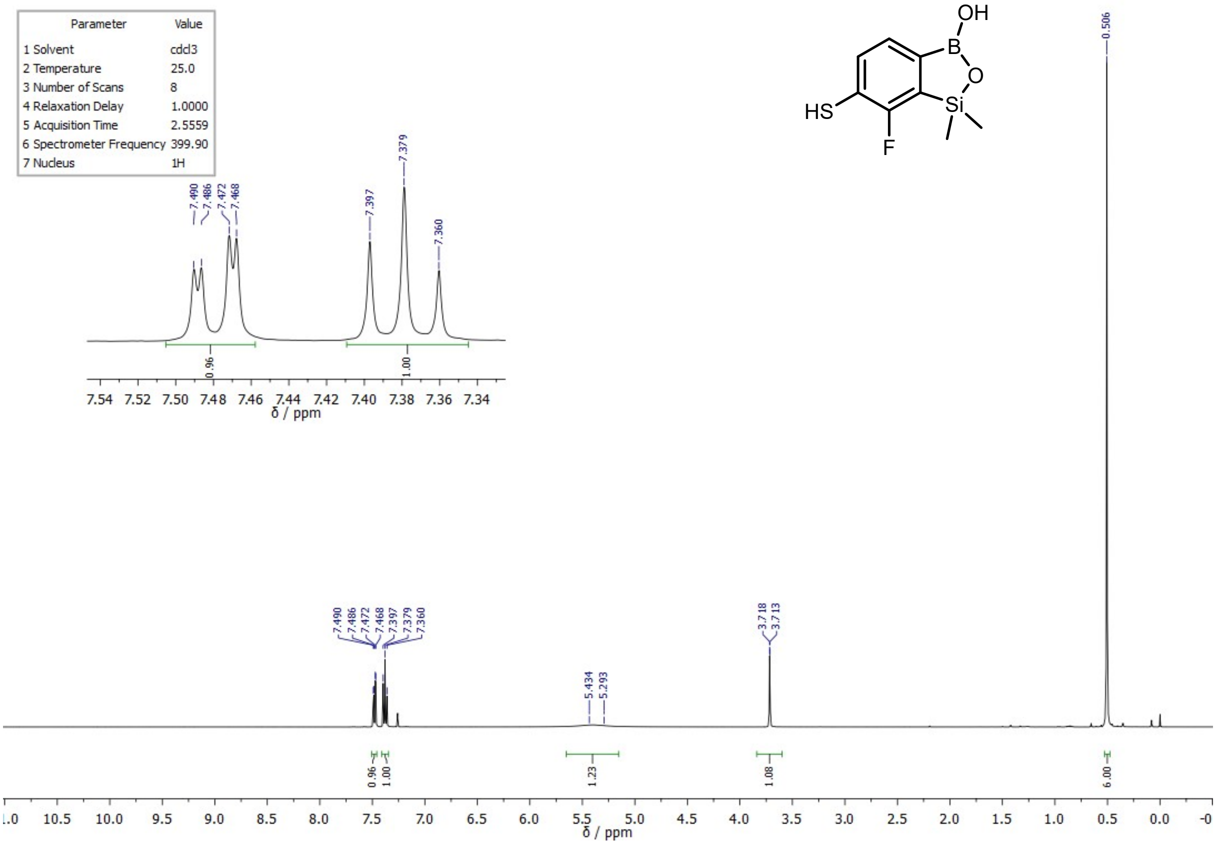

Figure S25.^{1}H NMR spectrum (400 MHz, $CDCl_3$) of **2e**.

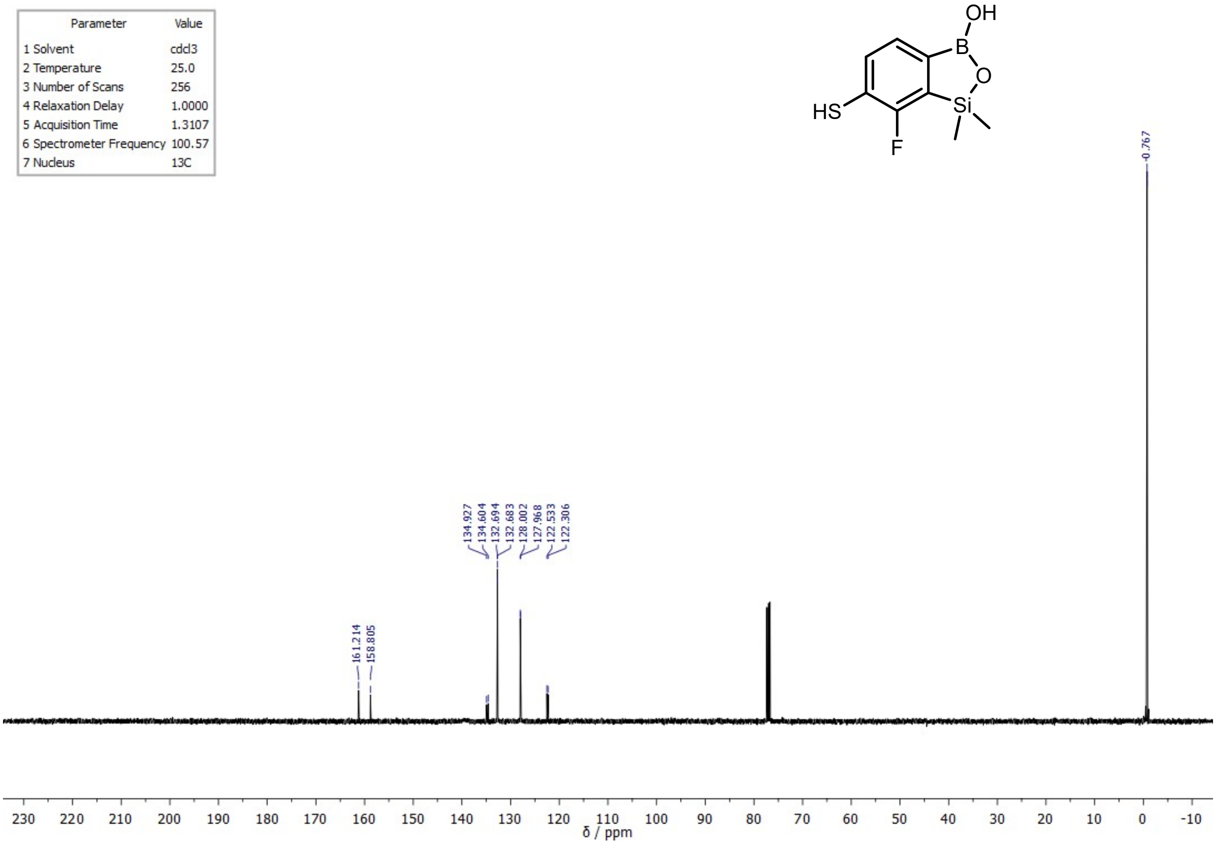

Figure S26. ^{13}C NMR spectrum (101 MHz, $CDCl_3$) of **2e**.

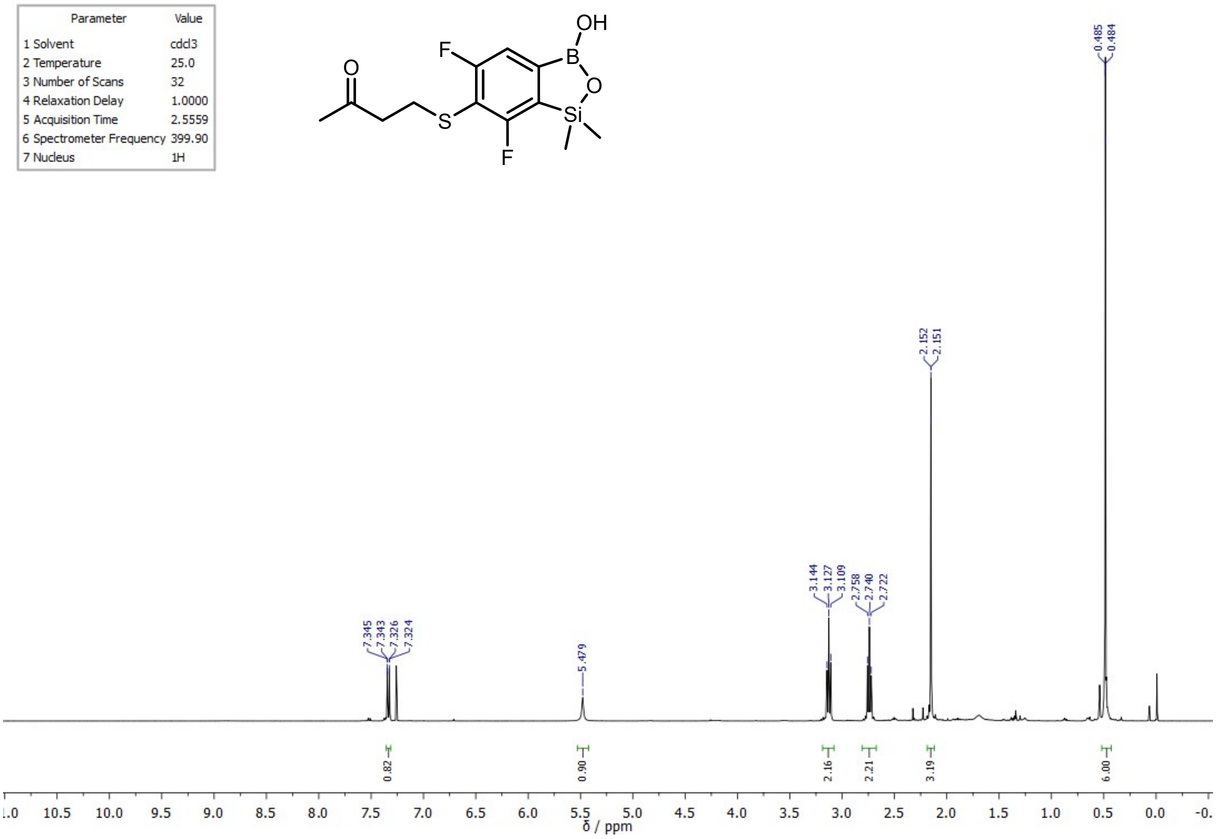

Figure S27. ^{1}H NMR spectrum (400 MHz, $CDCl_3$) of **3**.

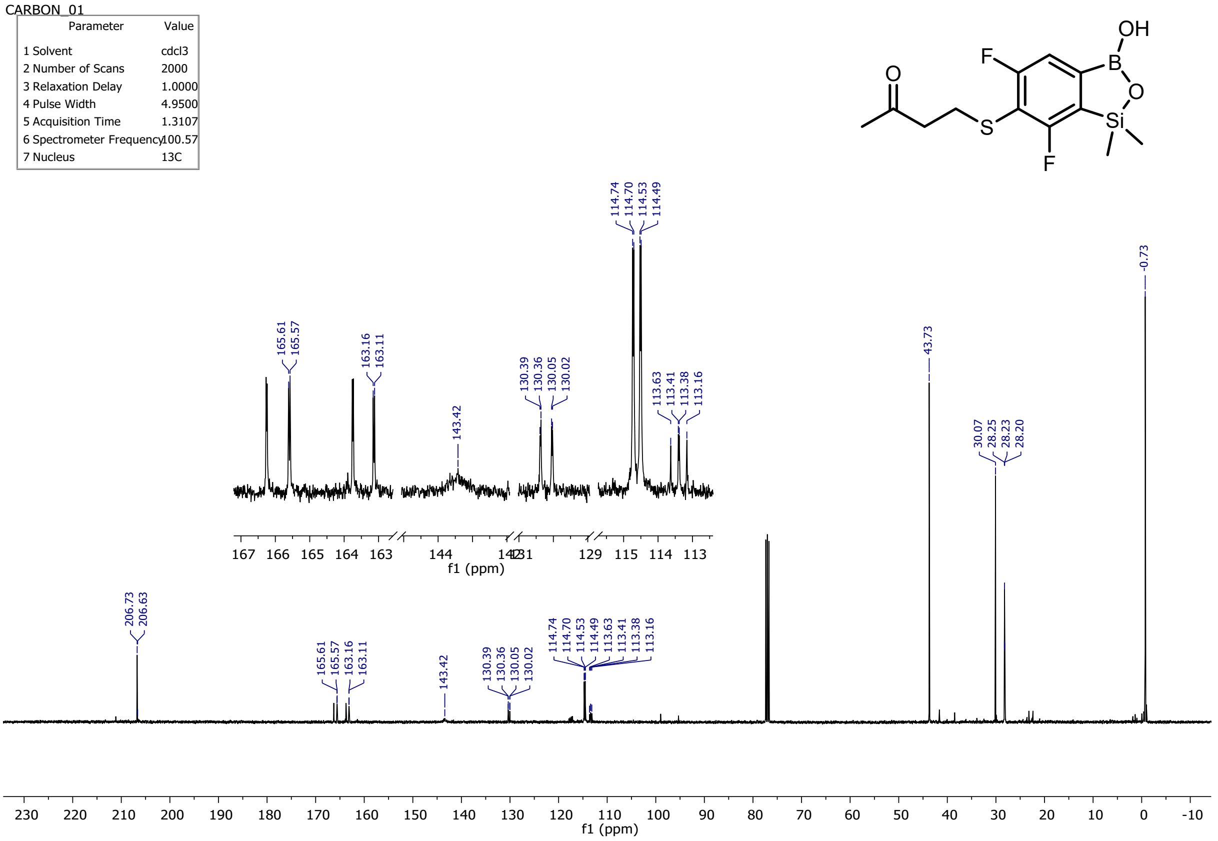

Figure S28. ^{13}C NMR spectrum (101 MHz, $CDCl_3$) of **3**.

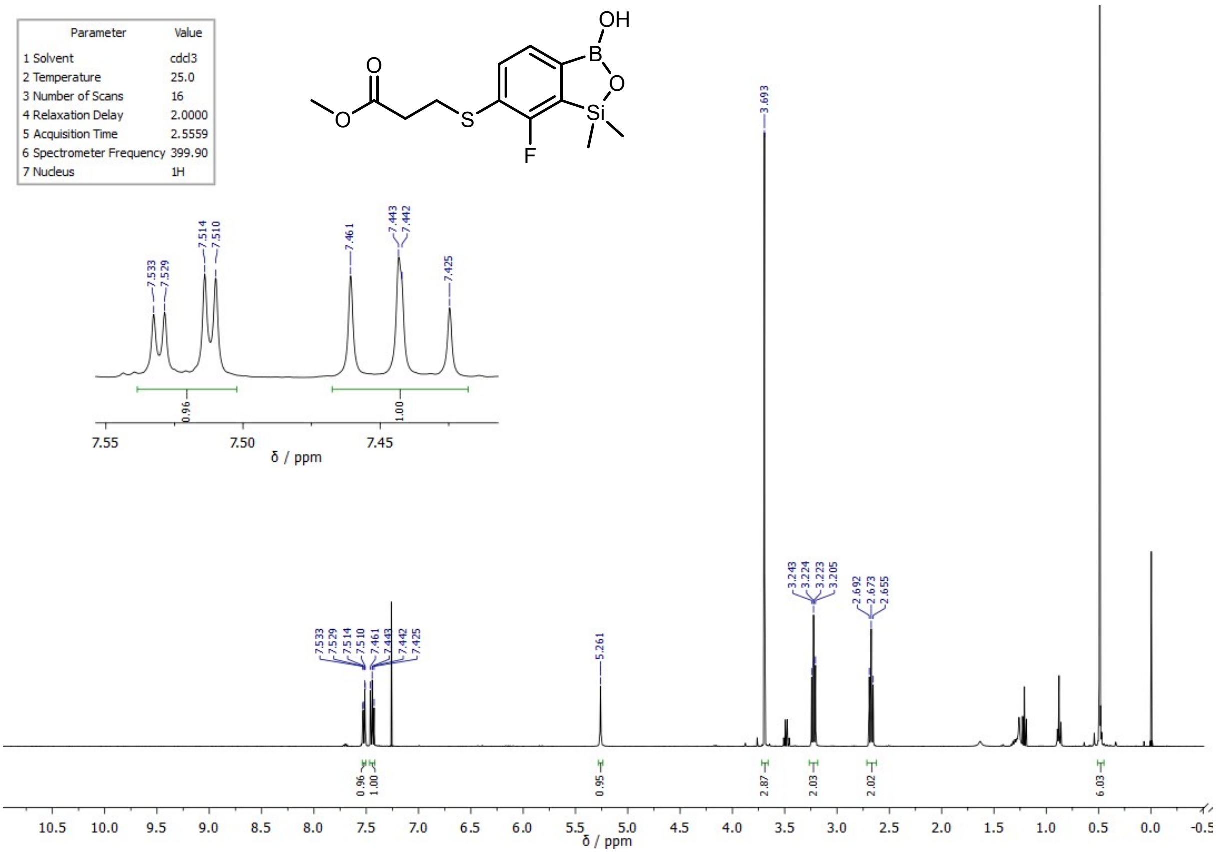

Figure S29. ^{1}H NMR spectrum (400 MHz, $CDCl_3$) of **4**.

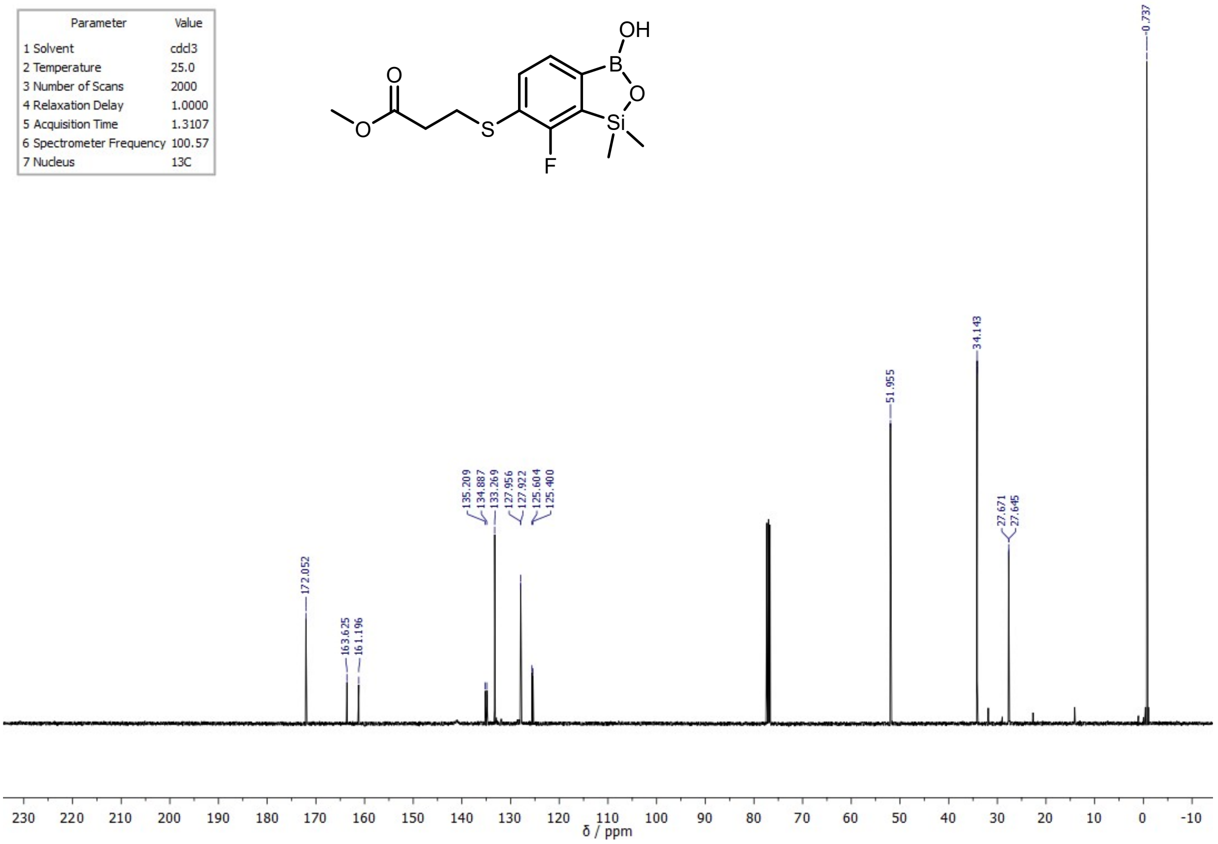

Figure S30. ^{13}C NMR spectrum (101 MHz, $CDCl_3$) of **4**.

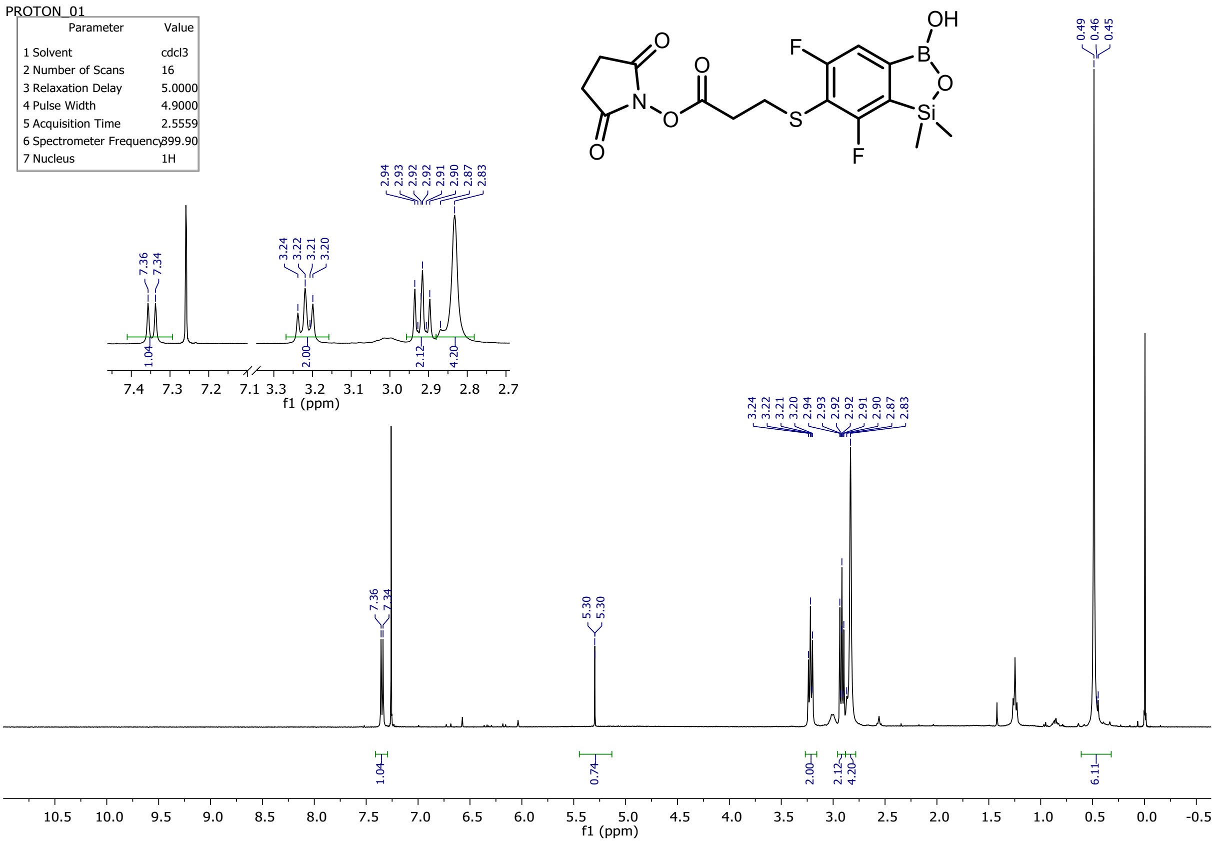

Figure S31. ^{1}H NMR spectrum (400 MHz, $CDCl_3$) of **5**.

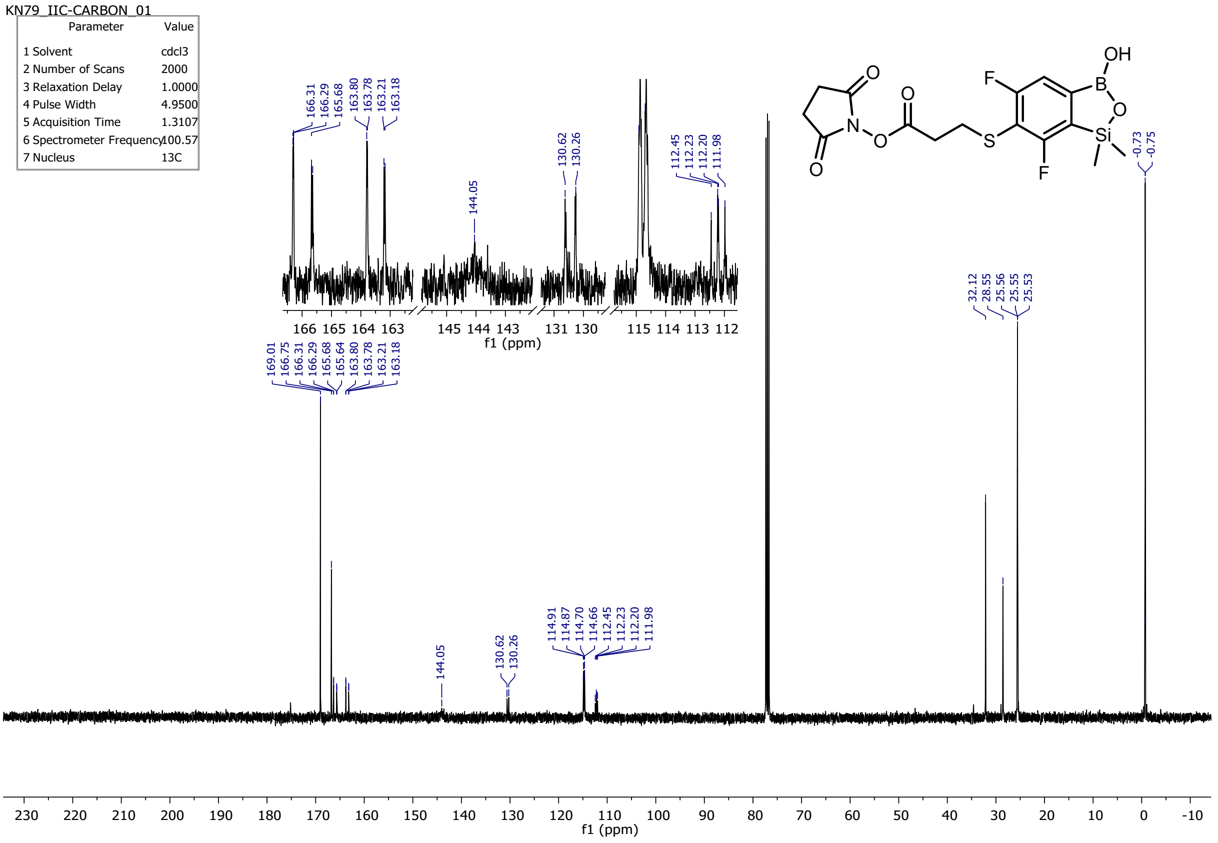

Figure S32. ^{13}C NMR spectrum (101 MHz, $CDCl_3$) of **5**.

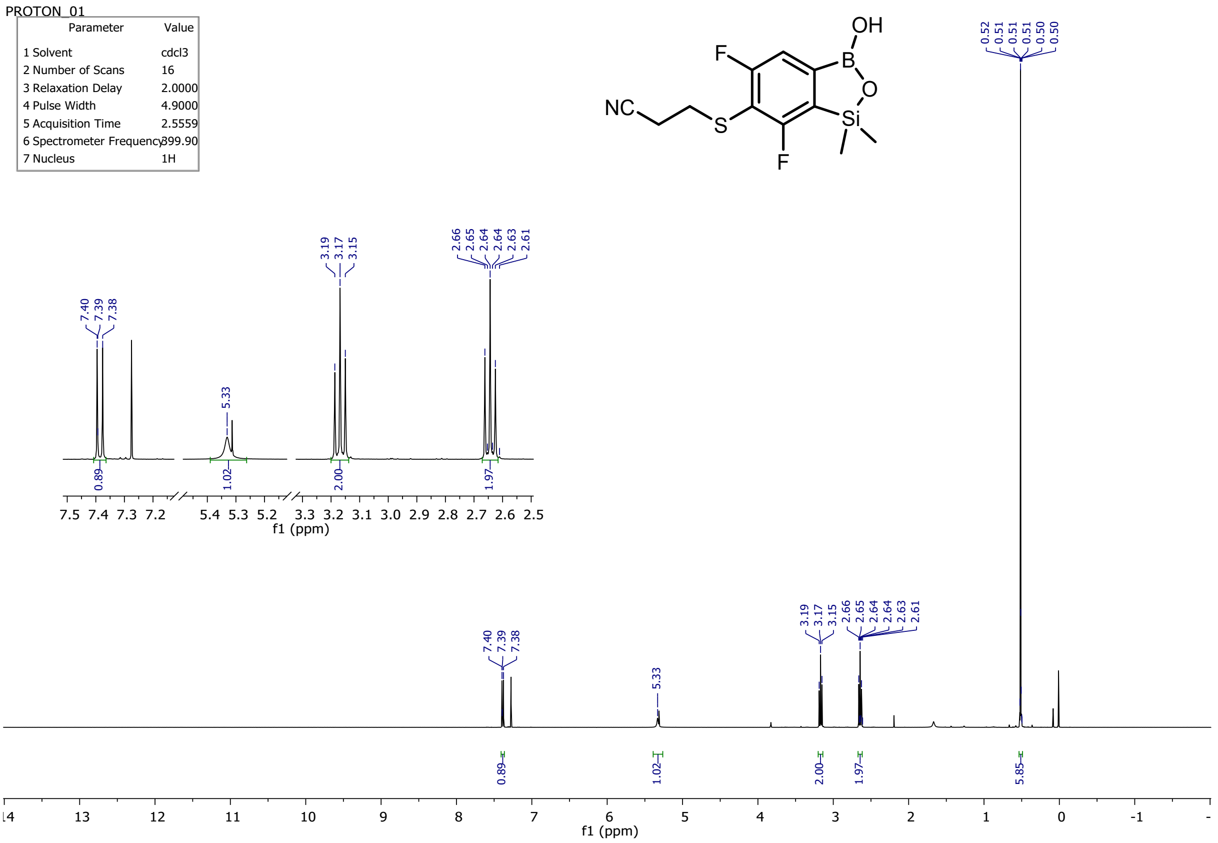

Figure S33. ^{1}H NMR spectrum (400 MHz, $CDCl_3$) of **6**.

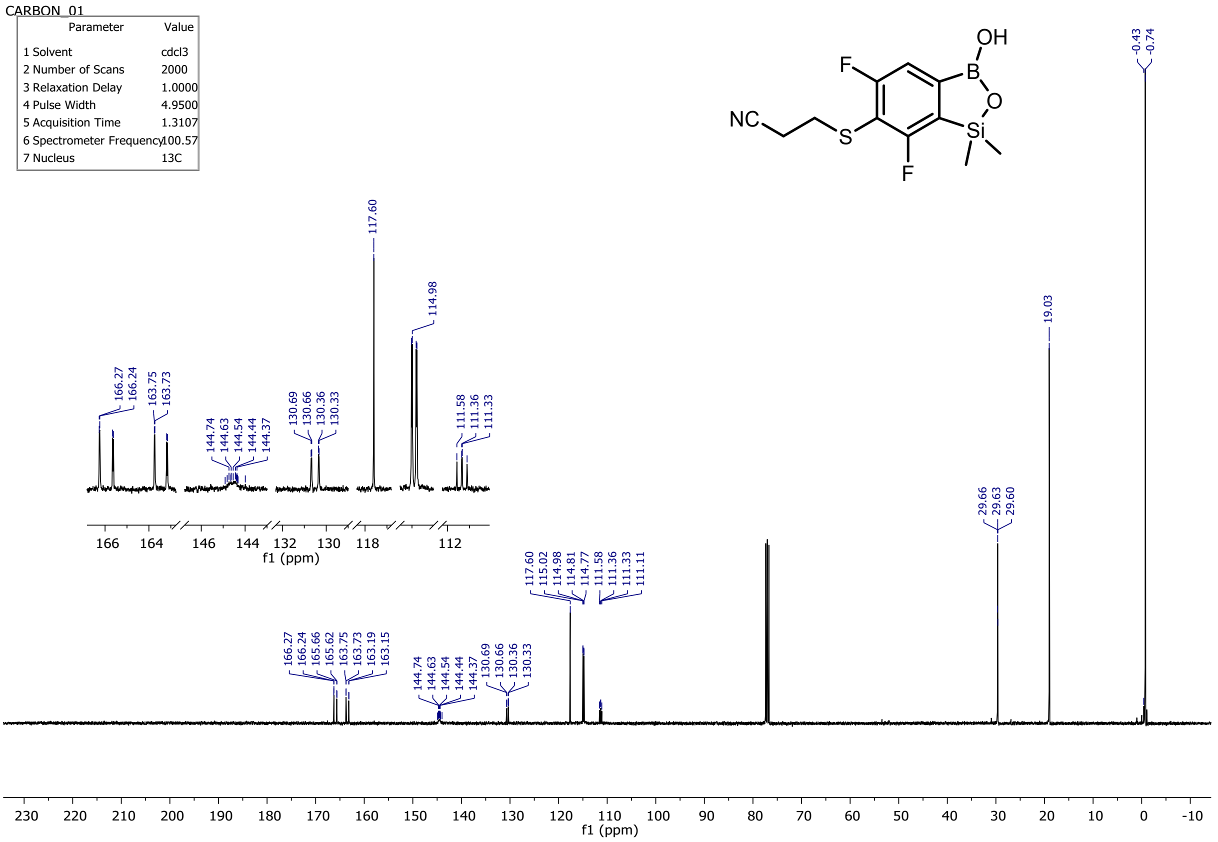

Figure S34. ^{13}C NMR spectrum (101 MHz, $CDCl_3$) of **6**.

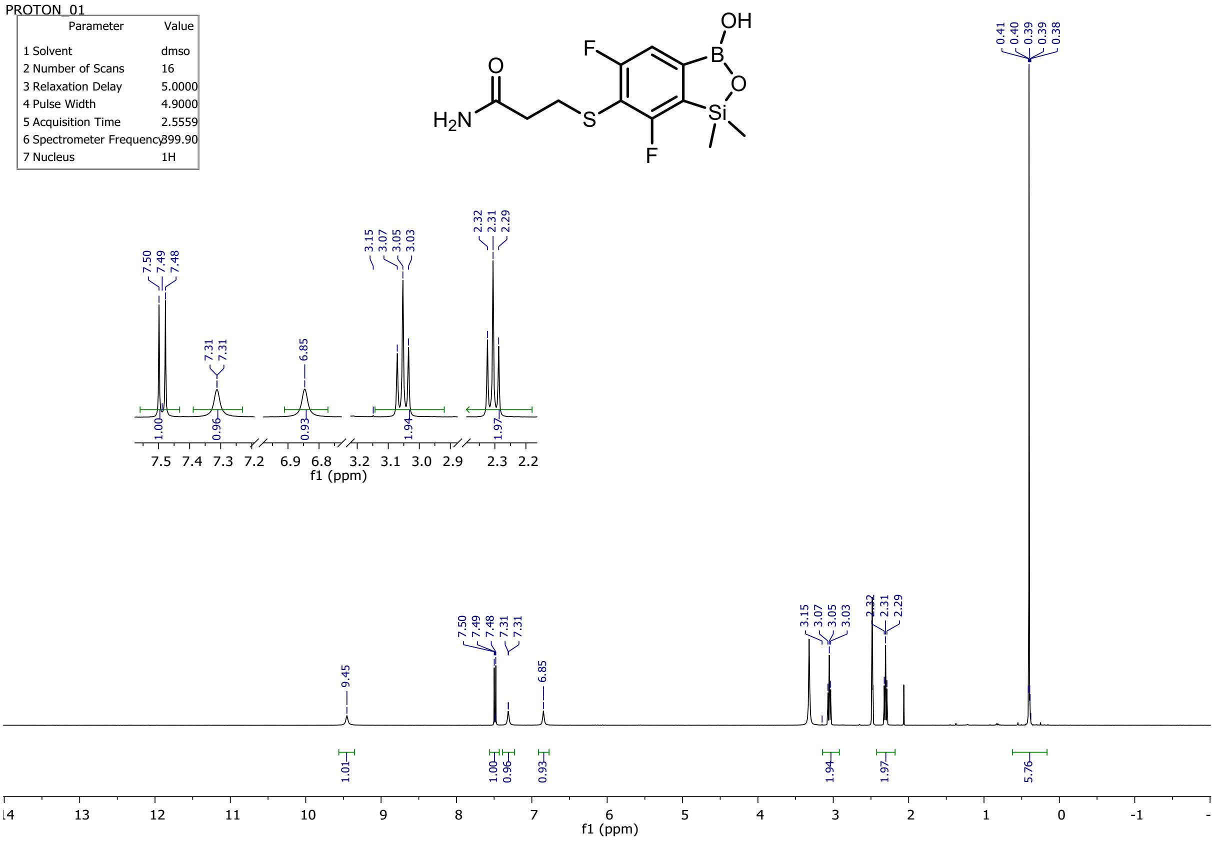

Figure S35. ^{1}H NMR spectrum (400 MHz, DMSO-d_6) of **7**.

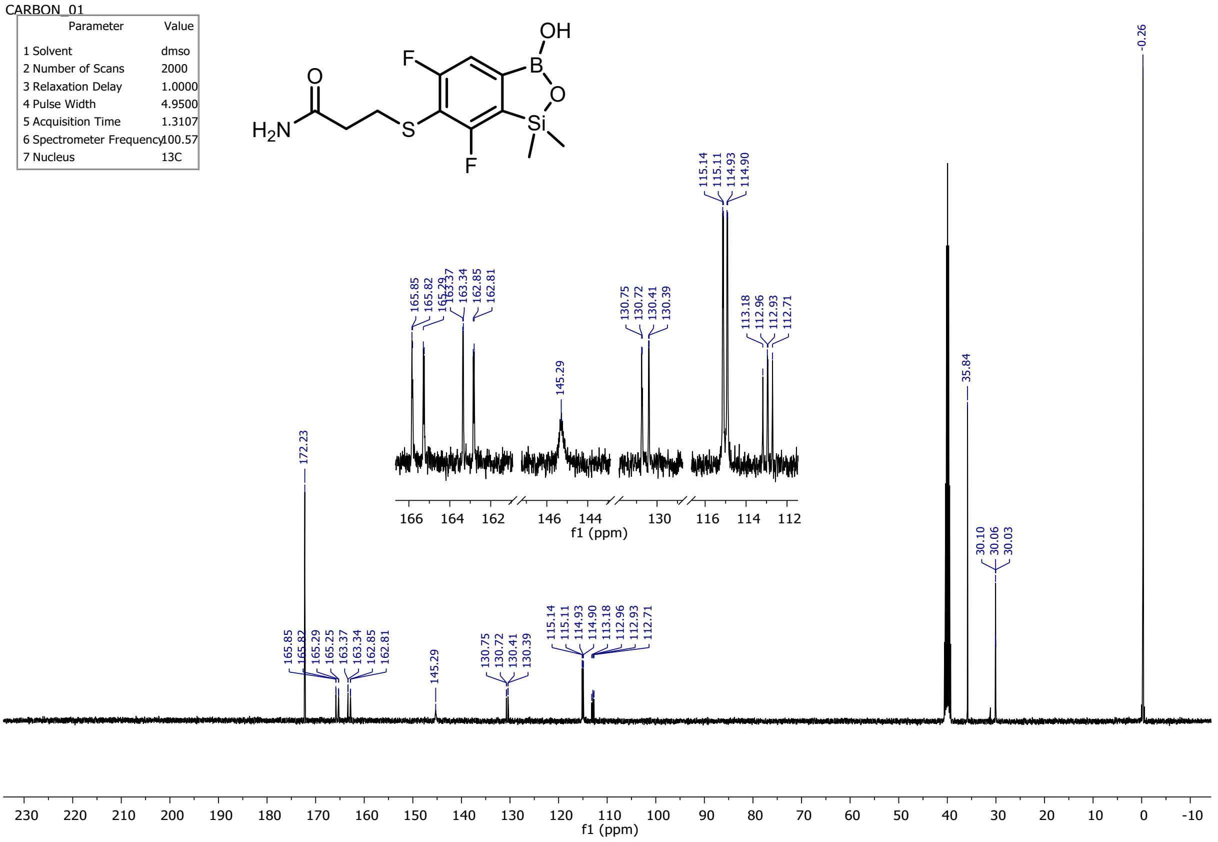

Figure S36. ^{13}C NMR spectrum (101 MHz, DMSO-d_6) of **7**.

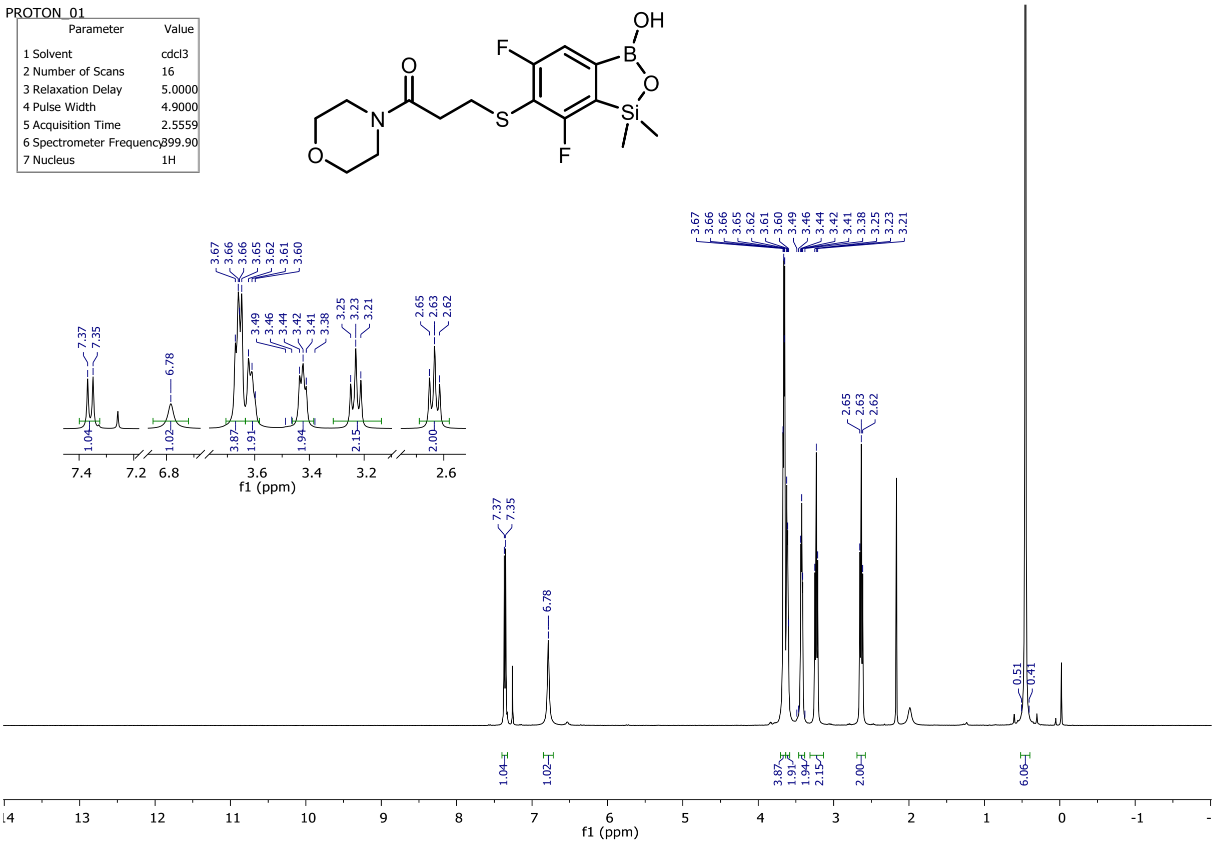

Figure S37. ^{1}H NMR spectrum (400 MHz, $CDCl_3$) of **8**.

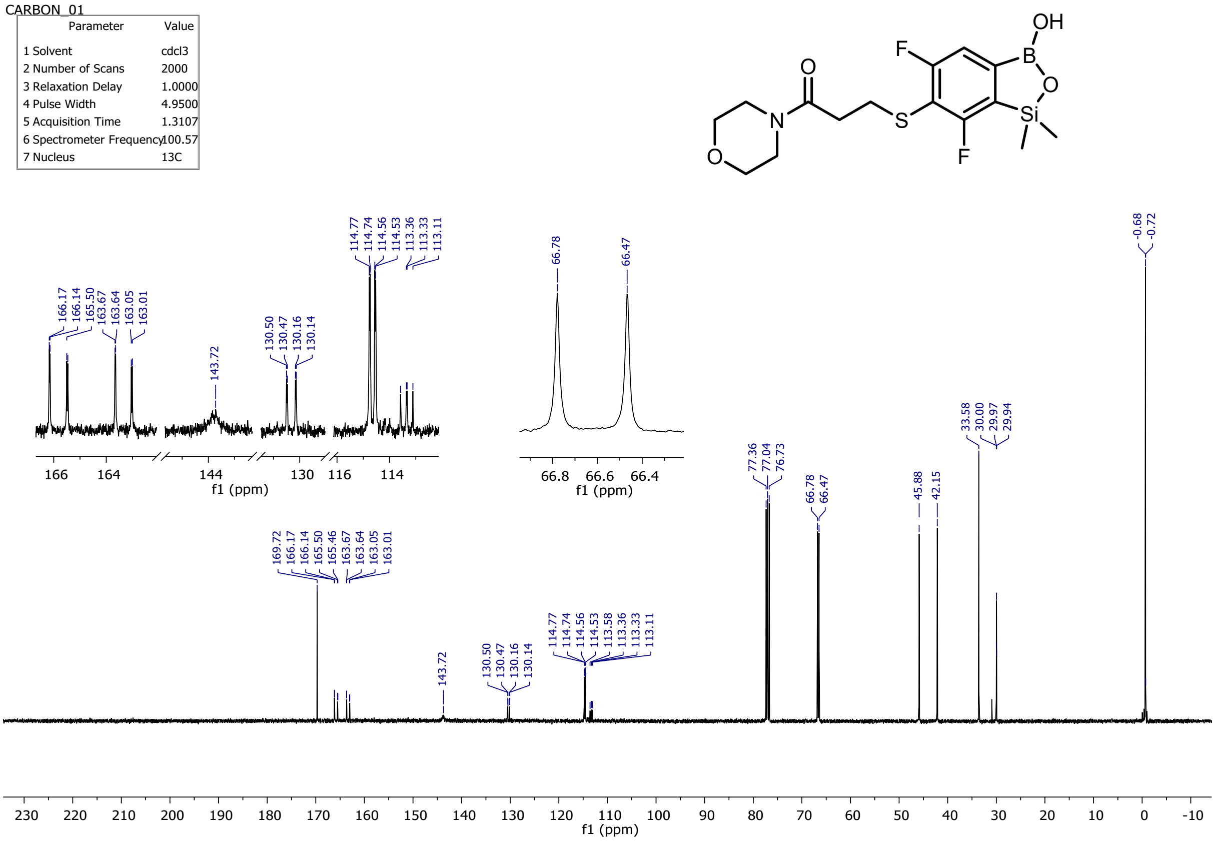

Figure S38. ^{13}C NMR spectrum (101 MHz, $CDCl_3$) of **8** .

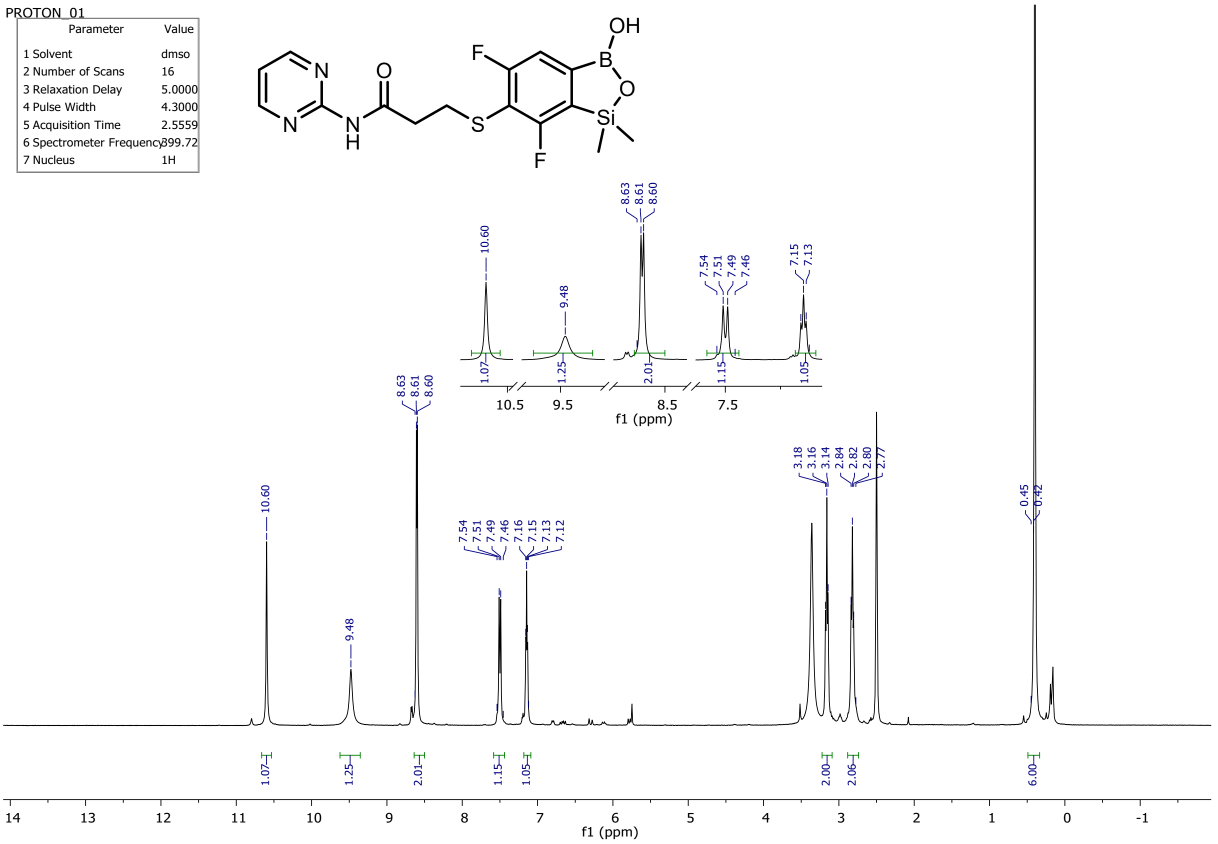

Figure S39. ^{1}H NMR spectrum (400 MHz, DMSO-d_6) of **9**.

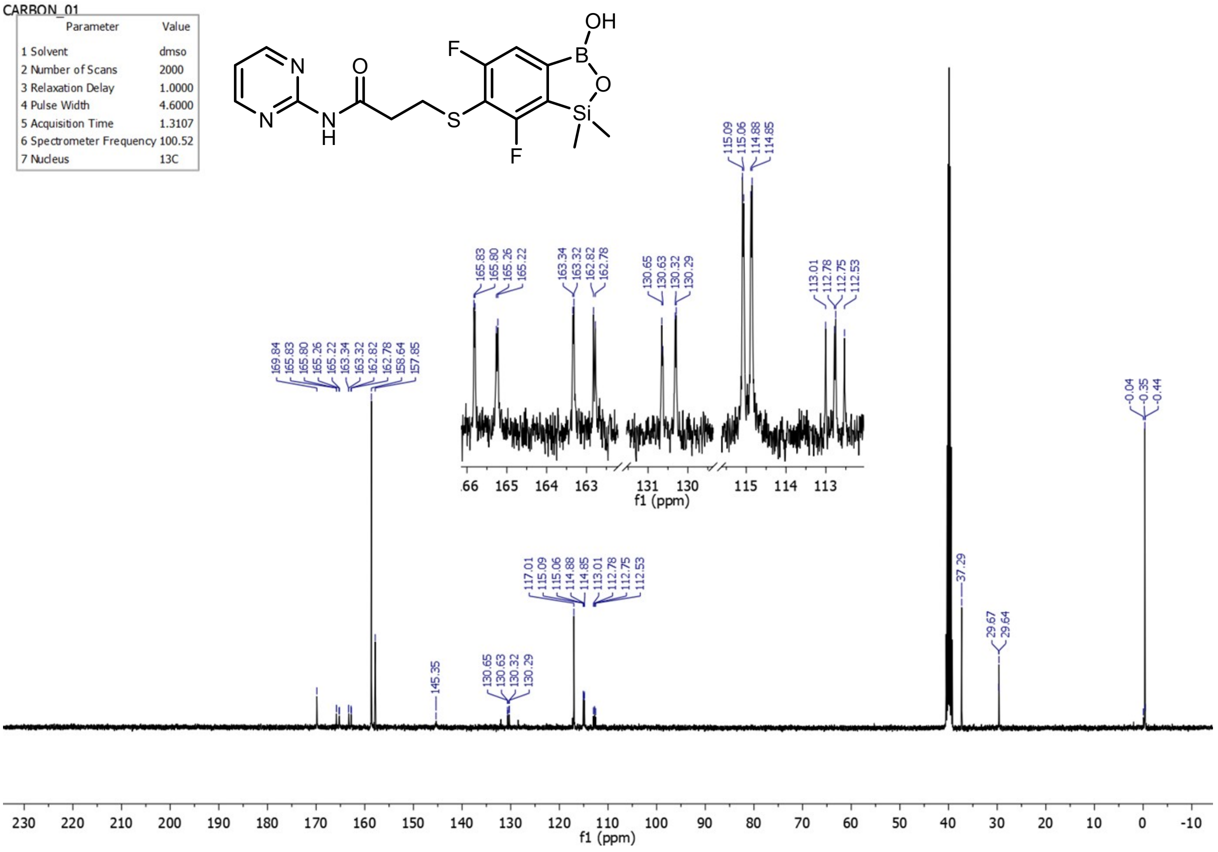

Figure S40. ^{13}C NMR spectrum (101 MHz, DMSO-d_6) of **9**.

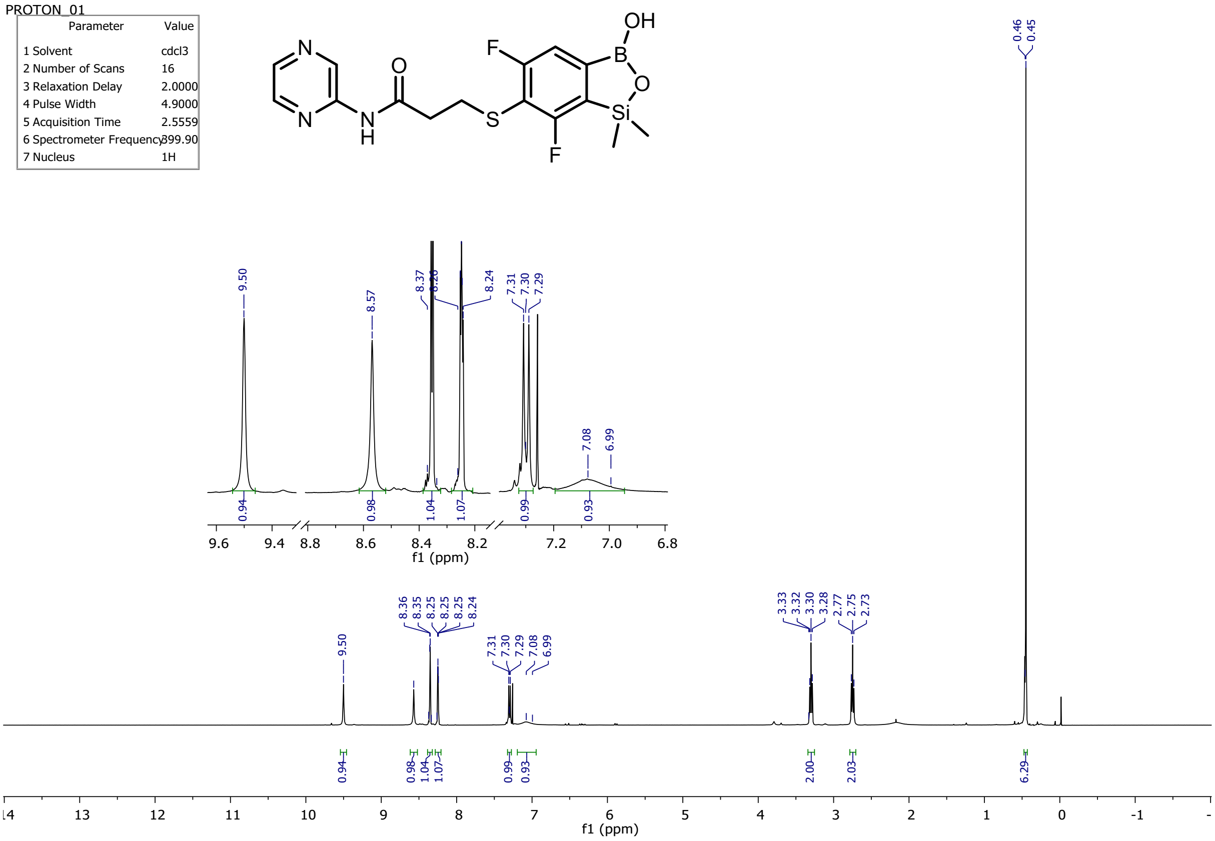

Figure S41. ^{1}H NMR spectrum (400 MHz, $CDCl_3$) of **10**.

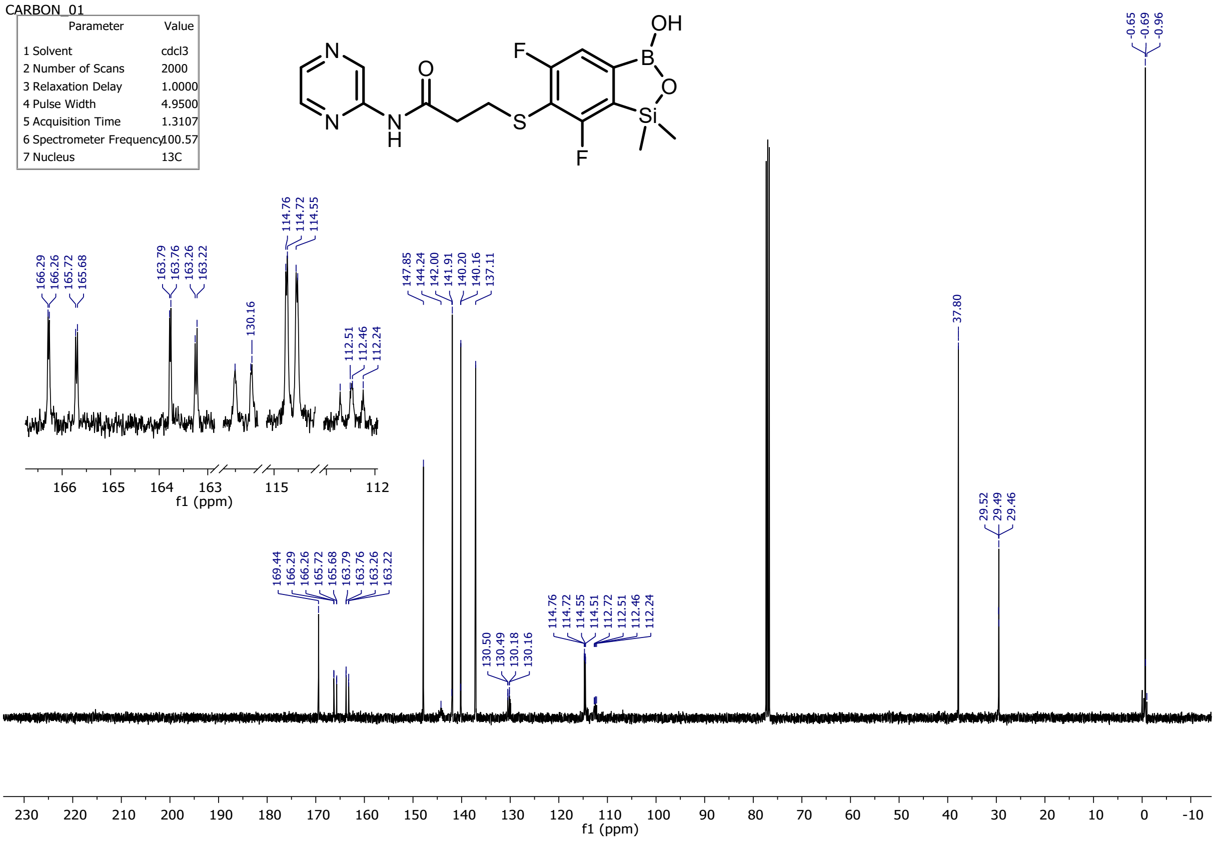

Figure S42. ^{13}C NMR spectrum (101 MHz, $CDCl_3$) of **10**.

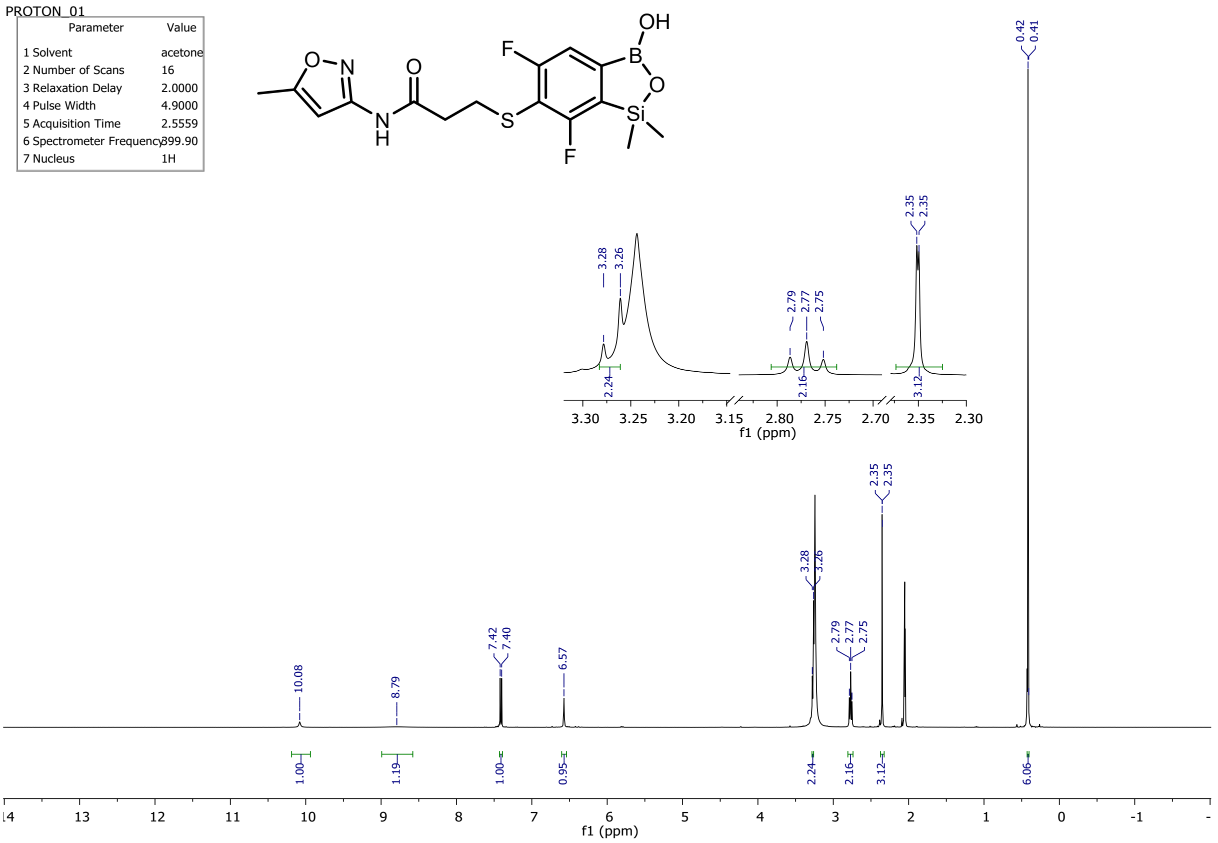

Figure S43. ^{1}H NMR spectrum (400 MHz, acetone-d_6) of **11**.

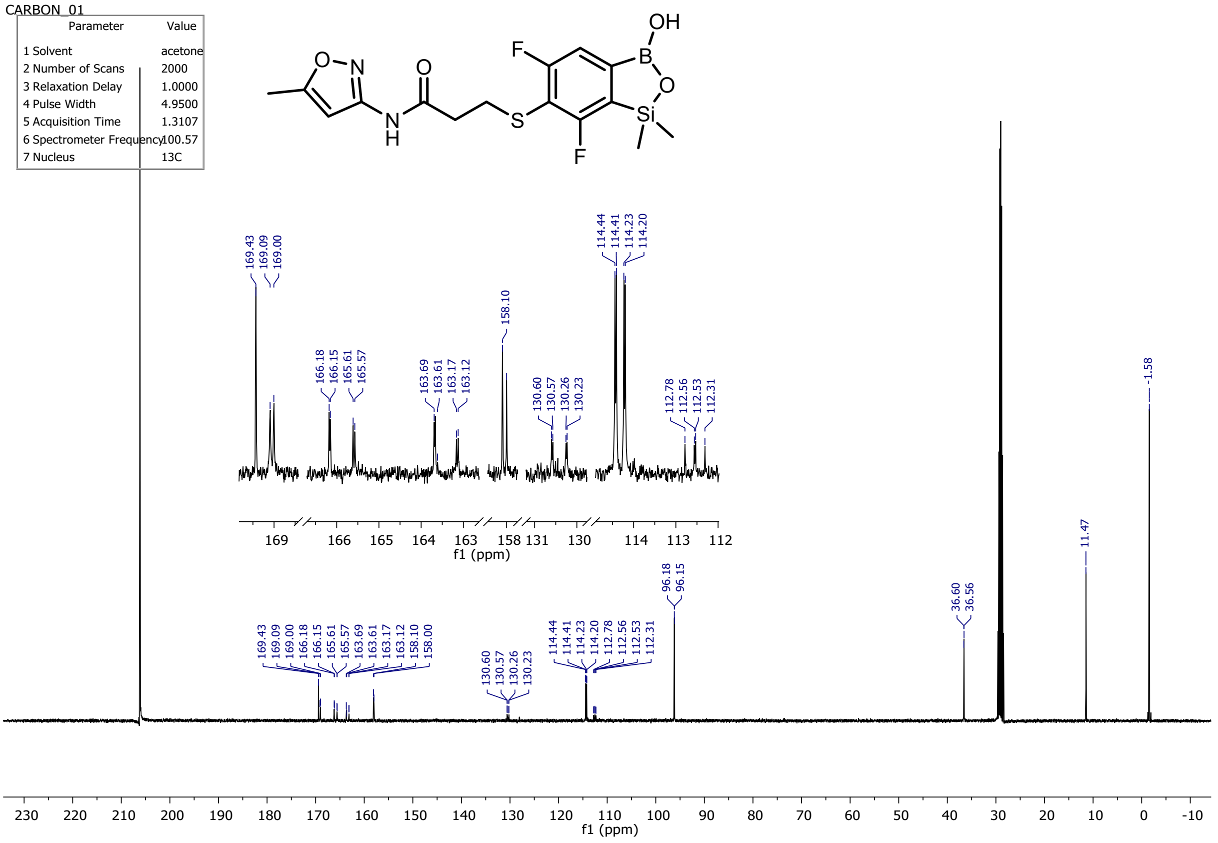

Figure S44. ^{13}C NMR spectrum (101 MHz, acetone-d_6) of **11**.

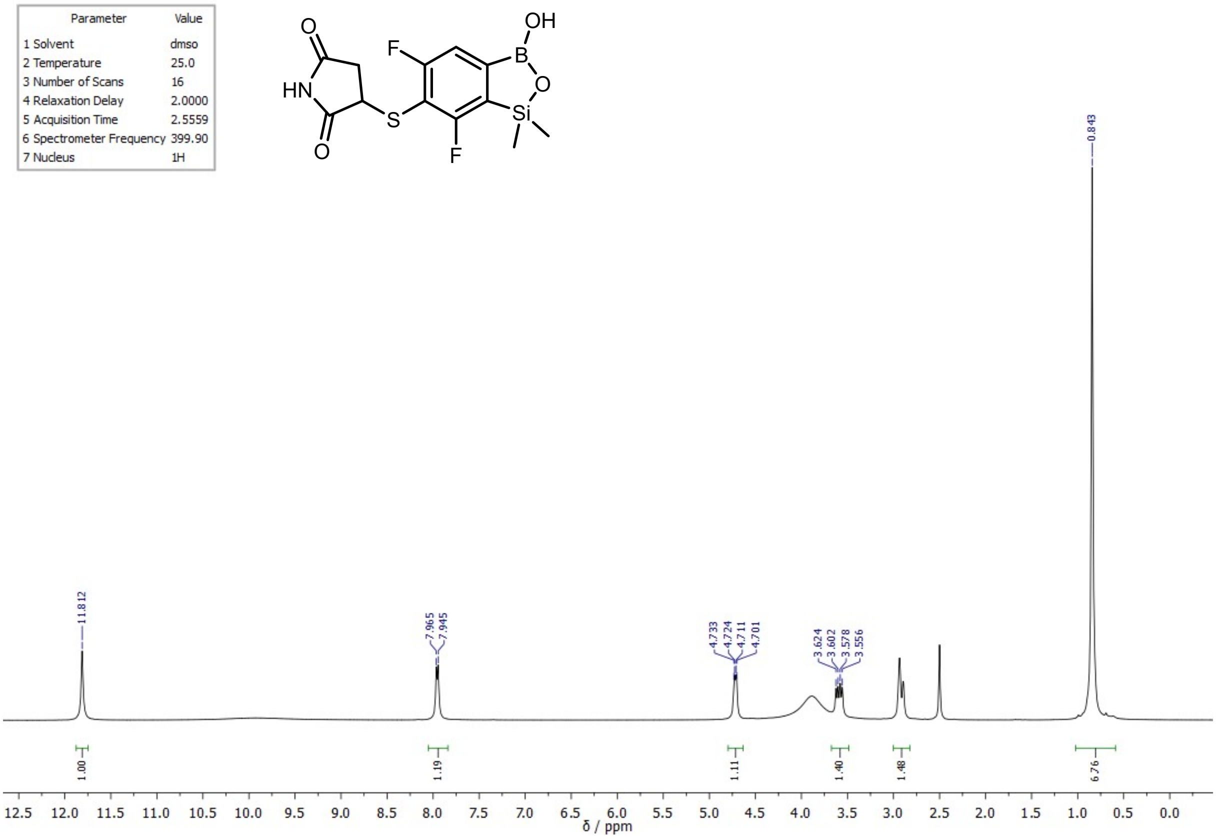

Figure S45. ^{1}H NMR spectrum (400 MHz, DMSO-d_6) of **12**.

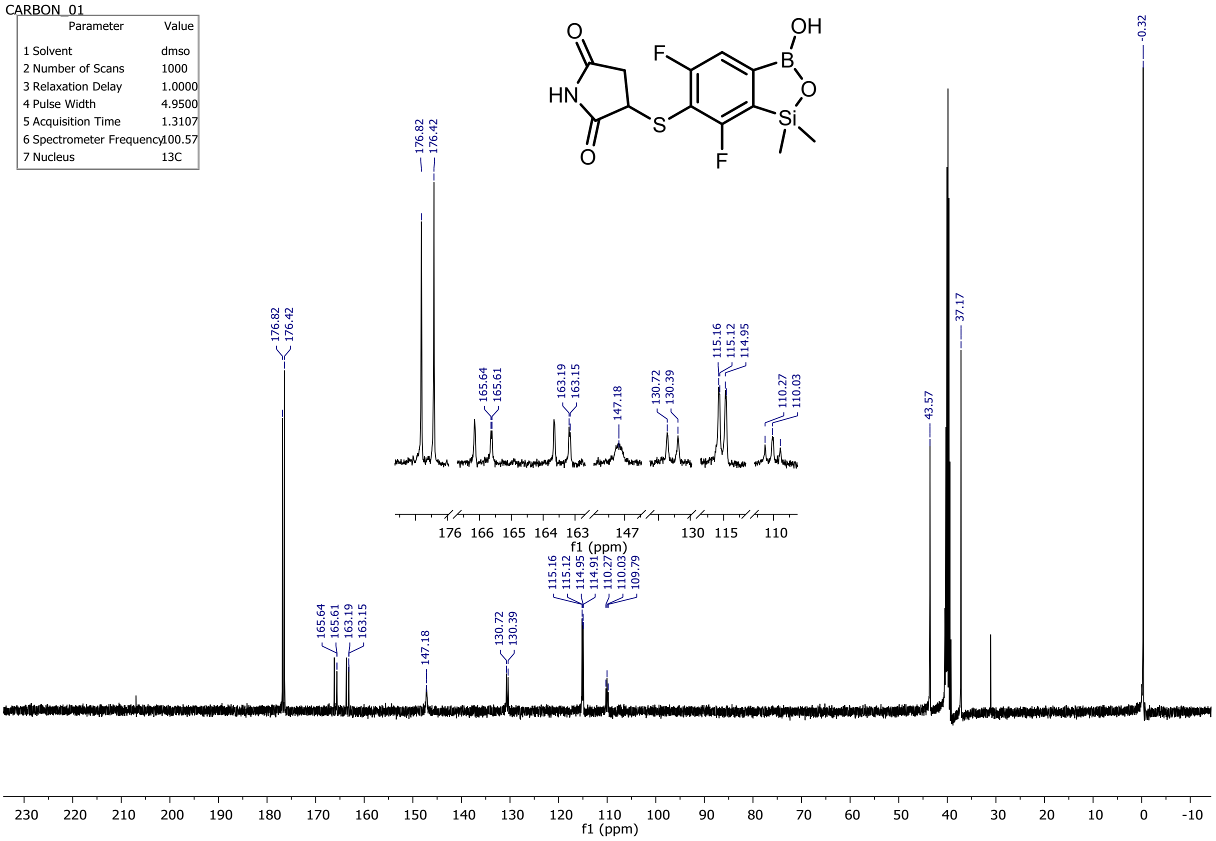

Figure S46. ^{13}C NMR spectrum (101 MHz, DMSO-d_6) of **12**.

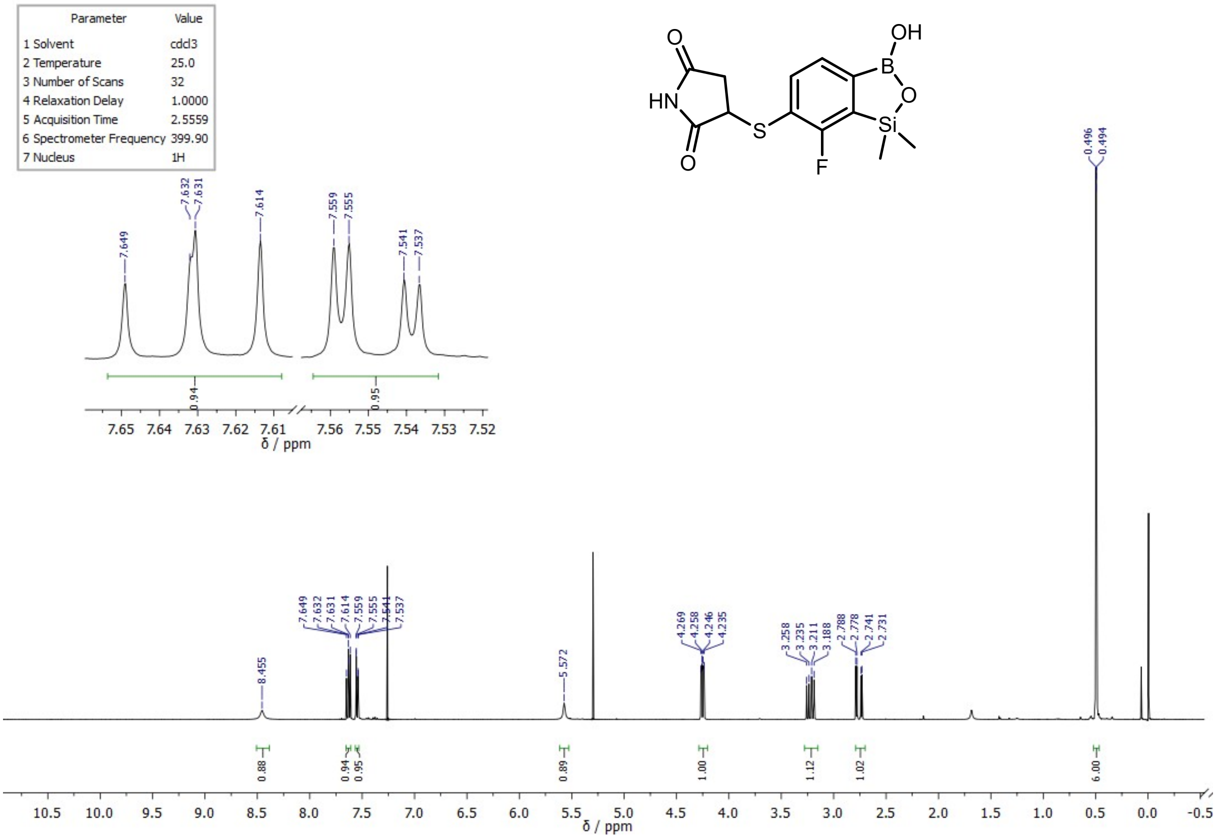

Figure S47. ^{1}H NMR spectrum (400 MHz, $CDCl_3$) of **13**.

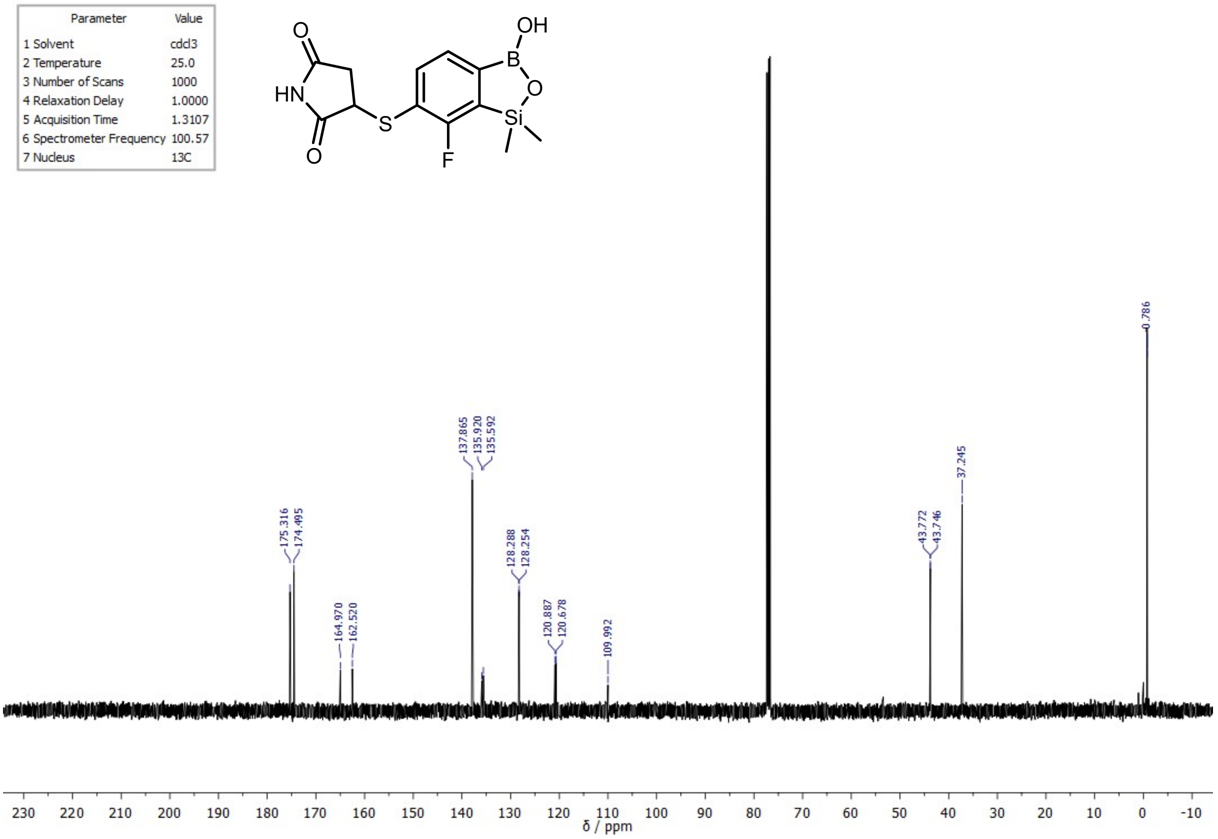

Figure S48. ^{13}C NMR spectrum (101 MHz, $CDCl_3$) of **13**.

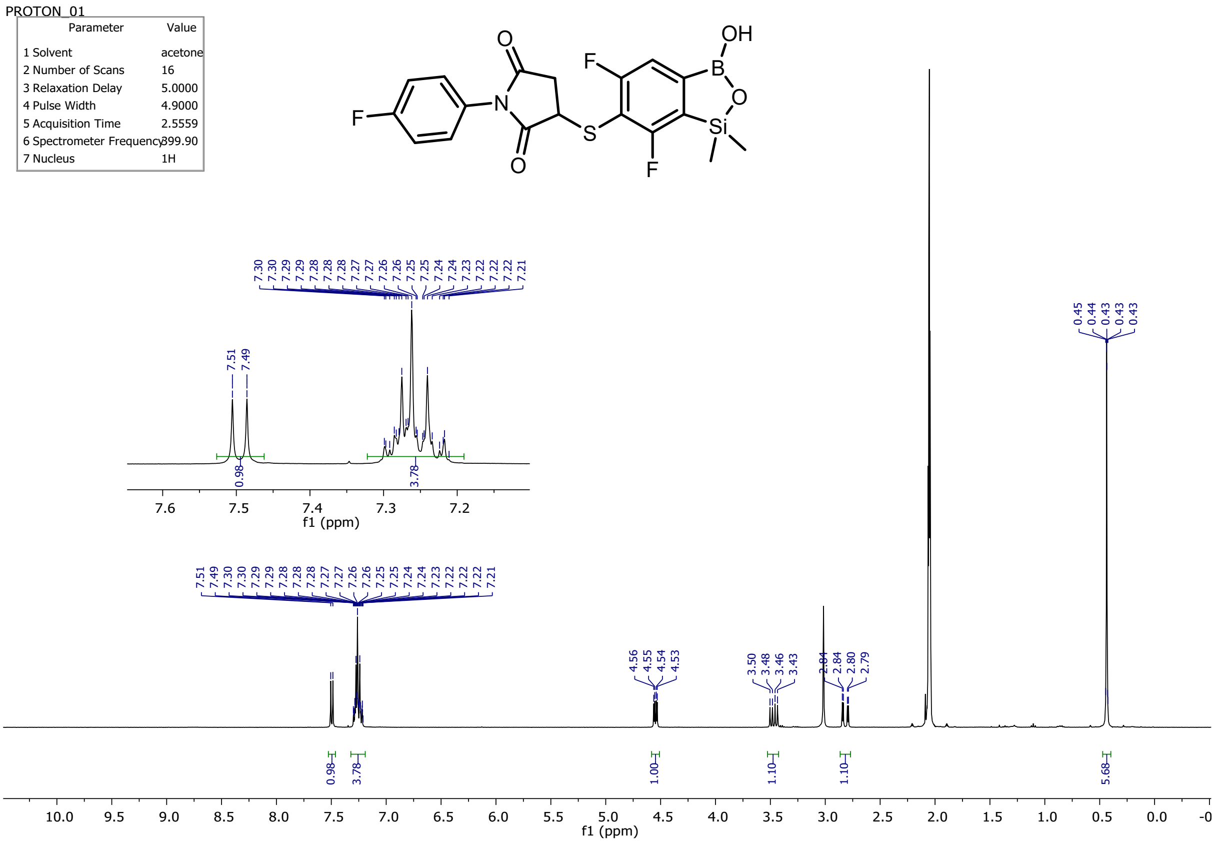

Figure S49. ^{1}H NMR spectrum (400 MHz, acetone-d_6) of **14**.

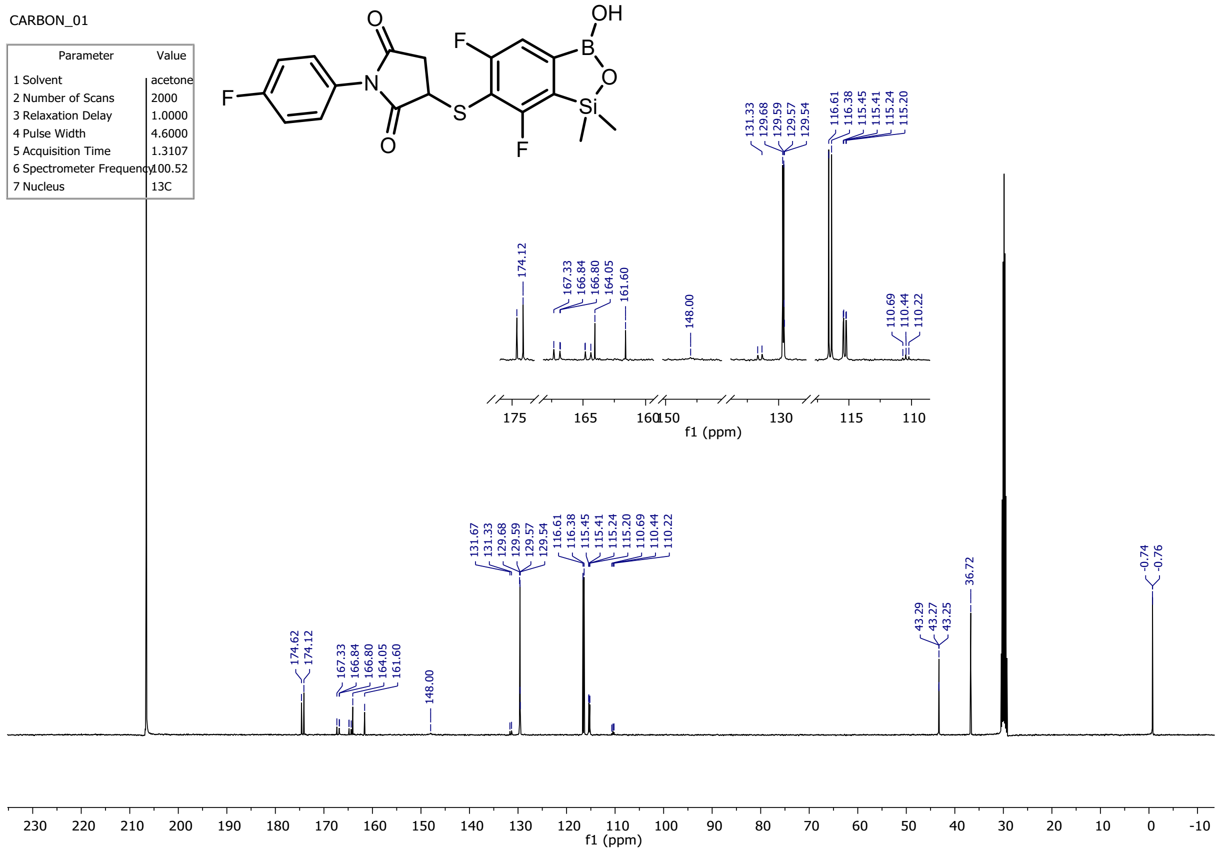

Figure S50. ^{13}C NMR spectrum (101 MHz, acetone-d_6) of **14**.

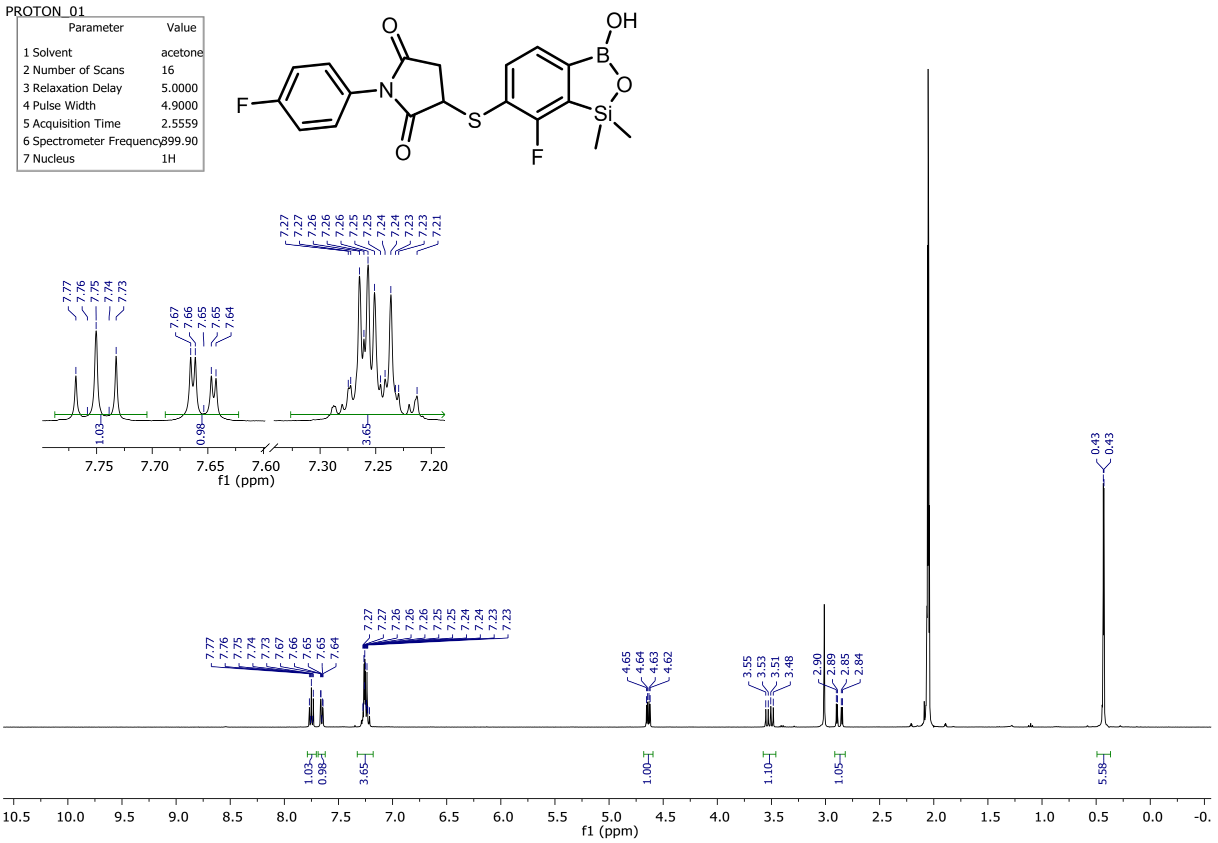

Figure S51. ^{1}H NMR spectrum (400 MHz, acetone-d_6) of **15**.

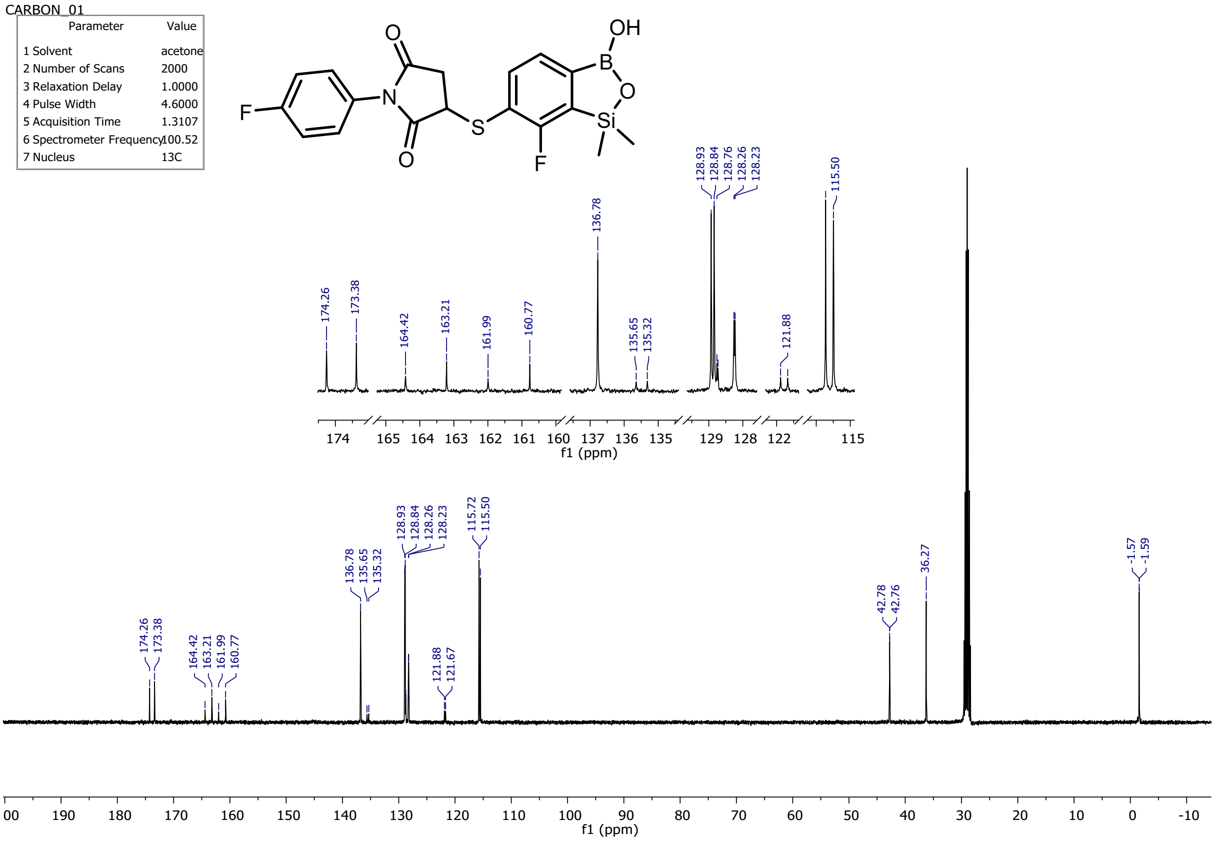

Figure S52. ^{13}C NMR spectrum (101 MHz, acetone-d_6) of **15**.

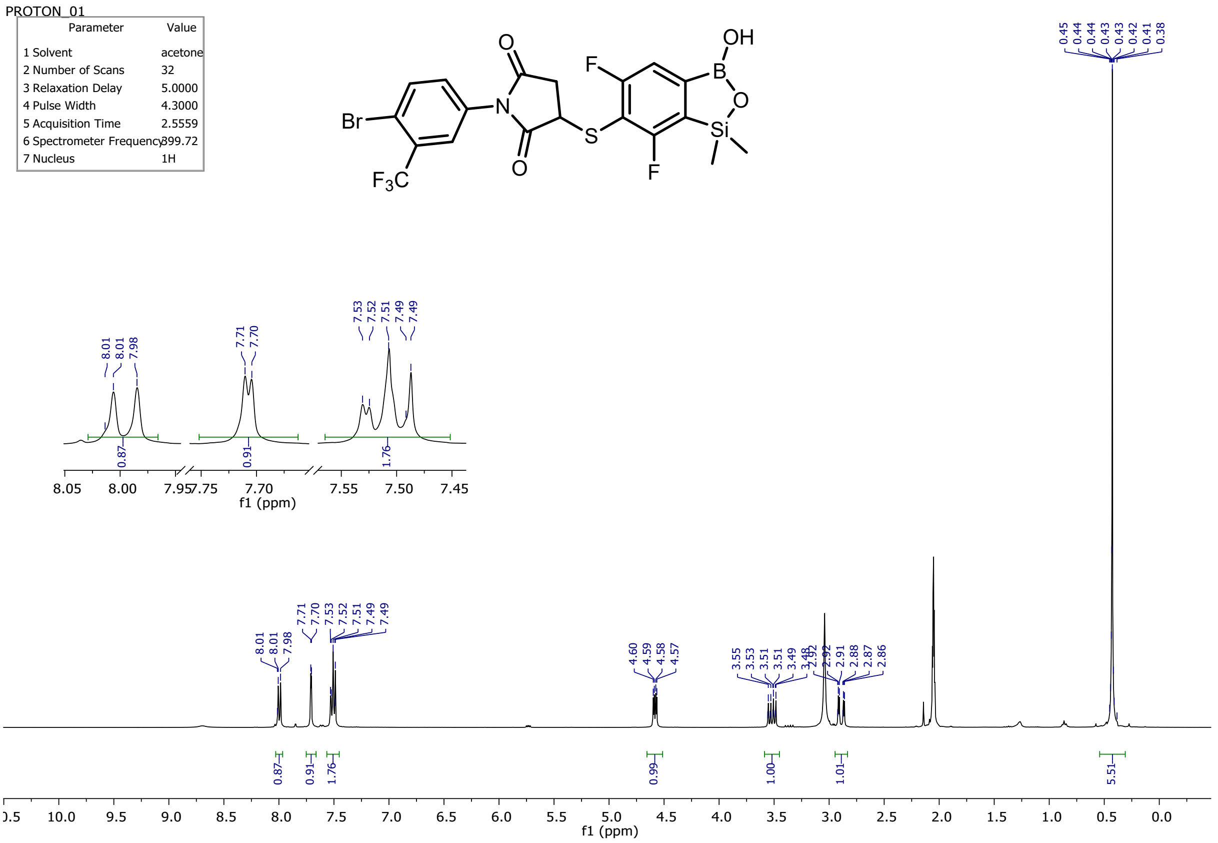

Figure S53. ^{1}H NMR spectrum (400 MHz, acetone-d_6) of **16**.

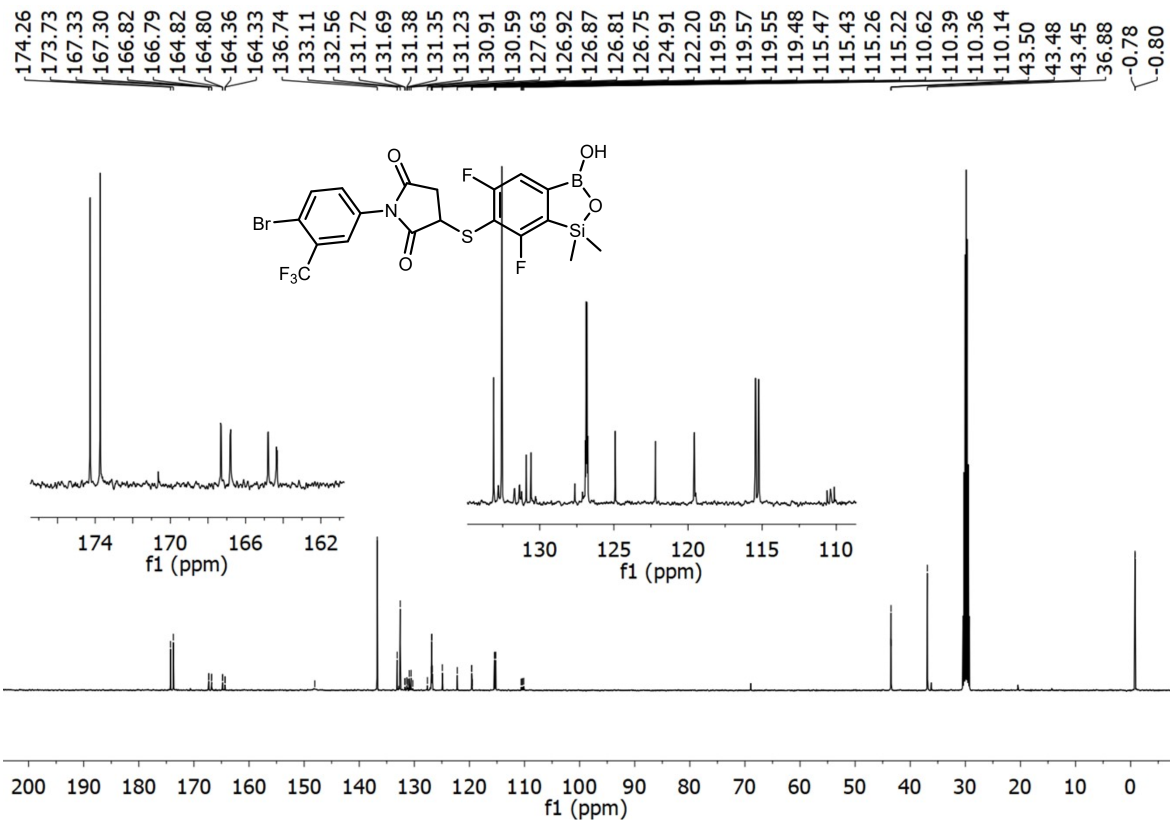

Figure S54. ^{13}C NMR spectrum (101 MHz, acetone-d_6) of **16**.

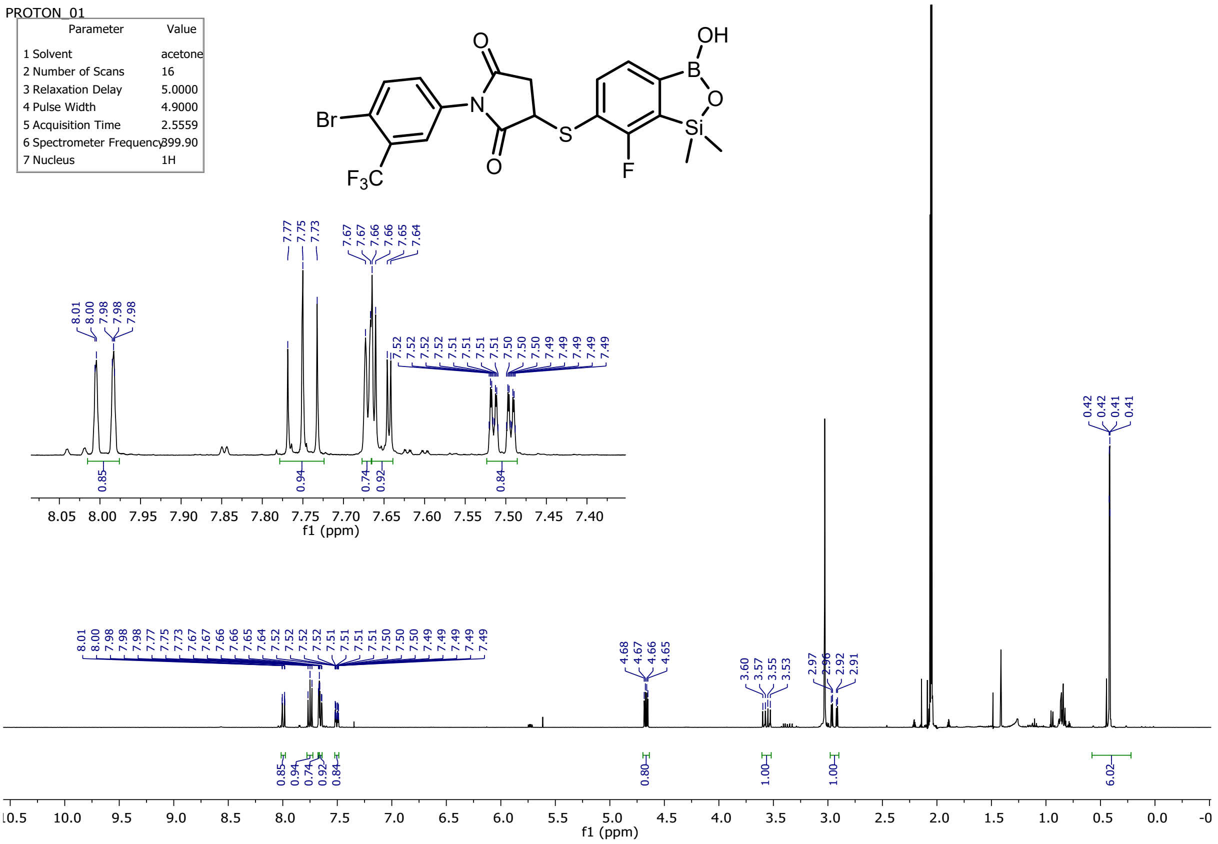

Figure S55. ^{1}H NMR spectrum (400 MHz, acetone-d_6) of **17**.

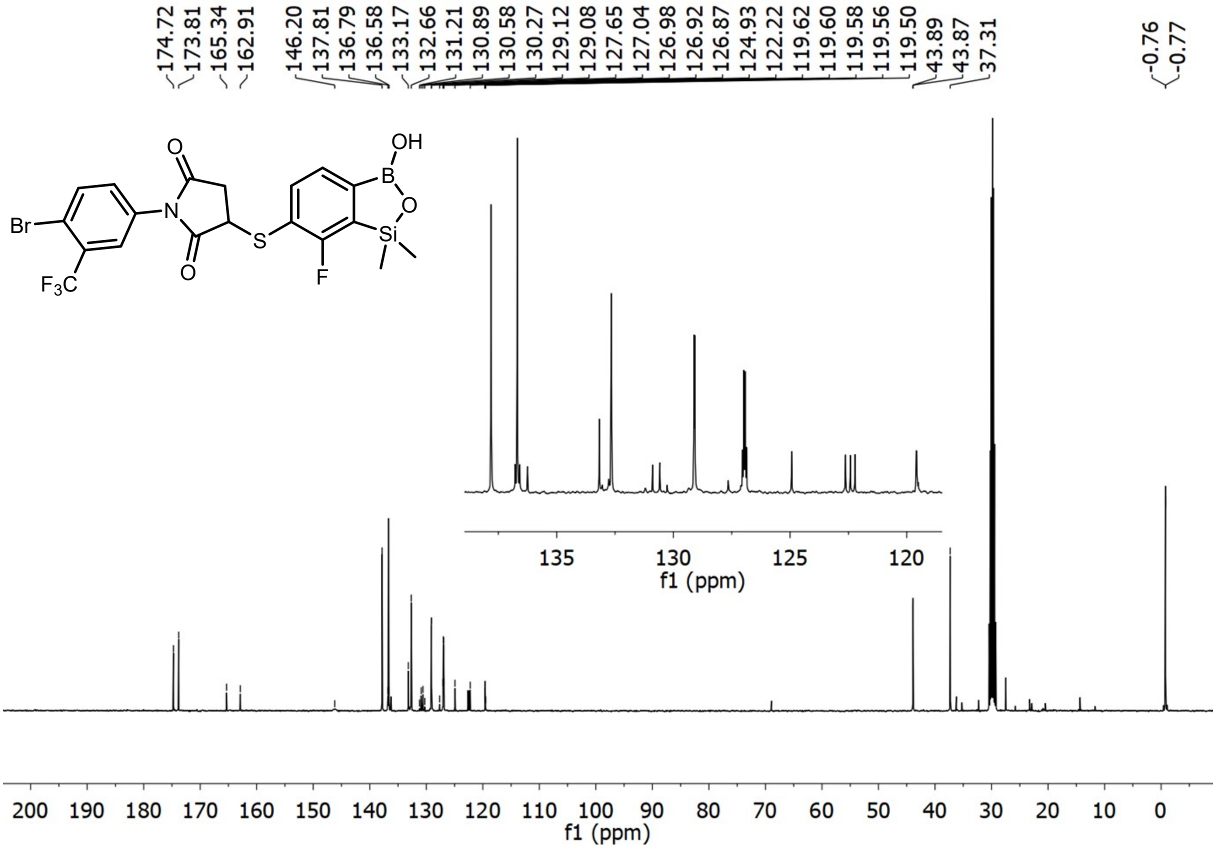

Figure S56. ^{13}C NMR (101 MHz, acetone-d_6) spectrum of **17**.

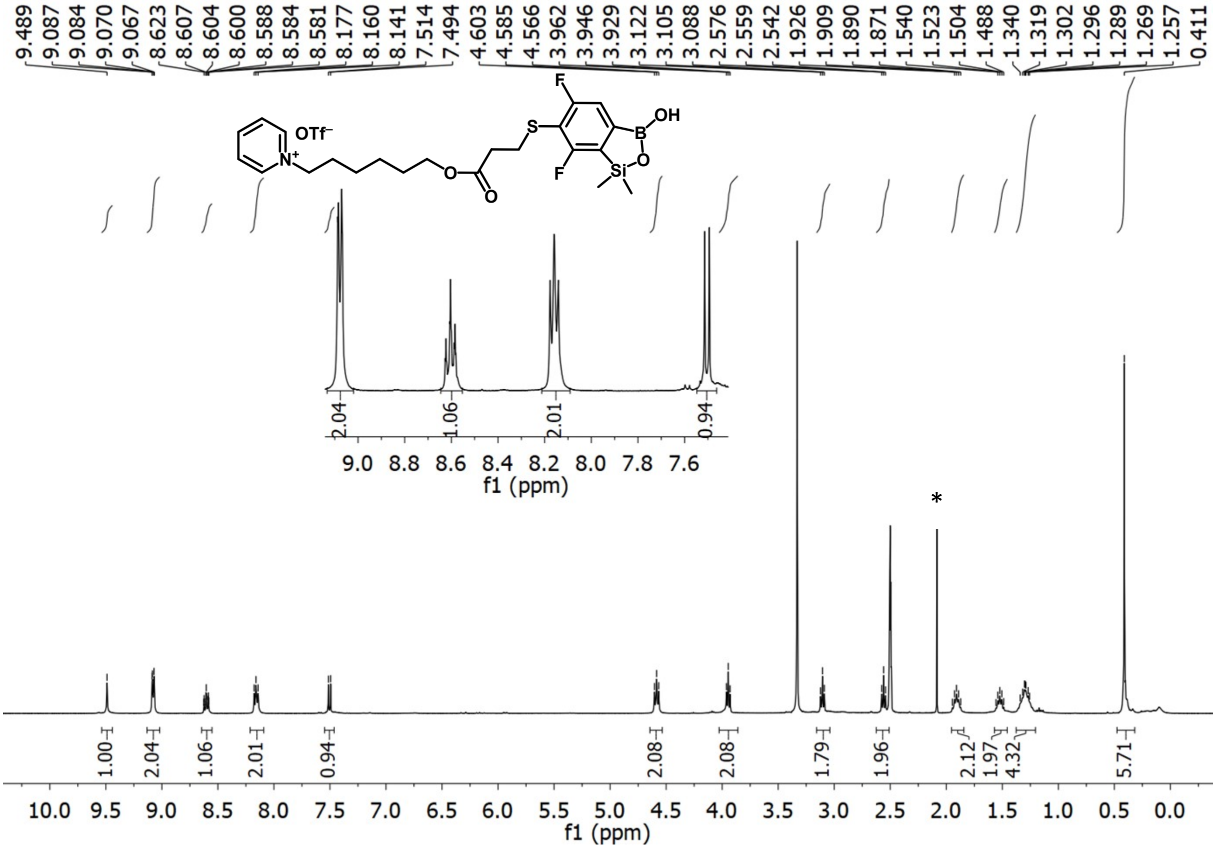

Figure S57. ^{1}H NMR spectrum (400 MHz, DMSO-d_6) of **18**. The signal of residual solvent (acetone) is marked with an asterisk.

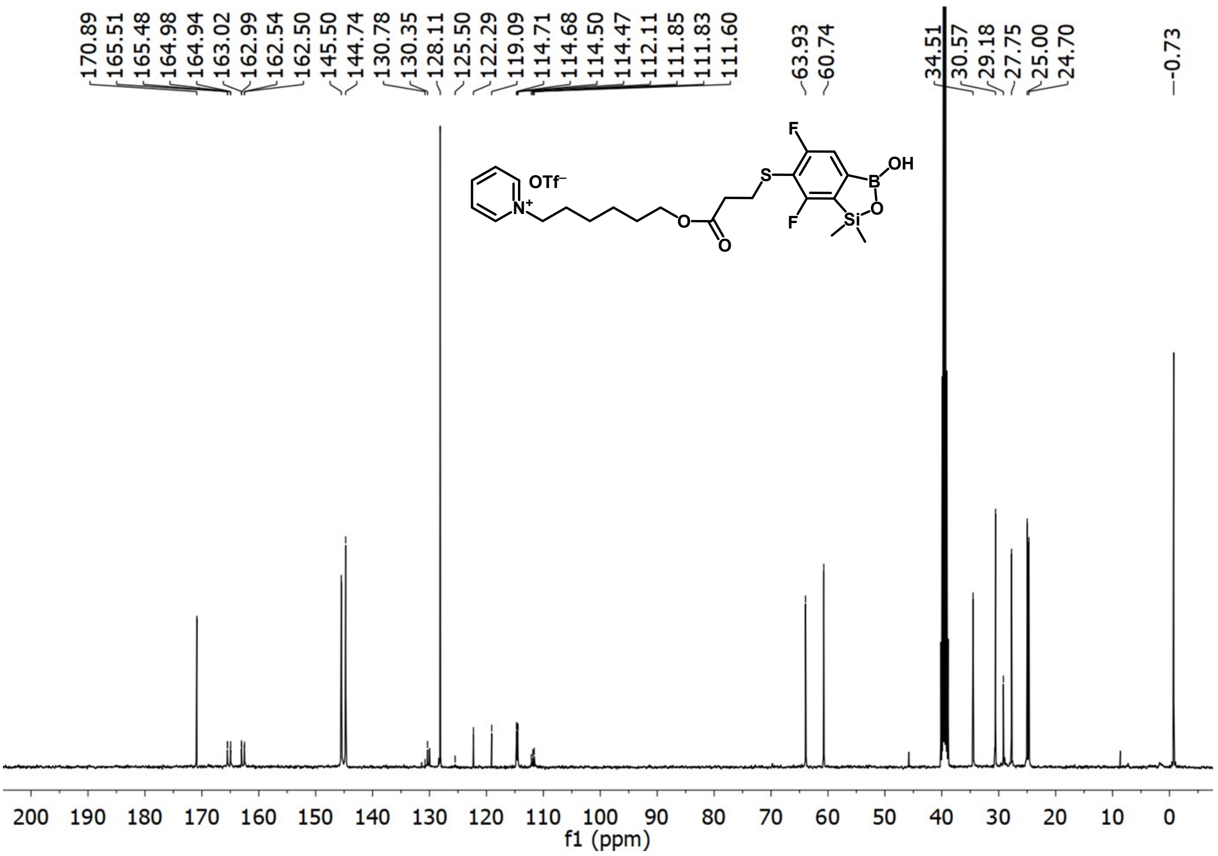

Figure S58. ^{13}C NMR spectrum (101 MHz, DMSO-d_6) of **18**.

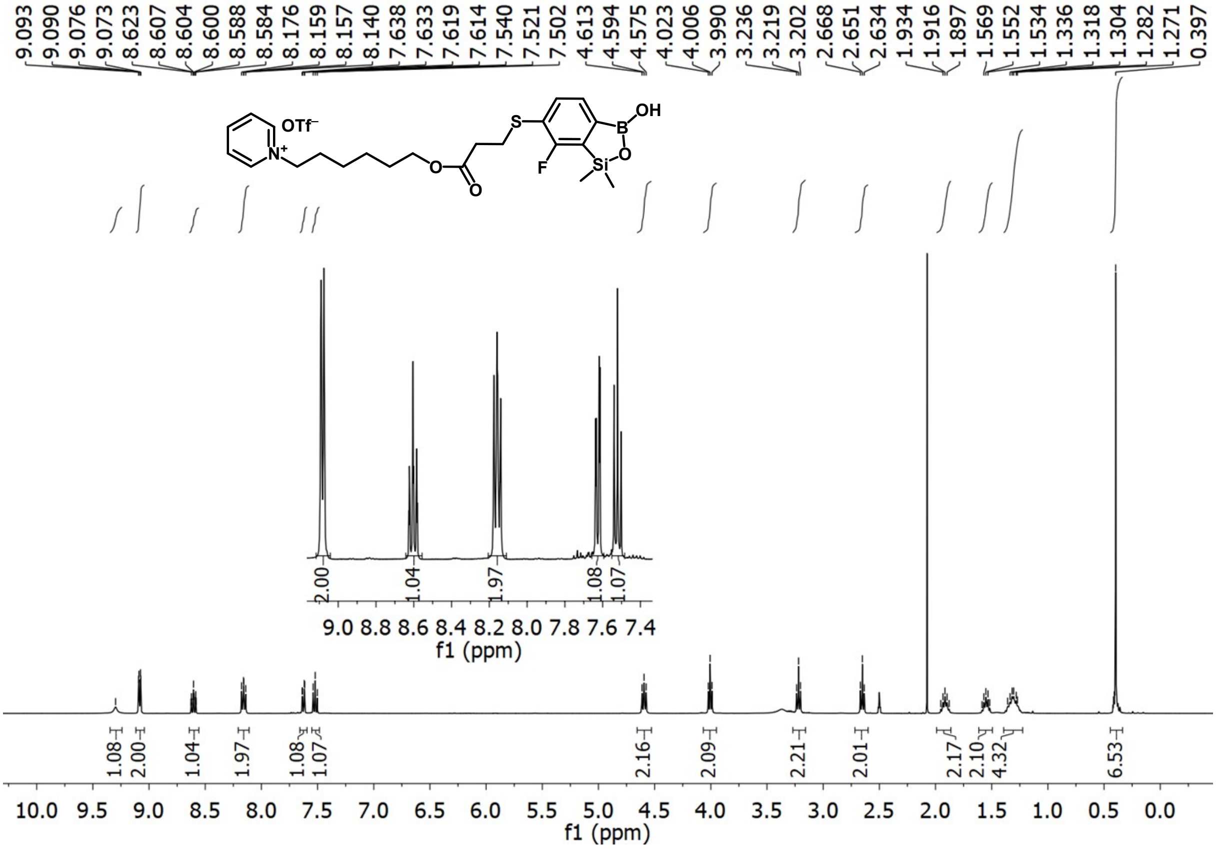

Figure S59. ^{1}H NMR spectrum (400 MHz, DMSO-d_6) of **19**. The signal of residual solvent (acetone) is marked with an asterisk.

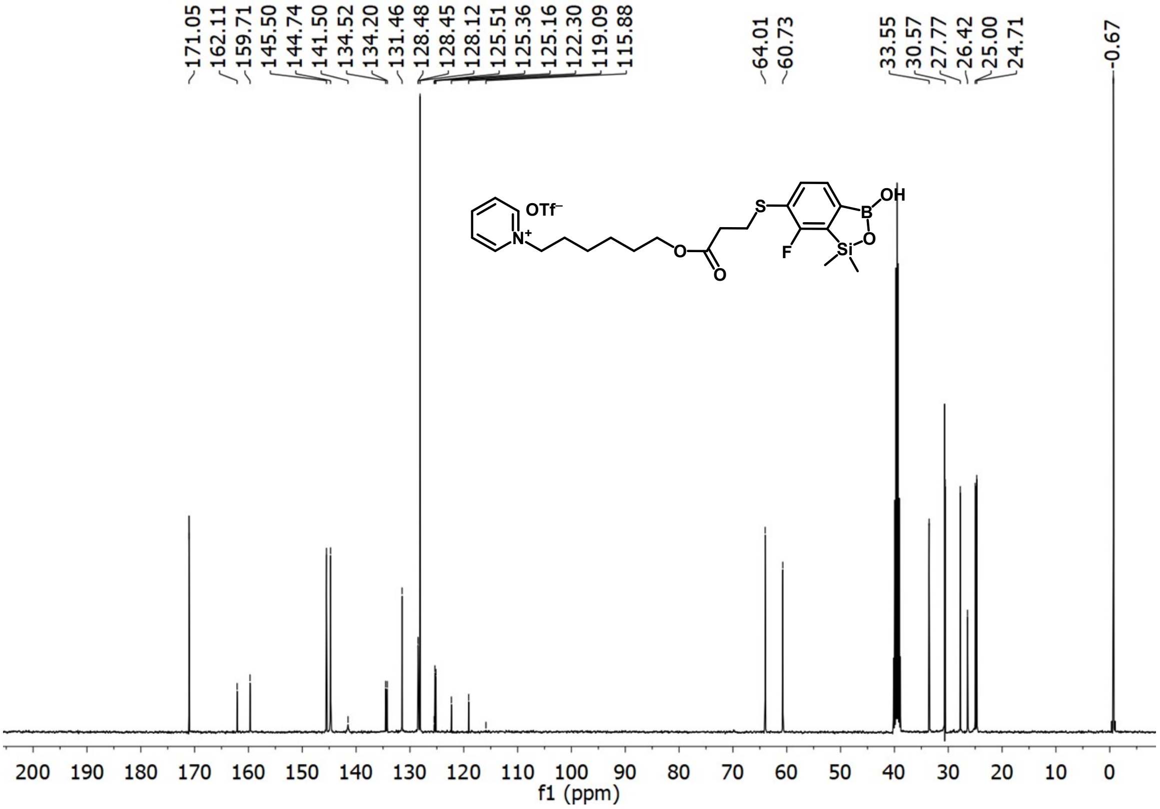

Figure S60. ^{13}C NMR spectrum (101 MHz, DMSO-d_6) of **19**.

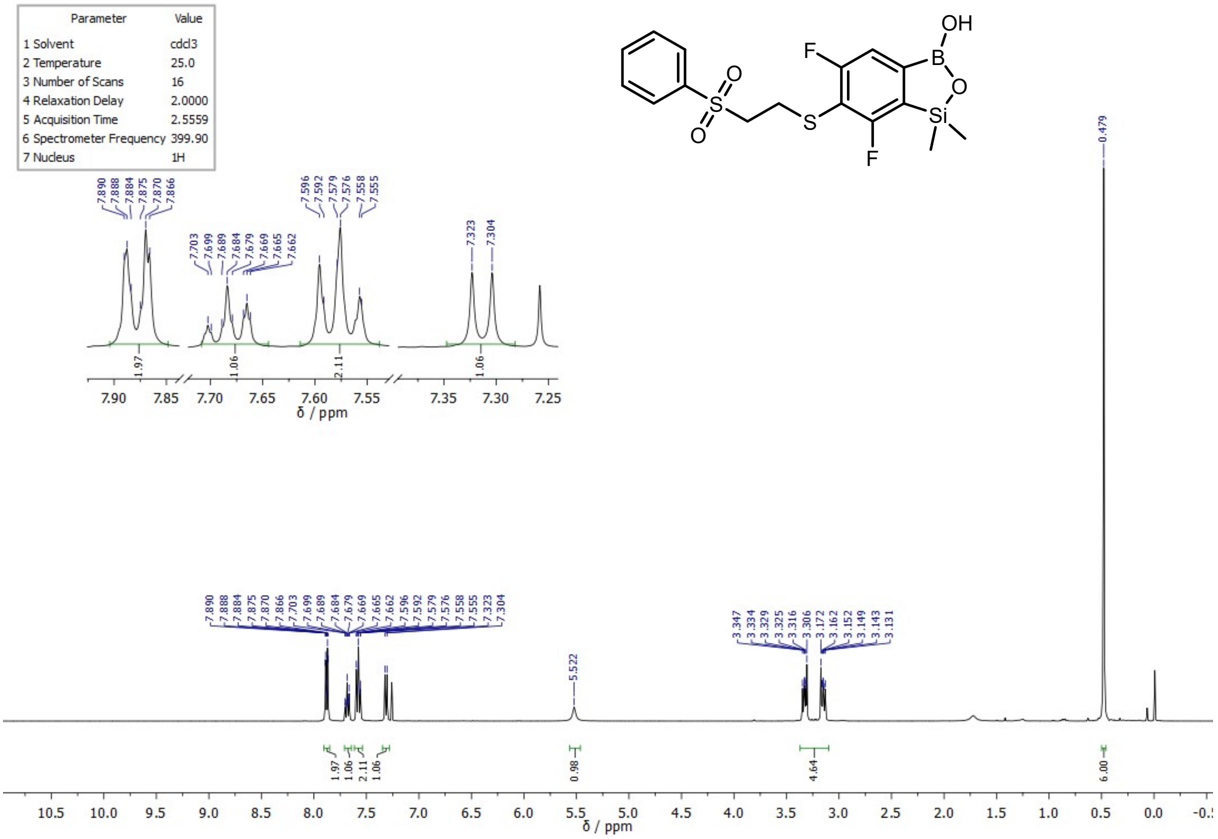

Figure S61. ^{1}H NMR spectrum (400 MHz, $CDCl_3$) of **20**.

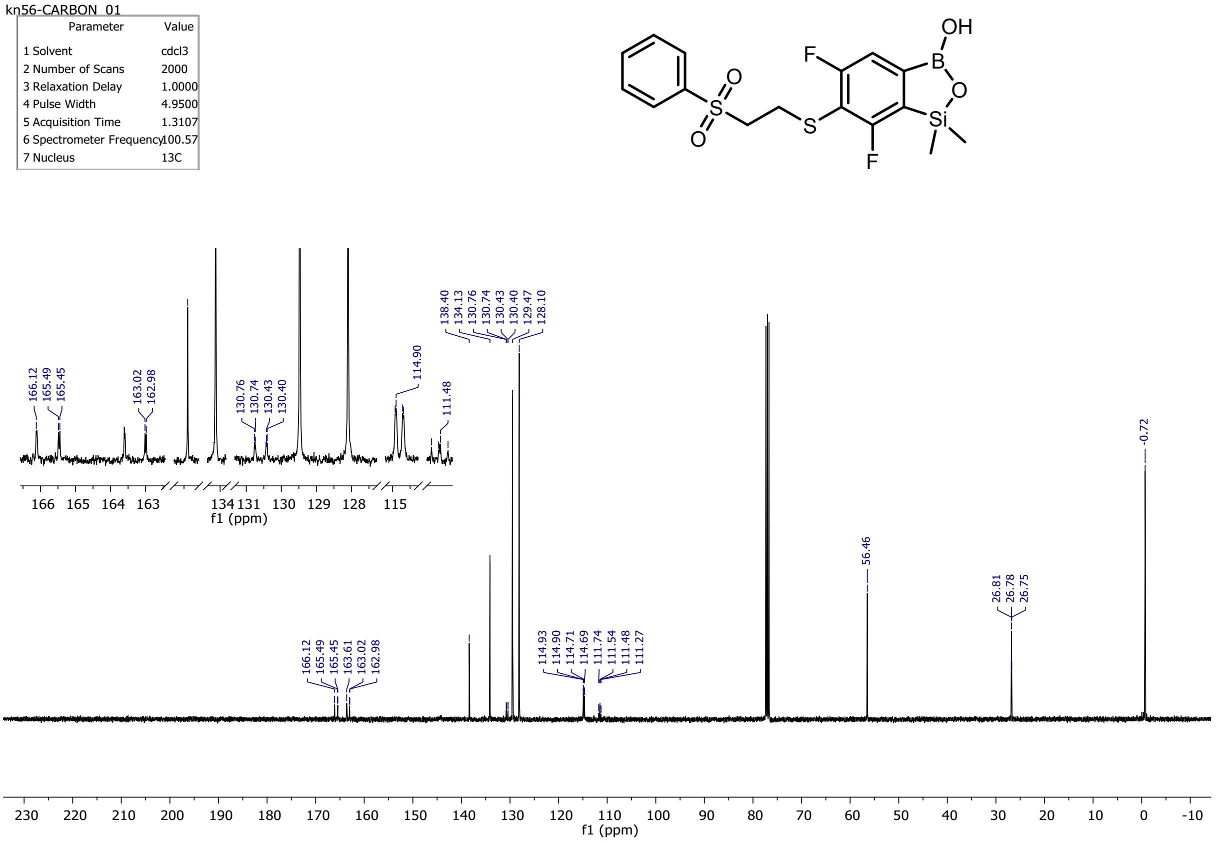

Figure S62. ^{13}C NMR spectrum (101 MHz, $CDCl_3$) of **20**.

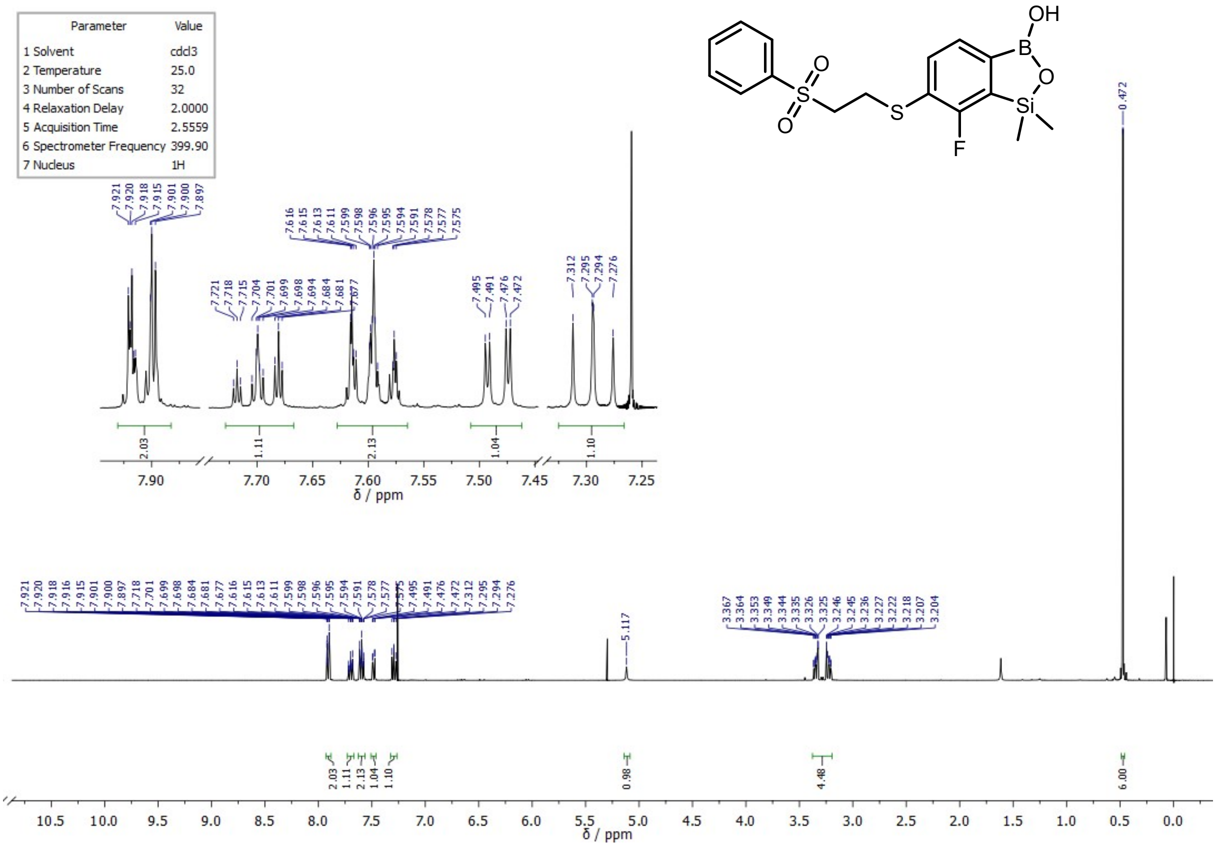

Figure S63. ^{1}H NMR spectrum (400 MHz, $CDCl_3$) of **21**.

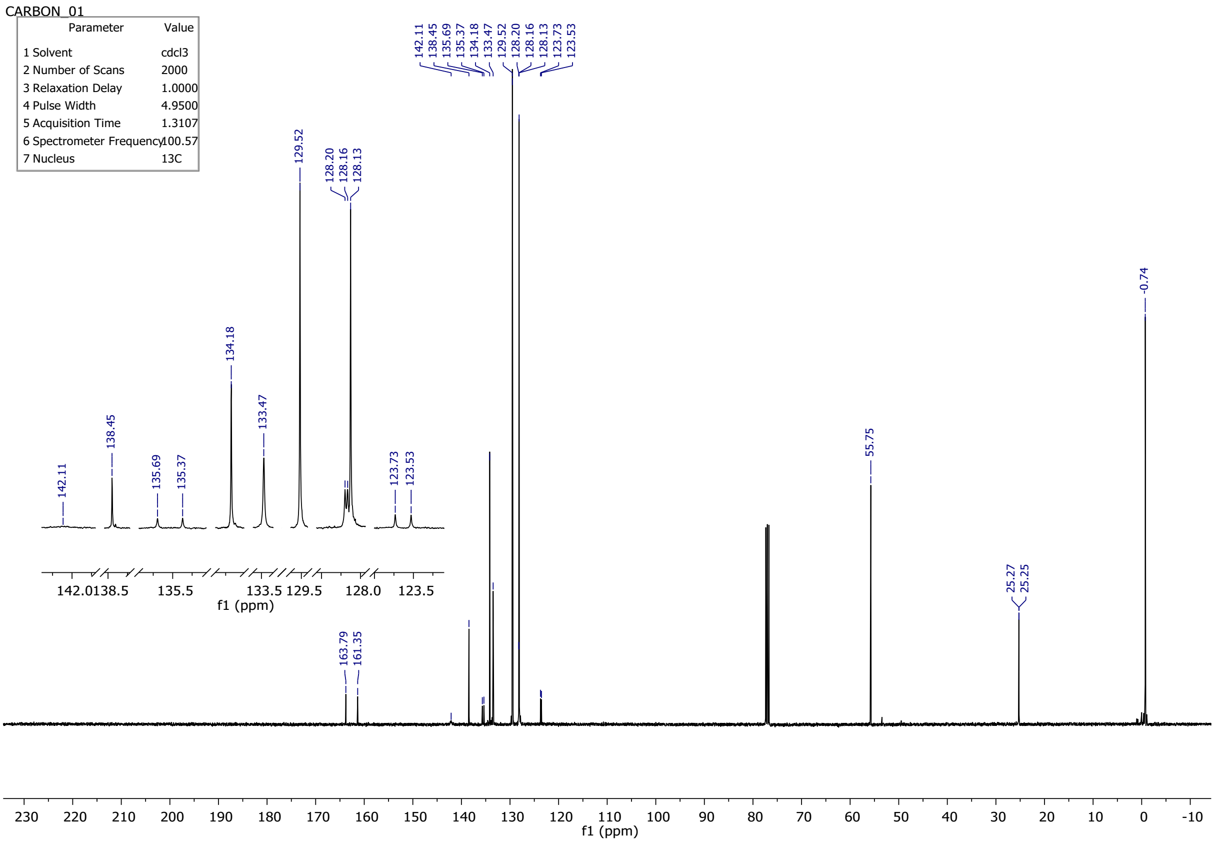

Figure S64. ^{13}C NMR spectrum (101 MHz, $CDCl_3$) of **21**.

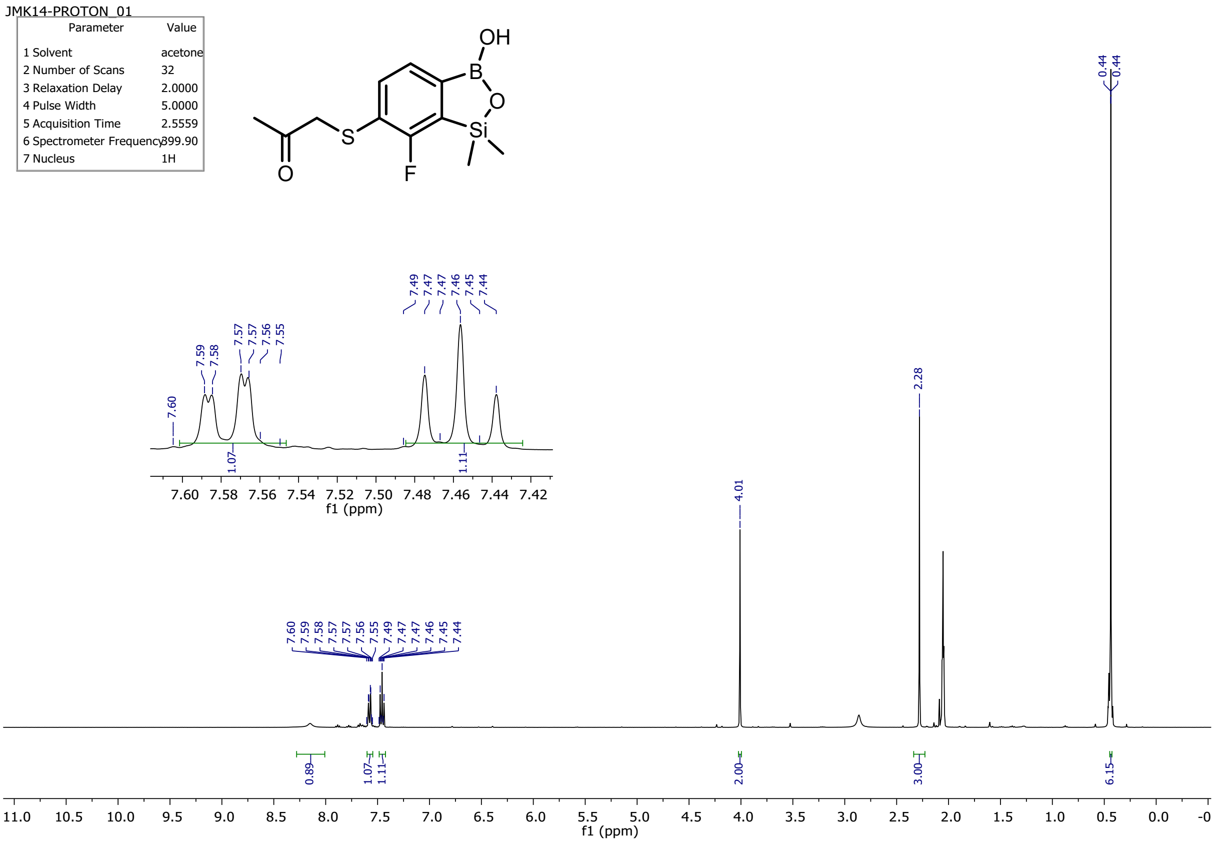

Figure S65. ^{1}H NMR spectrum (400 MHz, acetone-d_6) of **22**.

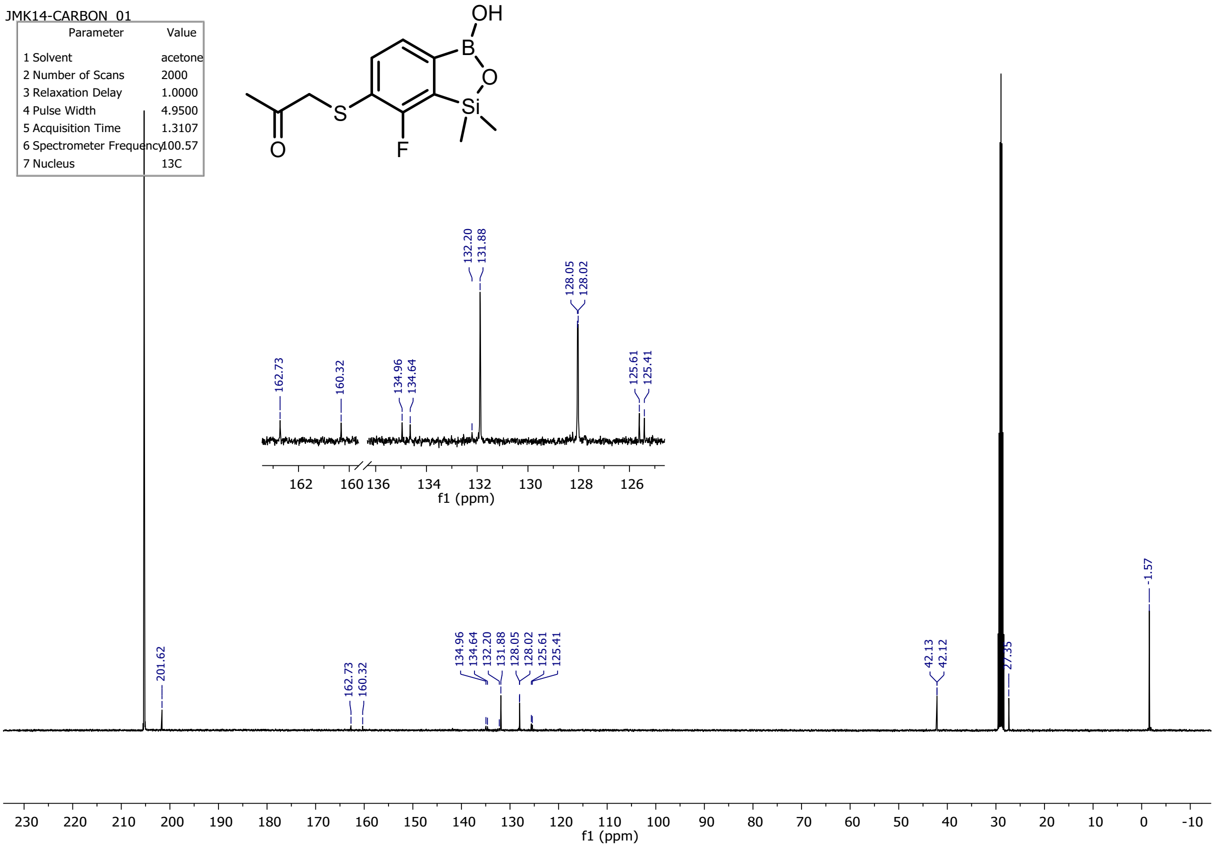

Figure S66. ^{13}C NMR spectrum (101 MHz, acetone-d_6) of **22** in acetone-d_6.

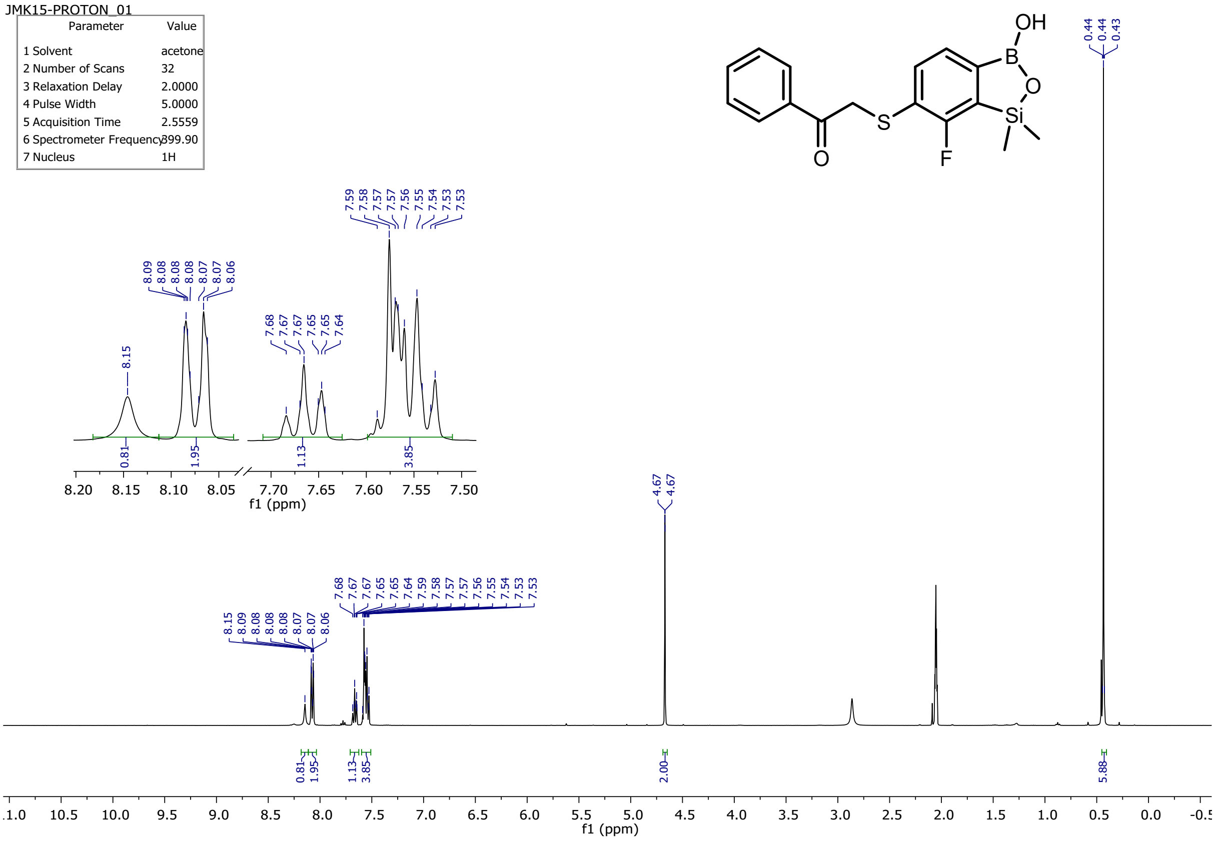

Figure S67. ^{1}H NMR spectrum (400 MHz, acetone-d_6) of **23**.

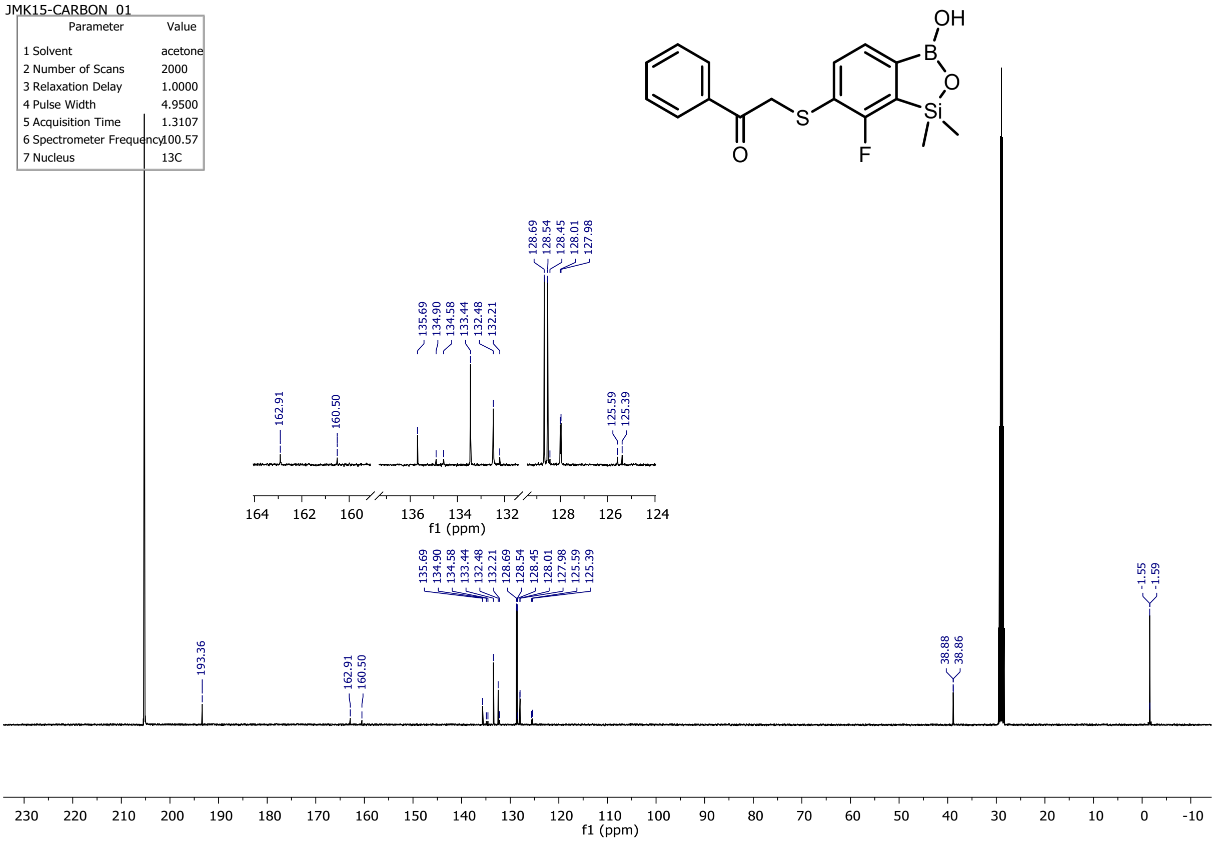

Figure S68. ^{13}C NMR spectrum (101 MHz, acetone-d_6) of **23**.

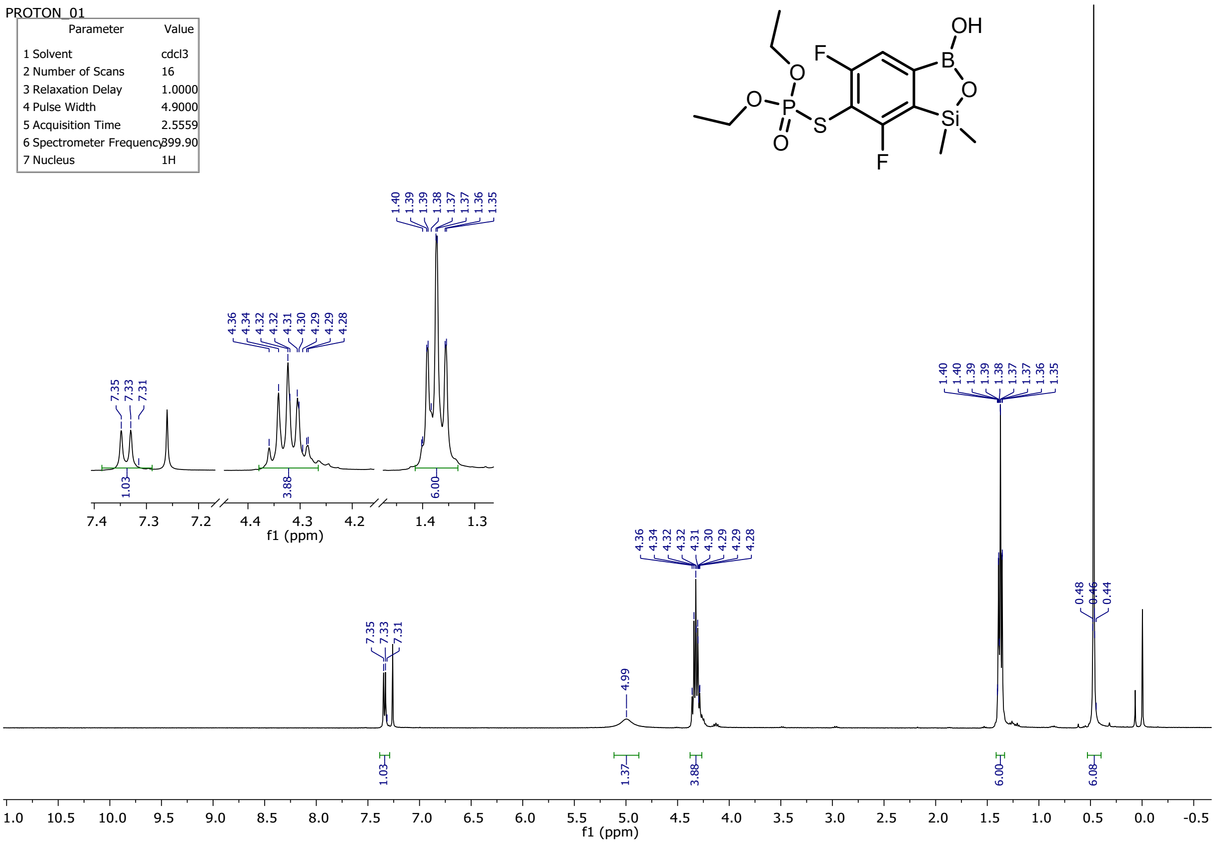

Figure S69. ^{1}H NMR spectrum (400 MHz, $CDCl_3$) of **24**.

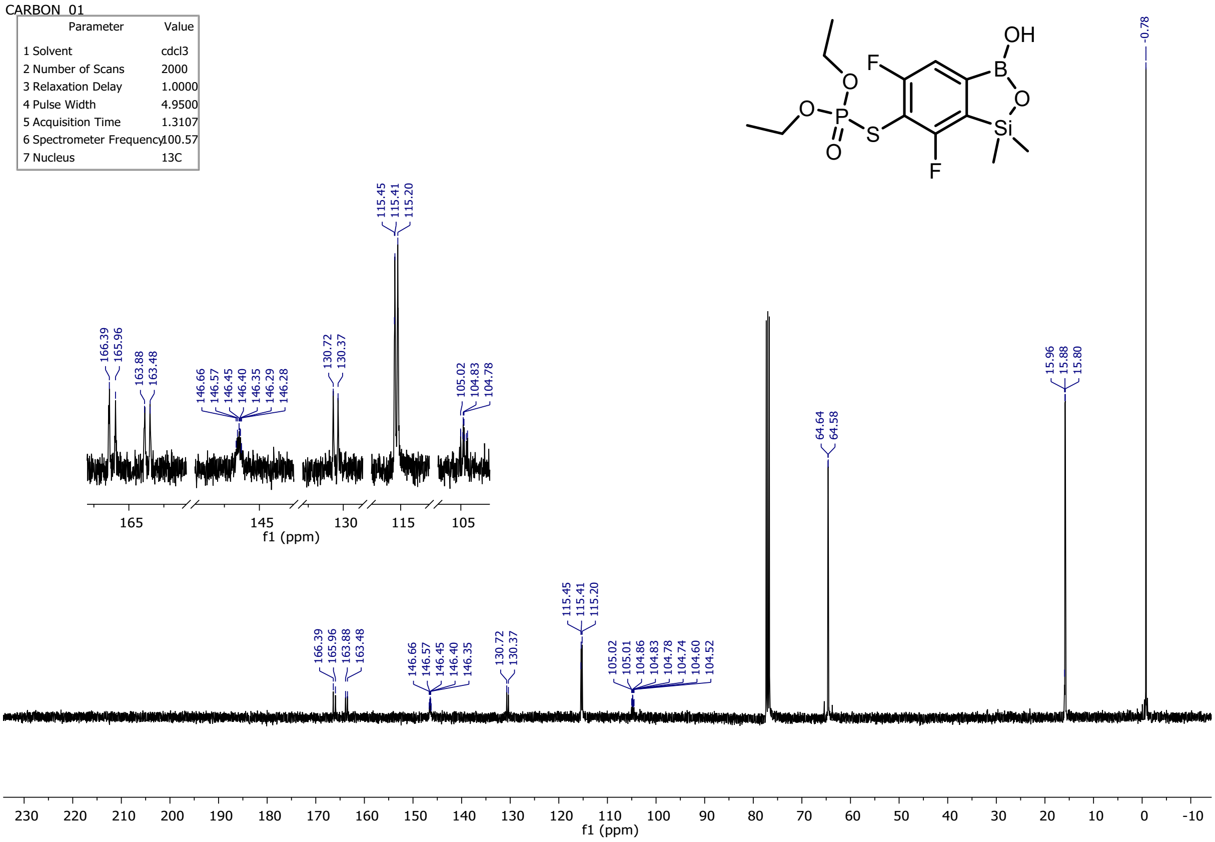

Figure S70. ^{13}C NMR spectrum (101 MHz, $CDCl_3$) of **24**.

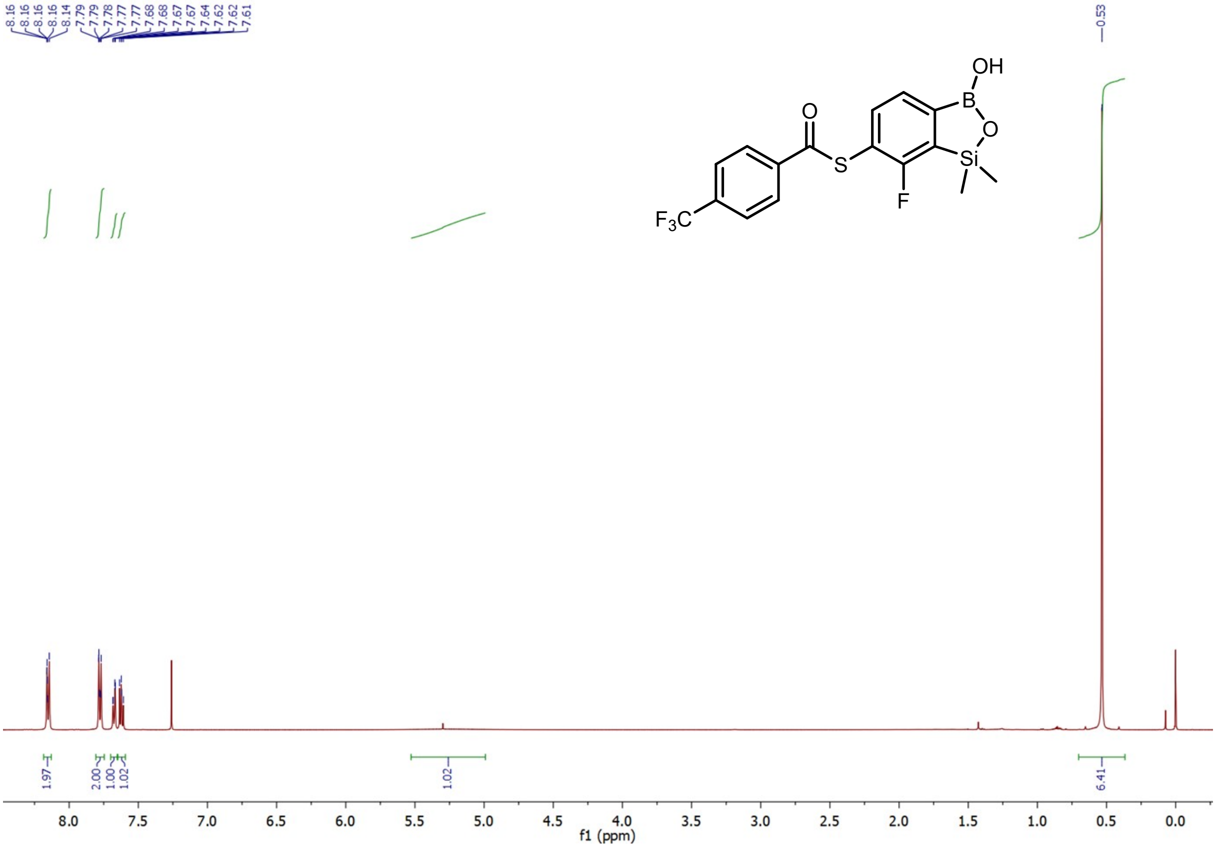

Figure S71. ^{1}H NMR spectrum (500 MHz, $CDCl_3$) of **25**.

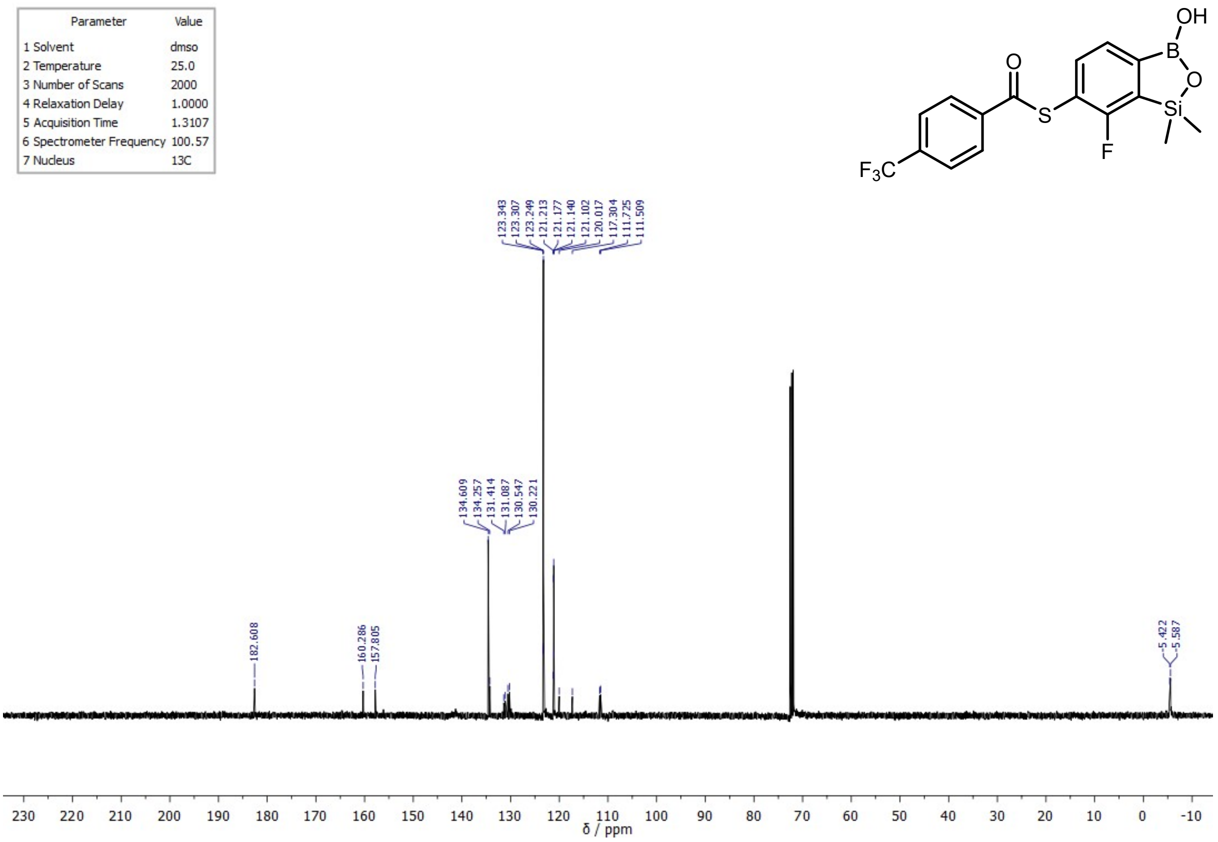

Figure S72. ^{13}C NMR (101 MHz, DMSO-d_6) spectrum of **25**.

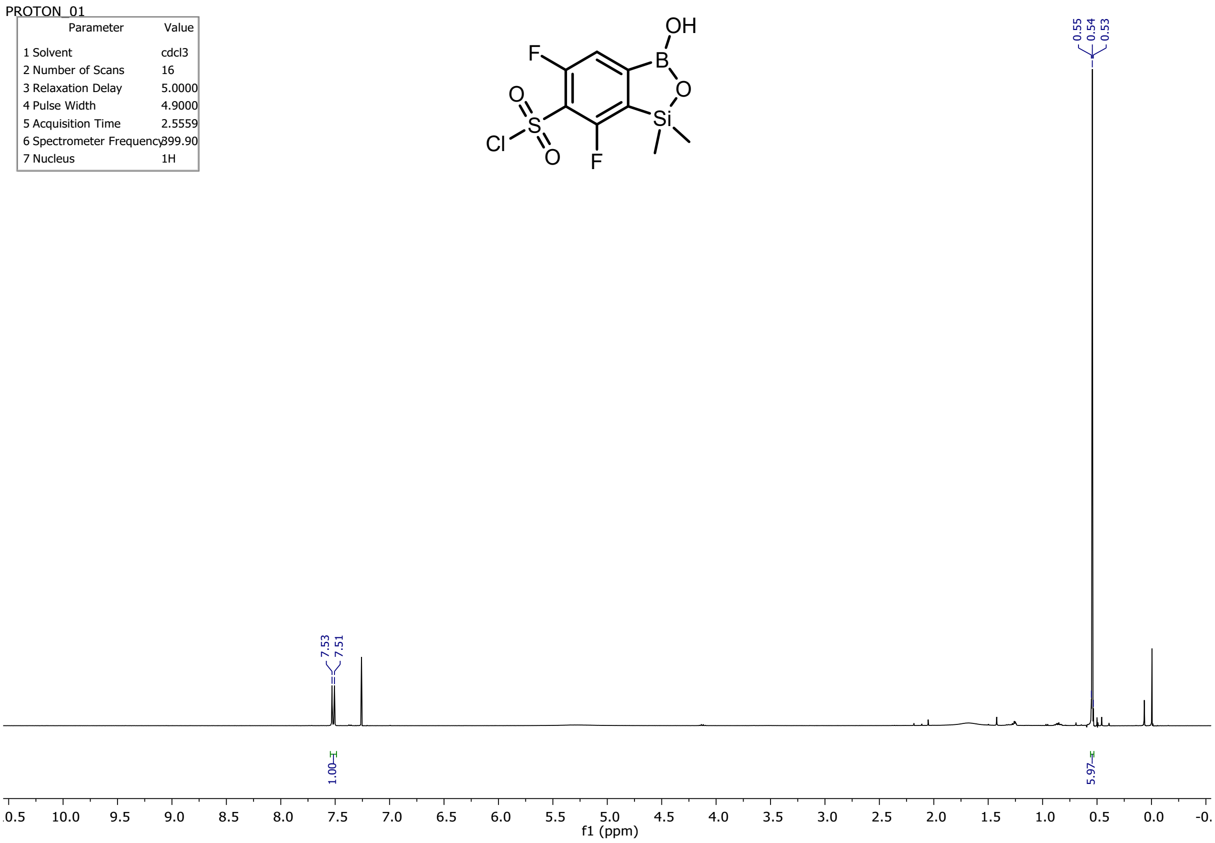

Figure S73. ^{1}H NMR spectrum (400 MHz, $CDCl_3$) of **26**.

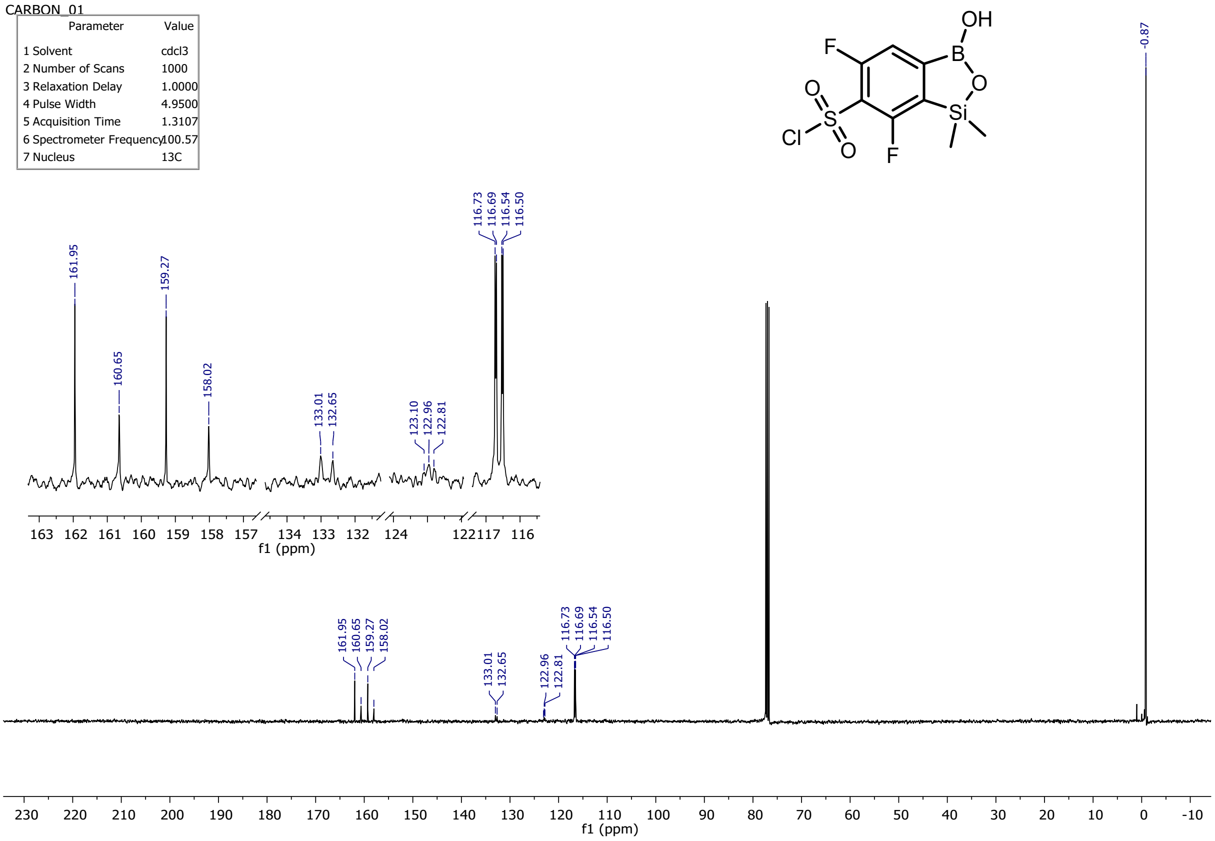

Figure S74. ^{13}C NMR spectrum (101 MHz, $CDCl_3$) of **26**.

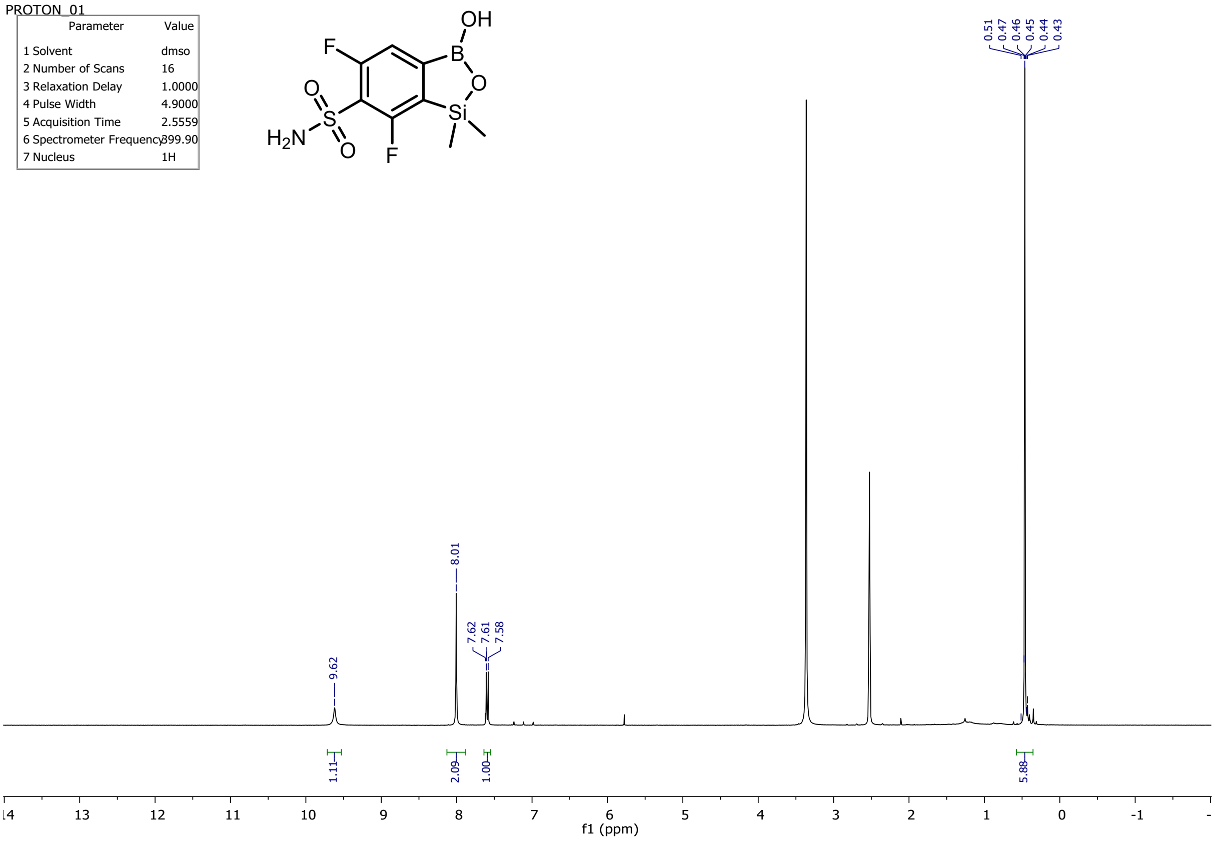

Figure S75. ^{1}H NMR spectrum (400 MHz, DMSO-d_6) of **27**.

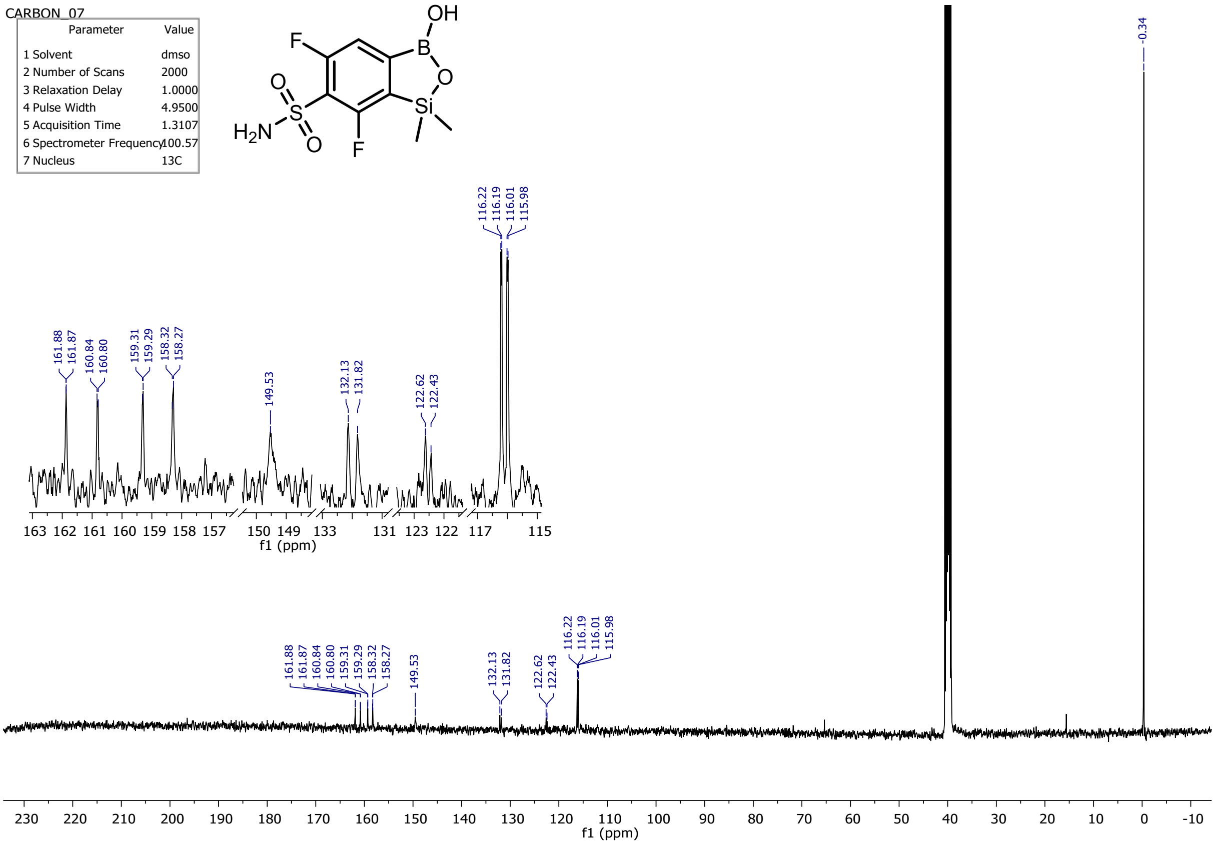

Figure S76. ^{13}C NMR spectrum (400 MHz, DMSO-d_6) of **27**.

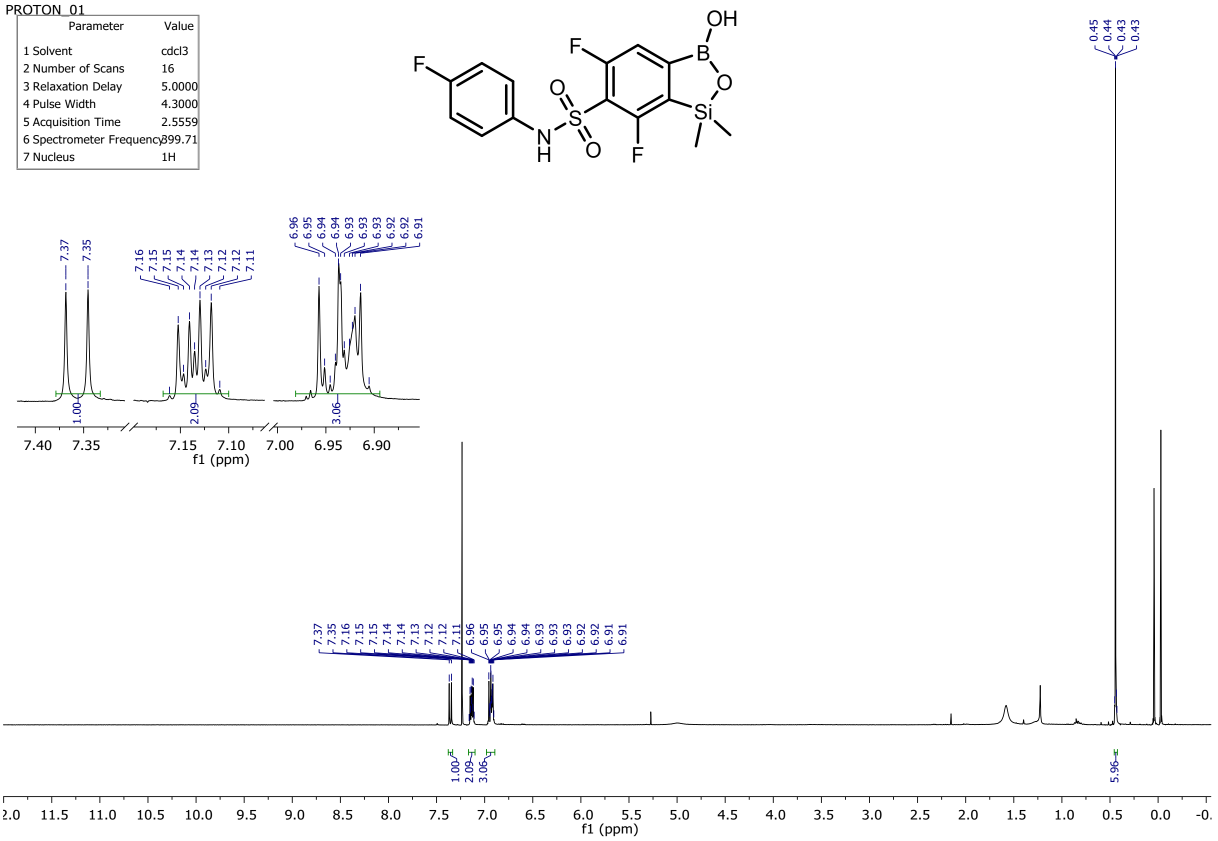

Figure S77. ^{1}H NMR spectrum (400 MHz, $CDCl_3$) of **28**.

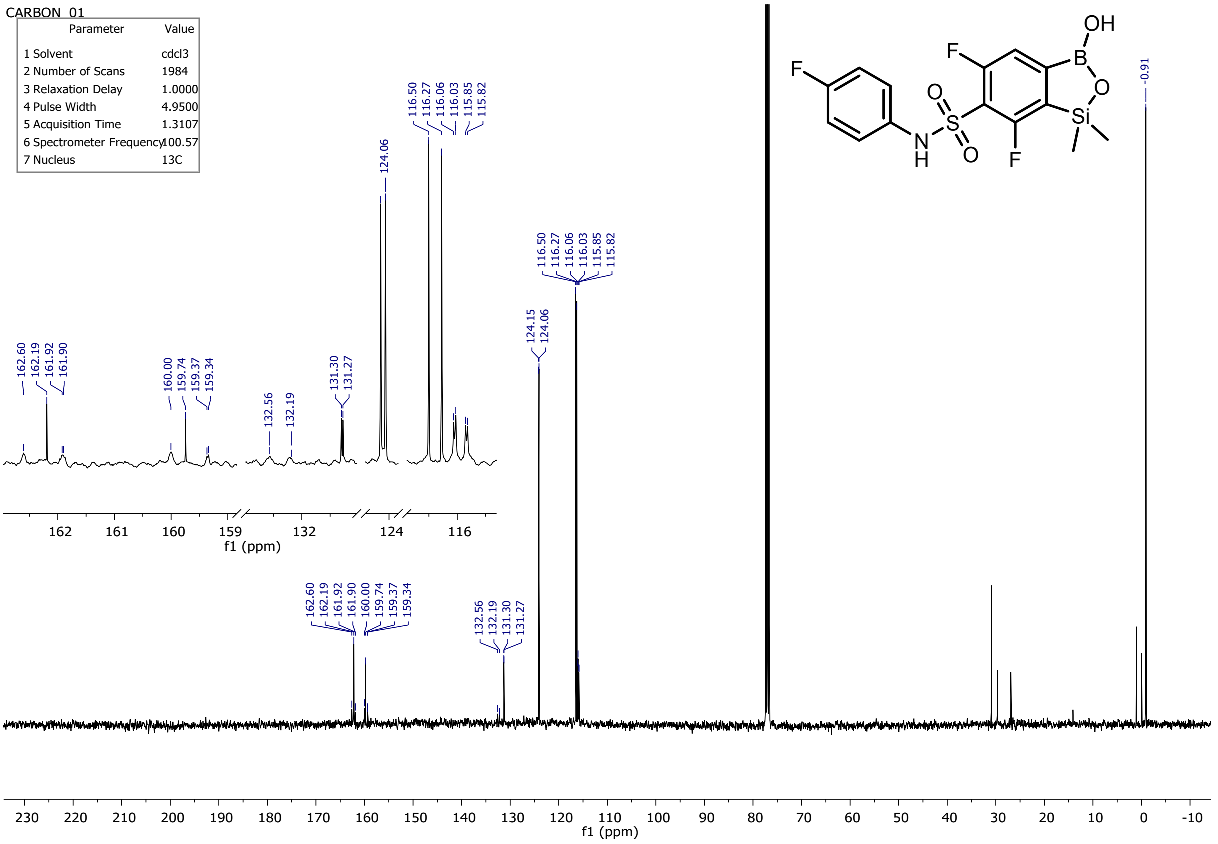

Figure S78. ^{13}C NMR spectrum (101 MHz, $CDCl_3$) of **28**.

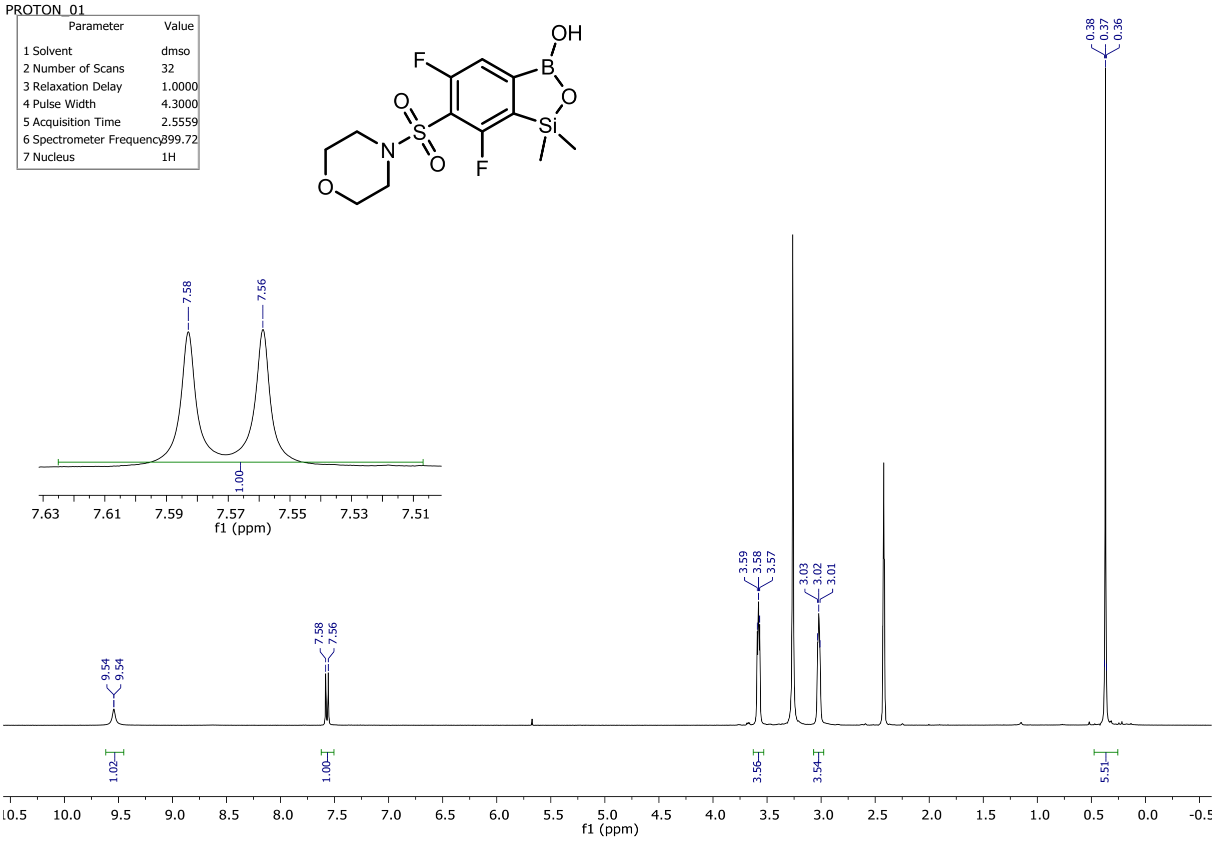

Figure S79. ^{1}H NMR spectrum (400 MHz, DMSO-d_6) of **29**.

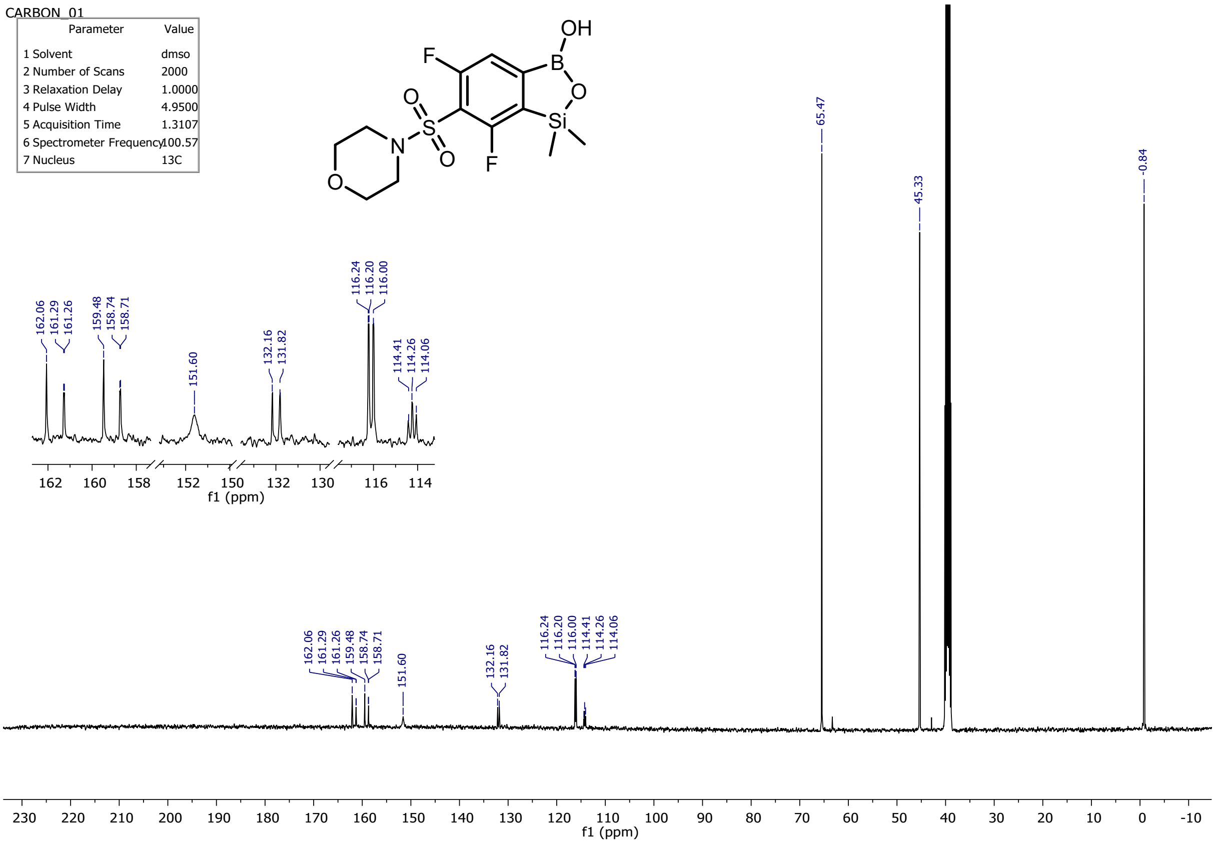

Figure S80. ^{13}C NMR spectrum (101 MHz, DMSO-d_6) of **29**.

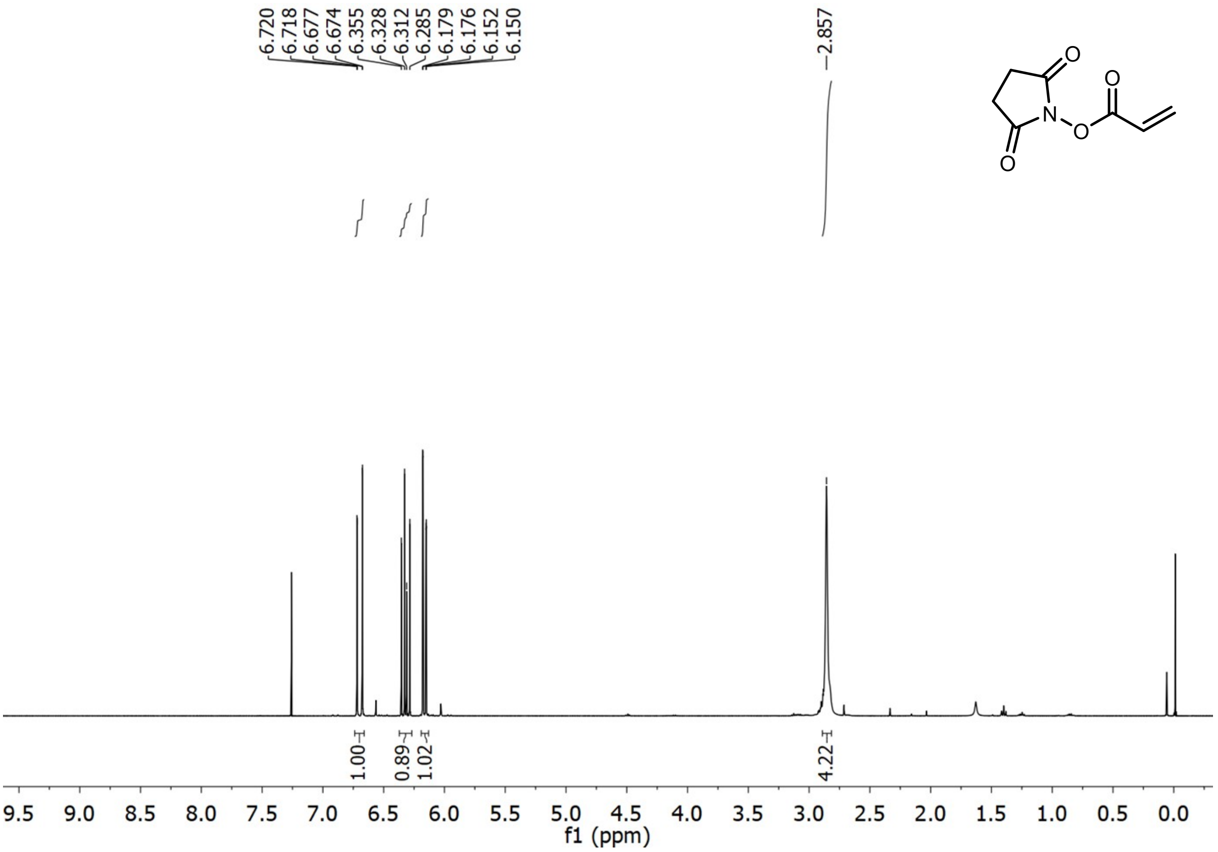

Figure S81. ^{1}H NMR spectrum (400 MHz, $CDCl_3$) of **S1**.

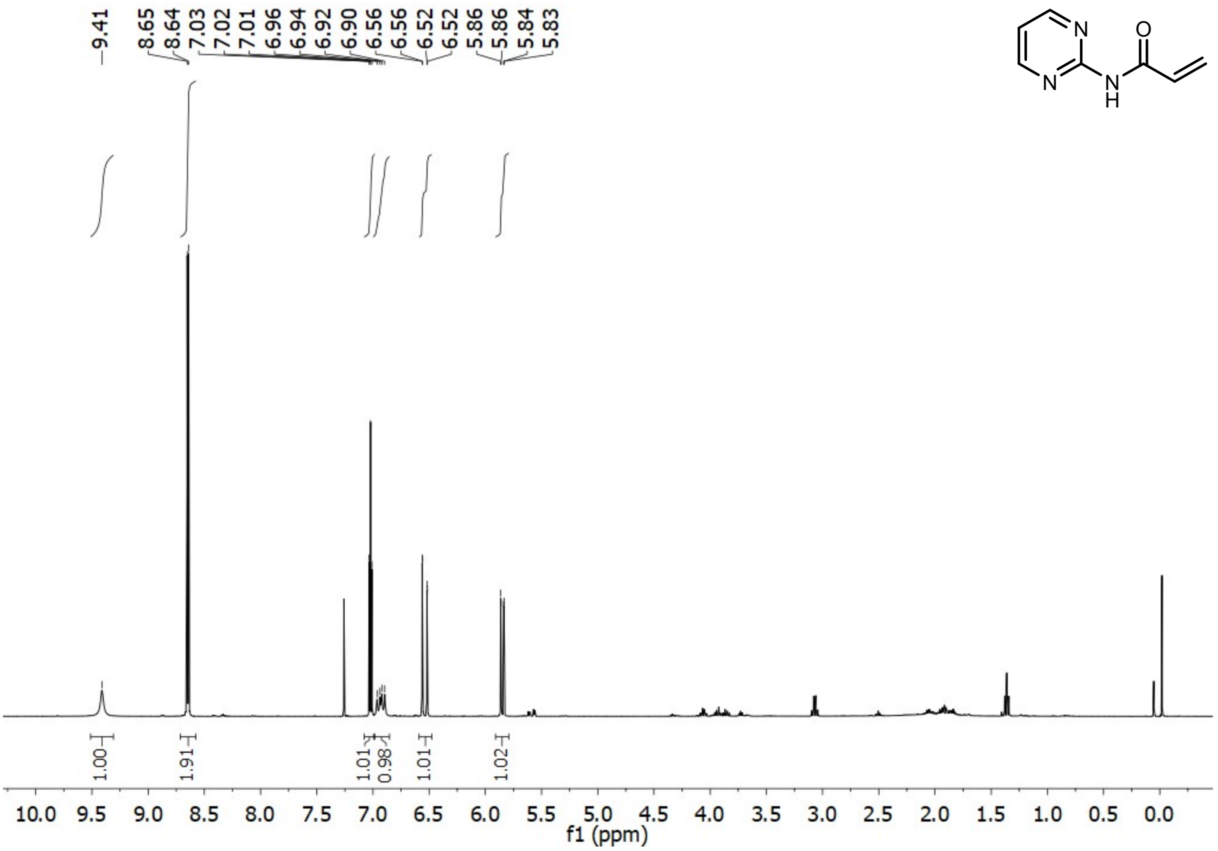

Figure S82. ^{1}H NMR spectrum (400 MHz, $CDCl_3$) of **S2**.

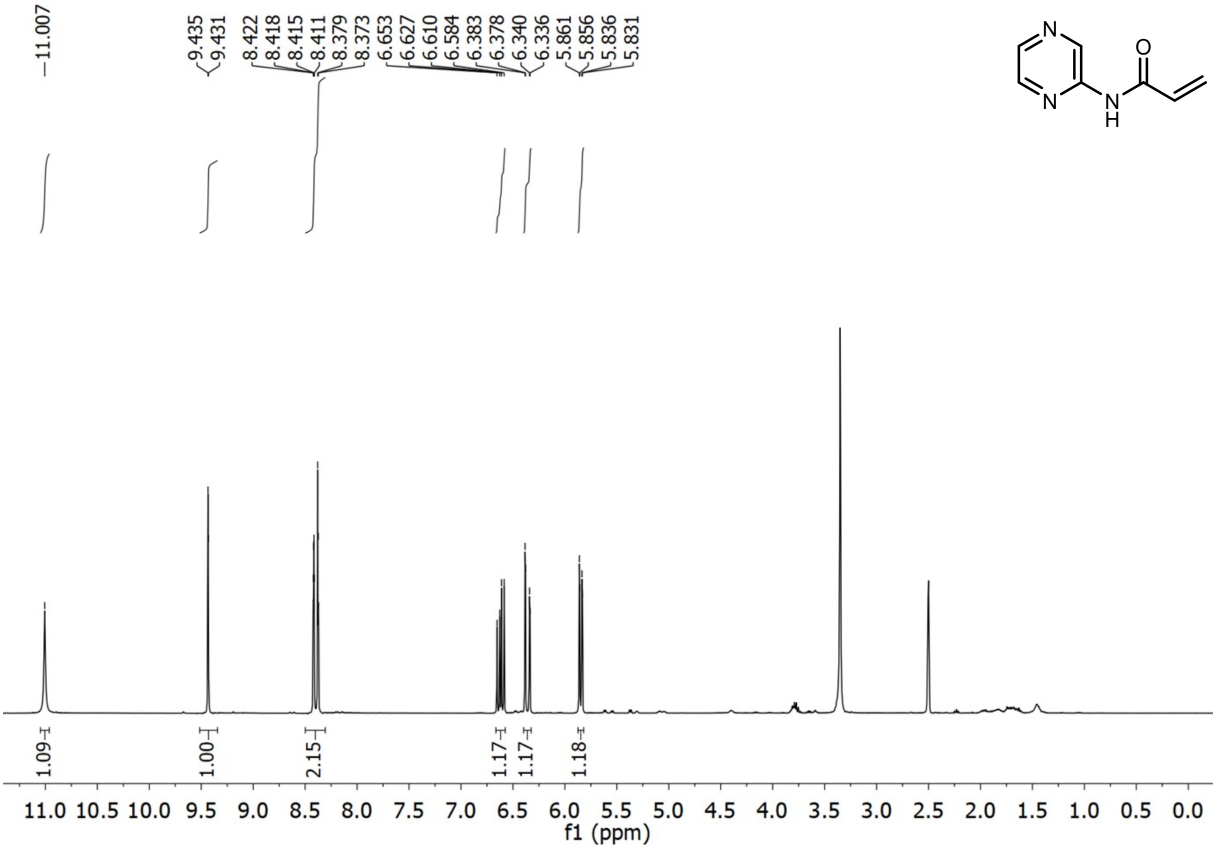

Figure S83. ^{1}H NMR spectrum (400 MHz, DMSO-d_6) of **S3**.

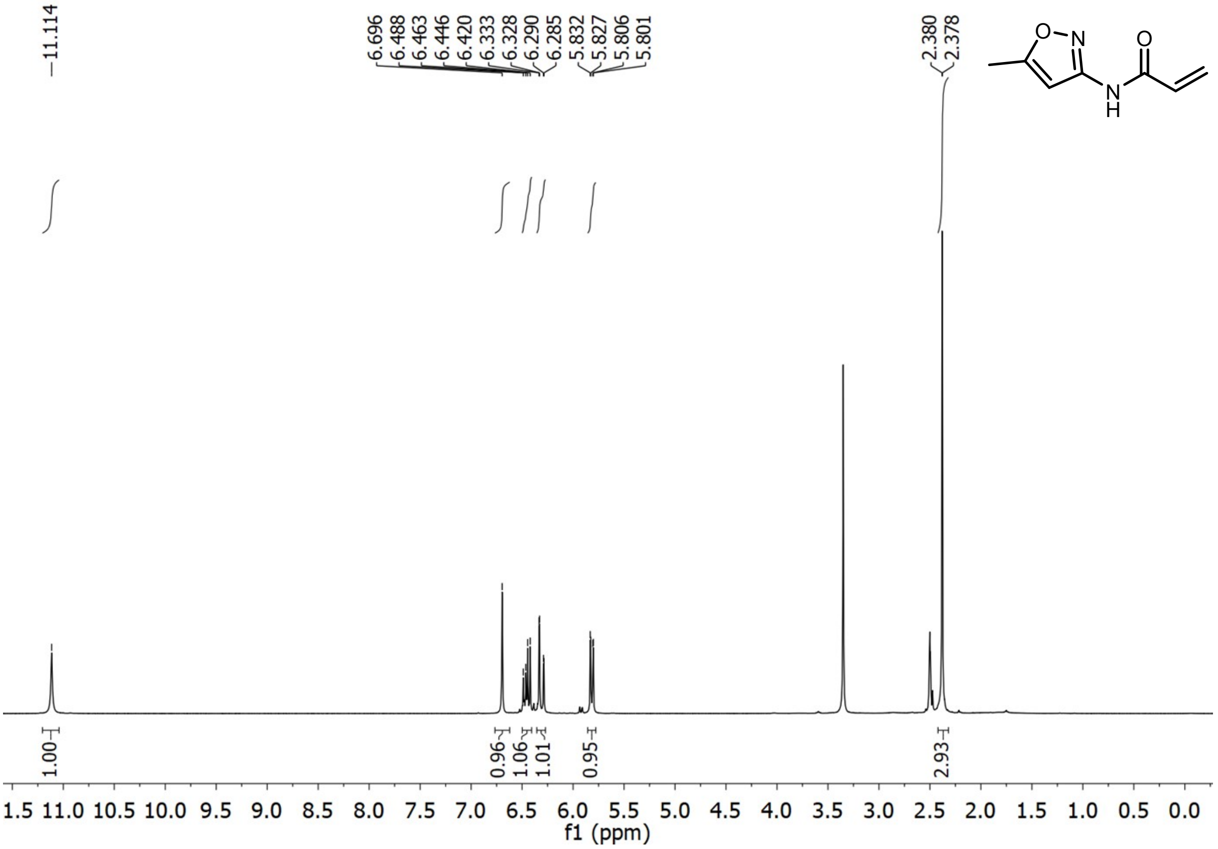

Figure S84. ^{1}H NMR spectrum (400 MHz, DMSO-d_6) of **S4**.

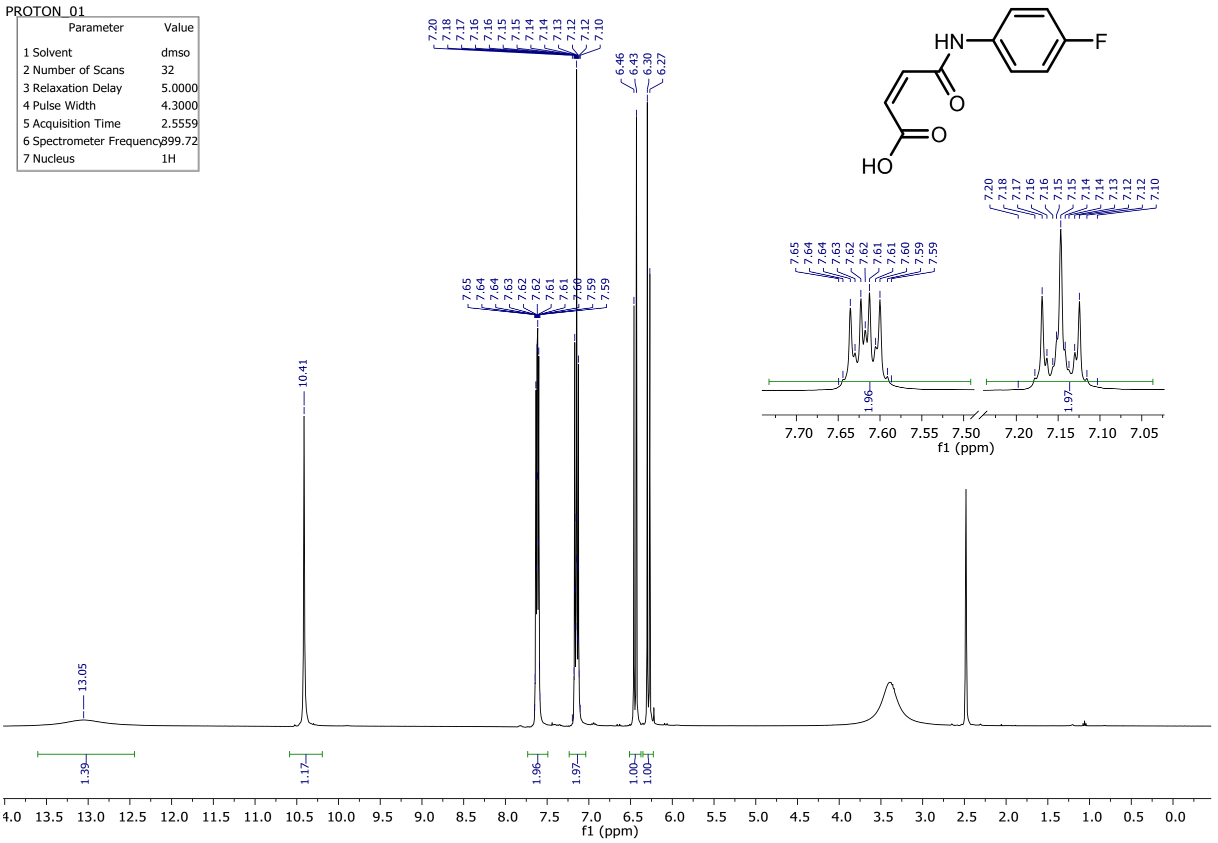

Figure S85. ^{1}H NMR spectrum (400 MHz, DMSO-d_6) of **S5**.

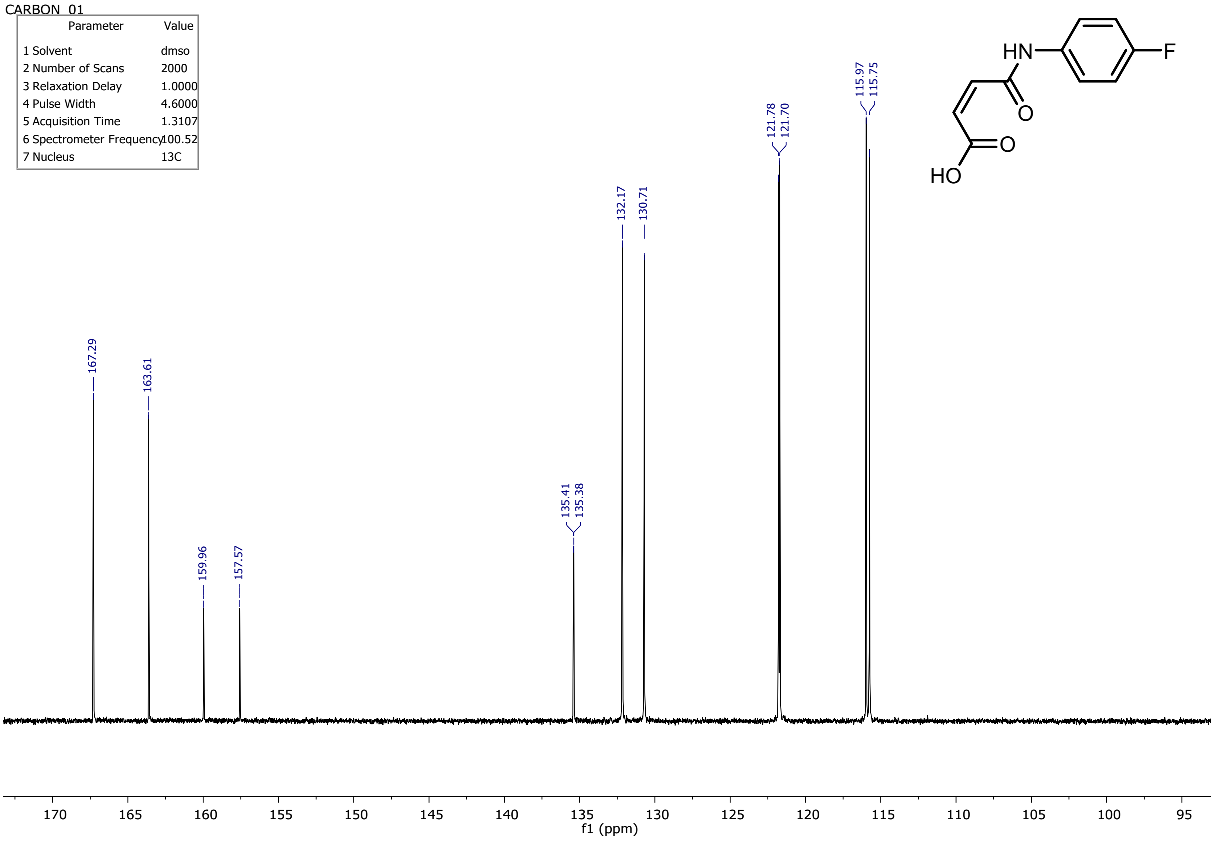

Figure S86. ^{13}C NMR spectrum (101 MHz, DMSO-d_6) of **S5**.

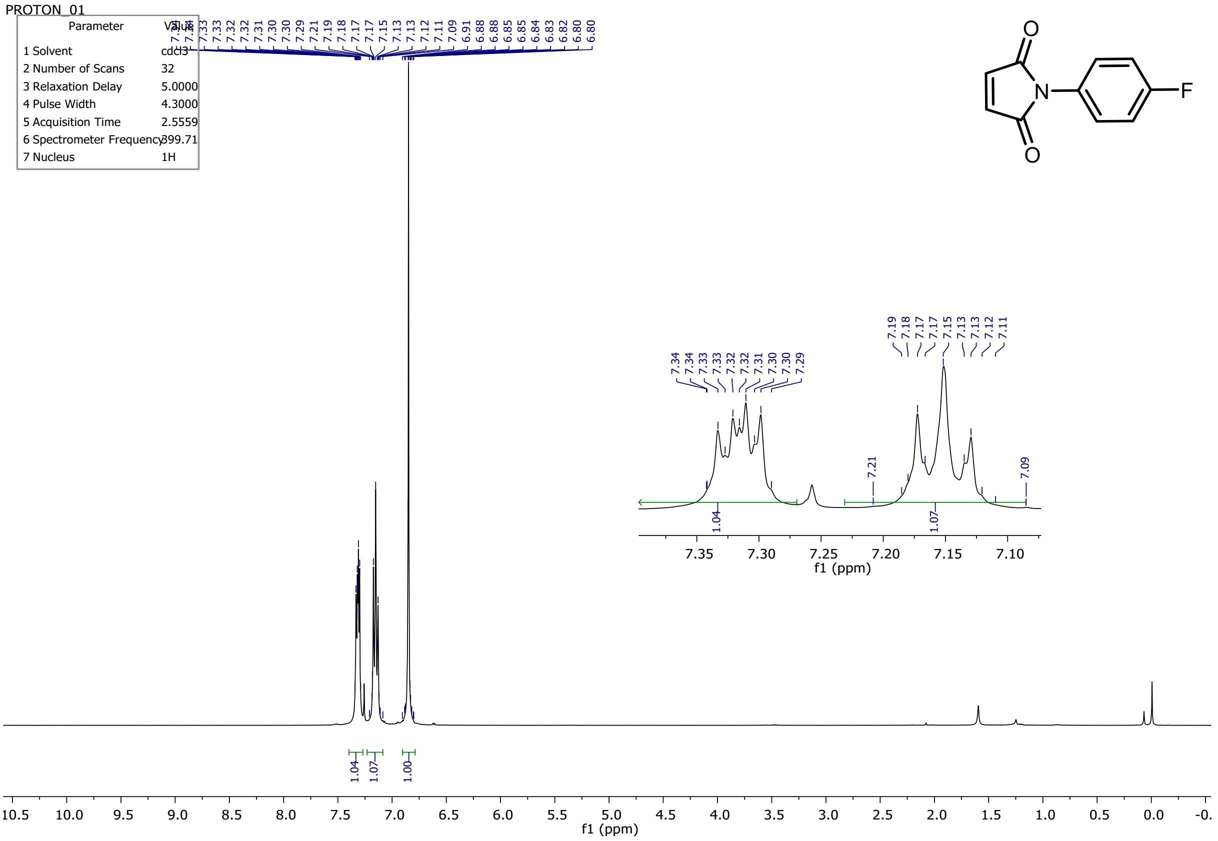

Figure S87. ^{1}H NMR spectrum (400 MHz, $CDCl_3$) of **S6**.

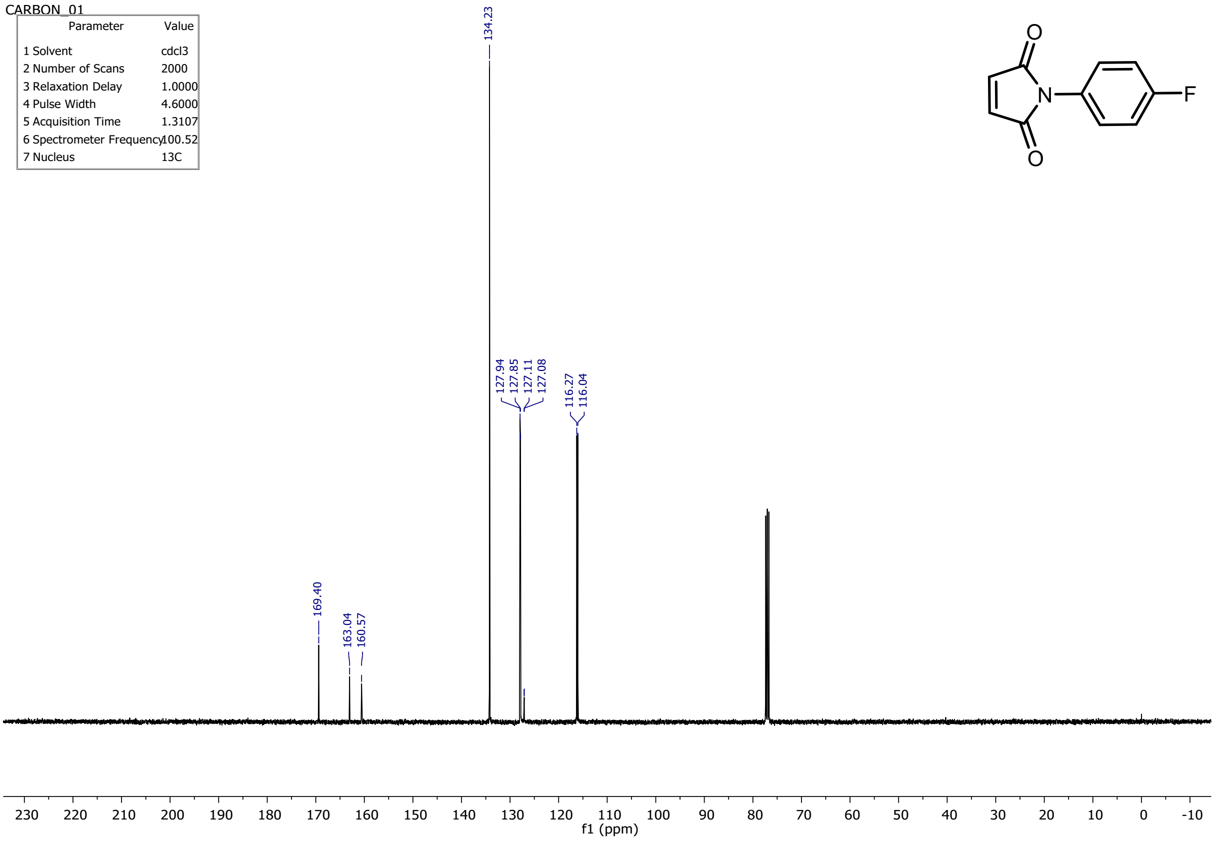

Figure S88. ^{13}C NMR spectrum (101 MHz, $CDCl_3$) of **S6**.

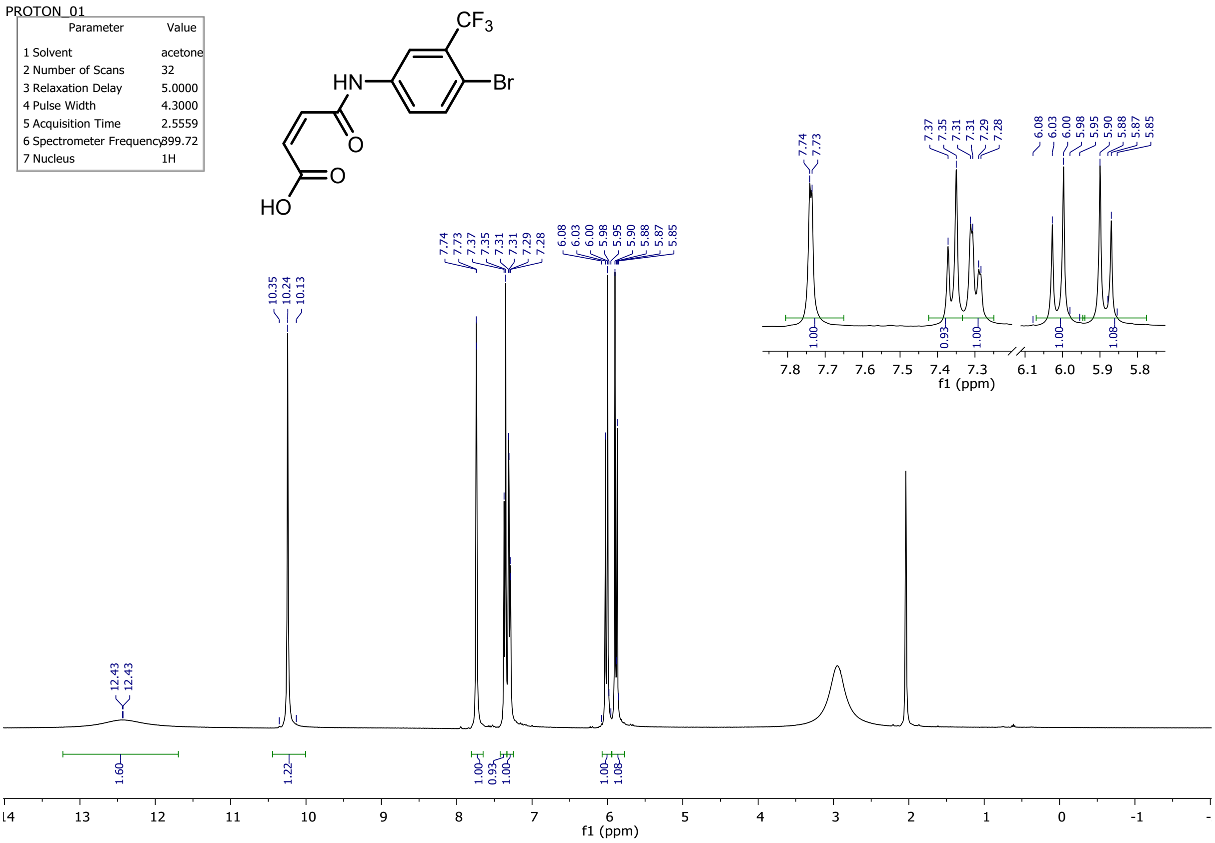

Figure S89. ^{1}H NMR spectrum (400 MHz, acetone-d_6) of **S7**.

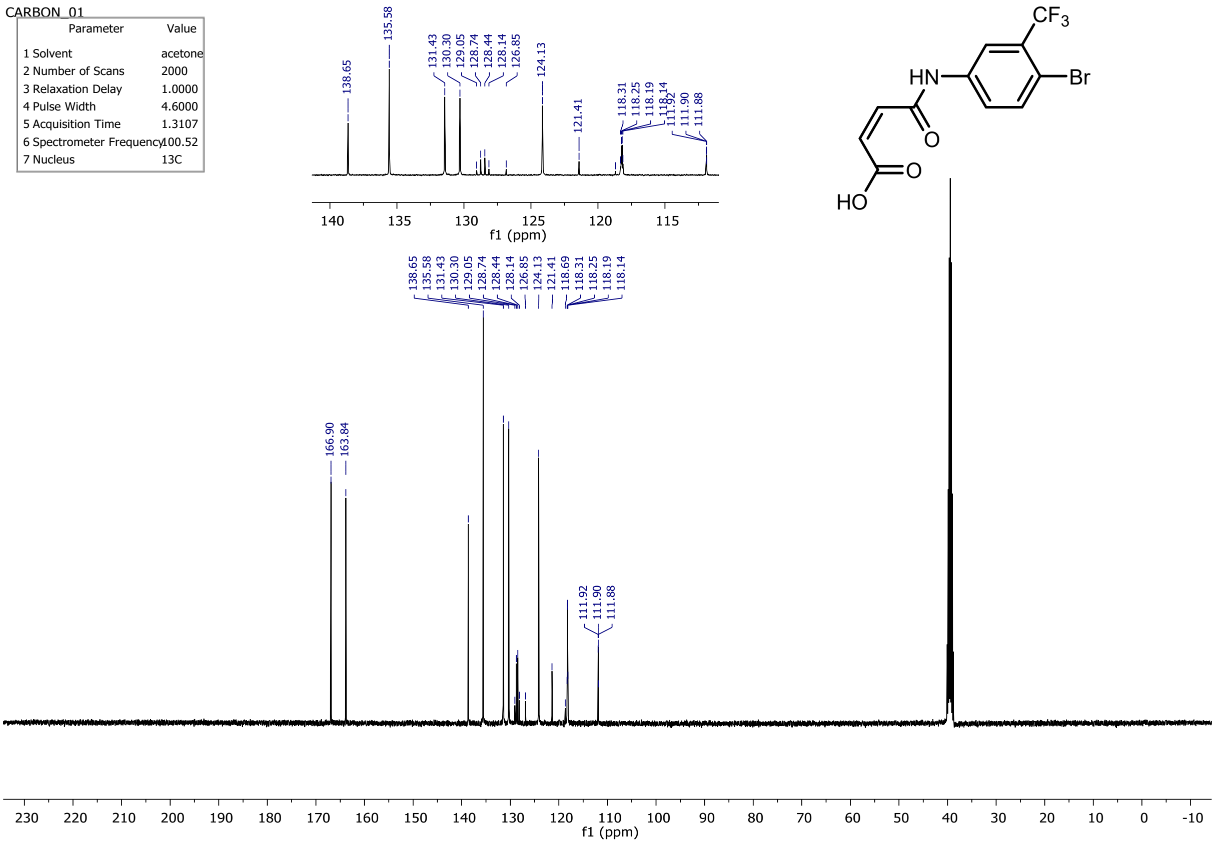

Figure S90. ^{13}C NMR spectrum (101 MHz, acetone-d_6) of **S7**.

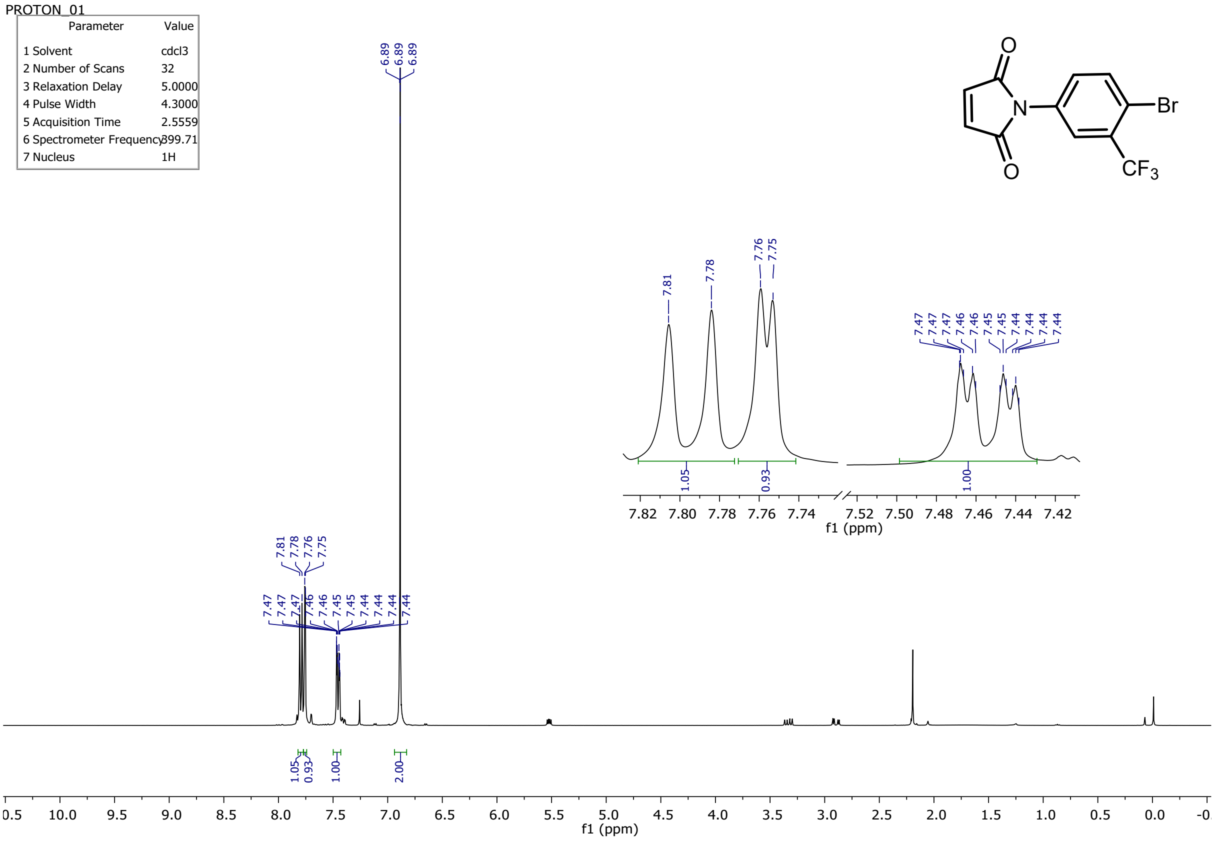

Figure S91. ^{1}H NMR spectrum (400 MHz, $CDCl_3$) of **S8**.

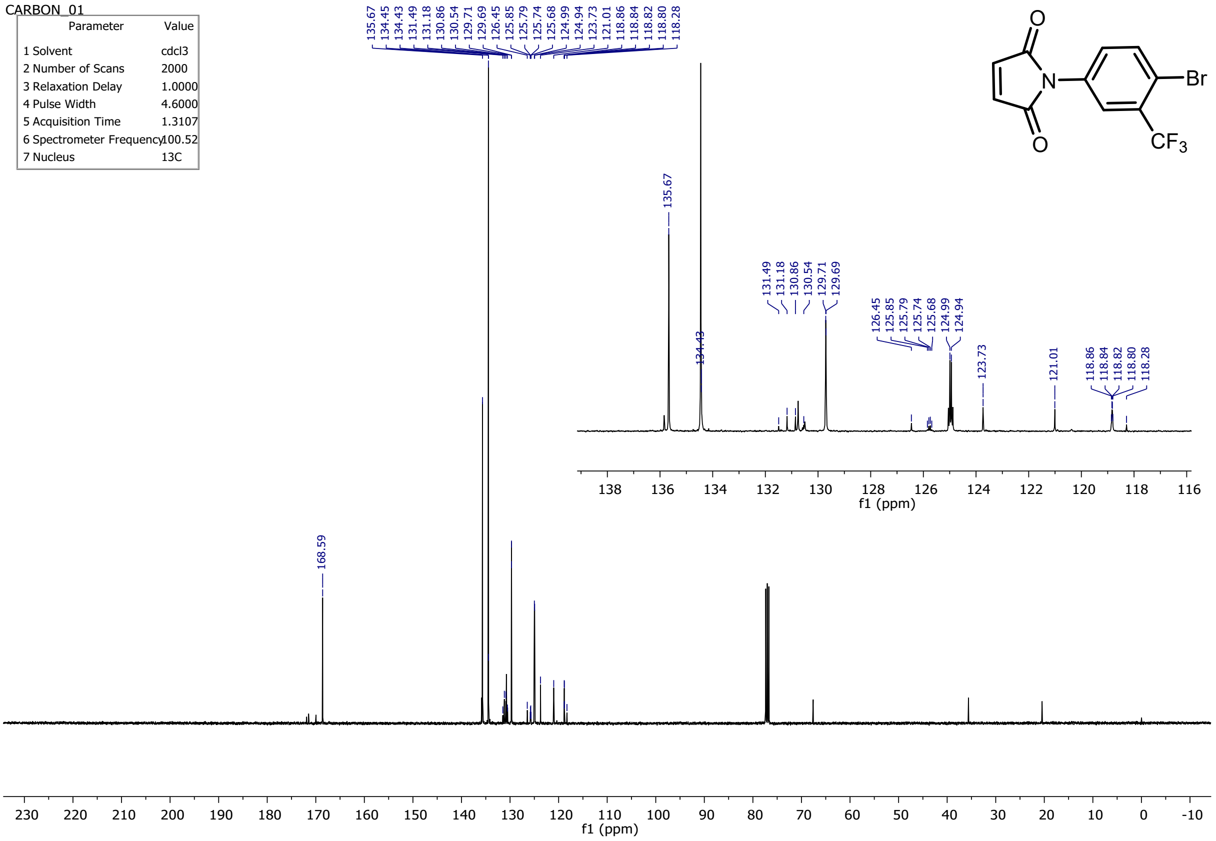

Figure S92. ^{13}C NMR spectrum (101 MHz, $CDCl_3$) of **S8**.

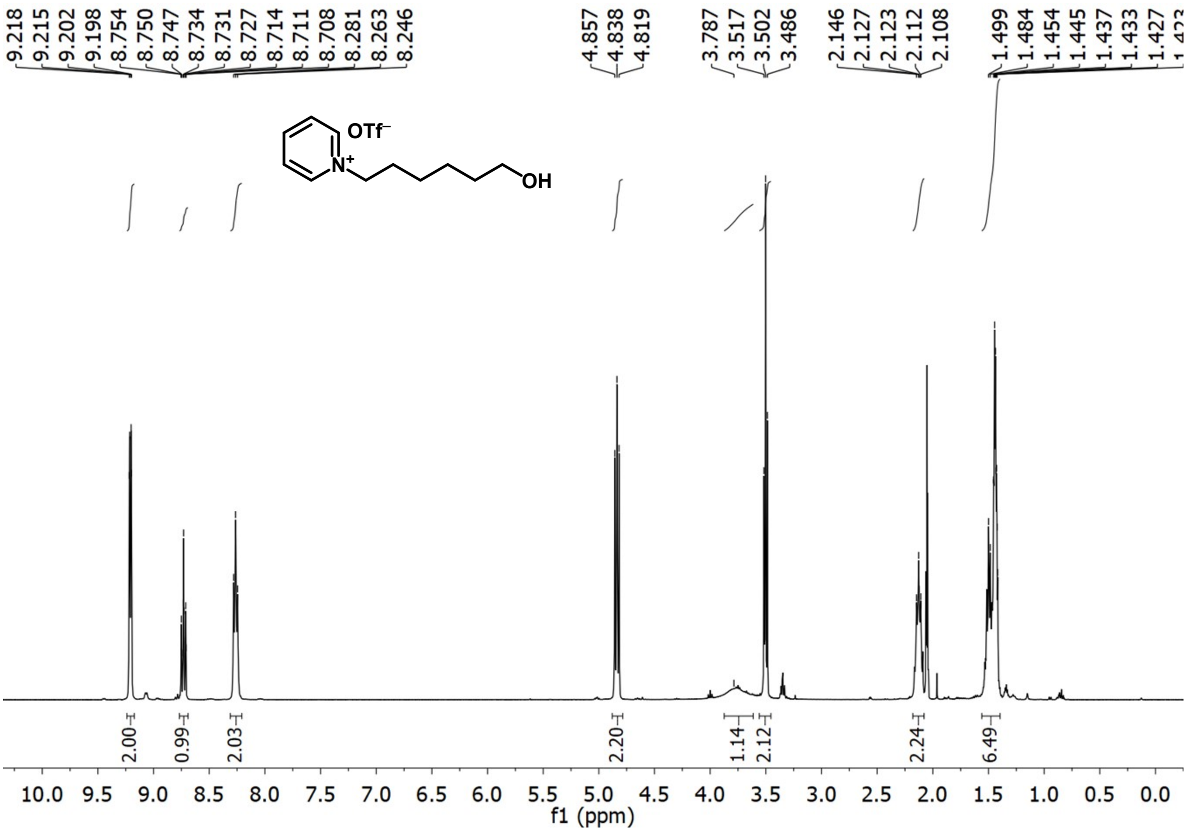

Figure S93. ^{1}H NMR spectrum (400 MHz, DMSO-d_6) of **S9**.

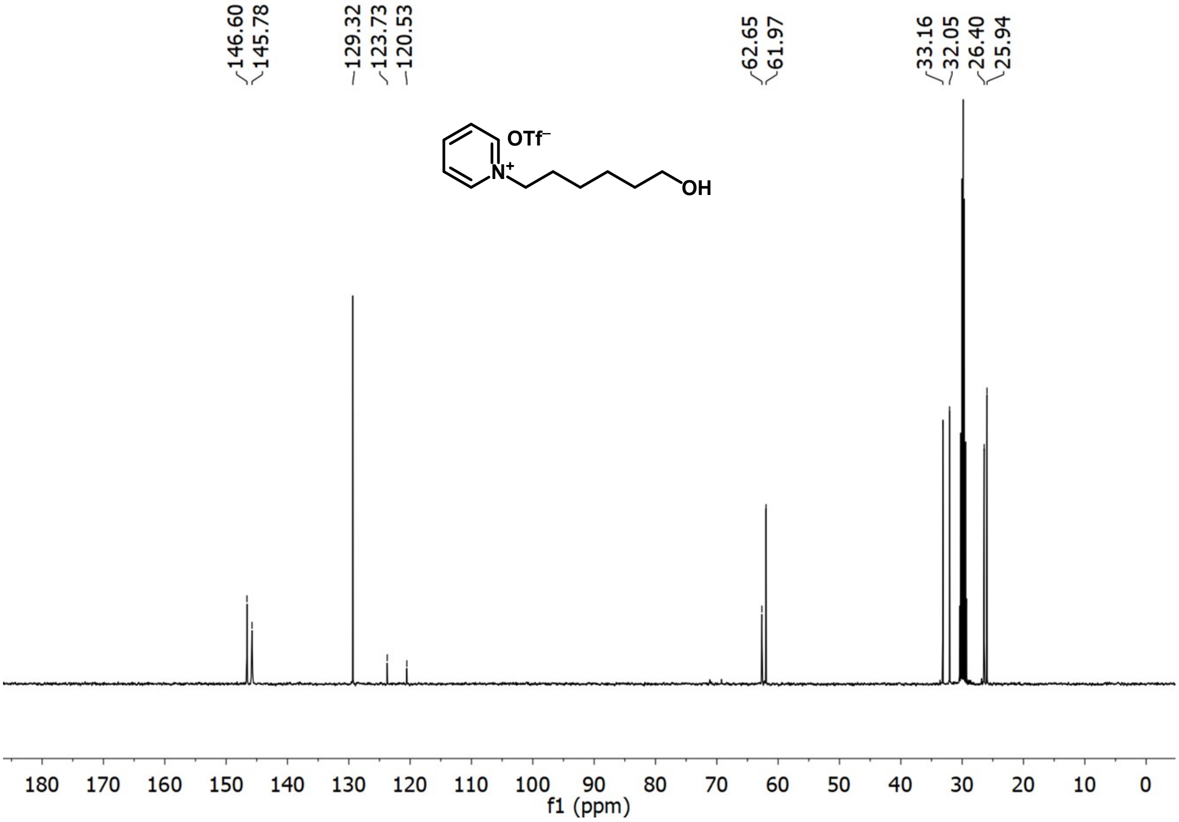

Figure S94. ^{13}C NMR spectrum (101 MHz, DMSO-d_6) of **S9**.

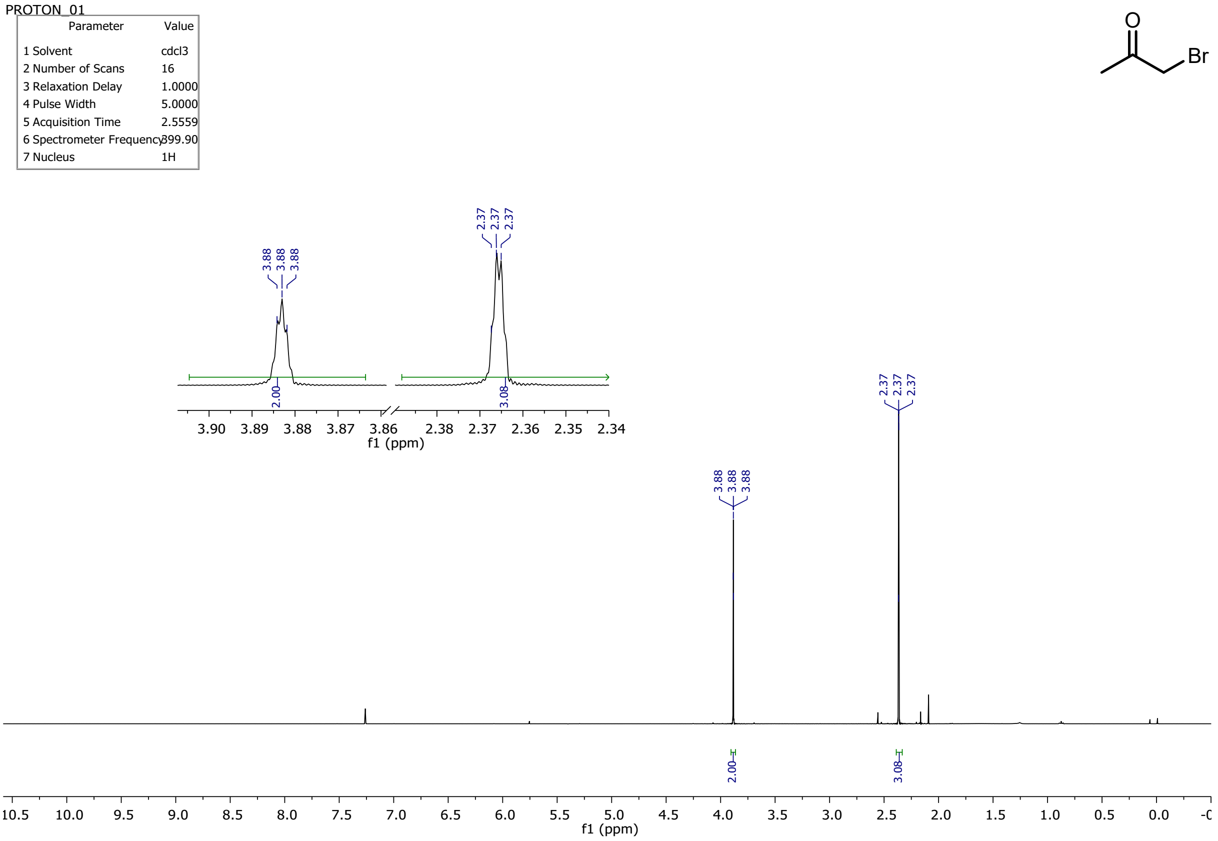

Figure S95. ^{1}H NMR spectrum (400 MHz, $CDCl_3$) of **S10**.

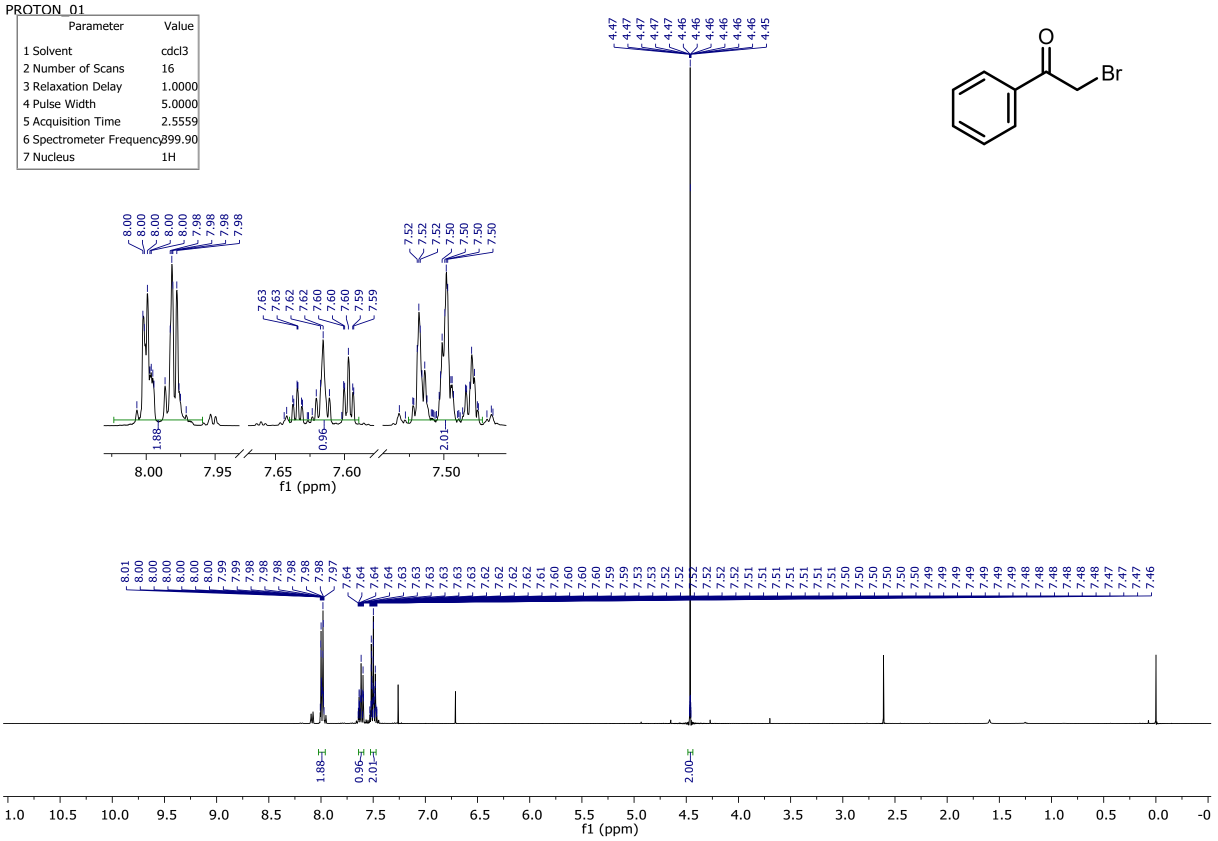

Figure S96. ^{1}H NMR spectrum (400 MHz, $CDCl_3$) of **S11**.

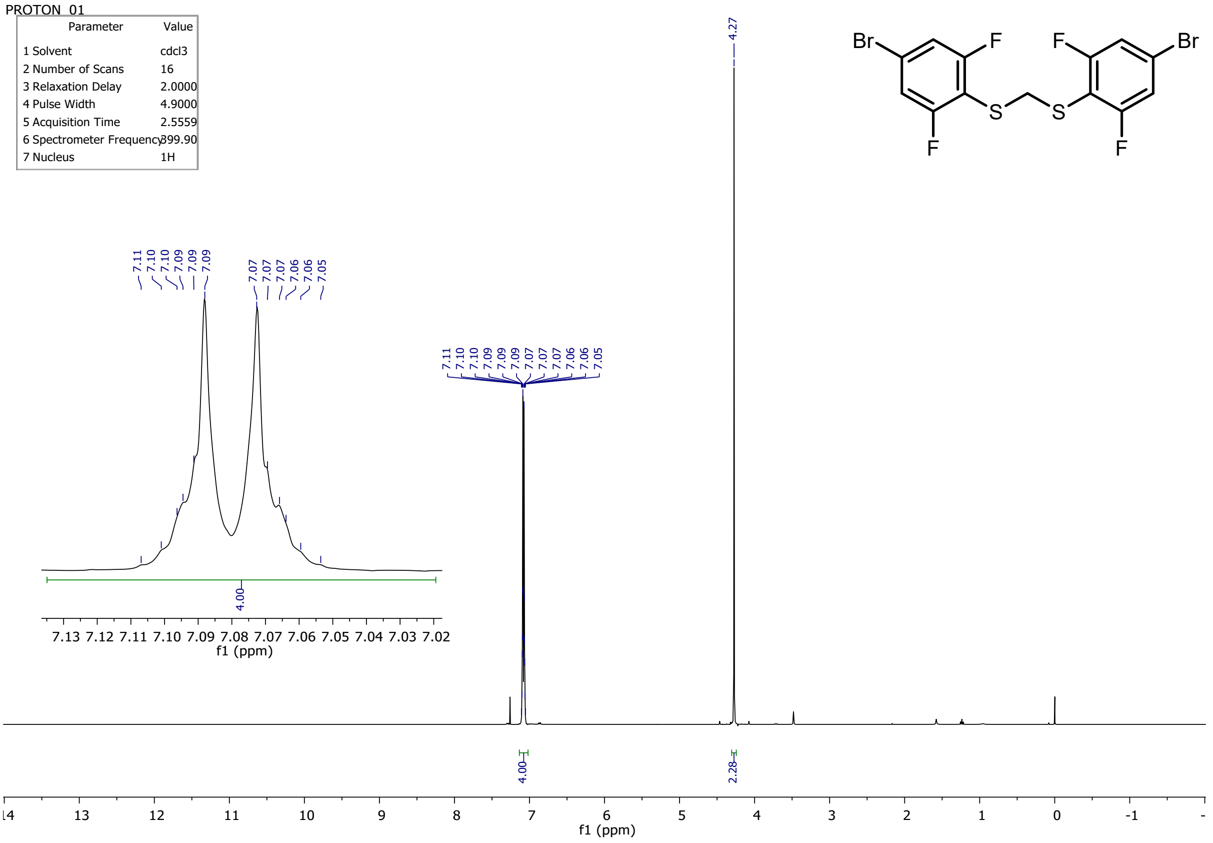

Figure S97. ^{1}H NMR spectrum (400 MHz, $CDCl_3$) of **1b_CH2**.

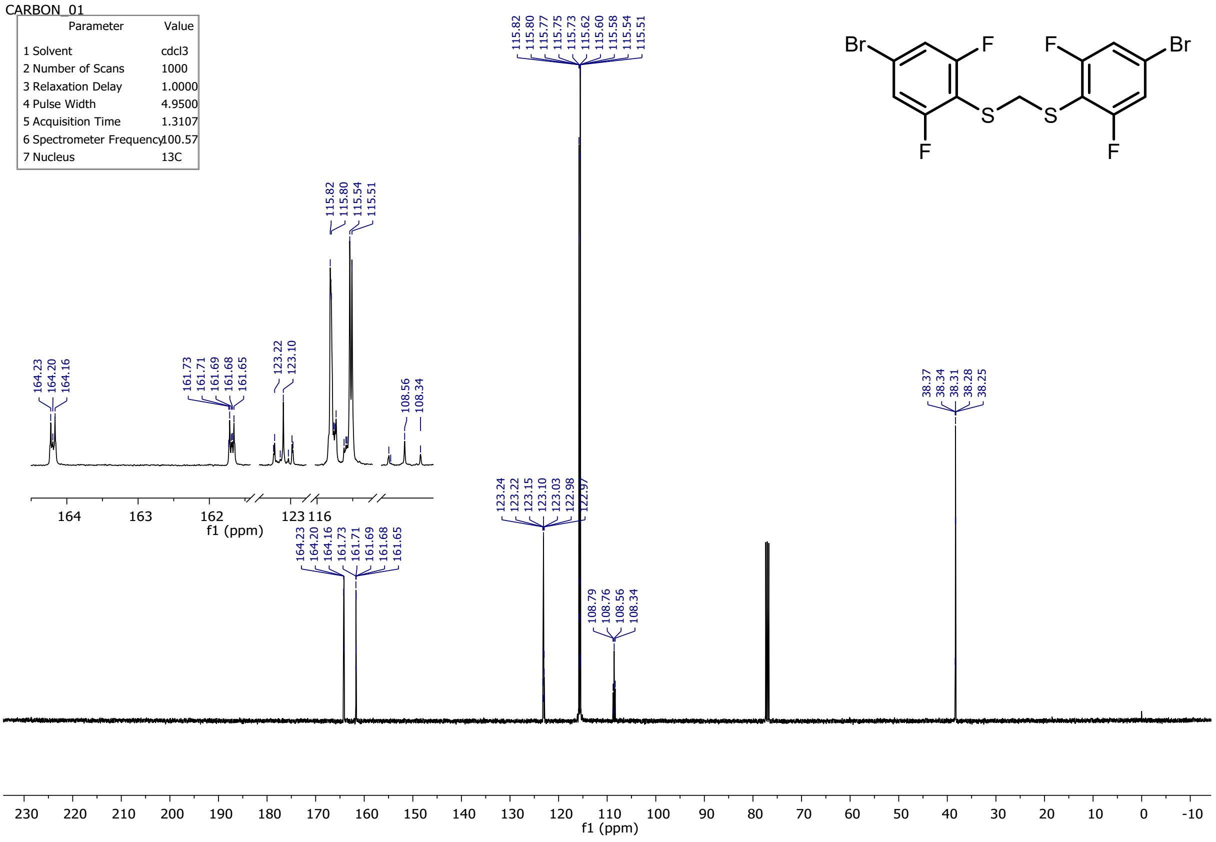

Figure S98. ^{13}C NMR spectrum (101 MHz, $CDCl_3$) of **1b_CH2**.

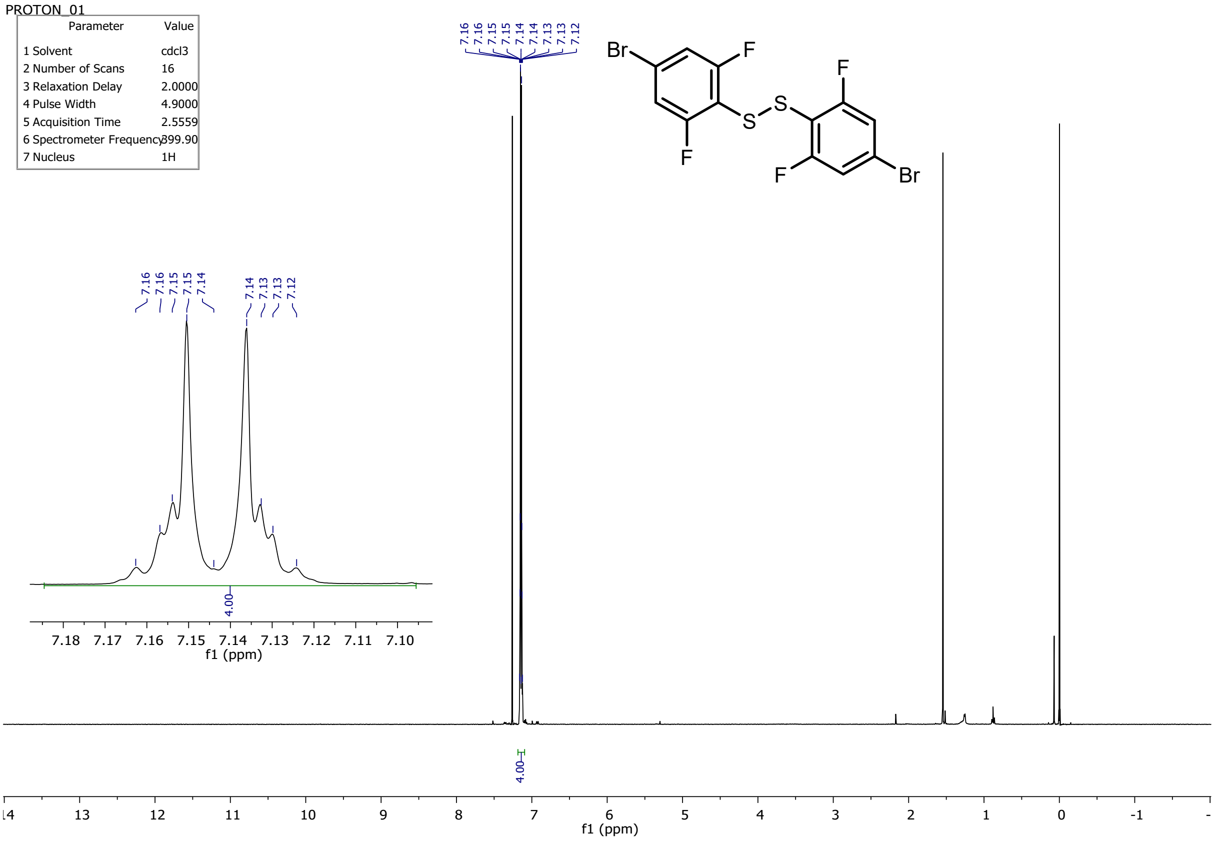

Figure S99. ^{1}H NMR spectrum (400 MHz, $CDCl_3$) of **1b_D**.

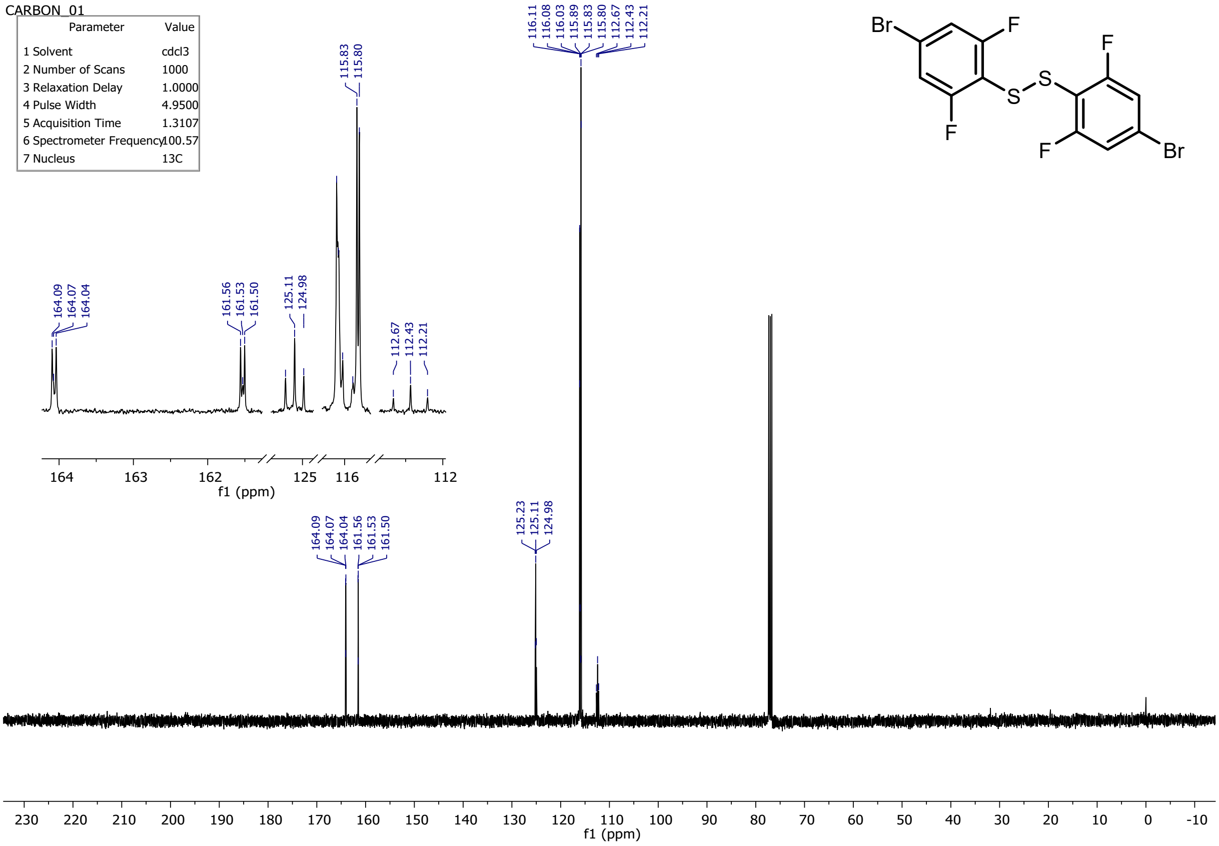

Figure S100. ^{13}C NMR spectrum (101 MHz, $CDCl_3$) of **1b_D**.
